# Supplementary material for: An Easy Synthesis of Monofluorinated Derivatives of Pyrroles from β-Fluoro-β-Nitrostyrenes
Source: Molecules. 2021 Jun 9;26(12):3515. doi: 10.3390/molecules26123515 (PMC8229656; doi:10.3390/molecules26123515)
Supplement: Supplementary file 1 [file molecules-26-03515-s001.zip › molecules-1241666-proof-SM.pdf]

Article

An Easy Synthesis of Monofluorinated Derivatives of Pyrroles from β-Fluoro-β-Nitrostyrenes

Alexander S. Aldoshin [1], Andrey A. Tabolin [2], Sema L. Ioffe [2] and Valentine G. Nenajdenko [1,*]

[1] Department of Chemistry, Lomonosov Moscow State University, Leninskie gory 1, Moscow 119991, Russia; aldon2258@mail.ru

[2] N. D. Zelinsky Institute of Organic Chemistry, Russian Academy of Sciences, Leninsky prosp. 47,
Moscow 119991, Russia; tabolin87@mail.ru (A.A.T.); iof@ioc.ac.ru (S.L.I.)

* Correspondence: nenajdenko@org.chem.msu.ru; Tel.: +7-9258500962

Supplementary information

Table of contents

Kinetic studies **S3**

NMR spectra of compounds obtained **S9**

2-(2-Fluoro-2-nitro-1-phenylethyl)-1*H*-pyrrole (**3a**) S9
2-(2-Fluoro-1-(4-methoxyphenyl)-2-nitroethyl)-1*H*-pyrrole (**3b**) S12
2-(1-(4-(*tert*-Butyl)phenyl)-2-fluoro-2-nitroethyl)-1*H*-pyrrole (**3c**) S15
2-(2-Fluoro-2-nitro-1-(p-tolyl)ethyl)-1H-pyrrole (**3d**) S18
2-(2-Fluoro-1-(4-fluorophenyl)-2-nitroethyl)-1*H*-pyrrole (**3e**) S21
2-(1-(4-Bromophenyl)-2-fluoro-2-nitroethyl)-1*H*-pyrrole (**3f**) S24
2-(1-(4-Chlorophenyl)-2-fluoro-2-nitroethyl)-1*H*-pyrrole (**3g**) S27
2-(1-(2,4-Dichlorophenyl)-2-fluoro-2-nitroethyl)-1*H*-pyrrole (**3h**) S30
2-(2-Fluoro-2-nitro-1-(4-(trifluoromethyl)phenyl)ethyl)-1*H*-pyrrole (**3i**) S33
Methyl 4-(2-fluoro-2-nitro-1-(1*H*-pyrrol-2-yl)ethyl)benzoate (**3j**) S36
4-(2-Fluoro-2-nitro-1-(1*H*-pyrrol-2-yl)ethyl)benzonitrile (**3k**) S39
2-(2-Fluoro-2-nitro-1-(4-nitrophenyl)ethyl)-1*H*-pyrrole (**3l**) S42
2-(2-Fluoro-2-nitro-1-(3-nitrophenyl)ethyl)-1*H*-pyrrole (**3m**) S45
2-(2-Fluoro-2-nitro-1-(2-nitrophenyl)ethyl)-1*H*-pyrrole (**3n**) S48
1,3-Bis(2-fluoro-2-nitro-1-(1*H*-pyrrol-2-yl)ethyl)benzene (**3o**) S51
2-(1-(4-Chlorophenyl)-2-fluoro-2-nitroethyl)-1-methyl-1*H*-pyrrole (**3p**) S54
2-(1-(4-Chlorophenyl)-2-fluoro-2-nitroethyl)-1-phenyl-1*H*-pyrrole (**3q**) S57
2-(1-(4-Chlorophenyl)-2-fluoro-2-nitroethyl)-1-(4-ethylphenyl)-1*H*-pyrrole (**3r**) S60
2-(1-(4-Chlorophenyl)-2-fluoro-2-nitroethyl)-1-(p-tolyl)-1*H*-pyrrole (**3s**) S64
1-(3-Chloro-4-methoxyphenyl)-2-(1-(4-chlorophenyl)-2-fluoro-2-nitroethyl)-1*H*-pyrrole (**3t**) S67
2-(1-(4-Chlorophenyl)-2-fluoro-2-nitroethyl)-1-(3-methoxyphenyl)-1*H*-pyrrole (**3u**) S70
2-(2-Fluoro-1-phenylvinyl)-1*H*-pyrrole (**4a**) S73
2-(2-Fluoro-1-(4-methoxyphenyl)vinyl)-1*H*-pyrrole (**4b**) S76
2-(1-(4-(*tert*-Butyl)phenyl)-2-fluorovinyl)-1*H*-pyrrole (**4c**) S79
(*Z*)-2-(2-Fluoro-1-(p-tolyl)vinyl)-1*H*-pyrrole (*Z*-**4d**) S82
(*E*)-2-(2-Fluoro-1-(p-tolyl)vinyl)-1*H*-pyrrole (*E*-**4d**) S85
2-(2-Fluoro-1-(4-fluorophenyl)vinyl)-1*H*-pyrrole (**4e**) S88
(*Z*)-2-(1-(4-Bromophenyl)-2-fluorovinyl)-1*H*-pyrrole (*Z*-**4f**) S91

(*E*)-2-(1-(2,4-Dichlorophenyl)-2-fluorovinyl)-1*H*-pyrrole (*E*-**4f**) S94
(*Z*)-2-(1-(4-Chlorophenyl)-2-fluorovinyl)-1*H*-pyrrole (*Z*-**4g**) S97
(*E*)-2-(1-(4-Chlorophenyl)-2-fluorovinyl)-1*H*-pyrrole (*E*-**4g**) S100
(*Z*)-2-(1-(2,4-Dichlorophenyl)-2-fluorovinyl)-1*H*-pyrrole (*Z*-**4h**) S103
(*E*)-2-(1-(2,4-Dichlorophenyl)-2-fluorovinyl)-1*H*-pyrrole (*E*-**4h**) S106
(*Z*)-2-(2-Fluoro-1-(4-(trifluoromethyl)phenyl)vinyl)-1*H*-pyrrole (*Z*-**4i**) S109
(*E*)-2-(2-Fluoro-1-(4-(trifluoromethyl)phenyl)vinyl)-1*H*-pyrrole (*E*-**4i**) S113
(*Z*)-Methyl 4-(2-fluoro-1-(1*H*-pyrrol-2-yl)vinyl)benzoate (Z-**4j**) S116
(*E*)-Methyl 4-(2-fluoro-1-(1*H*-pyrrol-2-yl)vinyl)benzoate (*E*-**4j**) S119
4-(2-Fluoro-1-(1*H*-pyrrol-2-yl)vinyl)benzonitrile (**4k**) S122
(*Z*)-2-(2-Fluoro-1-(4-nitrophenyl)vinyl)-1*H*-pyrrole (*Z*-**4l**) S125
(*E*)-2-(2-Fluoro-1-(4-nitrophenyl)vinyl)-1*H*-pyrrole (*E*-**4l**) S128
(*Z*)-2-(2-Fluoro-1-(3-nitrophenyl)vinyl)-1*H*-pyrrole (*Z*-**4m**) S131
(*E*)-2-(2-Fluoro-1-(3-nitrophenyl)vinyl)-1*H*-pyrrole (*E*-**4m**) S134
1,3-Bis((*Z*)-2-fluoro-1-(1*H*-pyrrol-2-yl)vinyl)benzene (*Z*,*Z*-**4o**) S137
1-((*E*)-2-Fluoro-1-(1*H*-pyrrol-2-yl)vinyl)-3-((*Z*)-2-fluoro-1-(1*H*-pyrrol-2-yl)vinyl)benzene (*E*,*Z*-**4o**) S140
1,3-Bis((*E*)-2-fluoro-1-(1*H*-pyrrol-2-yl)vinyl)benzene (*E*,*E*-**4o**) S143
2-(1-(4-Chlorophenyl)-2-fluorovinyl)-1-methyl-1*H*-pyrrole (**4p**) S146
2-(1-(4-Chlorophenyl)-2-fluorovinyl)-1-phenyl-1*H*-pyrrole (**4q**) S150
2-(1-(4-Chlorophenyl)-2-fluorovinyl)-1-(4-ethylphenyl)-1*H*-pyrrole (**4r**) S154
2-(1-(4-Chlorophenyl)-2-fluorovinyl)-1-(p-tolyl)-1*H*-pyrrole (**4s**) S157
1-(3-Chloro-4-methoxyphenyl)-2-(1-(4-chlorophenyl)-2-fluorovinyl)-1*H*-pyrrole (**4t**) S161
2-(1-(4-Chlorophenyl)-2-fluorovinyl)-1-(3-methoxyphenyl)-1*H*-pyrrole (**4u**) S165
2-(2-Nitro-1-phenylvinyl)-1*H*-pyrrole (**5a**) S168
(*Z*)-2-(1-(4-Methoxyphenyl)-2-nitrovinyl)-1*H*-pyrrole (*Z*-**5b**) S170
2-(2-Nitro-1-(p-tolyl)vinyl)-1*H*-pyrrole (**5d**) S172
(*Z*)-2-(1-(4-Fluorophenyl)-2-nitrovinyl)-1*H*-pyrrole (*Z*-**5e**) S174
(*Z*)-2-(1-(4-Bromophenyl)-2-nitrovinyl)-1*H*-pyrrole (*Z*-**5f**) S177
(*Z*)-2-(1-(4-Chlorophenyl)-2-nitrovinyl)-1*H*-pyrrole (*Z*-**5g**) S179
2-(1-(2,4-Dichlorophenyl)-2-nitrovinyl)-1*H*-pyrrole (**5h**) S181
(*Z*)-2-(2-Nitro-1-(4-(trifluoromethyl)phenyl)vinyl)-1*H*-pyrrole (*Z*-**5i**) S183
Methyl 4-(2-nitro-1-(1*H*-pyrrol-2-yl)vinyl)benzoate (**5j**) S186
4-(2-Nitro-1-(1*H*-pyrrol-2-yl)vinyl)benzonitrile (**5k**) S188
2-(2-Nitro-1-(3-nitrophenyl)vinyl)-1*H*-pyrrole (**5m**) S190
2-(1-(3-((Z)-2-Fluoro-1-(1*H*-pyrrol-2-yl)vinyl)phenyl)-2-nitrovinyl)-1*H*-pyrrole (*Z*-F-**5o**) S192
2-(1-(3-((*E*)-2-Fluoro-1-(1*H*-pyrrol-2-yl)vinyl)phenyl)-2-nitrovinyl)-1*H*-pyrrole (*E*-F-**5o**) S195
2-(1-(4-Chlorophenyl)-2-nitrovinyl)-1-methyl-1*H*-pyrrole (**5p**) S198
2-(2-Nitro-1-(p-tolyl)vinyl)-1*H*-pyrrole (**5s**) S200
1-(3-Chloro-4-methoxyphenyl)-2-(1-(4-chlorophenyl)-2-nitrovinyl)-1*H*-pyrrole (**5t**) S202
2-(1-(4-Chlorophenyl)-2-nitrovinyl)-1-(3-methoxyphenyl)-1*H*-pyrrole (**5u**) S204
5,5'-((4-Chlorophenyl)methylene)bis(2-(2-fluoro-2-nitro-1-(4-nitrophenyl)ethyl)-1*H*-pyrrole) (**6**) S206

1. Kinetic studies

Experimental procedure. In a typical kinetic experiment, nitrostyrene **1** (0.1 mmol; 1 mol. equiv.) was loaded a screw-top vial. After 1*H*-pyrrole (1.5 mL; 216 mol. equiv.) was added and the reaction mixtures were heated in a thermostat at the preset temperature (30 – 90 °C) with vigorous stirring for the set time (0.5-40 h). Samples of the reaction were taken at regular intervals and analyzed by ^{19}F NMR in $CDCl_3$ (^{1}H NMR in case of non-fluorinated nitrostyrenes). A ratio of products **3** and conversions (*F*) of nitrostyrenes **1** were calculated from integral values of corresponding characteristic peaks.

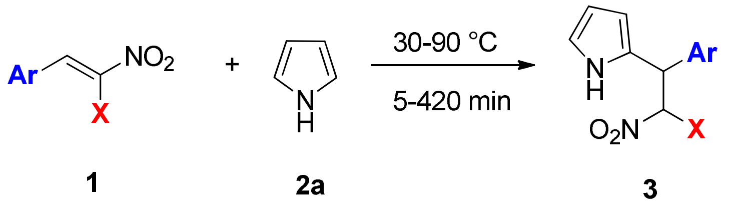

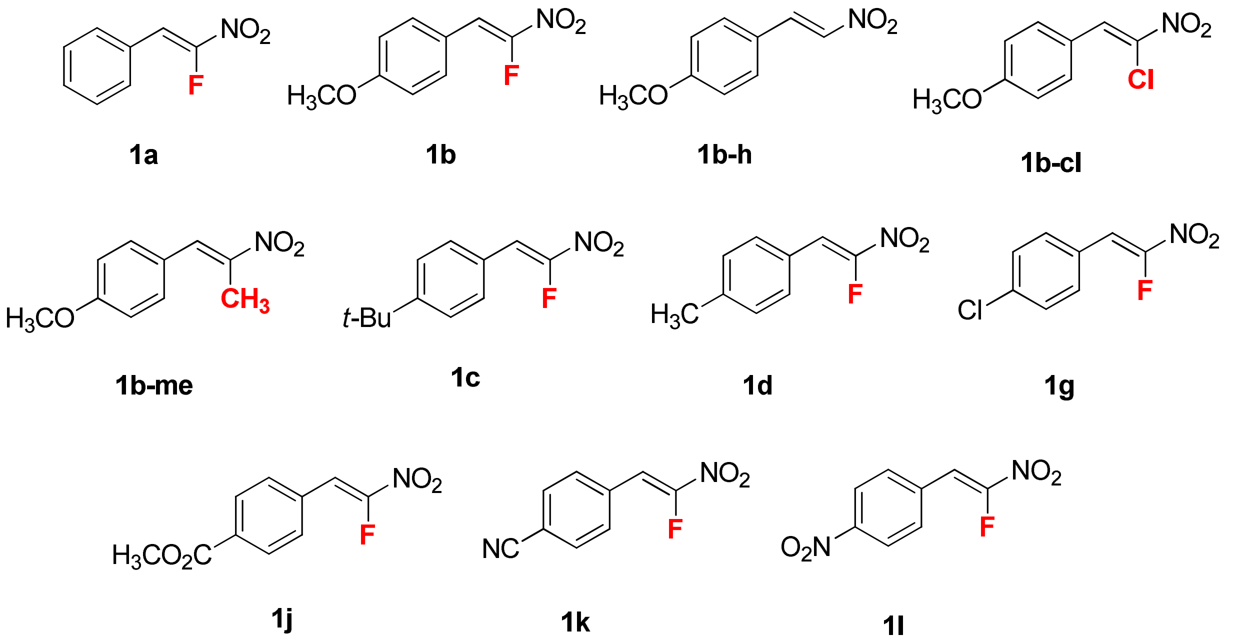

Figure S1. A series of nitrostyrenes **1** for kinetic studies

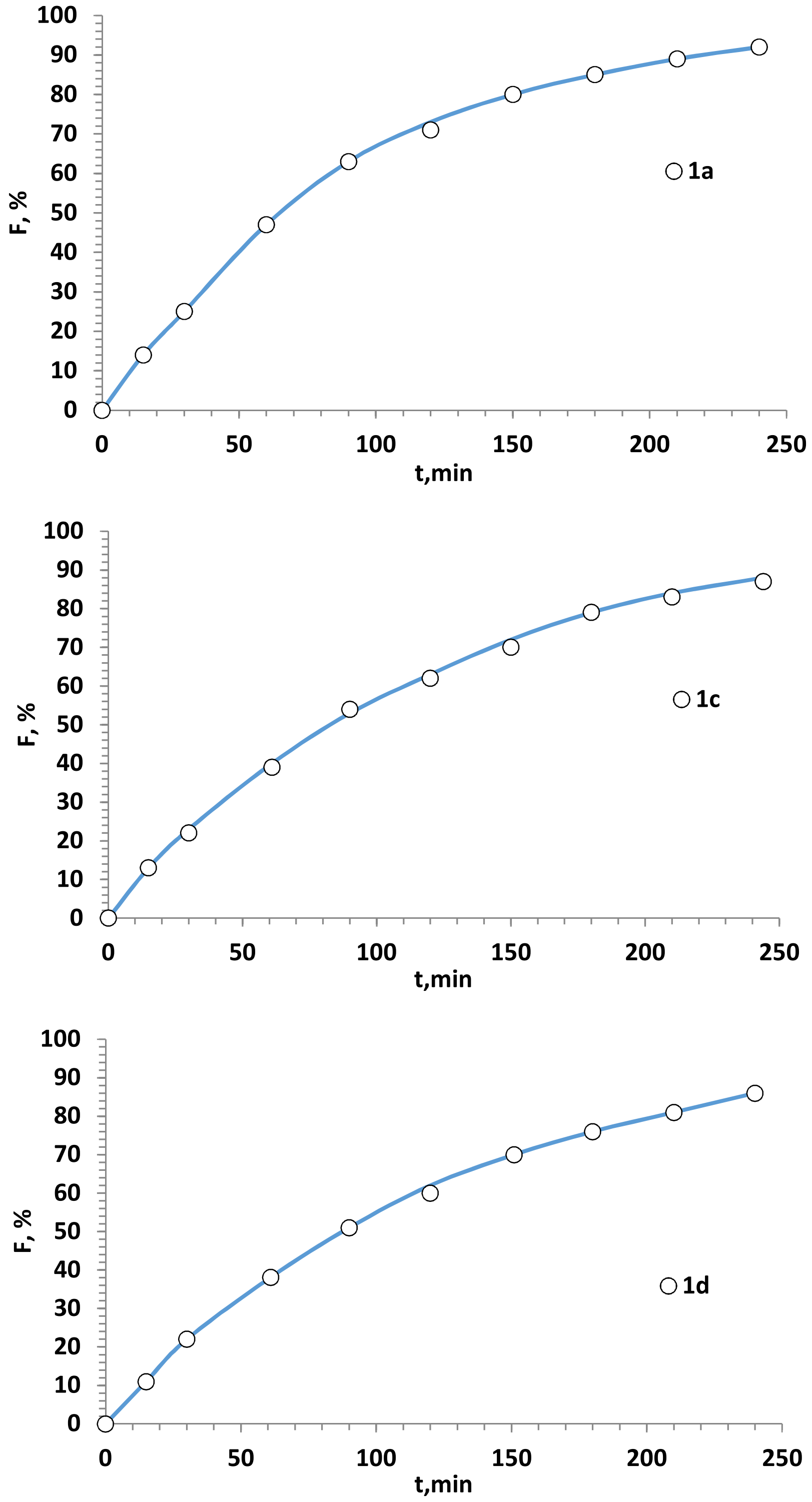

Figure S2. Kinetic curves of the reaction of nitrostyrenes **1** with pyrrole at 50 °C

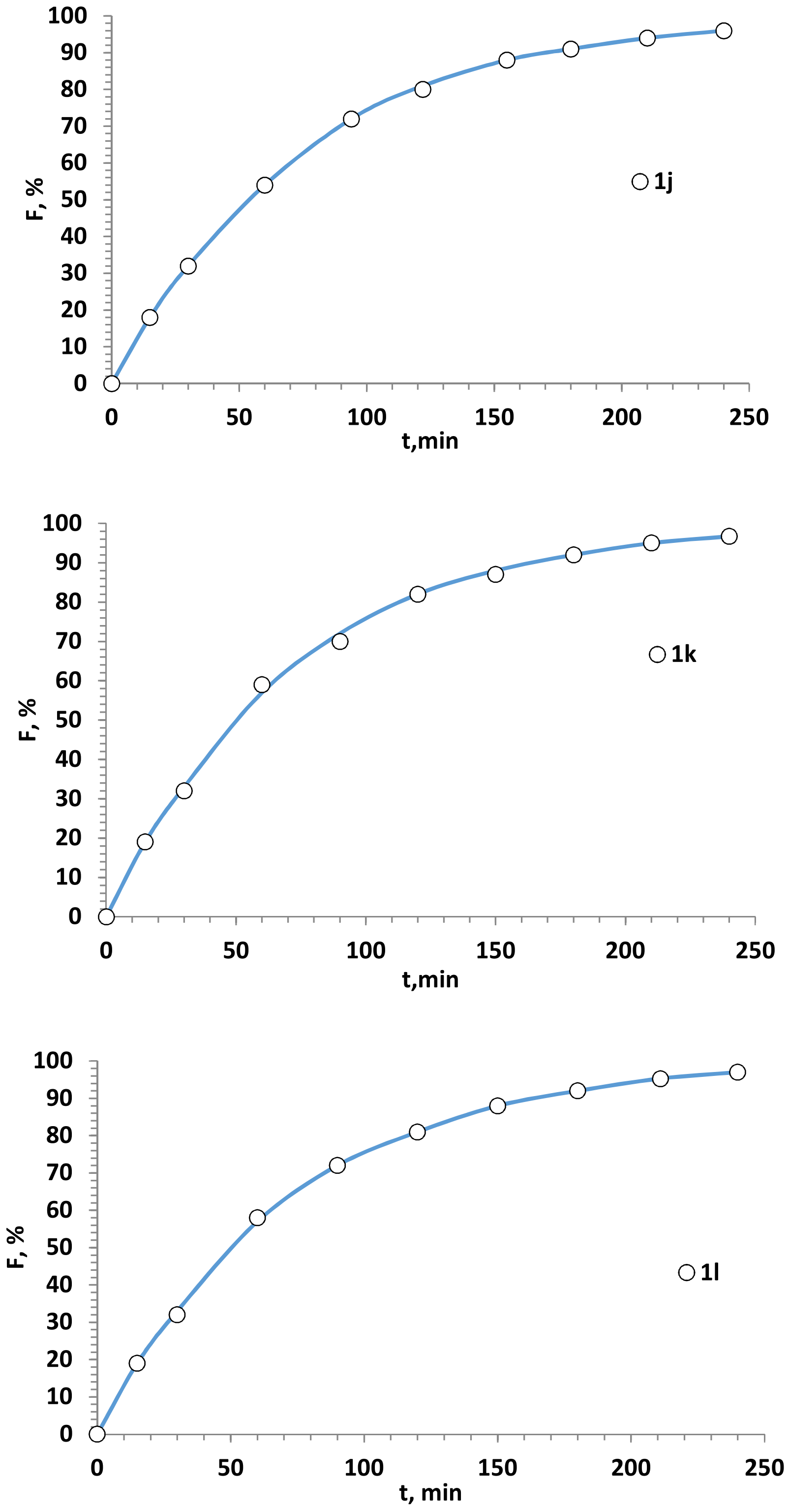

Figure S3. Kinetic curves of the reaction of nitrostyrenes **1** with pyrrole at 50 °C

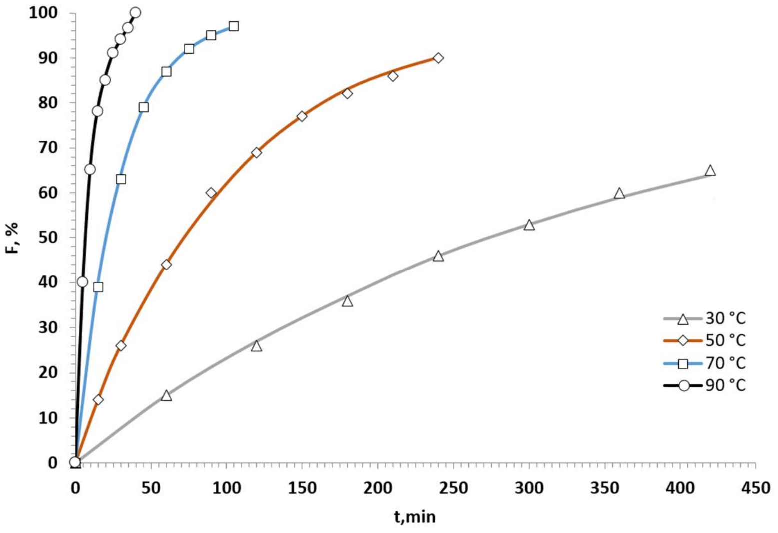

Figure S4. Kinetic curves of the reaction of **1h** with pyrrole at 30-90 °C

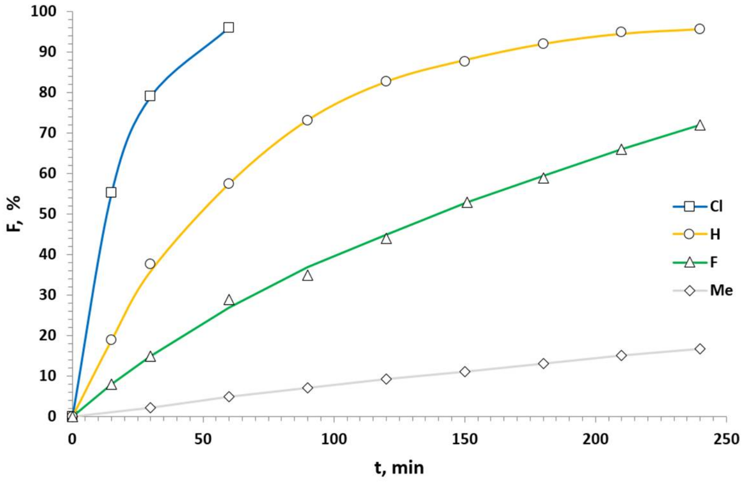

Figure S5. Kinetic curves of the reaction differently β-substituted nitrostyrenes **1b** with pyrrole at 50 °C

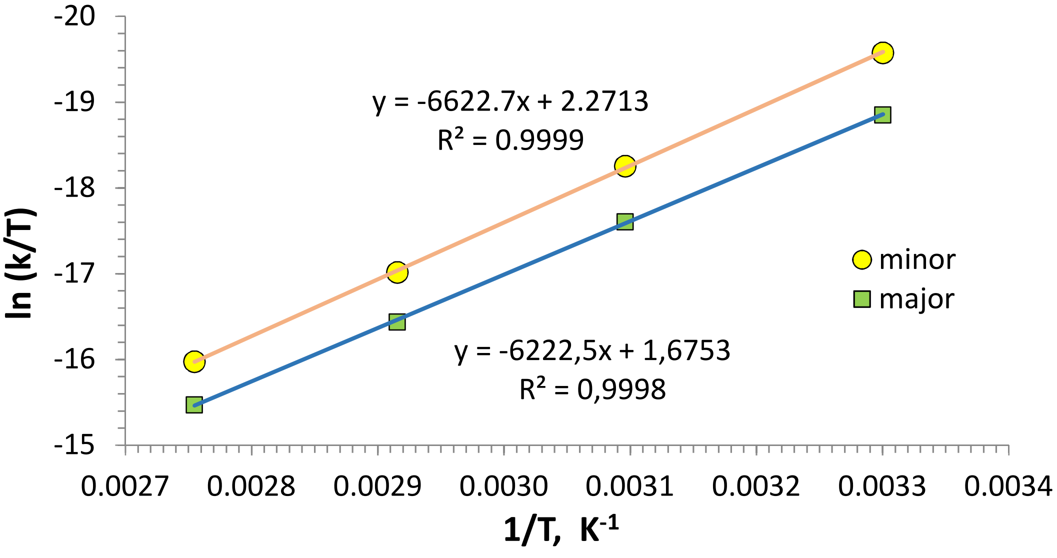

Figure S6. Plot of ln (k/T) vs. 1/T for reaction **1g** with pyrrole

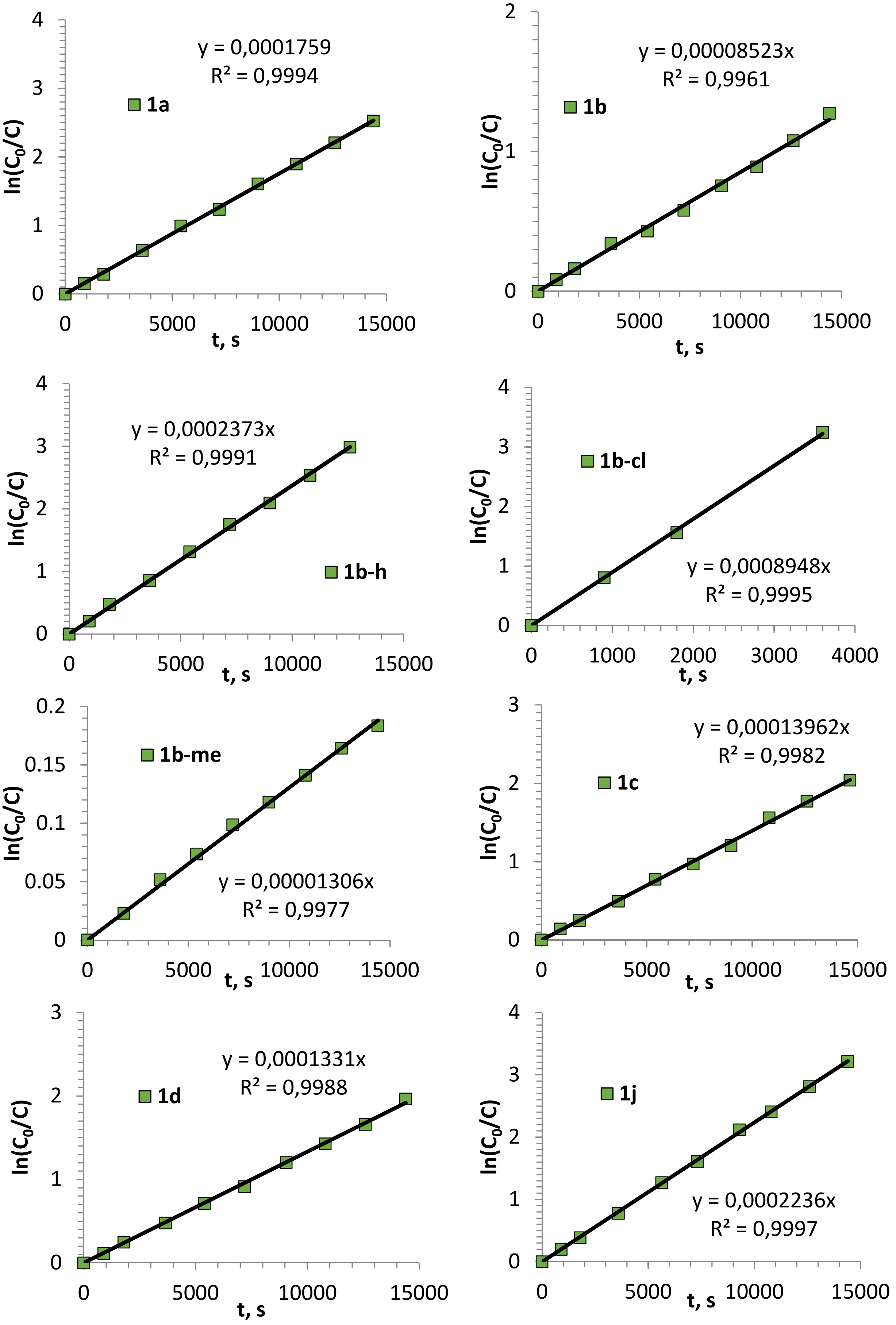

Figure S6. Plot of ln (C_0/C) vs. t for reaction nitrostyrenes **1** with pyrrole at 50 °C

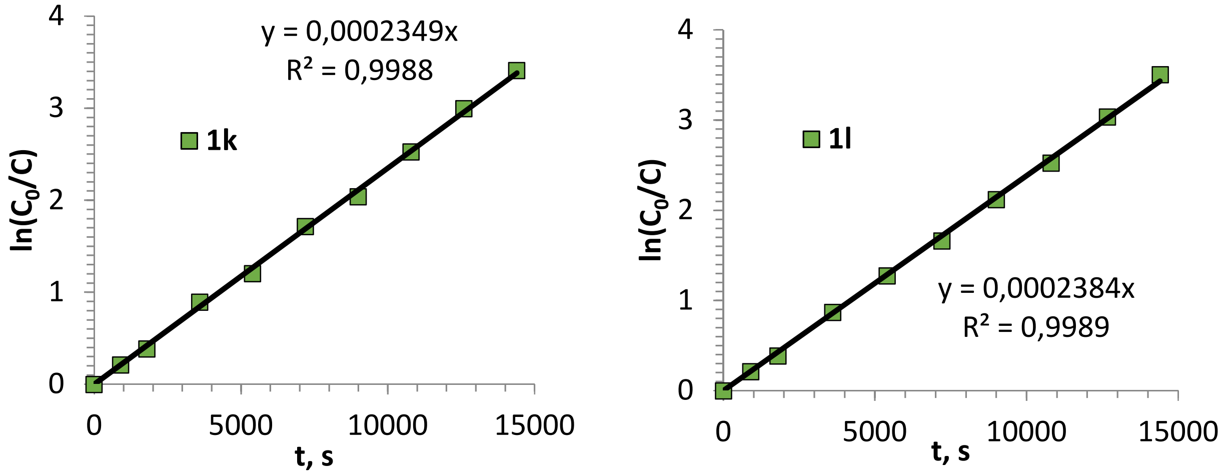

Figure S7. Plot of ln (C_0/C) vs. t for reaction nitrostyrenes **1** with pyrrole at 50 °C

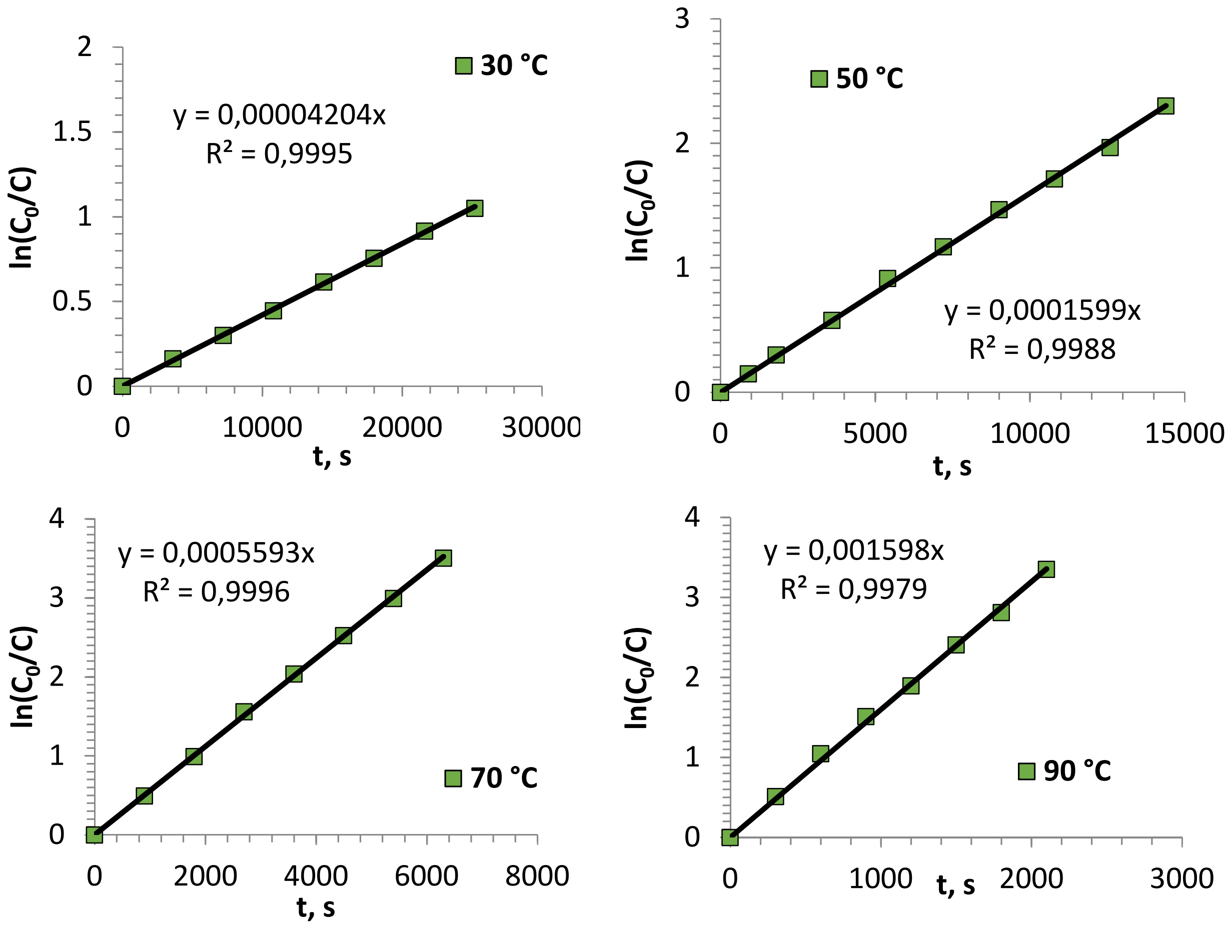

Figure S8. Plot of ln (C_0/C) vs. t for reaction nitrostyrenes **1g** with pyrrole at 50-90 °C

2. NMR spectra of compounds obtained

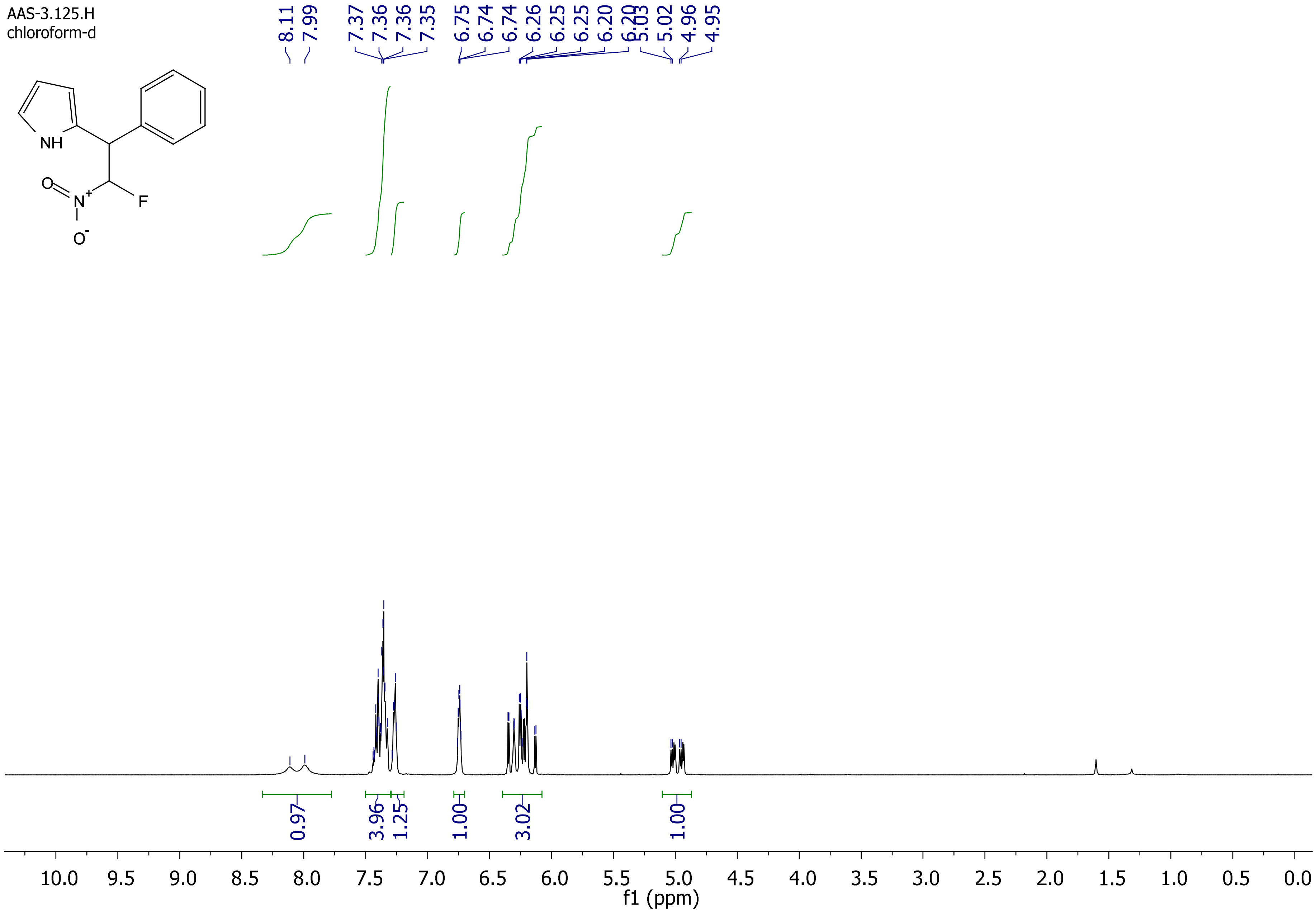

^{1}H NMR spectrum of 2-(2-fluoro-2-nitro-1-phenylethyl)-1*H*-pyrrole (**3a**)

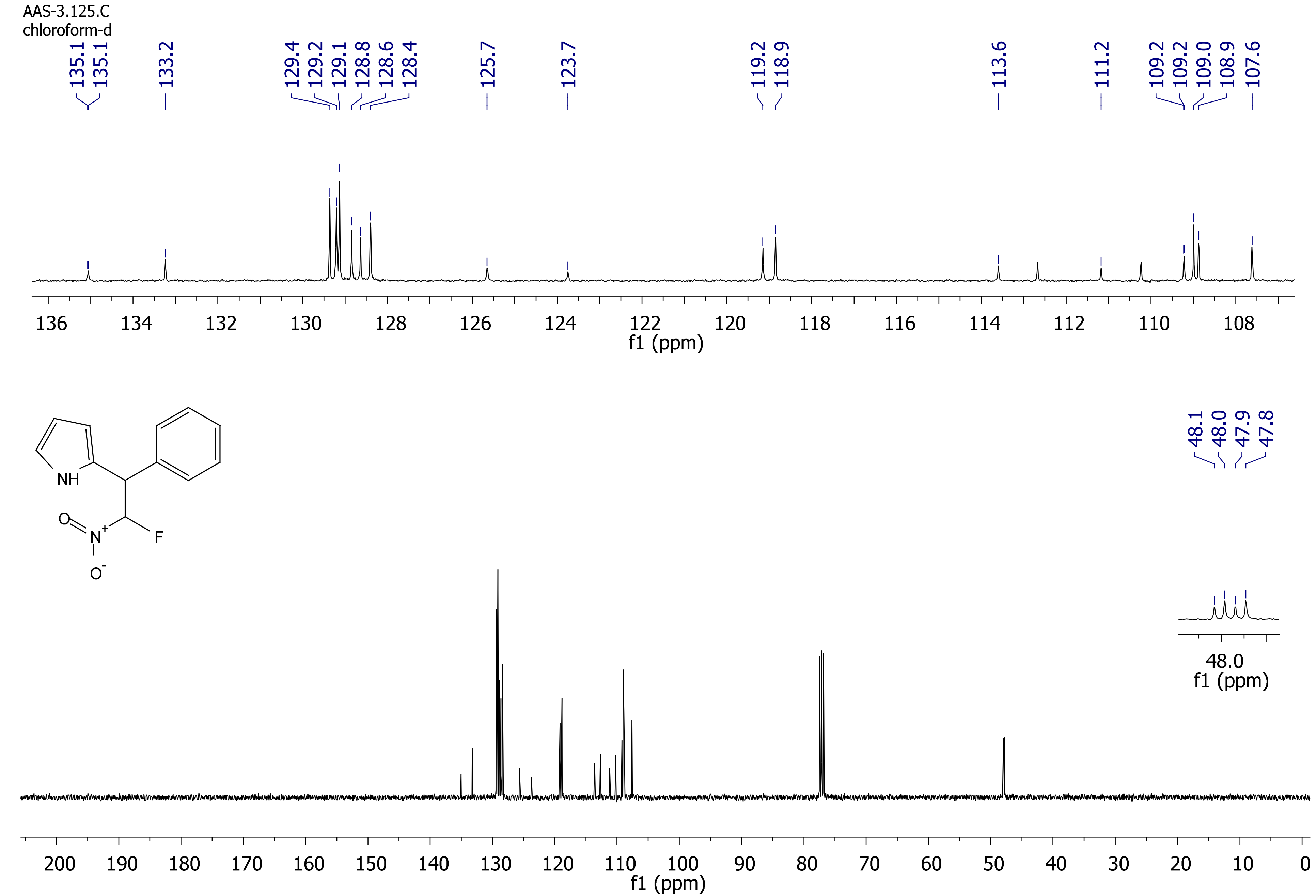

^{13}C NMR spectrum of 2-(2-fluoro-2-nitro-1-phenylethyl)-1*H*-pyrrole (**3a**)

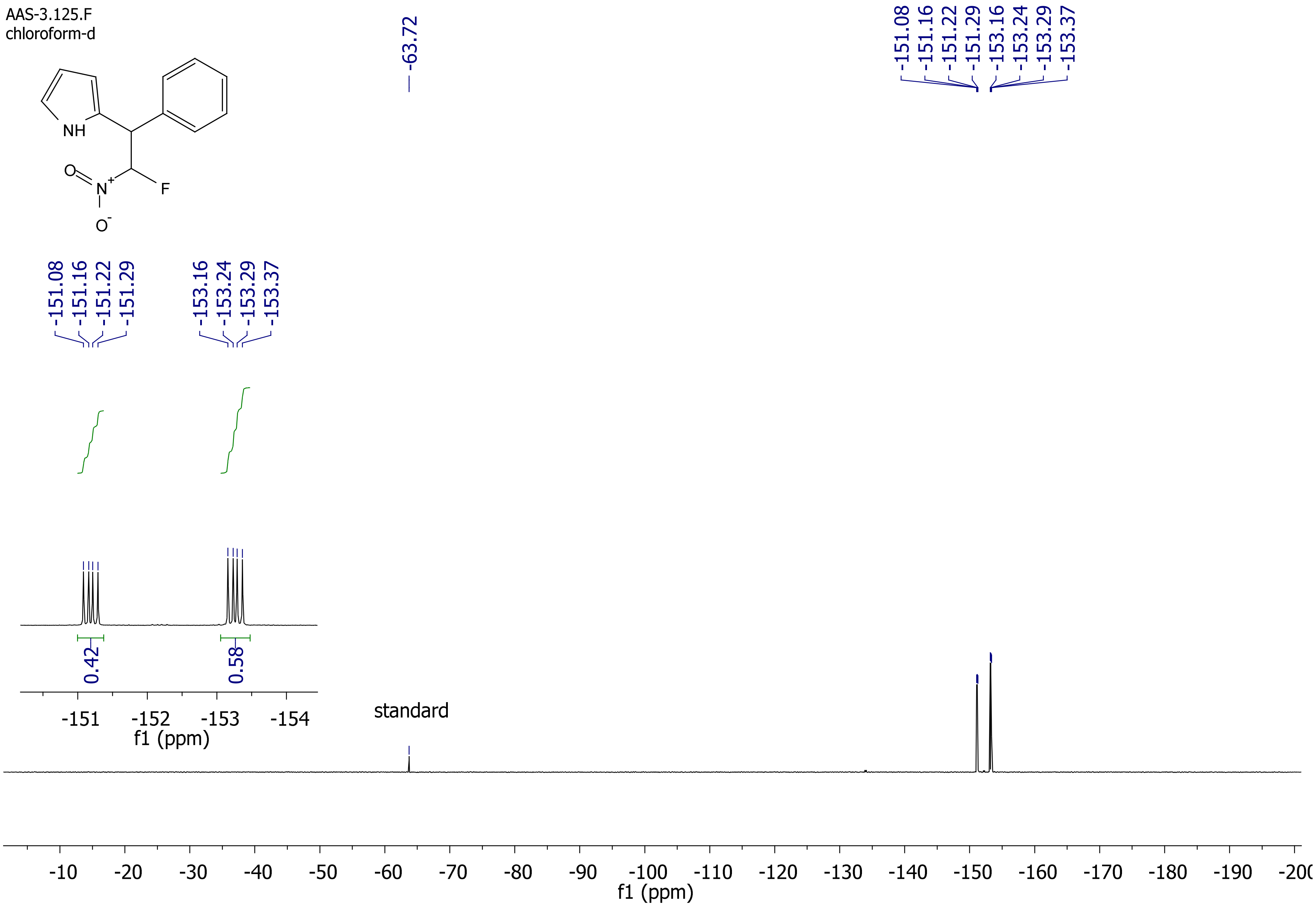

^{19}F NMR spectrum of of 2-(2-fluoro-2-nitro-1-phenylethyl)-1*H*-pyrrole (**3a**)

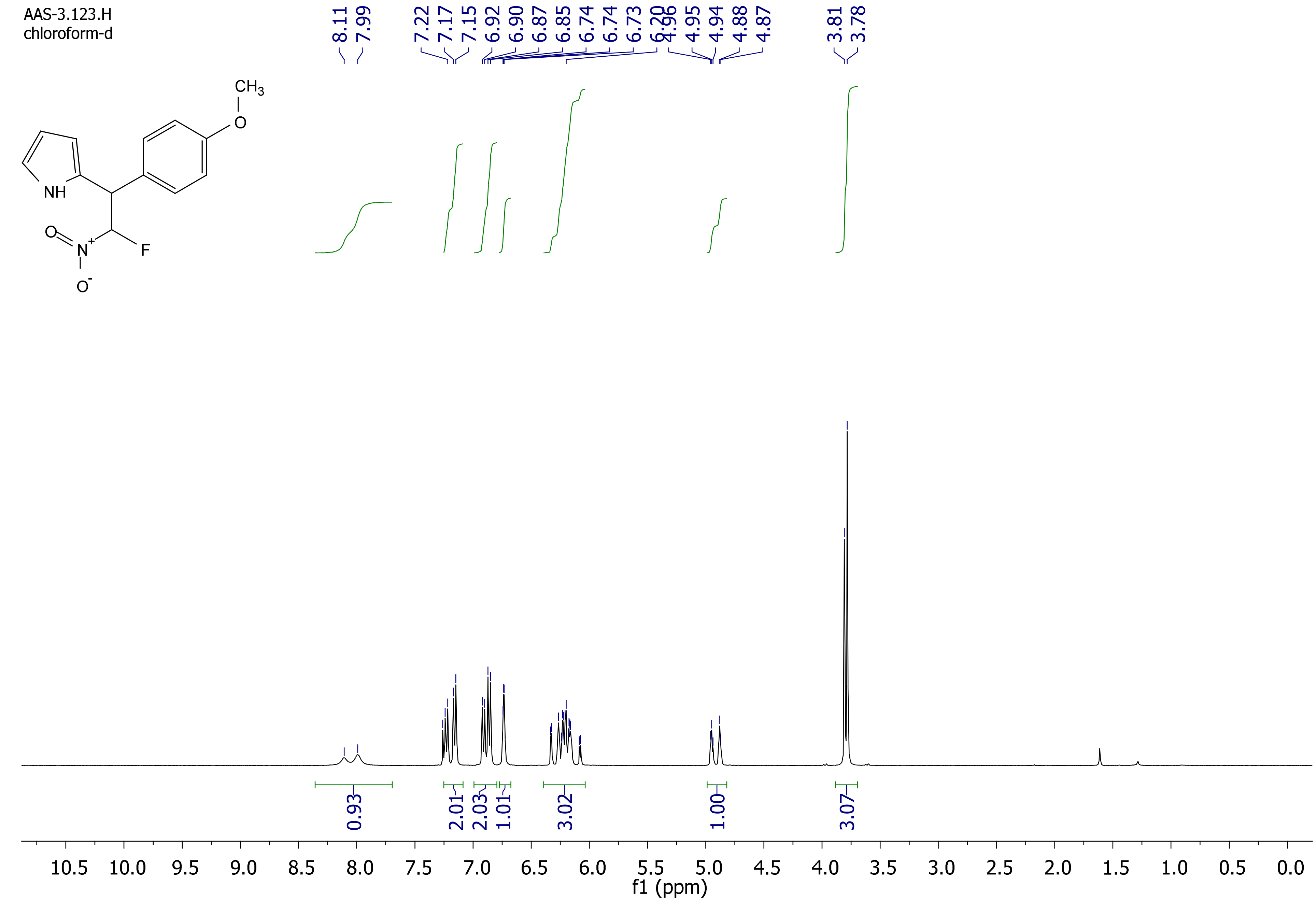

^{1}H NMR spectrum of 2-(2-fluoro-1-(4-methoxyphenyl)-2-nitroethyl)-1*H*-pyrrole (**3b**)

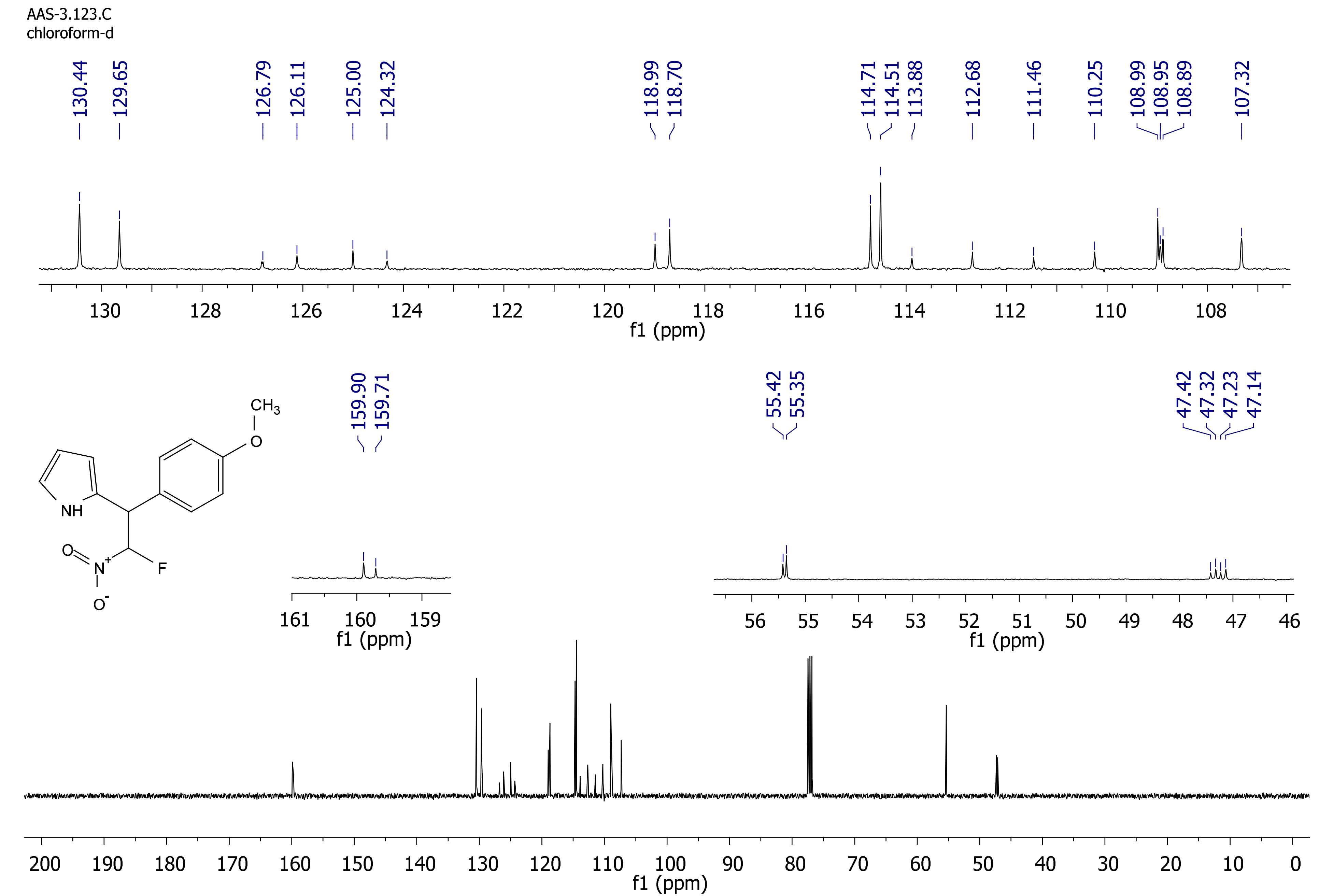

^{13}C NMR spectrum of 2-(2-fluoro-1-(4-methoxyphenyl)-2-nitroethyl)-1*H*-pyrrole (**3b**)

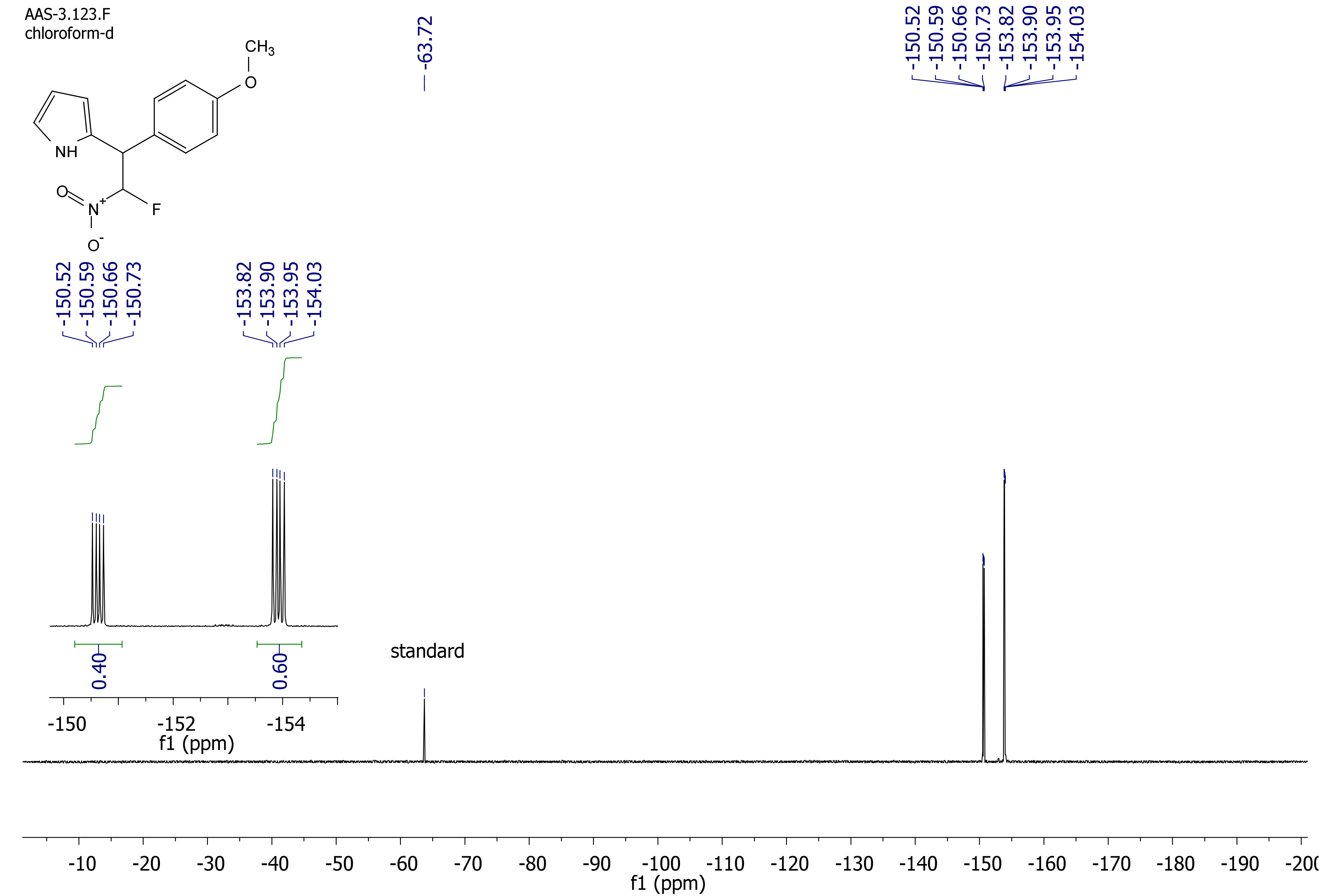

^{19}F NMR spectrum of 2-(2-fluoro-1-(4-methoxyphenyl)-2-nitroethyl)-1*H*-pyrrole (**3b**)

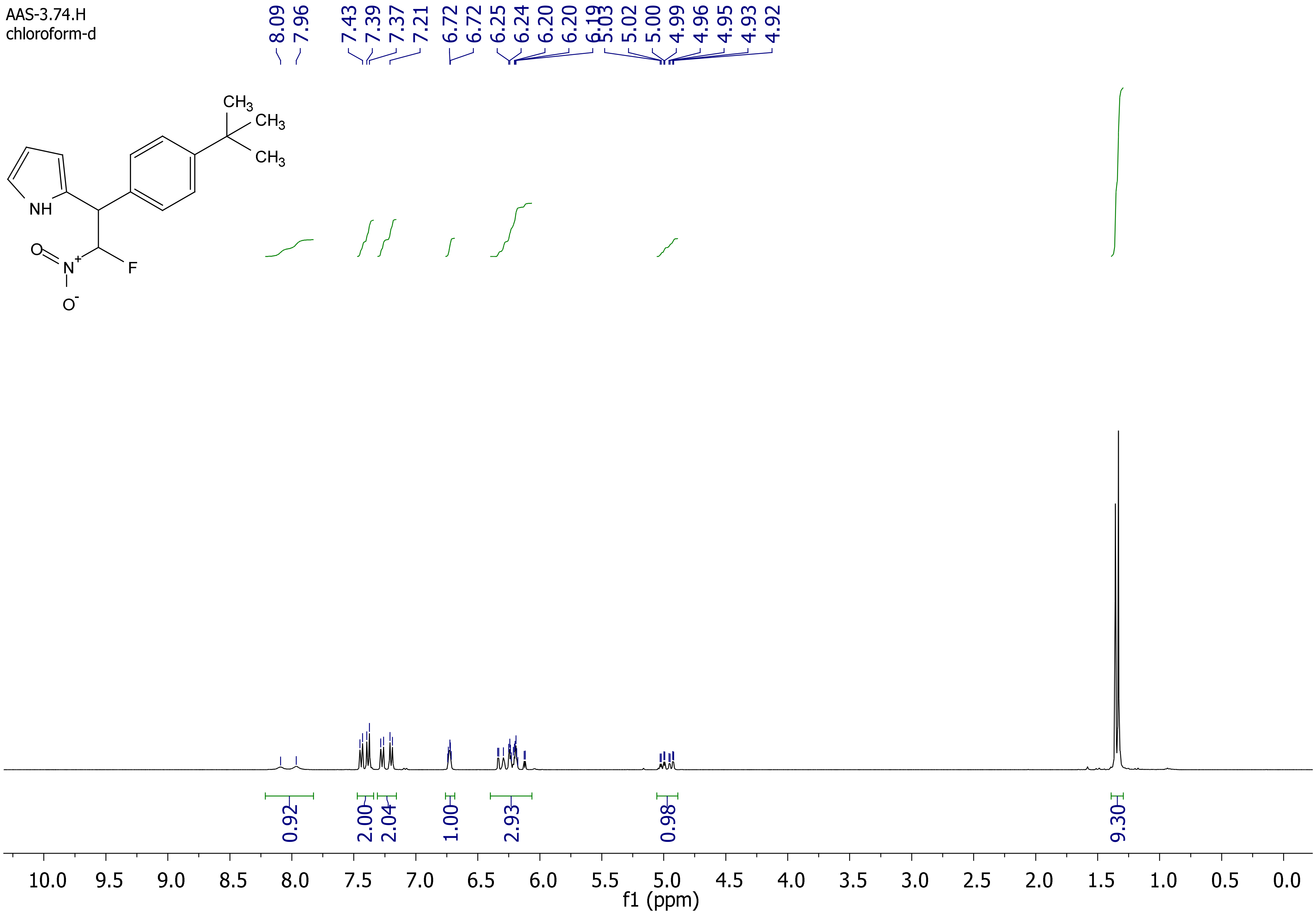

^{1}H NMR spectrum of 2-(1-(4-(*tert*-butyl)phenyl)-2-fluoro-2-nitroethyl)-1*H*-pyrrole (**3c**)

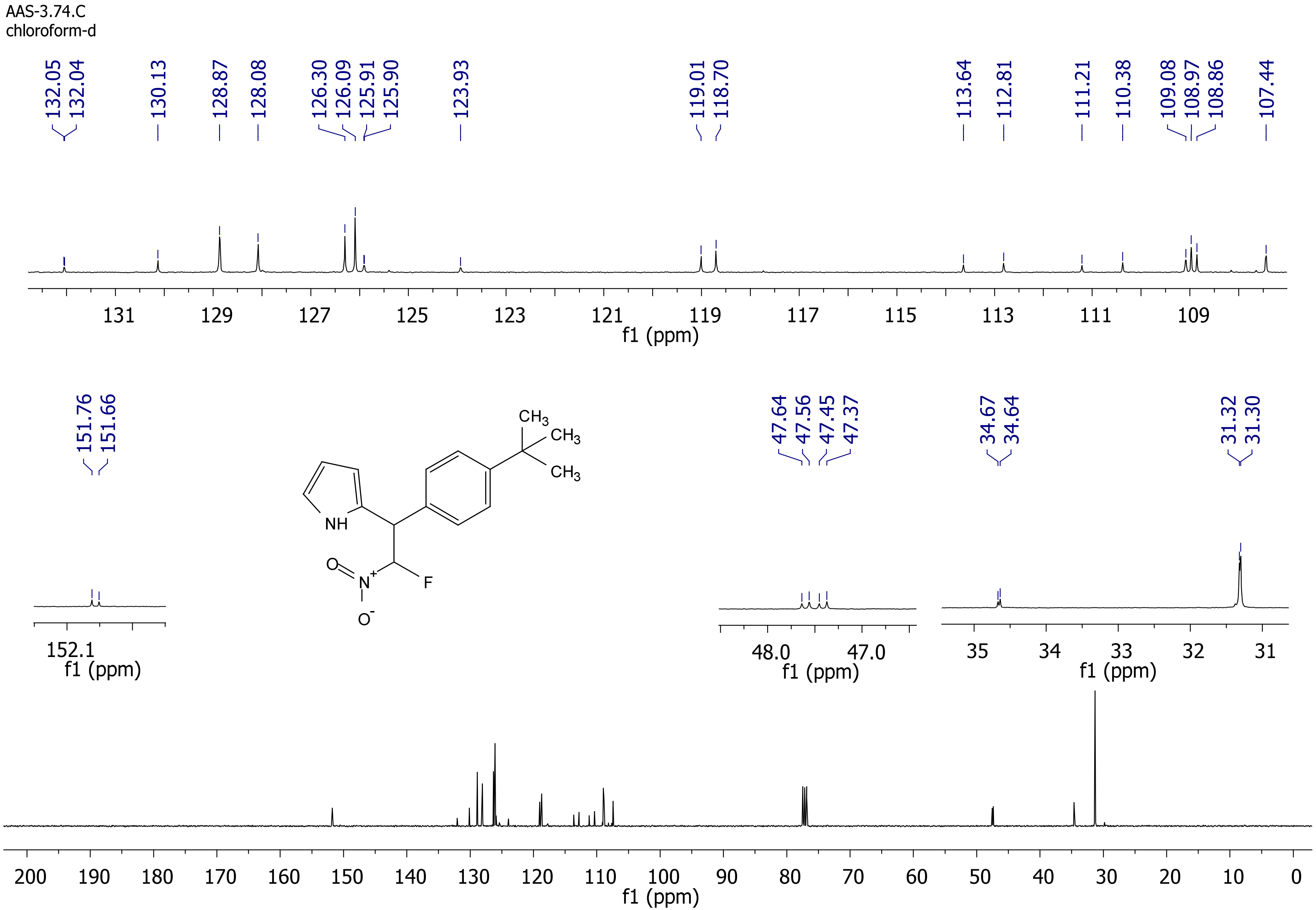

^{13}C NMR spectrum of 2-(1-(4-(*tert*-butyl)phenyl)-2-fluoro-2-nitroethyl)-1*H*-pyrrole (**3c**)

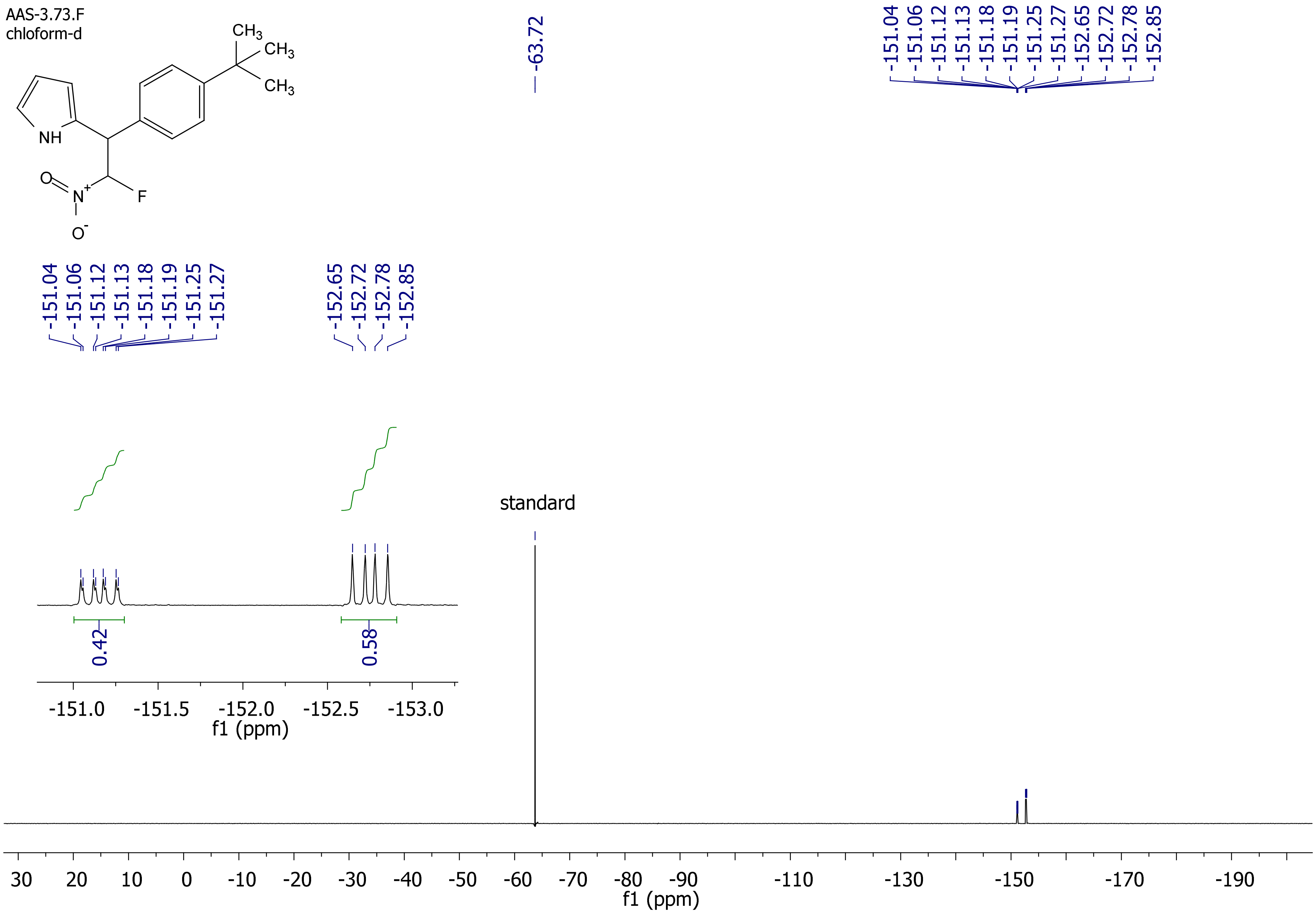

^{19}F NMR spectrum of 2-(1-(4-(*tert*-butyl)phenyl)-2-fluoro-2-nitroethyl)-1*H*-pyrrole (**3c**)

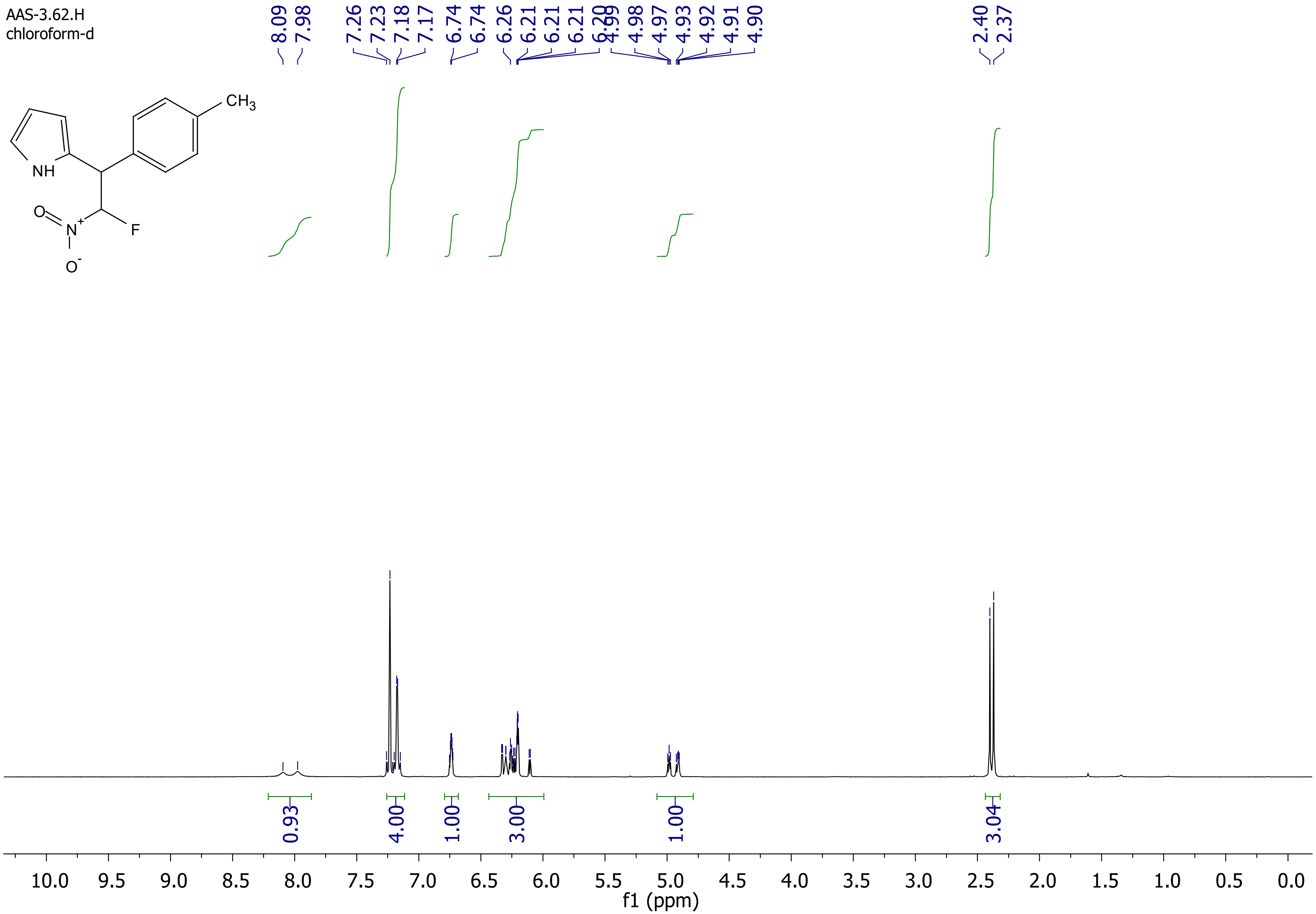

^{1}H NMR spectrum of 2-(2-fluoro-2-nitro-1-(p-tolyl)ethyl)-1*H*-pyrrole (**3d**)

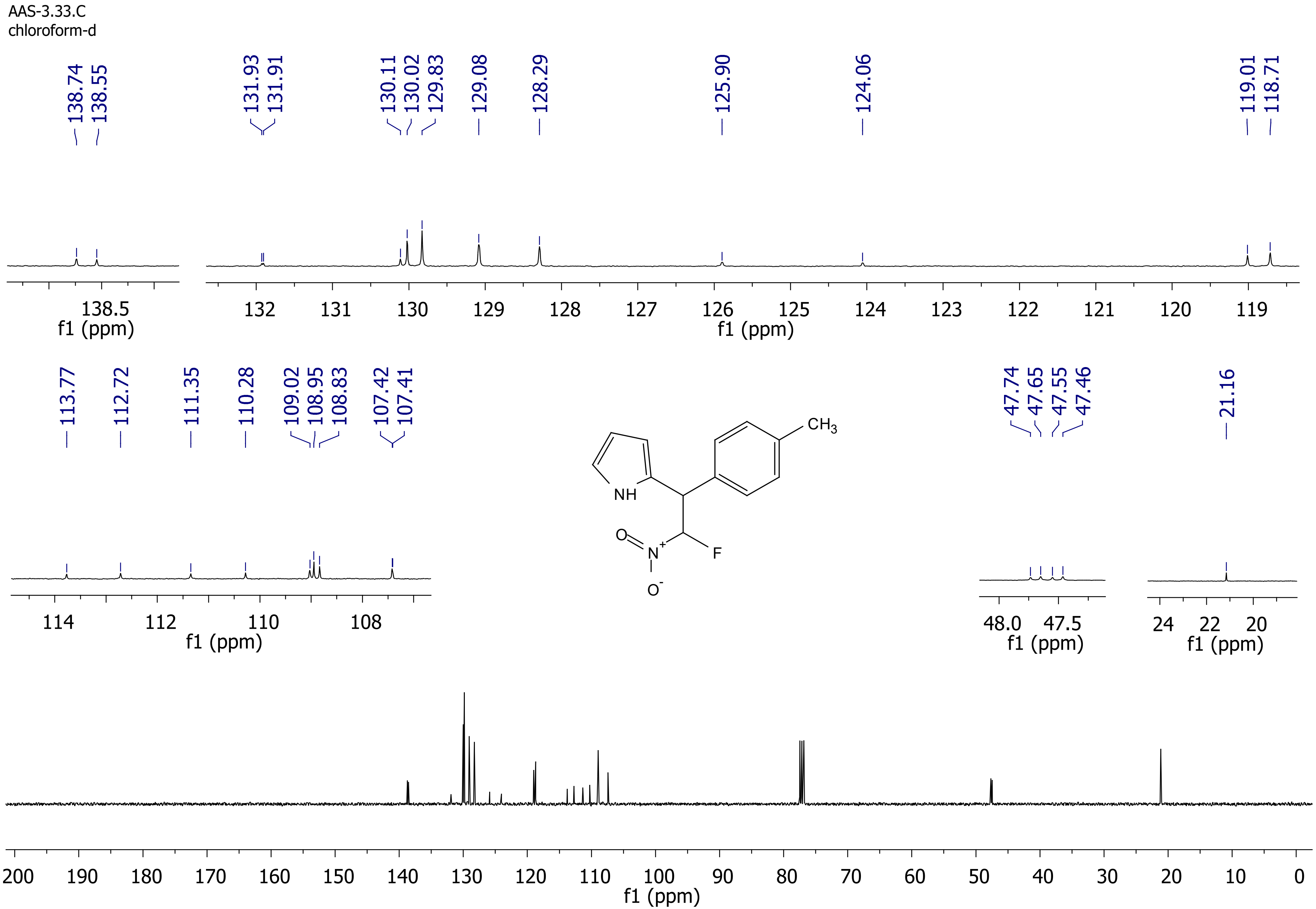

^{13}C NMR spectrum of 2-(2-fluoro-2-nitro-1-(p-tolyl)ethyl)-1*H*-pyrrole (**3d**)

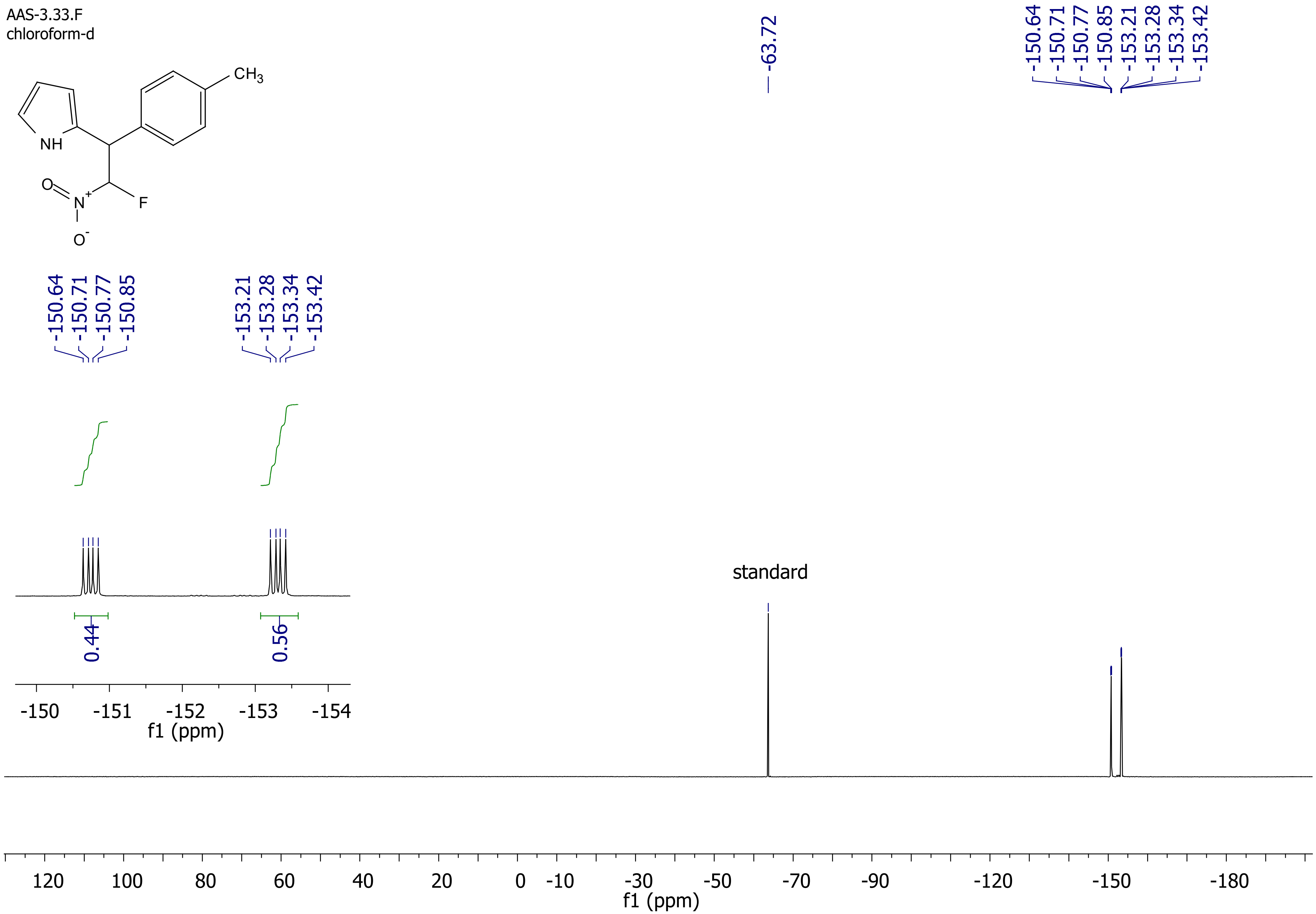

^{19}F NMR spectrum of 2-(2-fluoro-2-nitro-1-(p-tolyl)ethyl)-1*H*-pyrrole (**3d**)

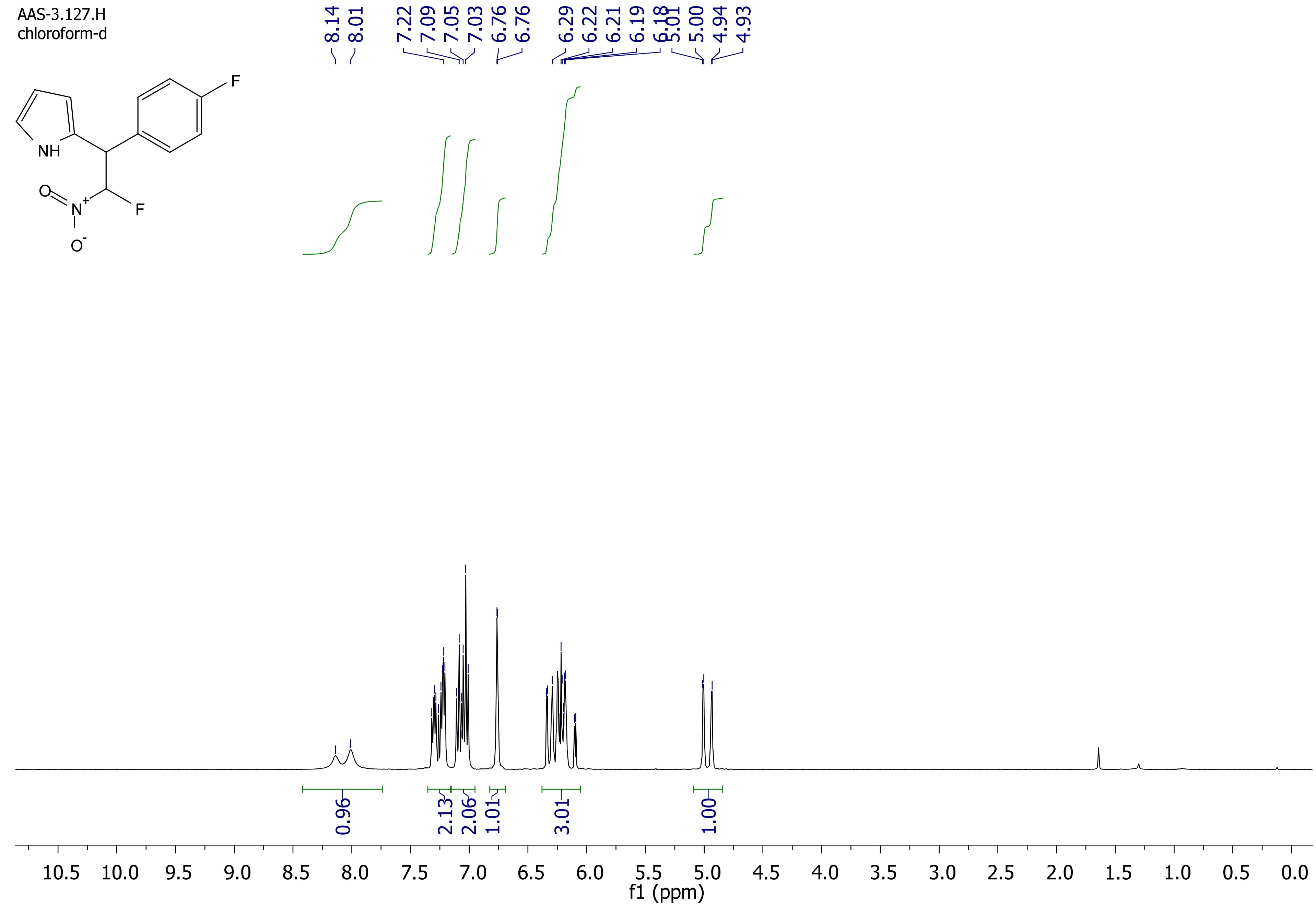

¹H NMR spectrum of 2-(2-fluoro-1-(4-fluorophenyl)-2-nitroethyl)-1*H*-pyrrole (**3e**)

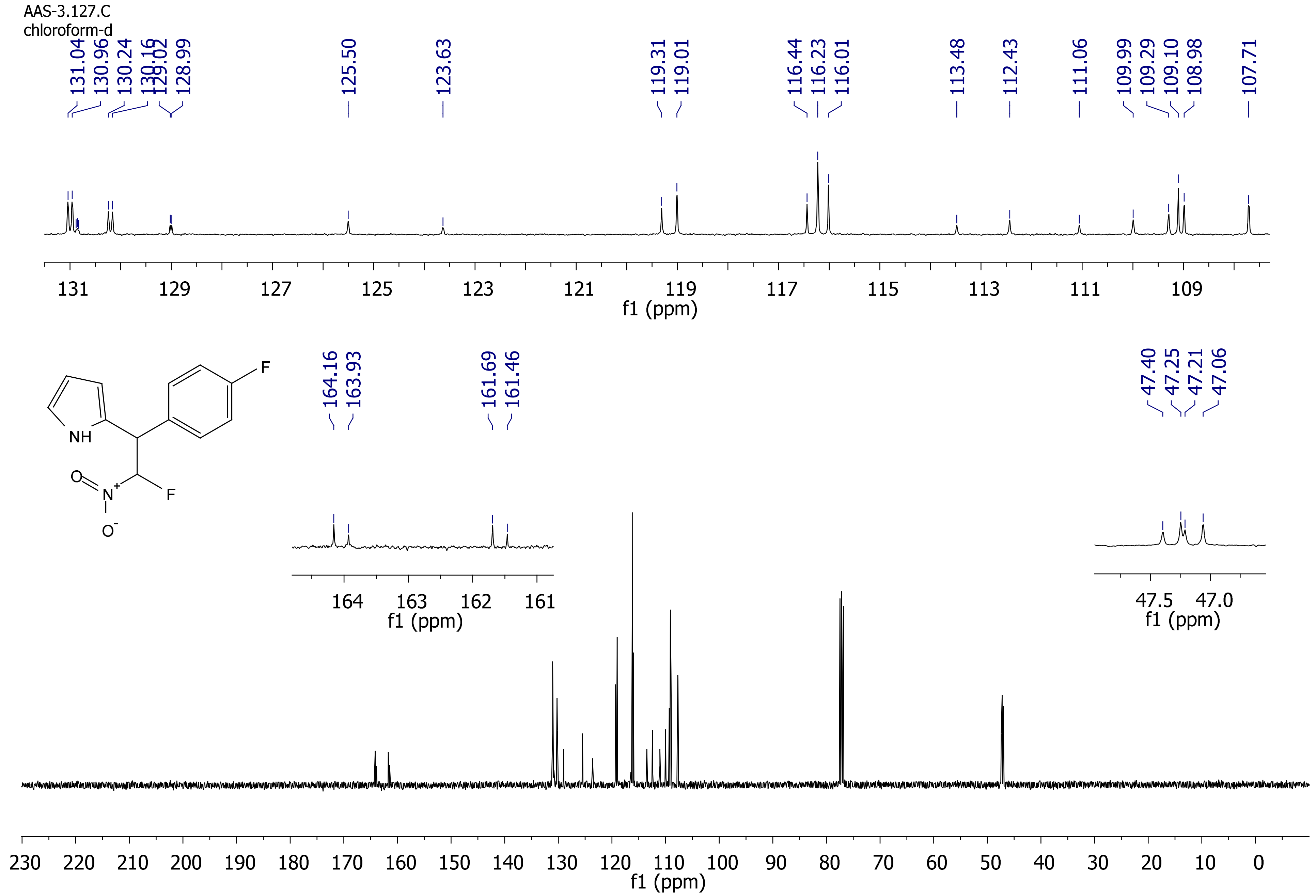

^{13}C NMR spectrum of 2-(2-fluoro-1-(4-fluorophenyl)-2-nitroethyl)-1*H*-pyrrole (**3e**)

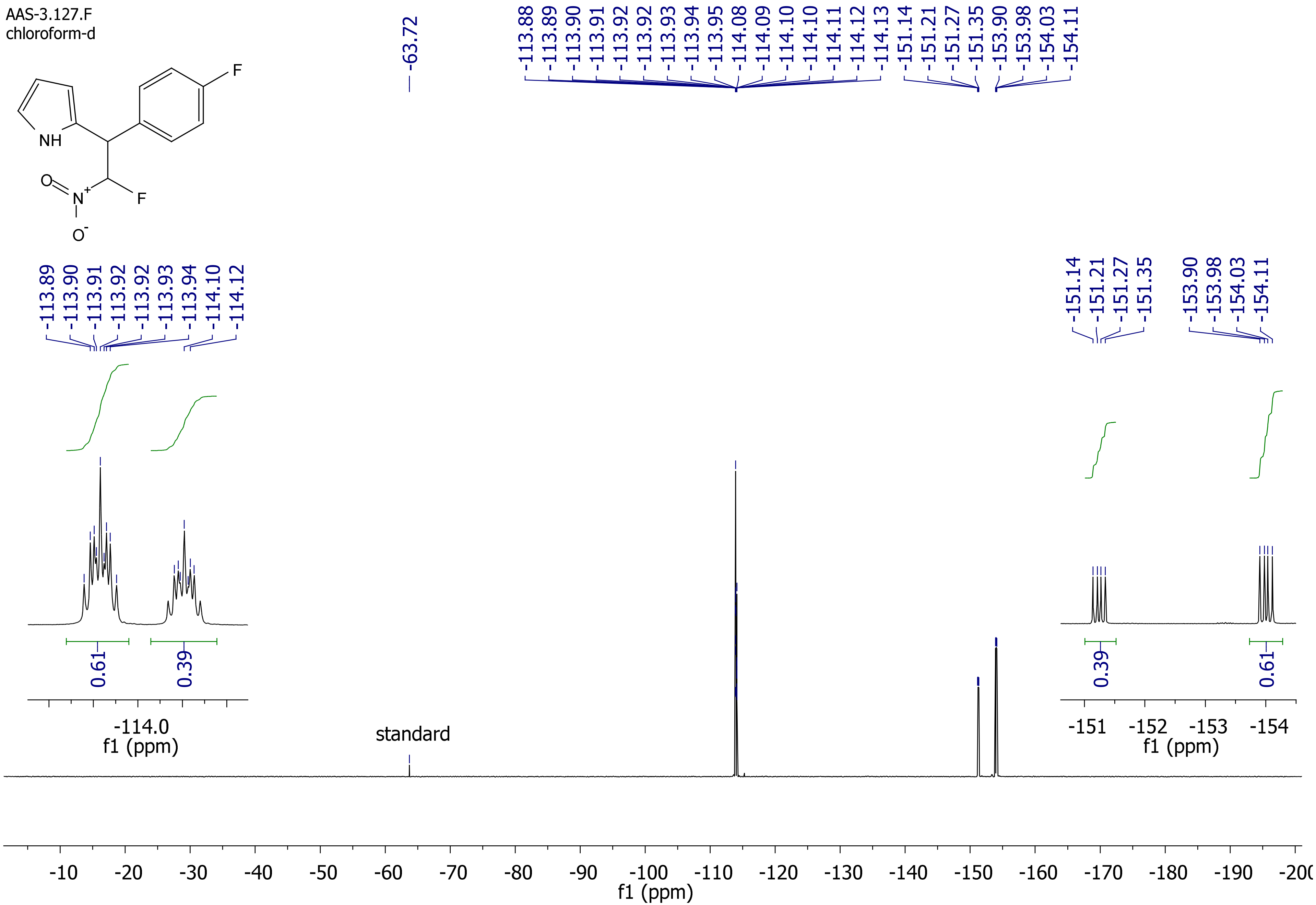

^{19}F NMR spectrum of 2-(2-fluoro-1-(4-fluorophenyl)-2-nitroethyl)-1*H*-pyrrole (**3e**)

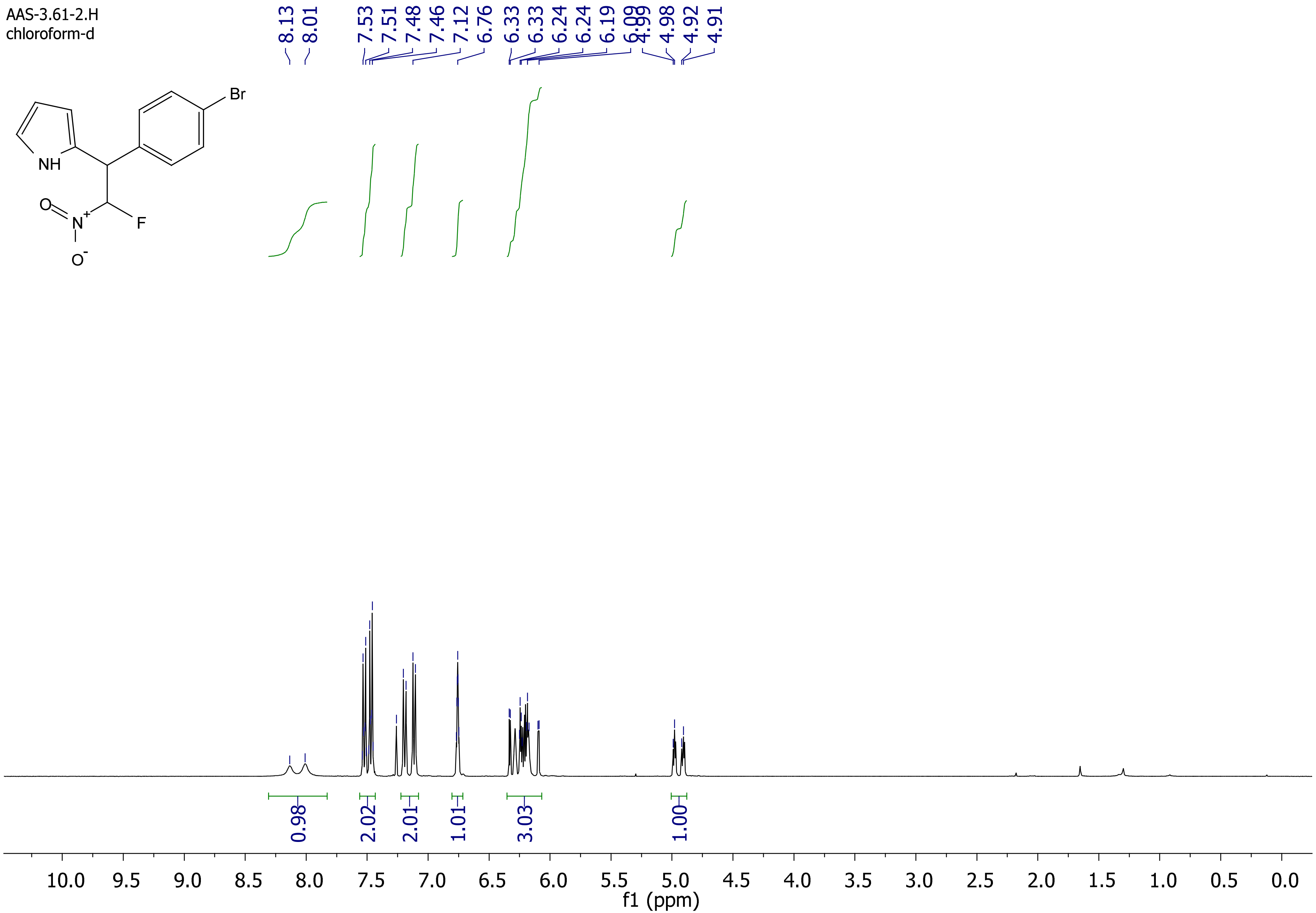

^{1}H NMR spectrum of 2-(1-(4-bromophenyl)-2-fluoro-2-nitroethyl)-1*H*-pyrrole (**3f**)

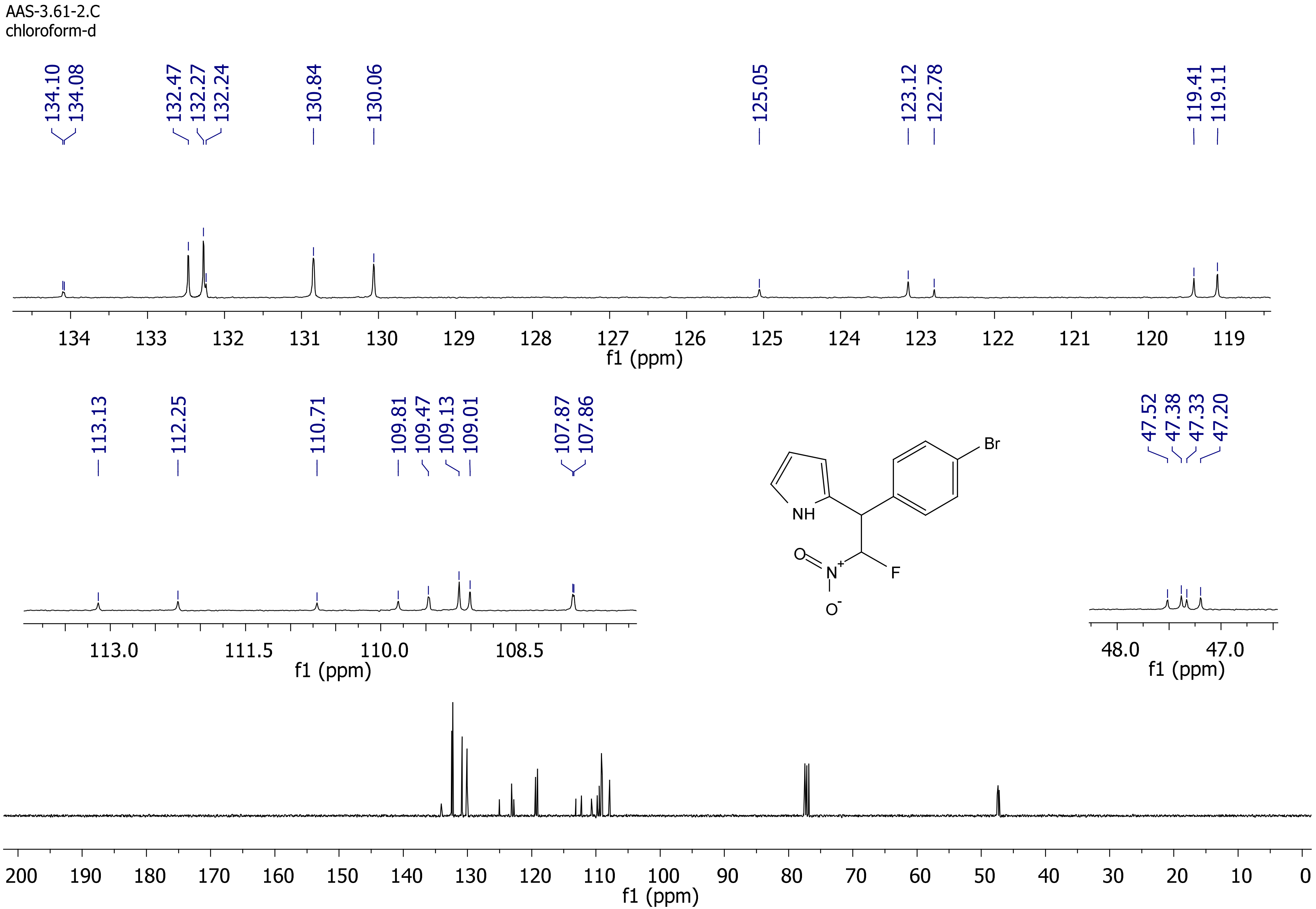

^{13}C NMR spectrum of 2-(1-(4-bromophenyl)-2-fluoro-2-nitroethyl)-1*H*-pyrrole (**3f**)

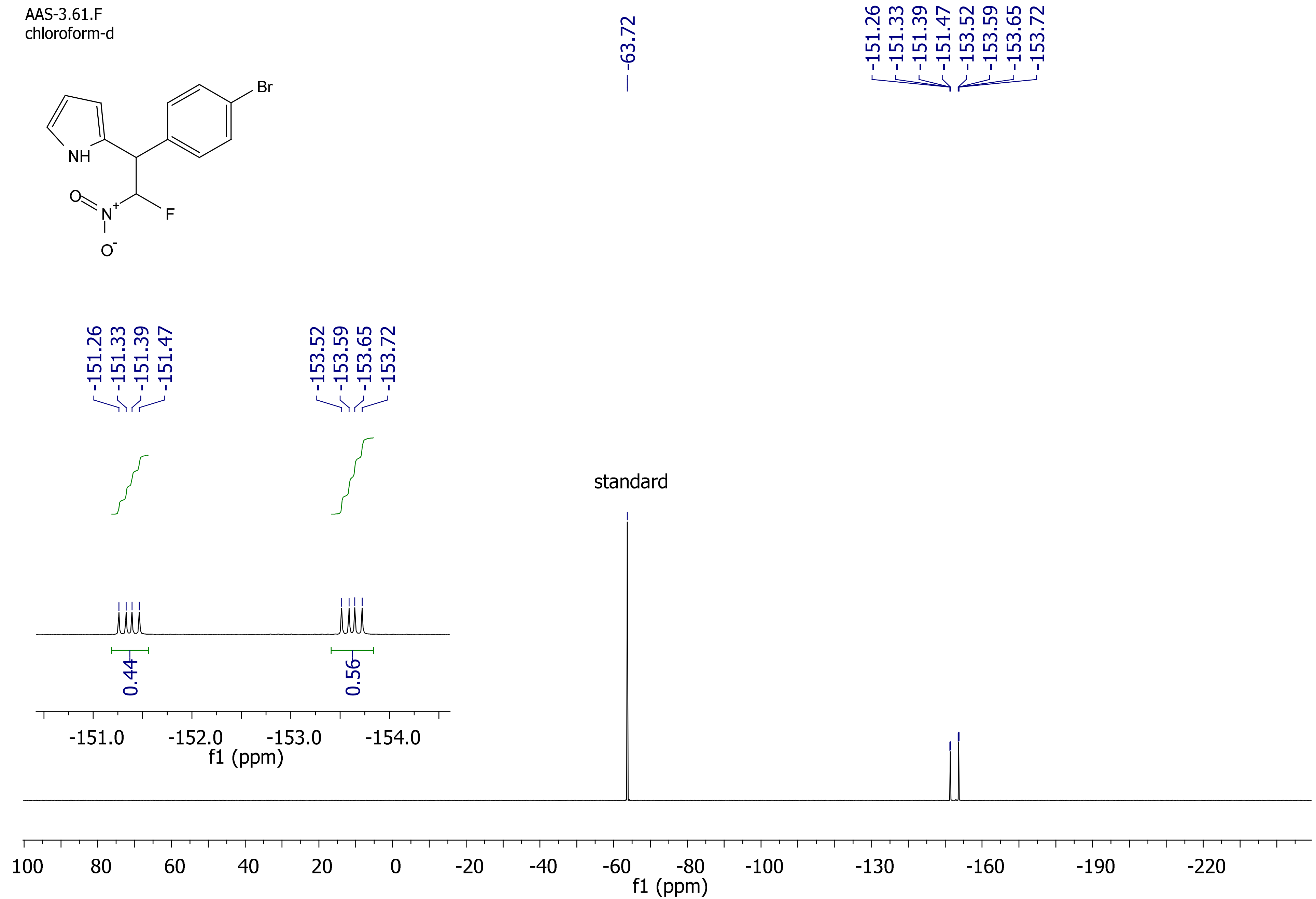

^{19}F NMR spectrum of 2-(1-(4-bromophenyl)-2-fluoro-2-nitroethyl)-1*H*-pyrrole (**3f**)

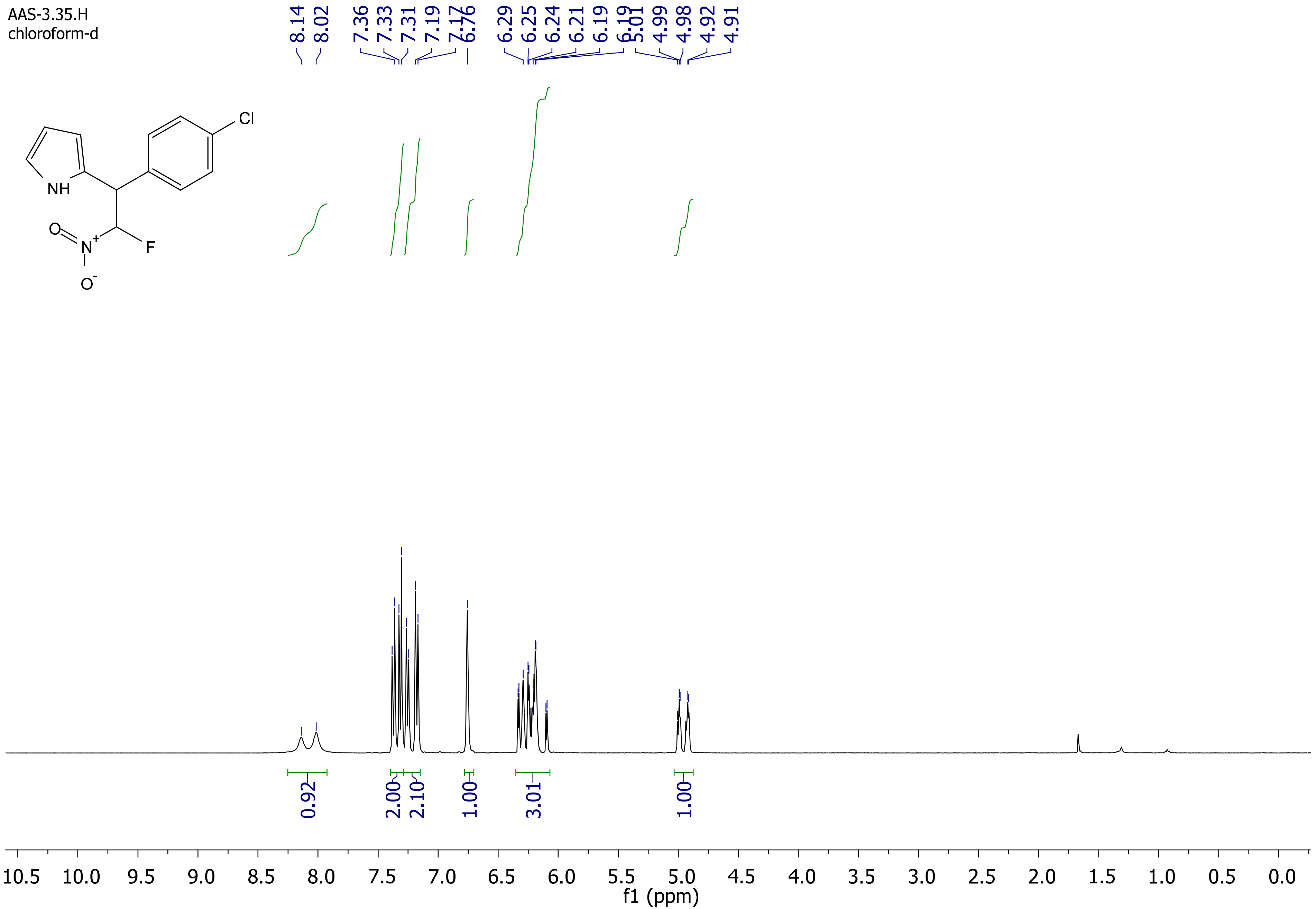

^{1}H NMR spectrum of 2-(1-(4-chlorophenyl)-2-fluoro-2-nitroethyl)-1*H*-pyrrole (**3g**)

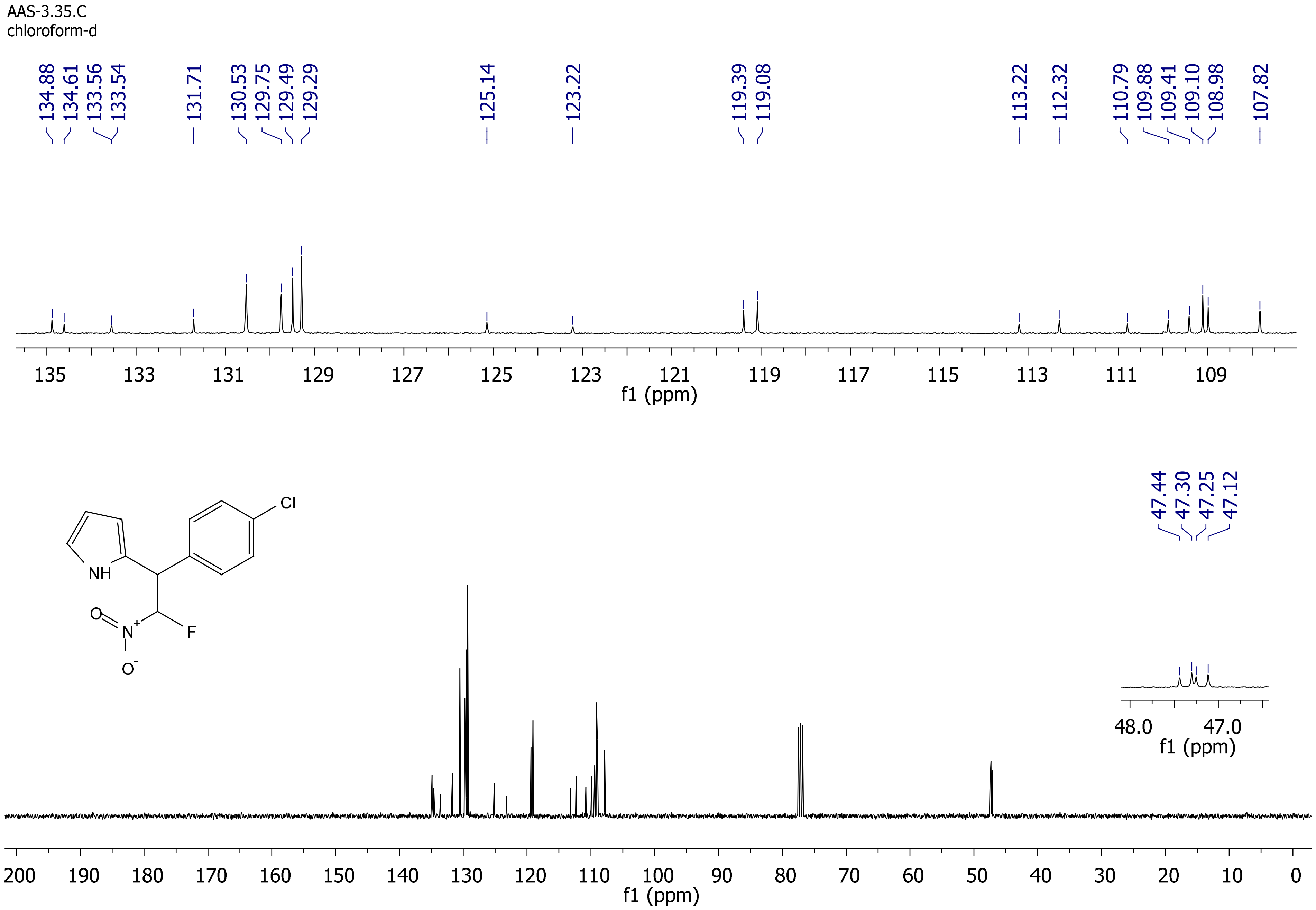

^{13}C NMR spectrum of 2-(1-(4-chlorophenyl)-2-fluoro-2-nitroethyl)-1*H*-pyrrole (**3g**)

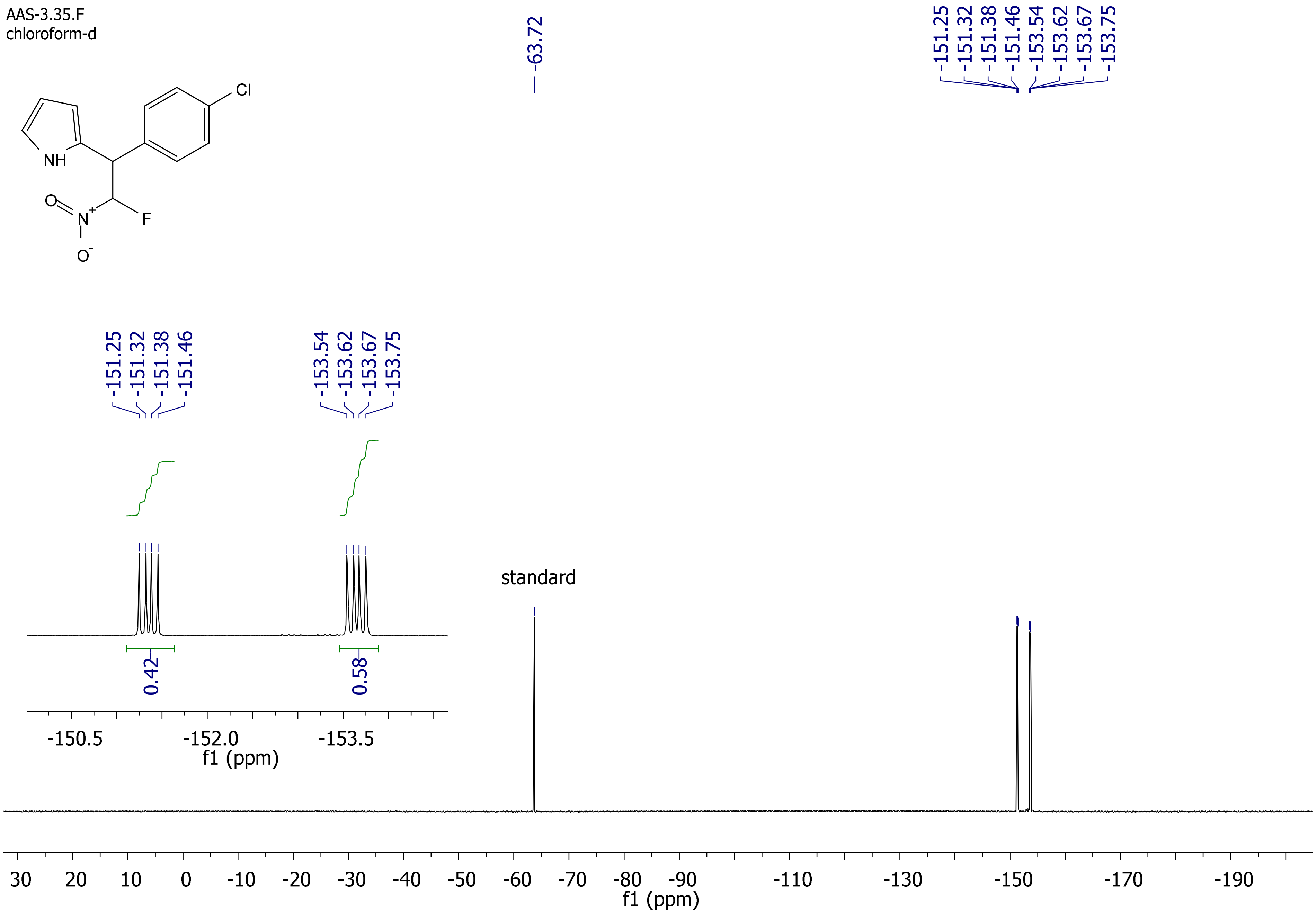

^{19}F NMR spectrum of 2-(1-(4-chlorophenyl)-2-fluoro-2-nitroethyl)-1*H*-pyrrole (**3g**)

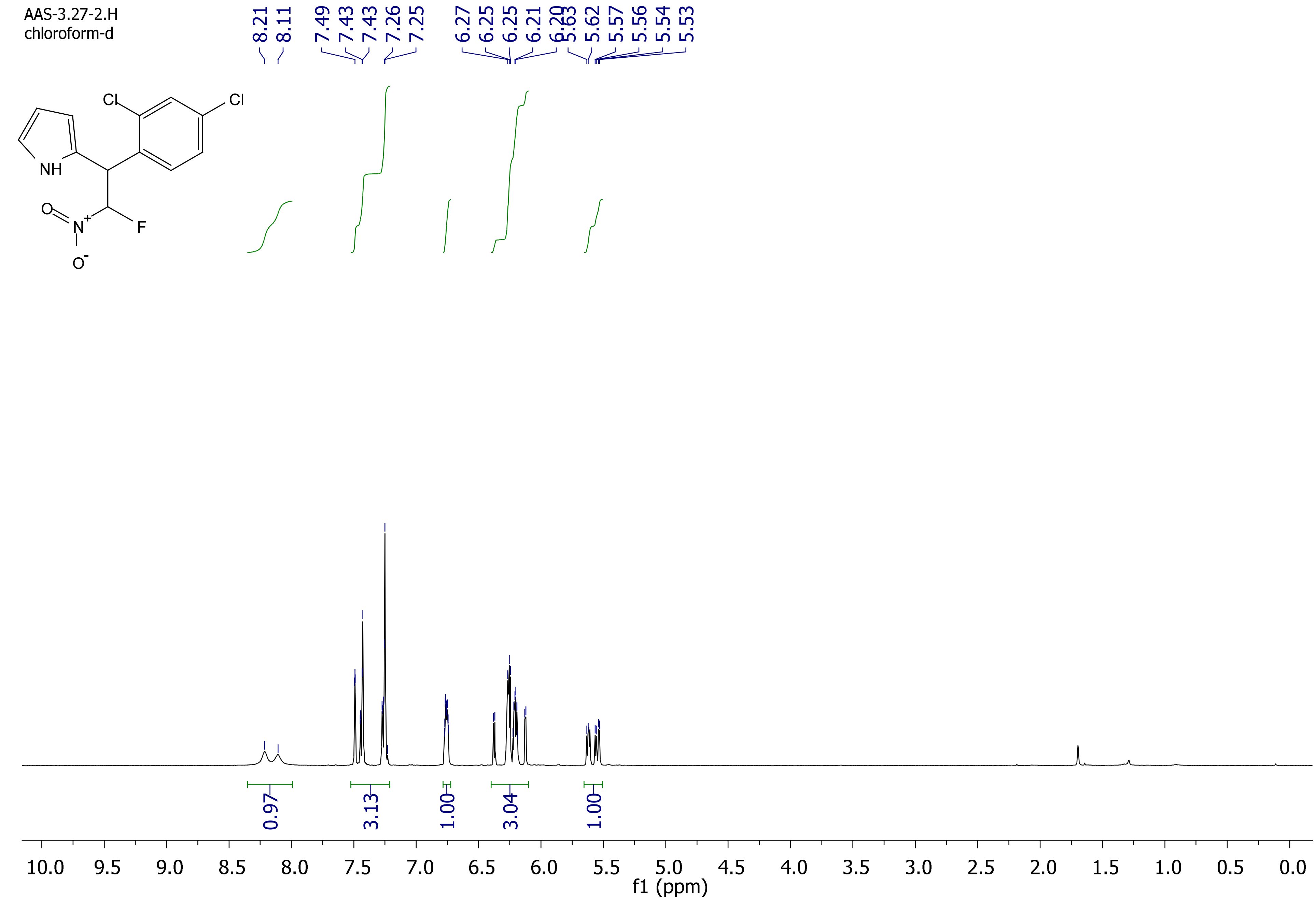

^{1}H NMR spectrum of 2-(1-(2,4-dichlorophenyl)-2-fluoro-2-nitroethyl)-1*H*-pyrrole (**3h**)

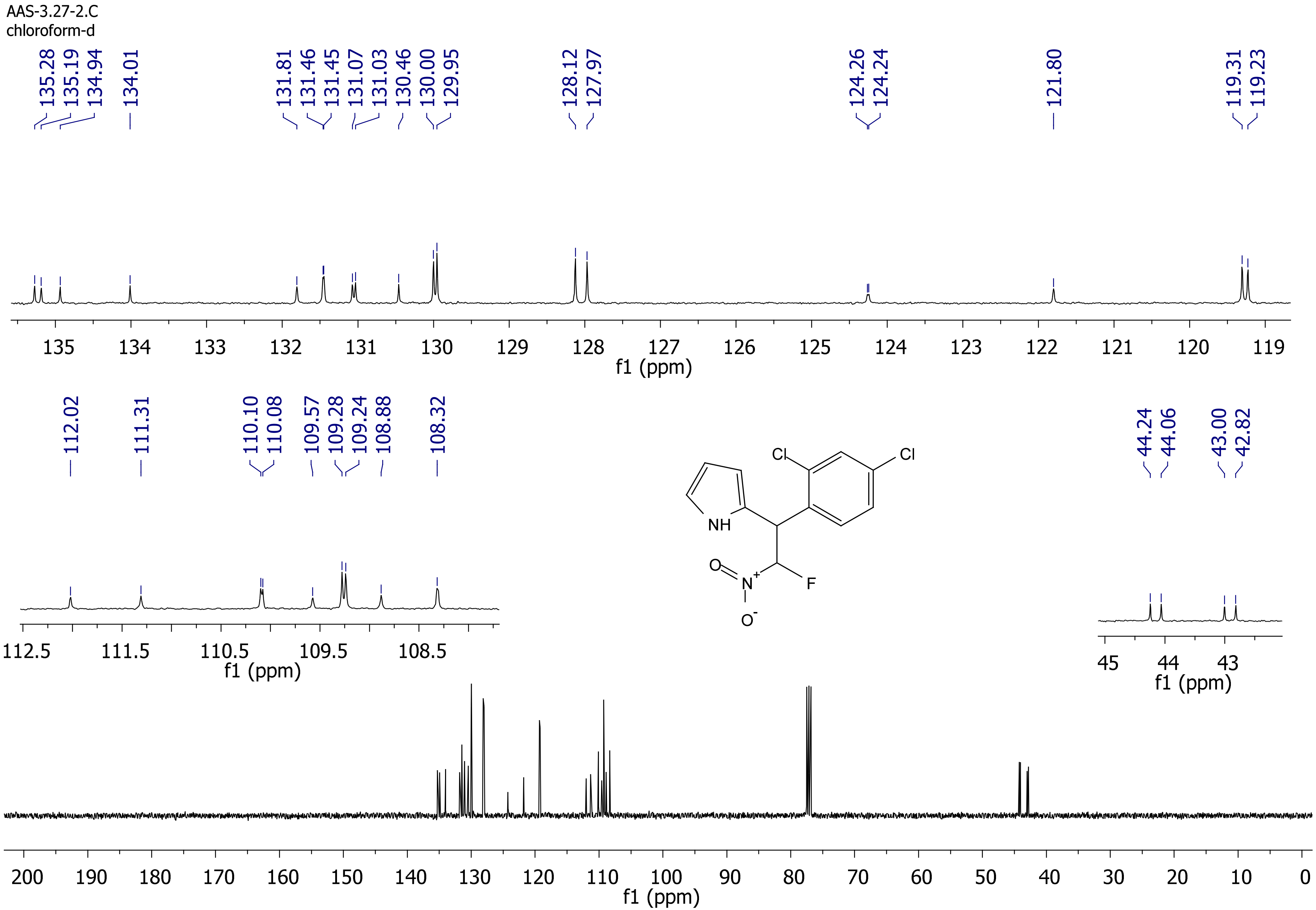

^{13}C NMR spectrum of 2-(1-(2,4-dichlorophenyl)-2-fluoro-2-nitroethyl)-1*H*-pyrrole (**3h**)

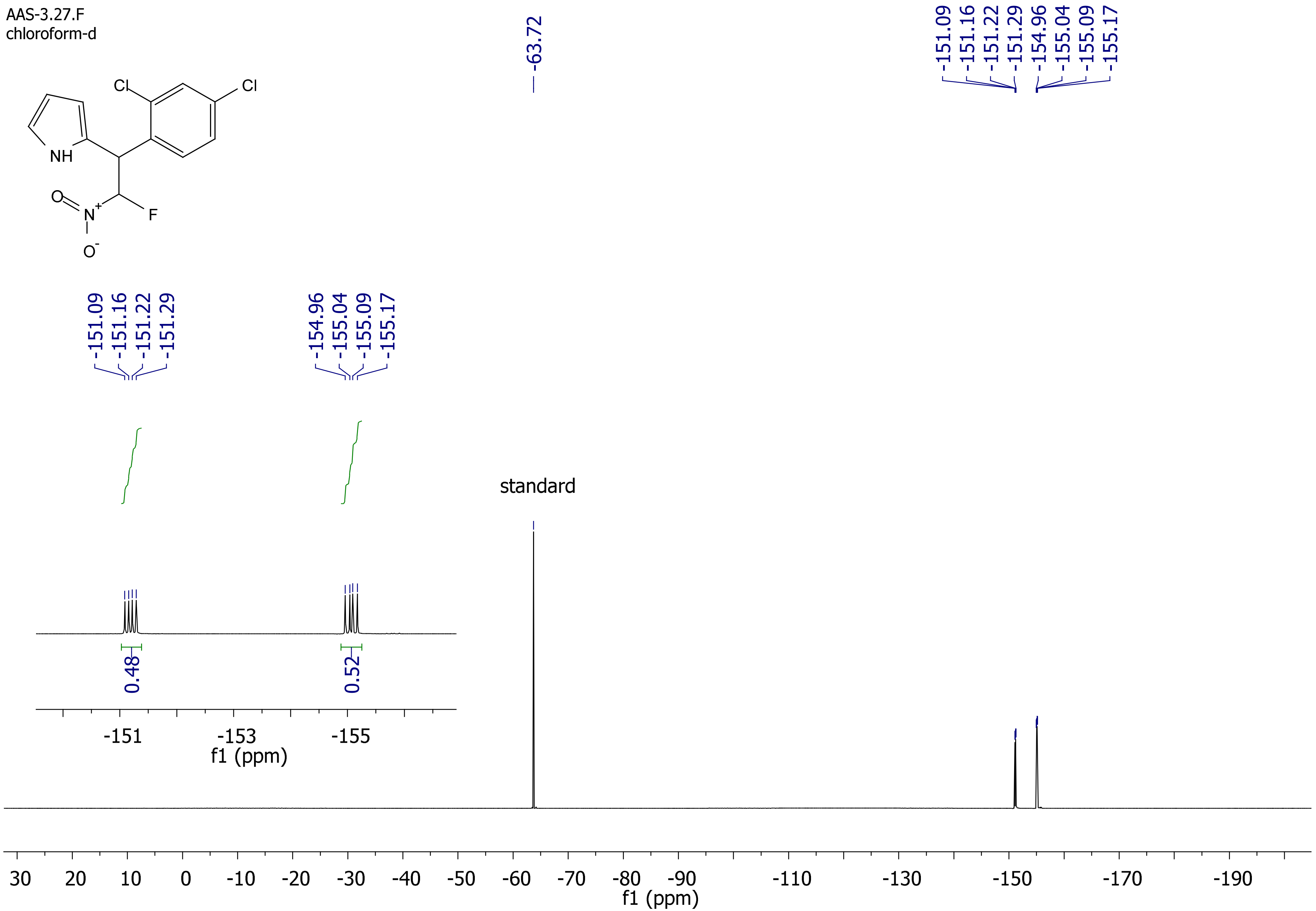

^{19}F NMR spectrum of 2-(1-(2,4-dichlorophenyl)-2-fluoro-2-nitroethyl)-1*H*-pyrrole (**3h**)

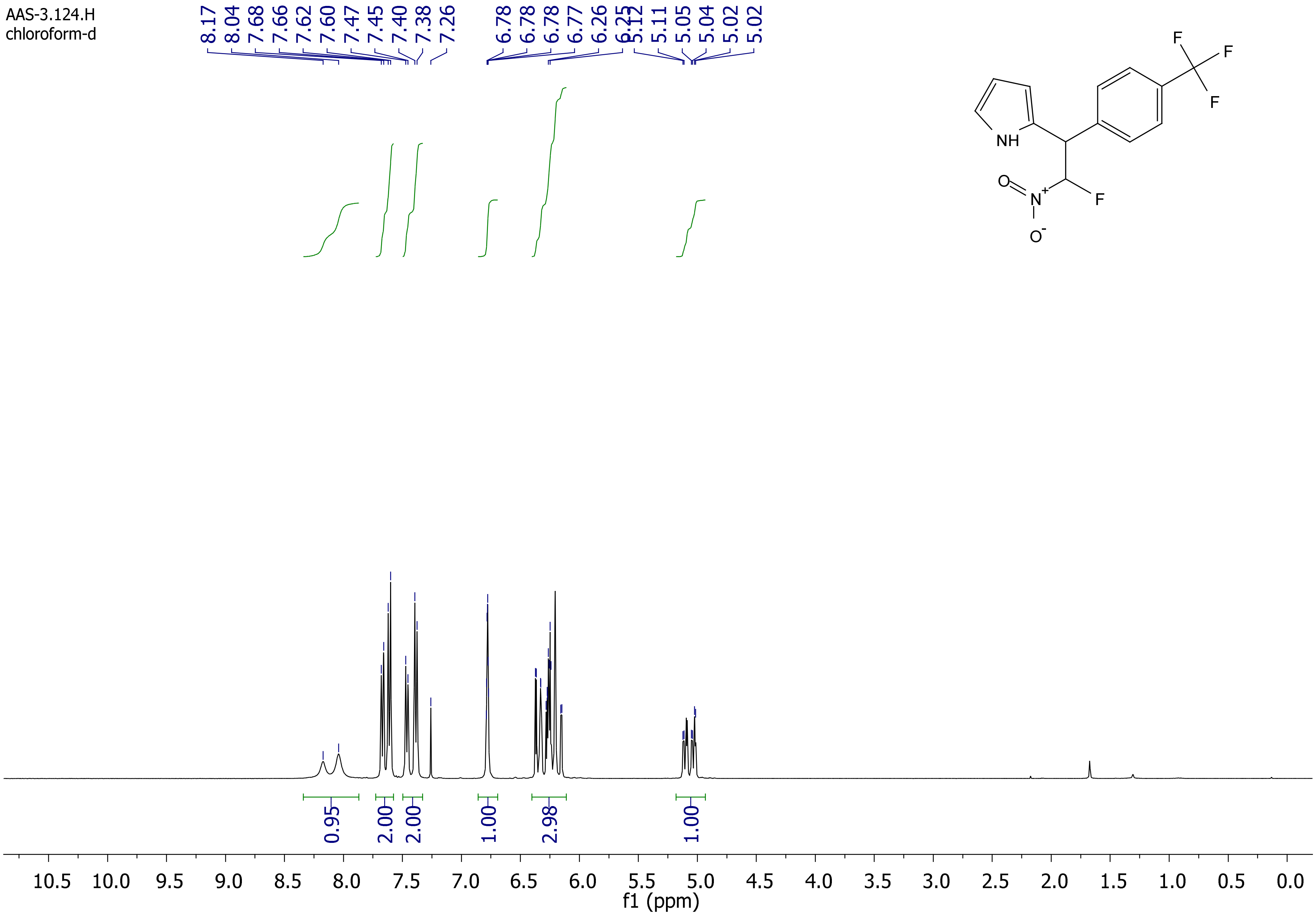

^{1}H NMR spectrum of 2-(2-fluoro-2-nitro-1-(4-(trifluoromethyl)phenyl)ethyl)-1*H*-pyrrole (**3i**)

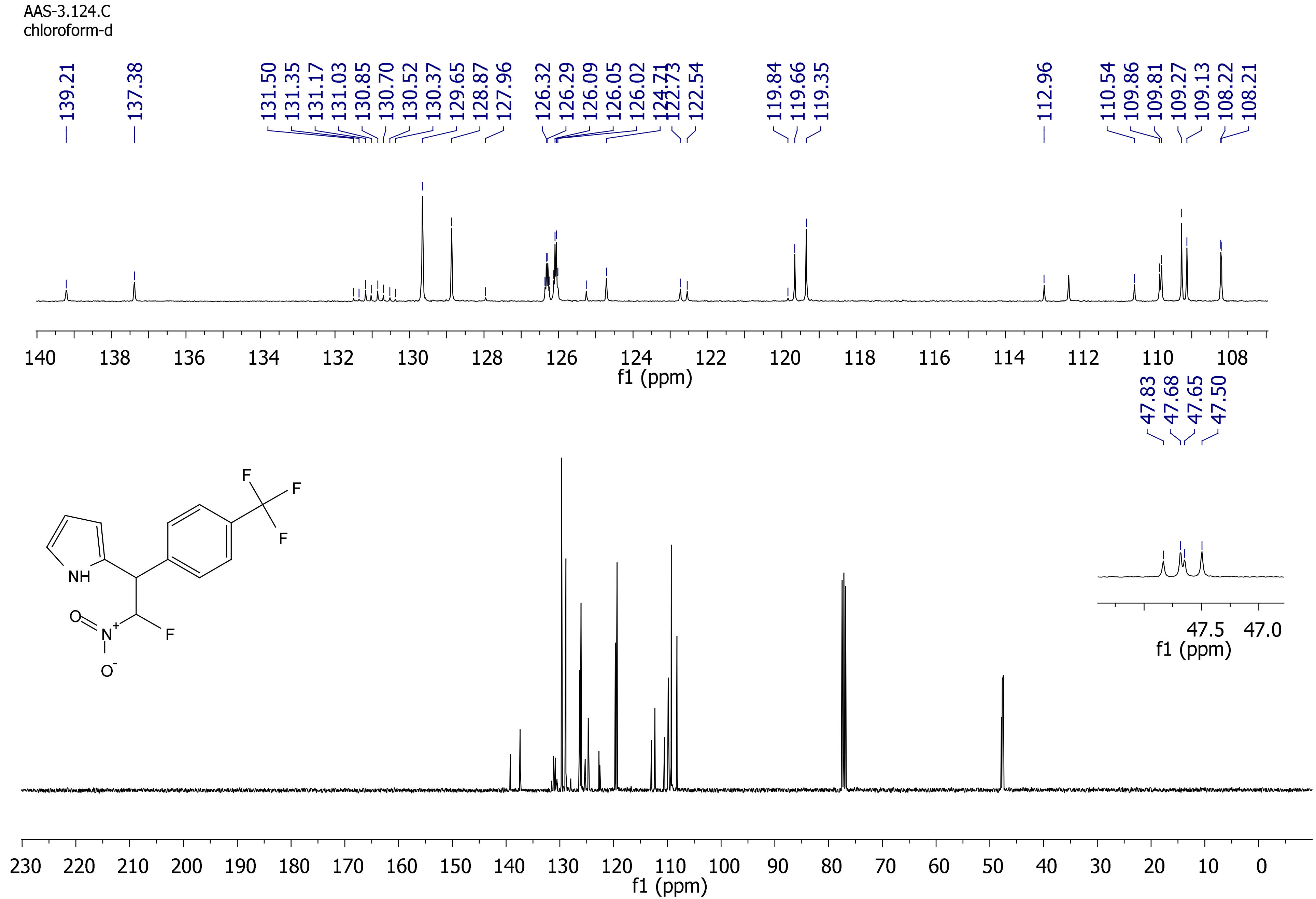

^{13}C NMR spectrum of 2-(2-fluoro-2-nitro-1-(4-(trifluoromethyl)phenyl)ethyl)-1*H*-pyrrole (**3i**)

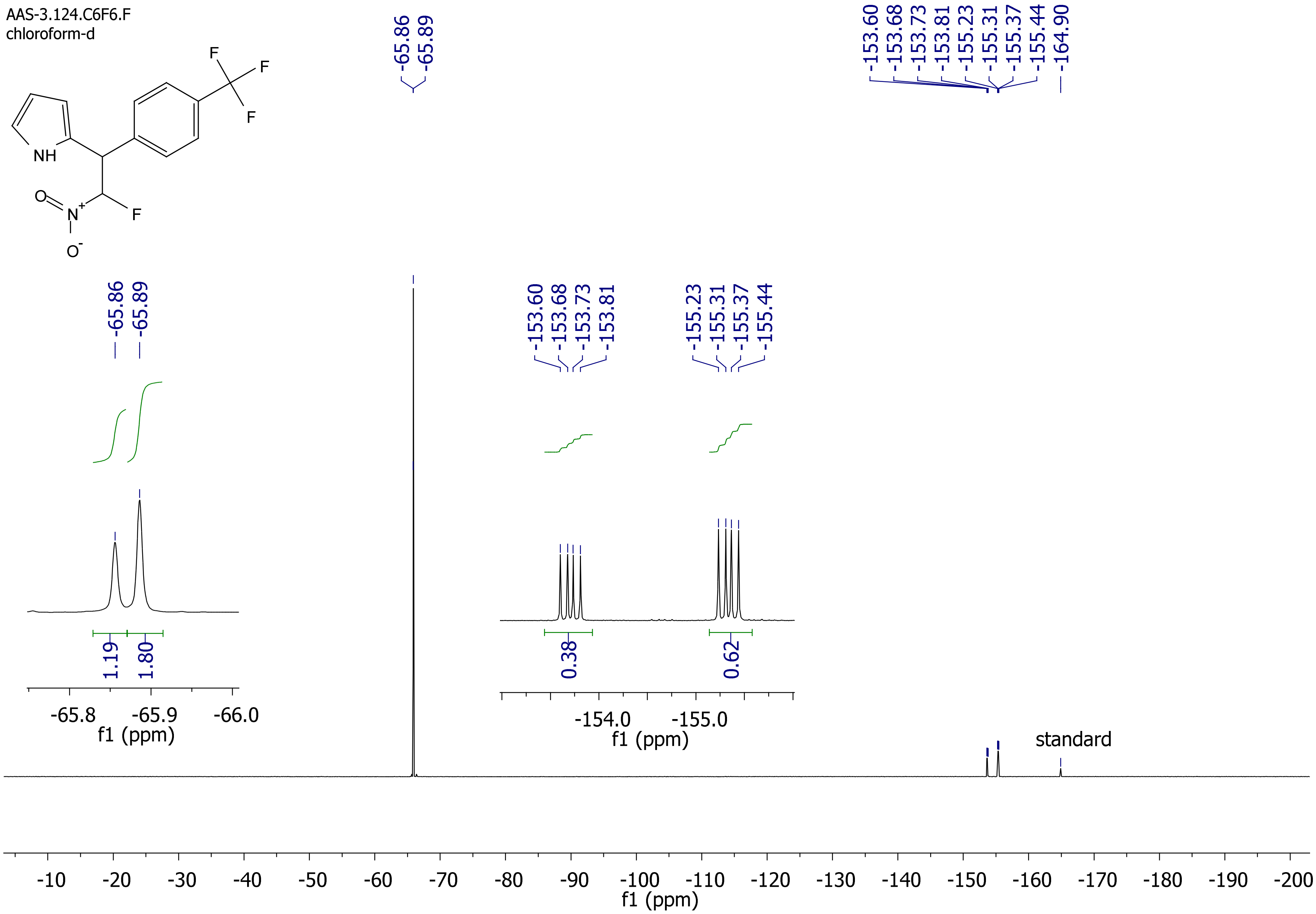

^{19}F NMR spectrum of 2-(2-fluoro-2-nitro-1-(4-(trifluoromethyl)phenyl)ethyl)-1*H*-pyrrole (**3i**)

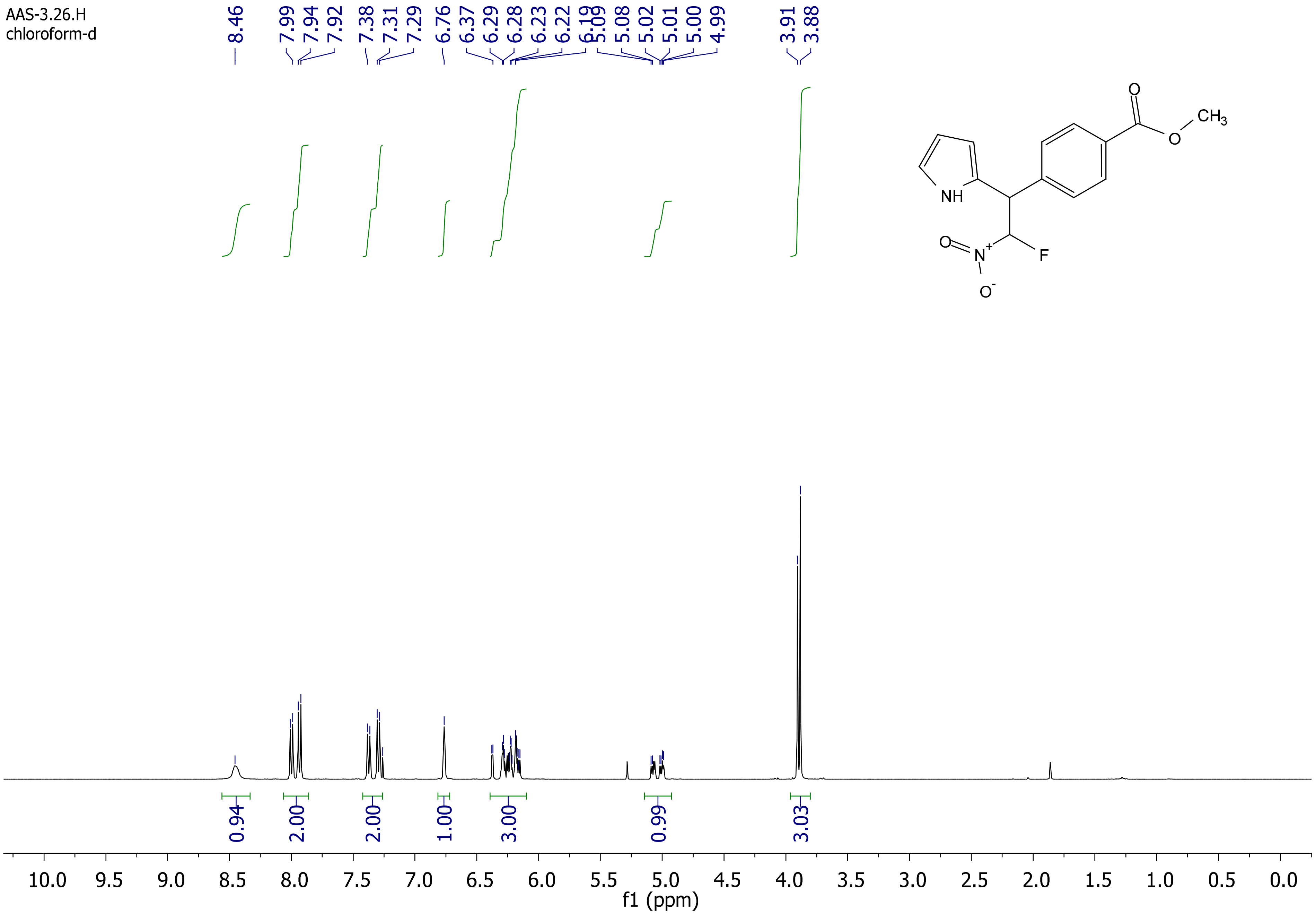

^{1}H NMR spectrum of methyl 4-(2-fluoro-2-nitro-1-(1*H*-pyrrol-2-yl)ethyl)benzoate (**3j**)

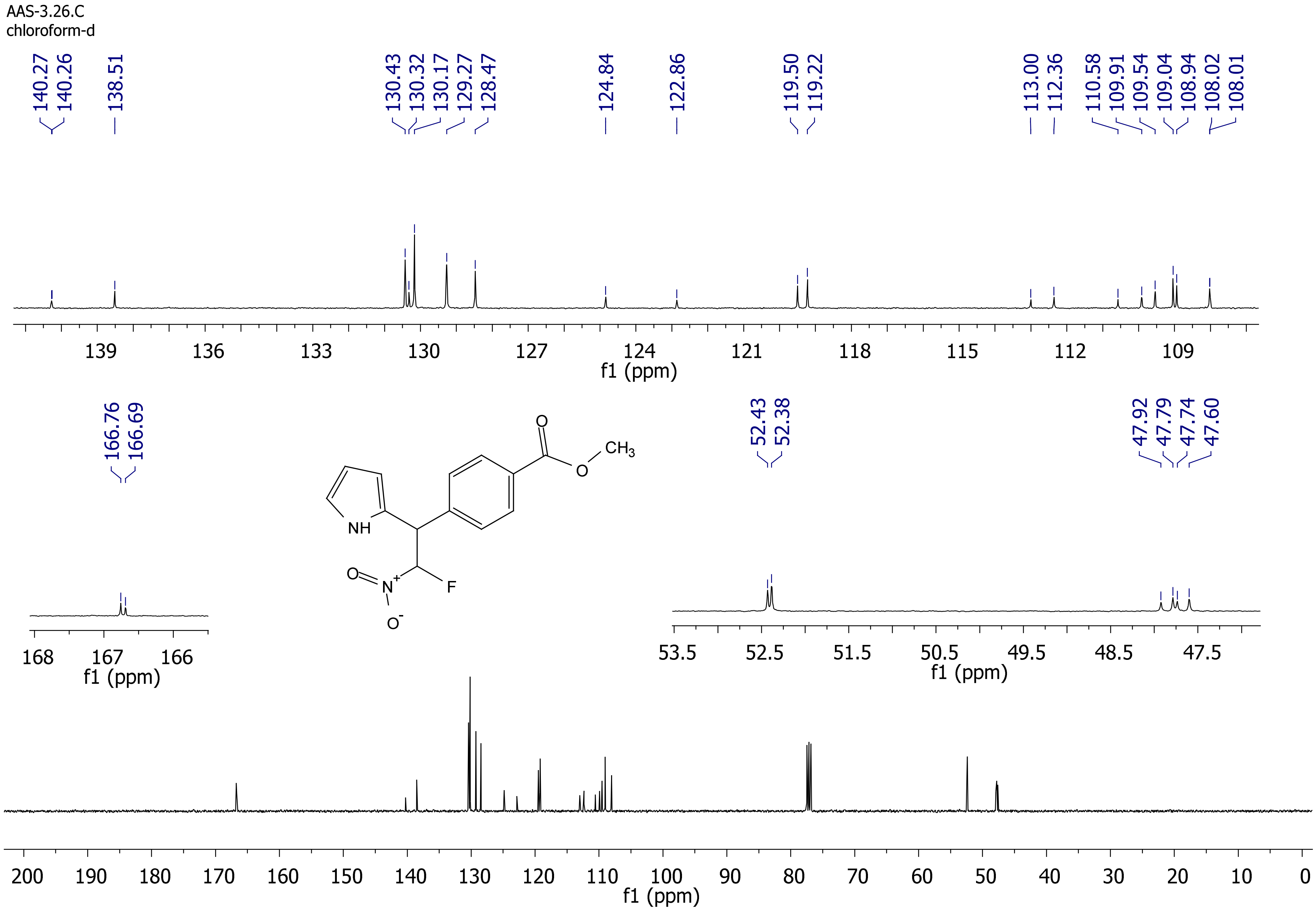

^{13}C NMR spectrum of methyl 4-(2-fluoro-2-nitro-1-(1*H*-pyrrol-2-yl)ethyl)benzoate (**3j**)

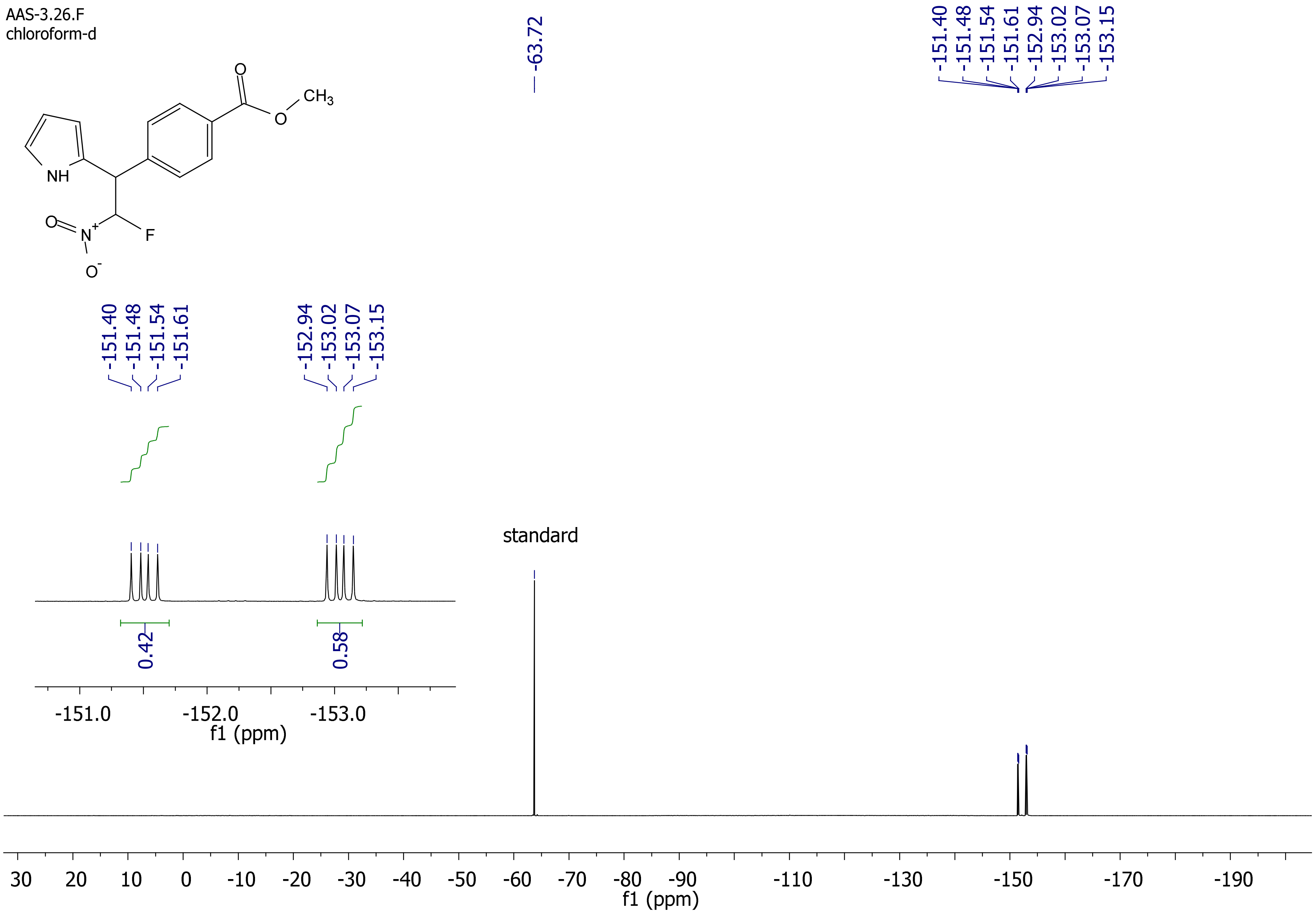

^{19}F NMR spectrum of methyl 4-(2-fluoro-2-nitro-1-(1*H*-pyrrol-2-yl)ethyl)benzoate (**3j**)

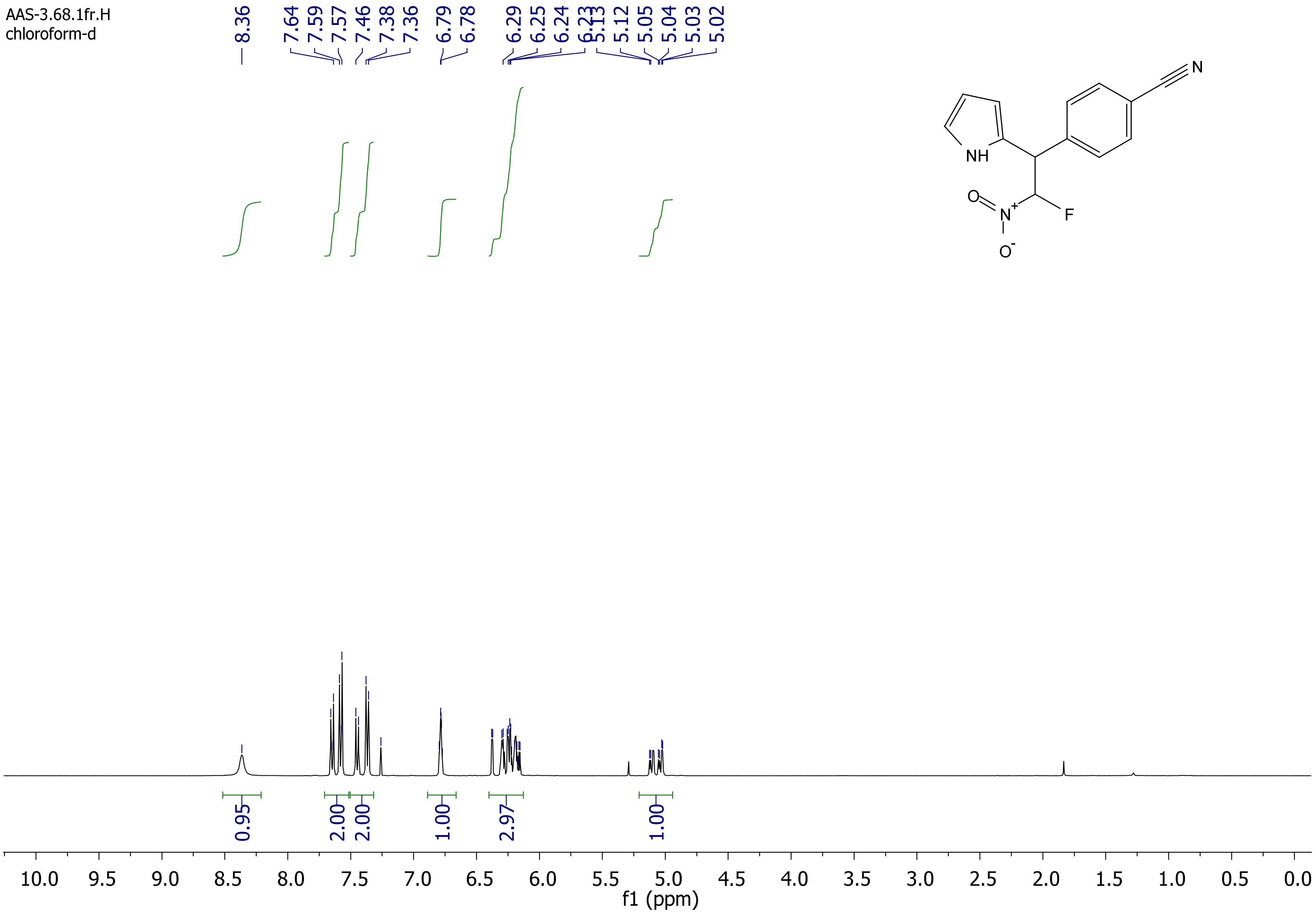

^{1}H NMR spectrum of 4-(2-fluoro-2-nitro-1-(1*H*-pyrrol-2-yl)ethyl)benzonitrile (**3k**)

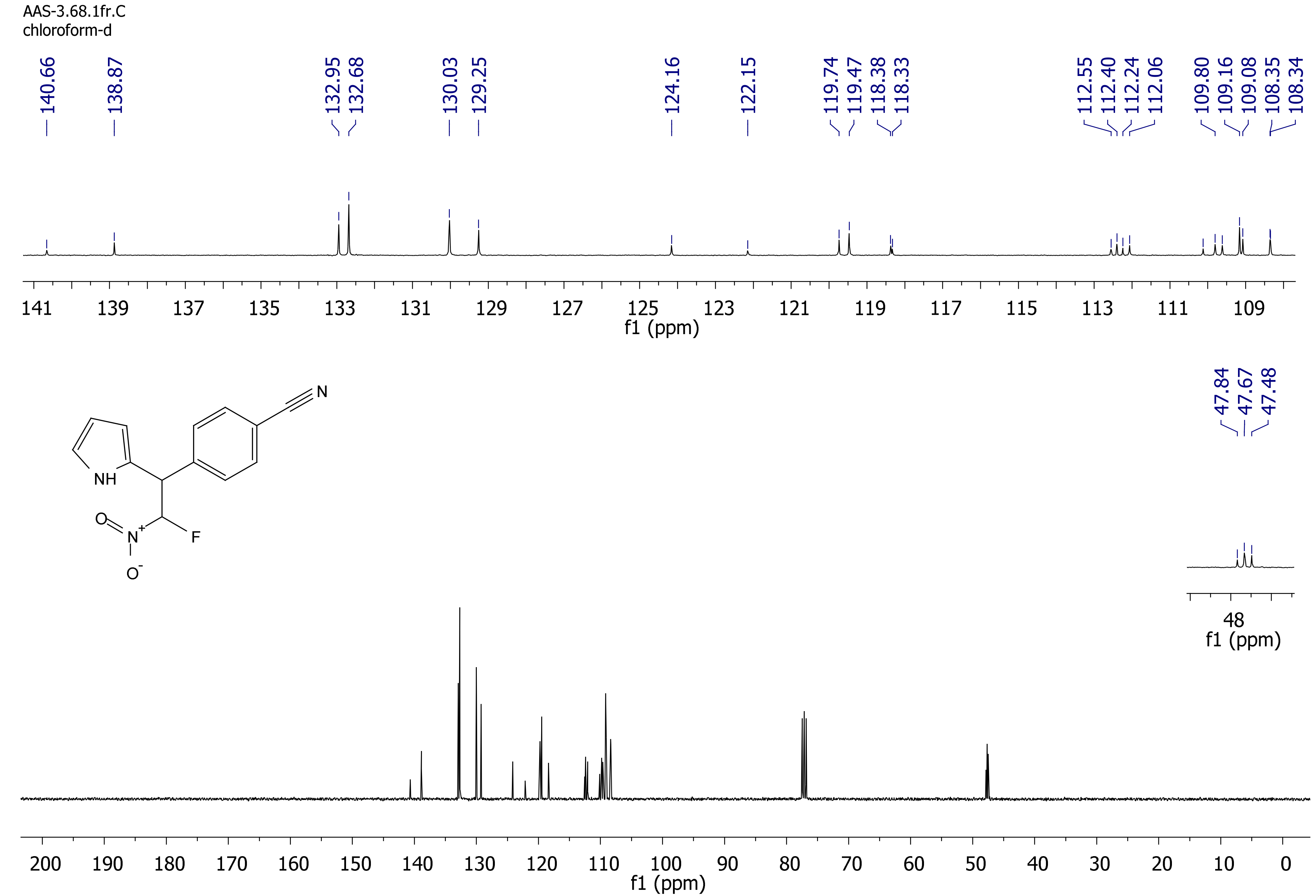

^{13}C NMR spectrum of 4-(2-fluoro-2-nitro-1-(1*H*-pyrrol-2-yl)ethyl)benzonitrile (**3k**)

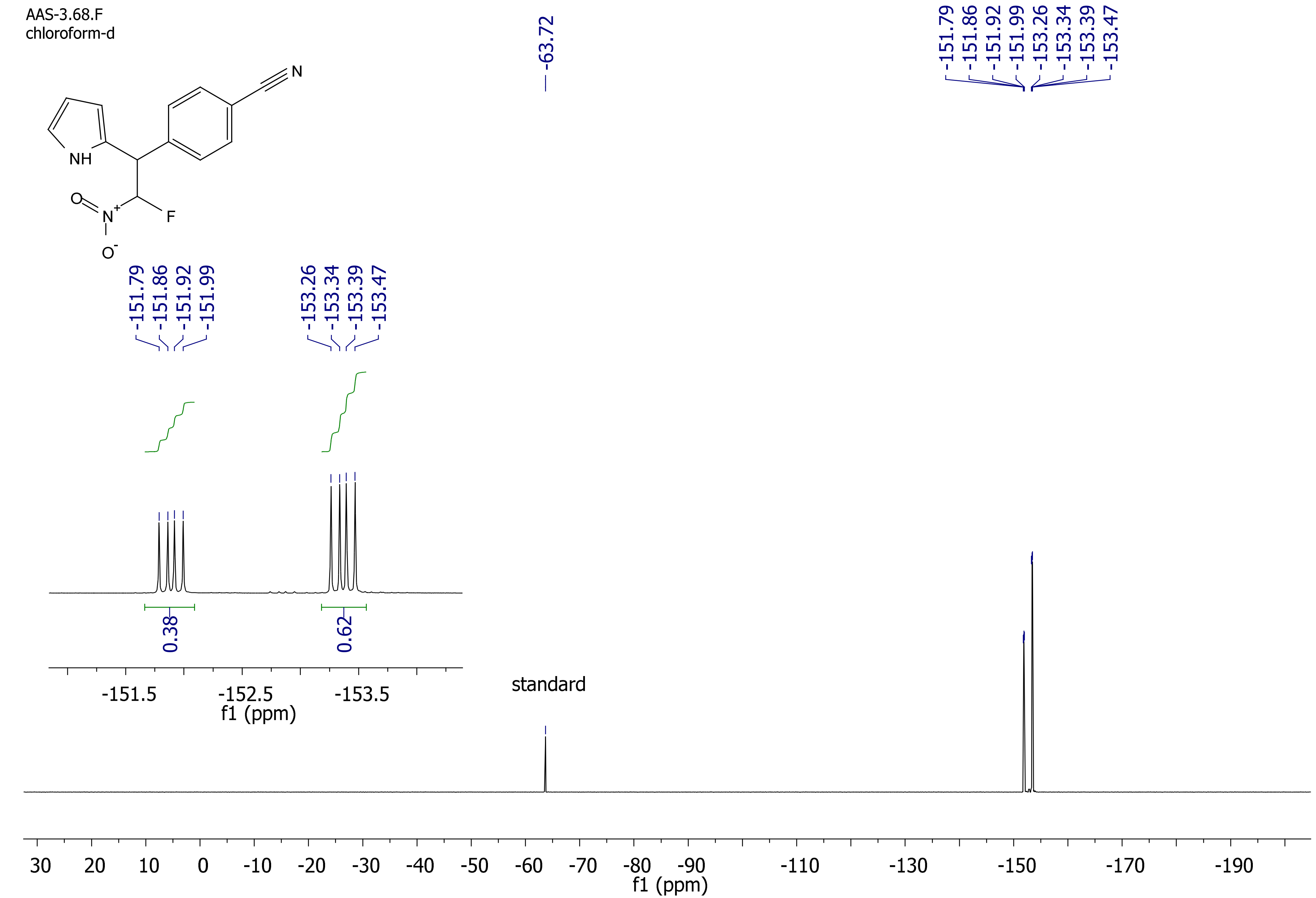

^{19}F NMR spectrum of 4-(2-fluoro-2-nitro-1-(1*H*-pyrrol-2-yl)ethyl)benzonitrile (**3k**)

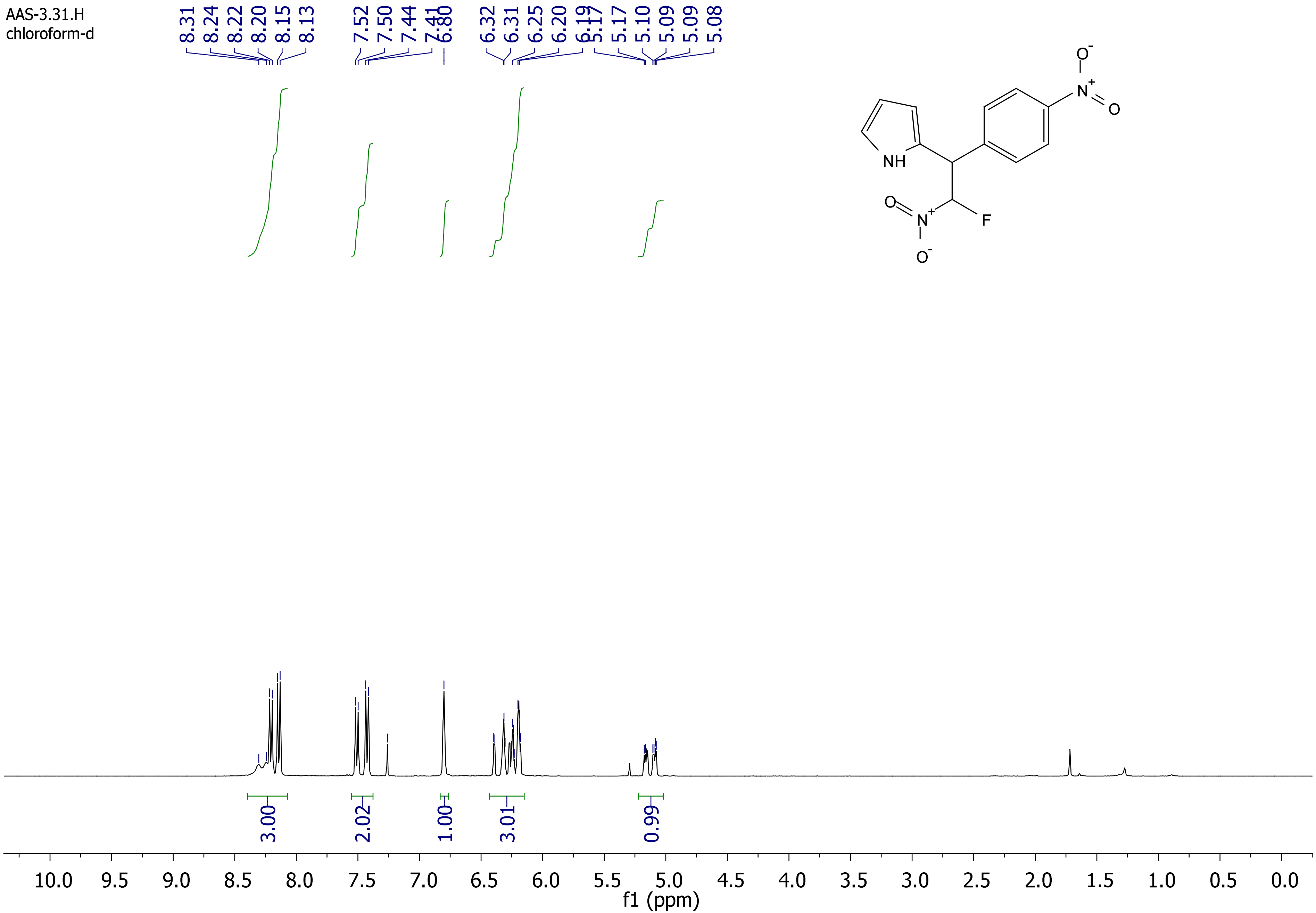

^{1}H NMR spectrum of 2-(2-fluoro-2-nitro-1-(4-nitrophenyl)ethyl)-1*H*-pyrrole (**3l**)

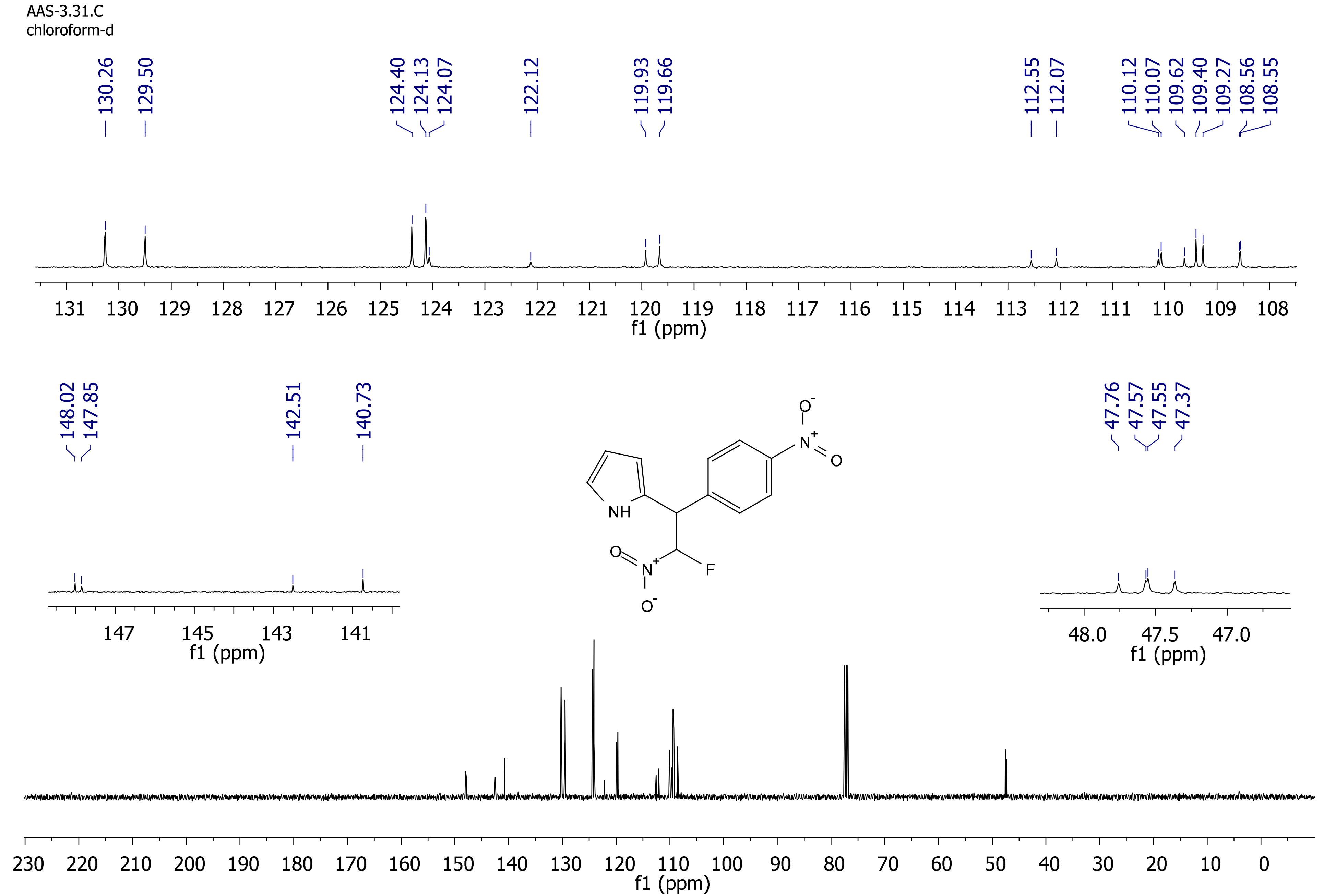

^{13}C NMR spectrum of 2-(2-fluoro-2-nitro-1-(4-nitrophenyl)ethyl)-1*H*-pyrrole (**3l**)

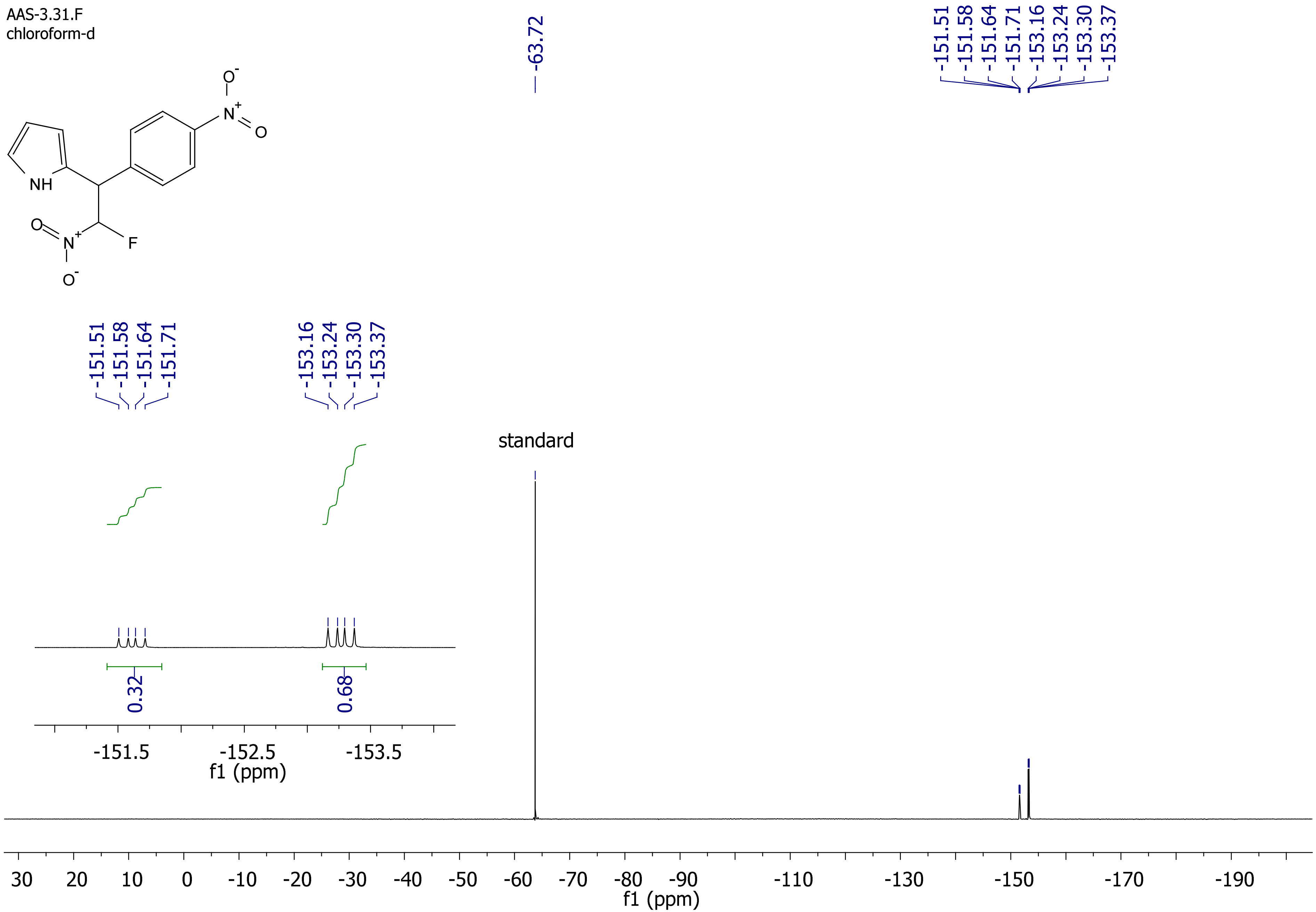

^{19}F NMR spectrum of 2-(2-fluoro-2-nitro-1-(4-nitrophenyl)ethyl)-1*H*-pyrrole (**3l**)

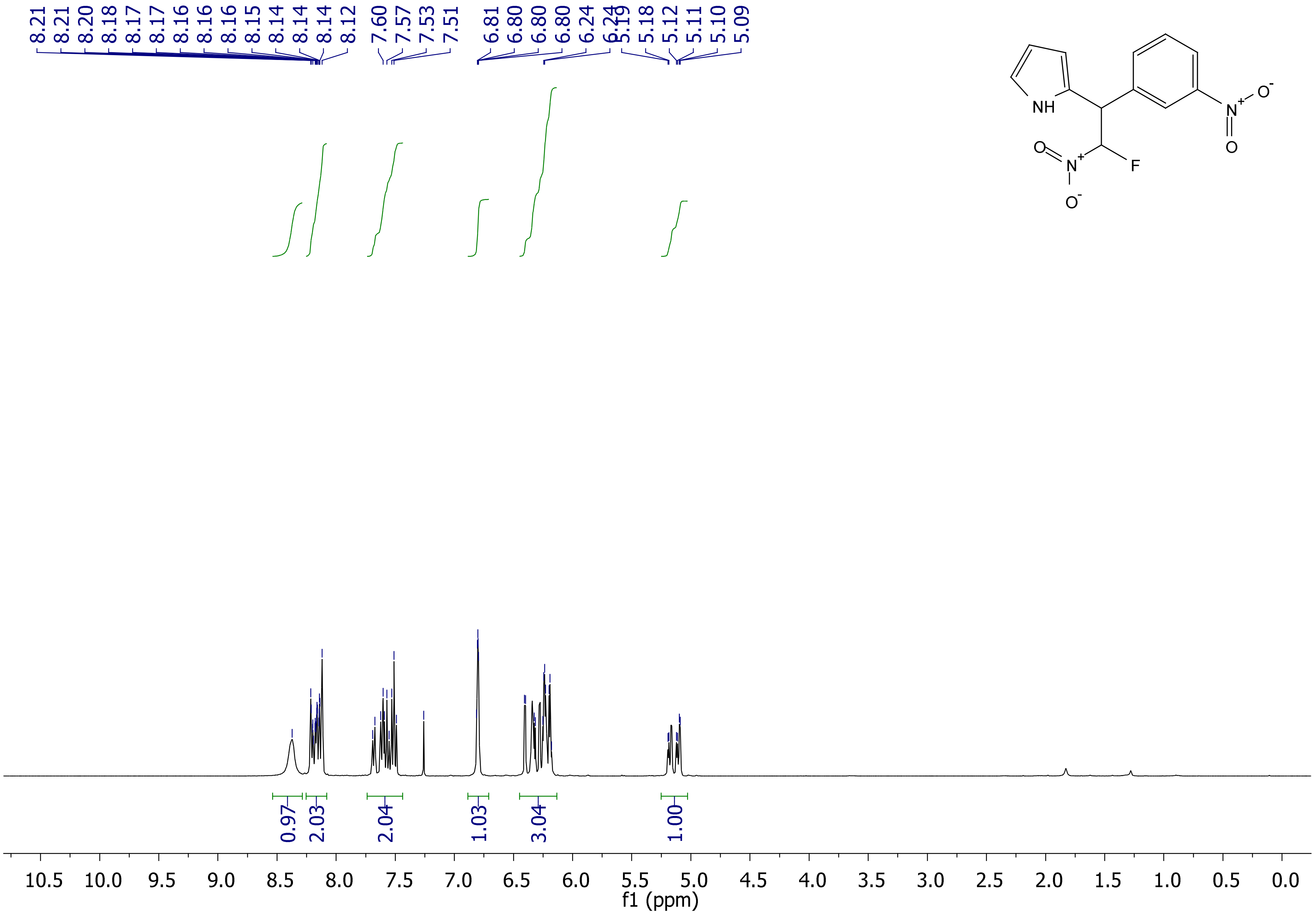

^{1}H NMR spectrum of 2-(2-fluoro-2-nitro-1-(3-nitrophenyl)ethyl)-1*H*-pyrrole (**3m**)

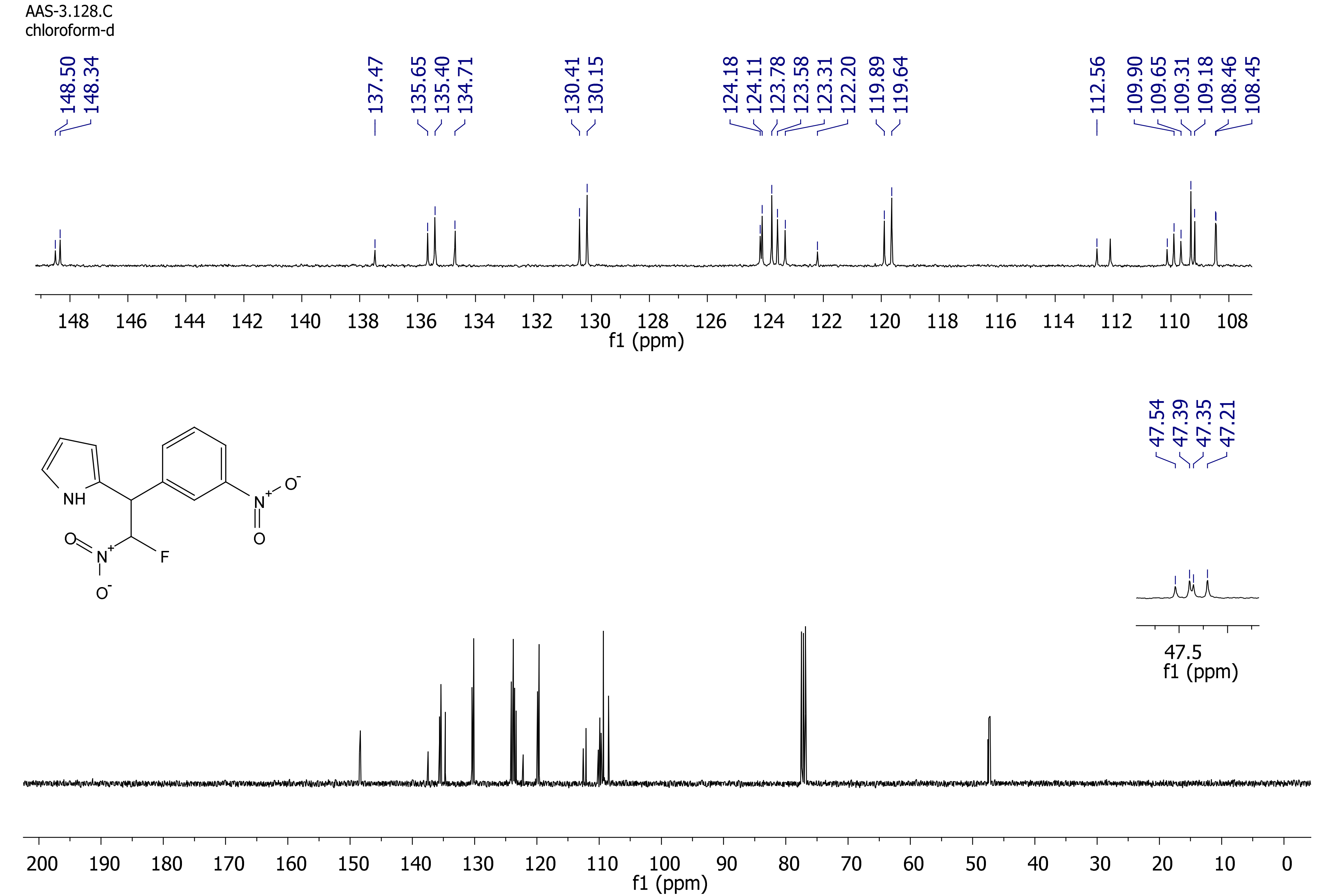

^{13}C NMR spectrum of 2-(2-fluoro-2-nitro-1-(3-nitrophenyl)ethyl)-1*H*-pyrrole (**3m**)

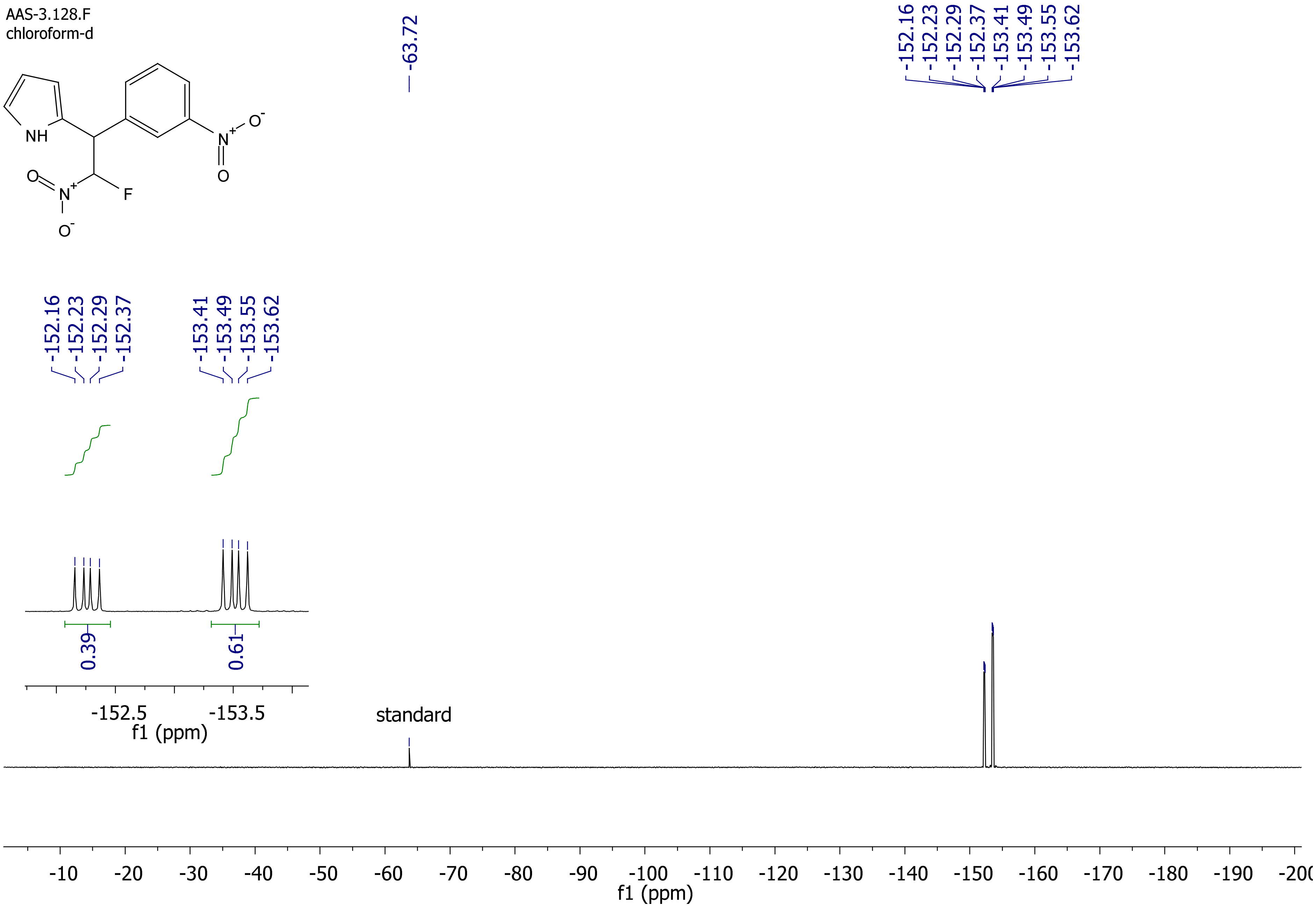

^{19}F NMR spectrum of 2-(2-fluoro-2-nitro-1-(3-nitrophenyl)ethyl)-1*H*-pyrrole (**3m**)

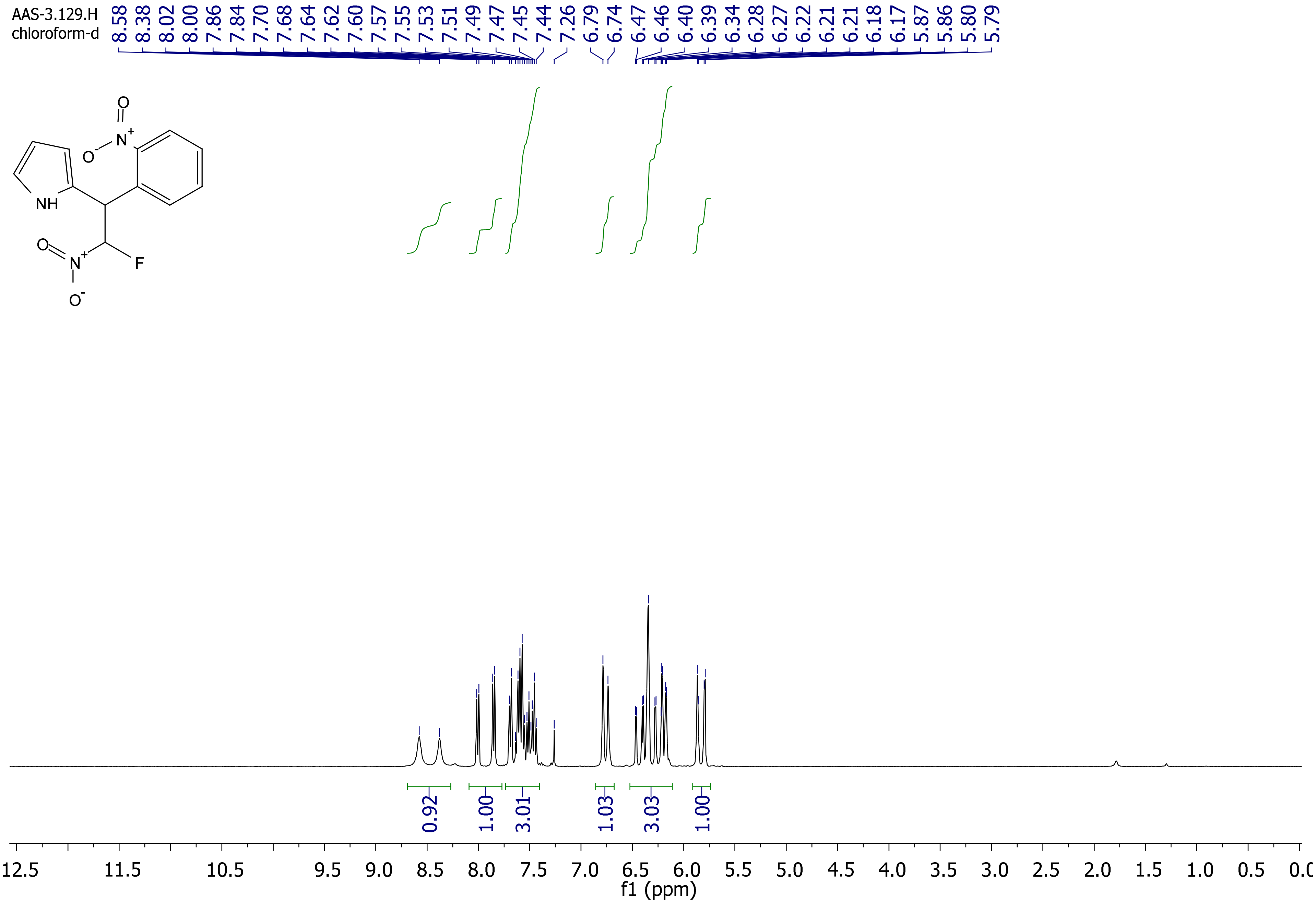

^{1}H NMR spectrum of 2-(2-fluoro-2-nitro-1-(2-nitrophenyl)ethyl)-1*H*-pyrrole (**3n**)

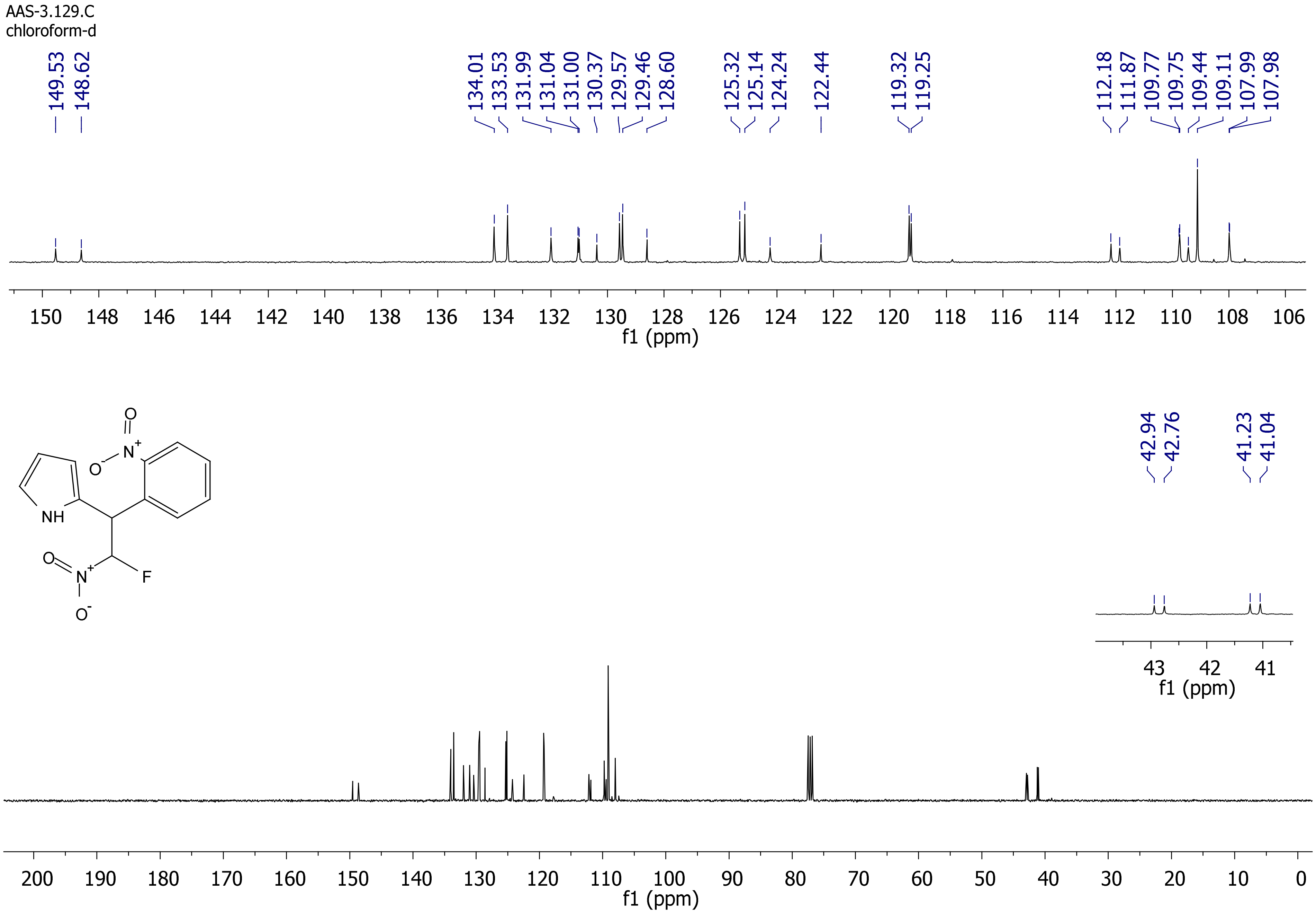

^{13}C NMR spectrum of 2-(2-fluoro-2-nitro-1-(2-nitrophenyl)ethyl)-1*H*-pyrrole (**3n**)

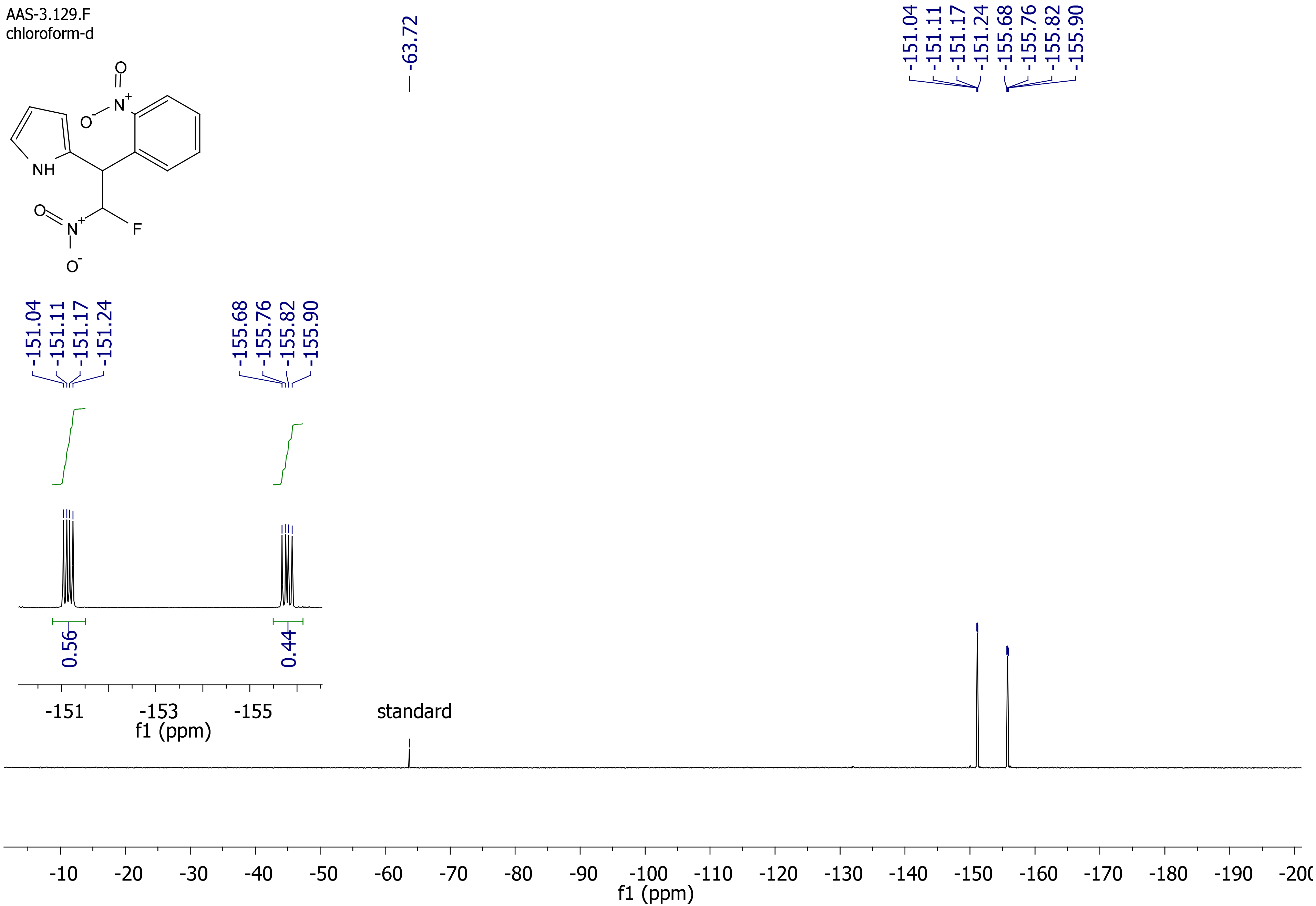

^{19}F NMR spectrum of 2-(2-fluoro-2-nitro-1-(2-nitrophenyl)ethyl)-1*H*-pyrrole (**3n**)

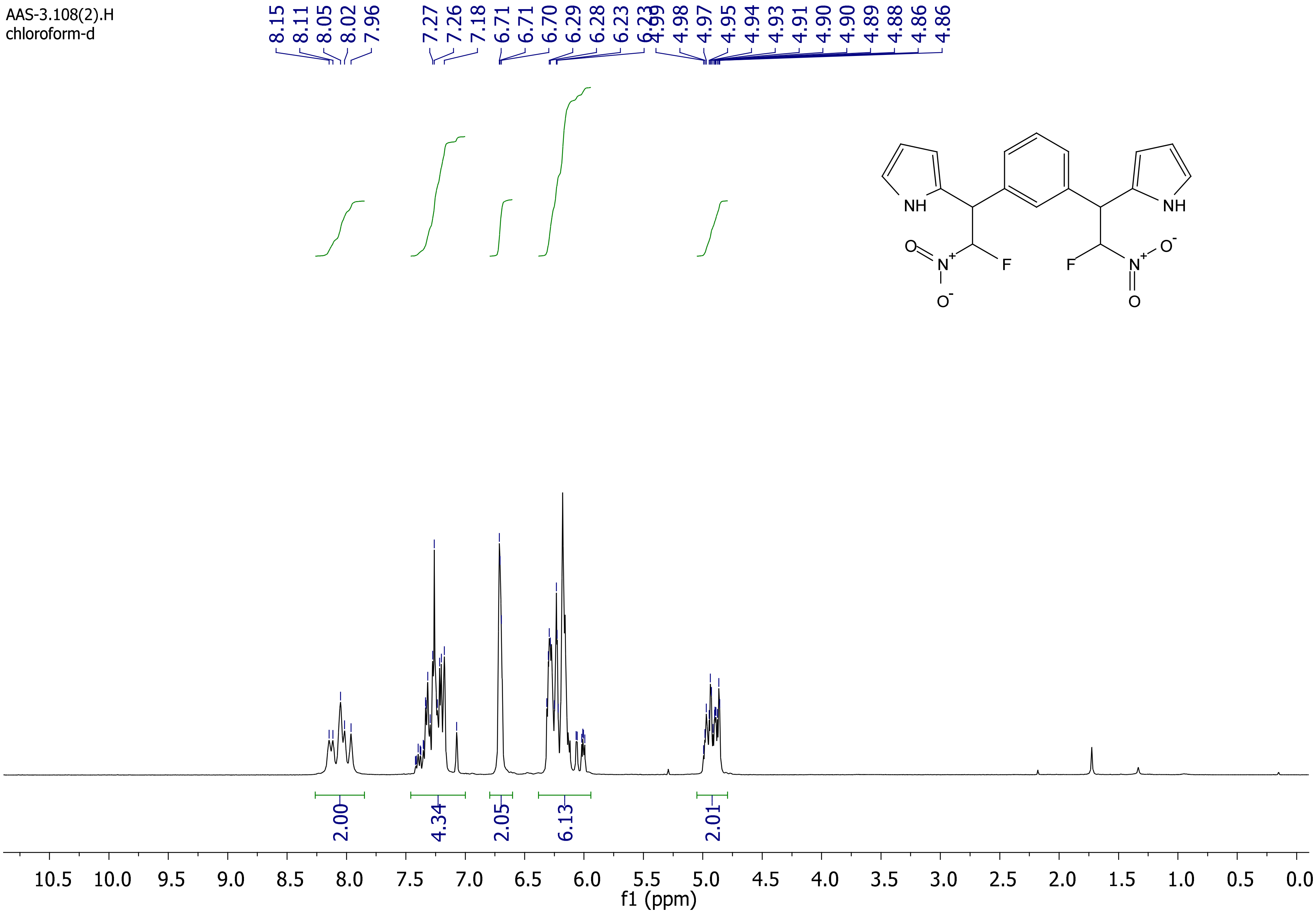

^{1}H NMR spectrum of 1,3-bis(2-fluoro-2-nitro-1-(1H-pyrrol-2-yl)ethyl)benzene (**3o**)

AAS-3.108(2).C
chloroform-d

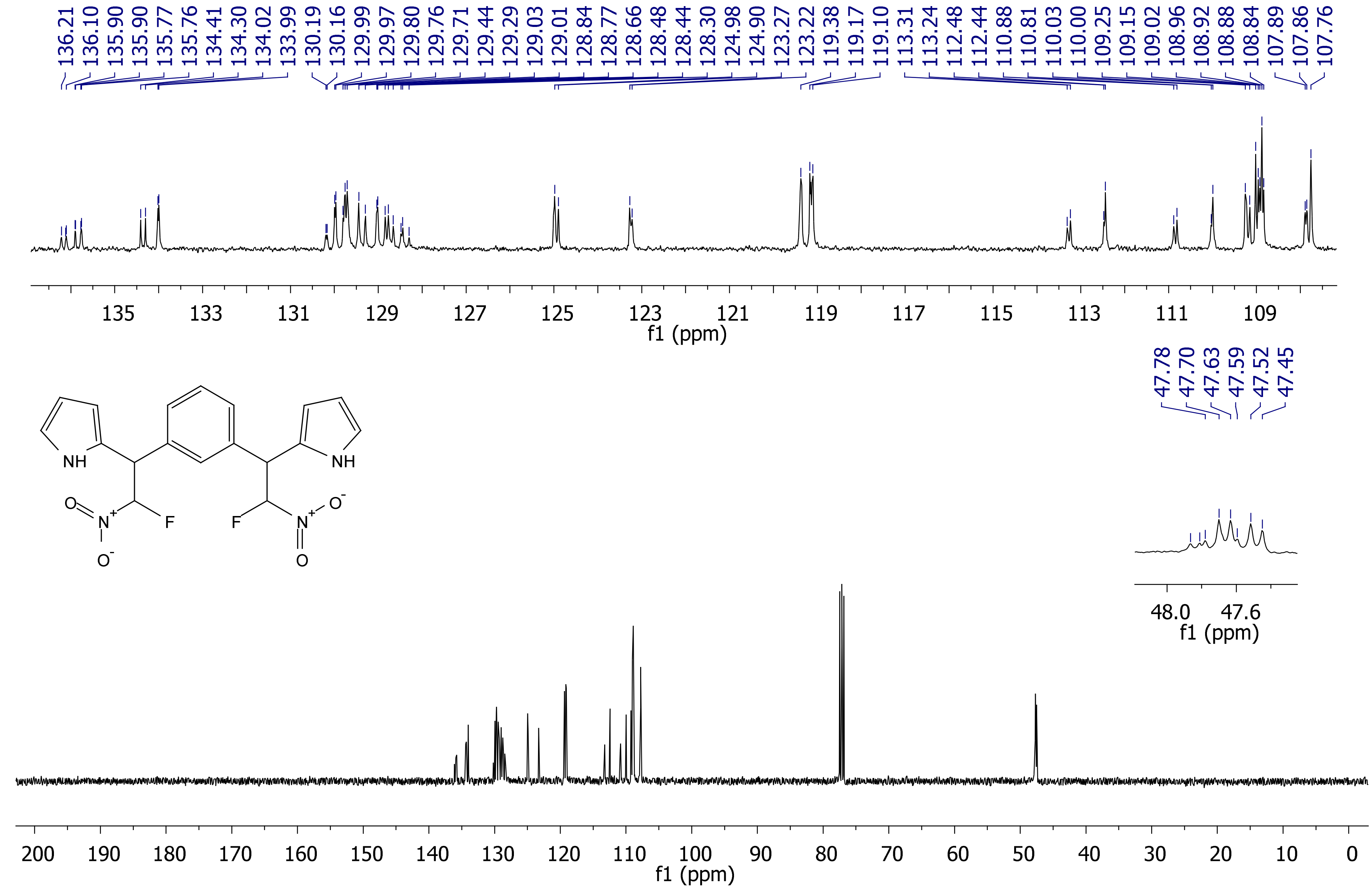

^{13}C NMR spectrum of 2-(2-fluoro-2-nitro-1-(2-nitrophenyl)ethyl)-1*H*-pyrrole (**3o**)

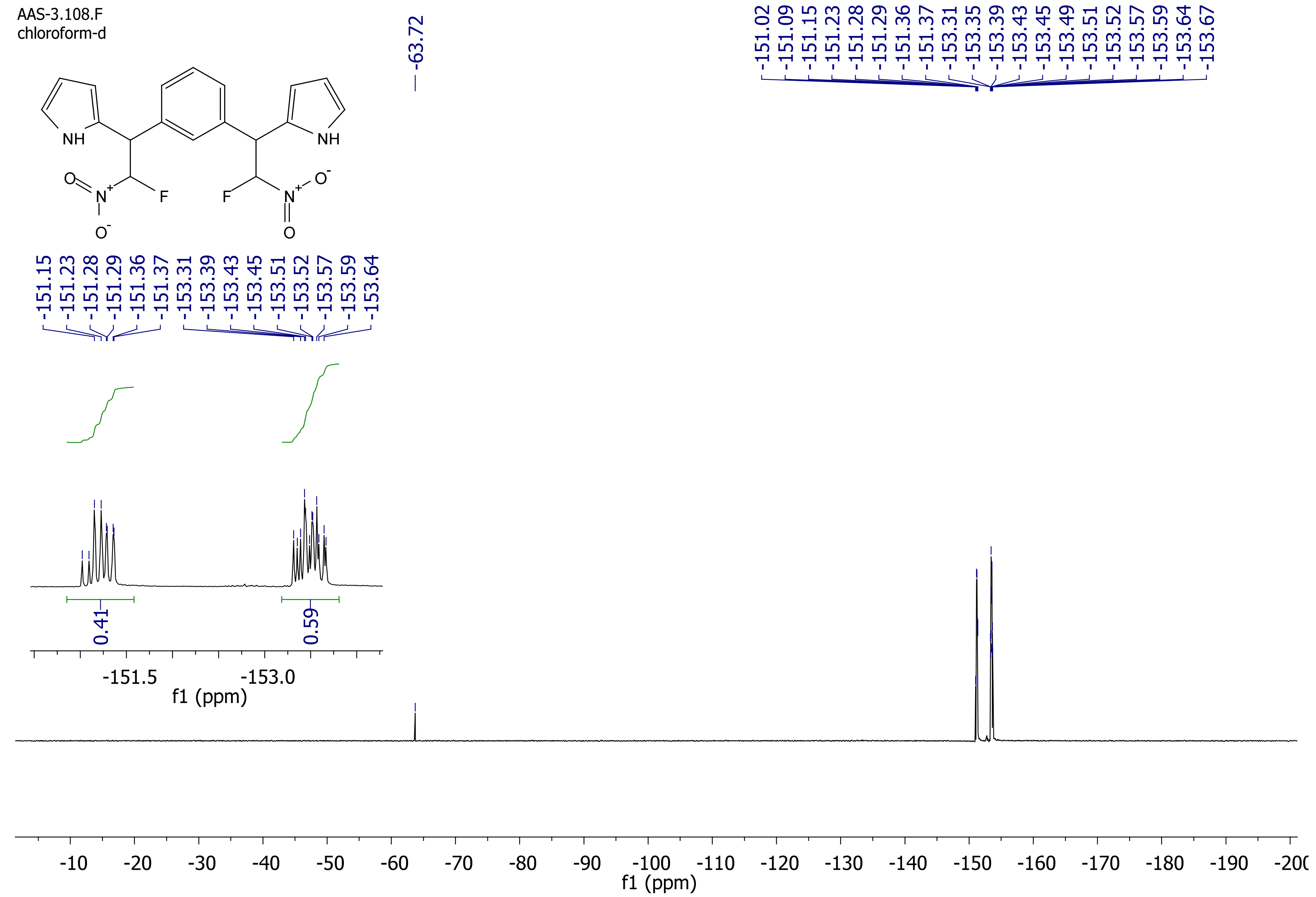

^{19}F NMR spectrum of 2-(2-fluoro-2-nitro-1-(2-nitrophenyl)ethyl)-1*H*-pyrrole (**3o**)

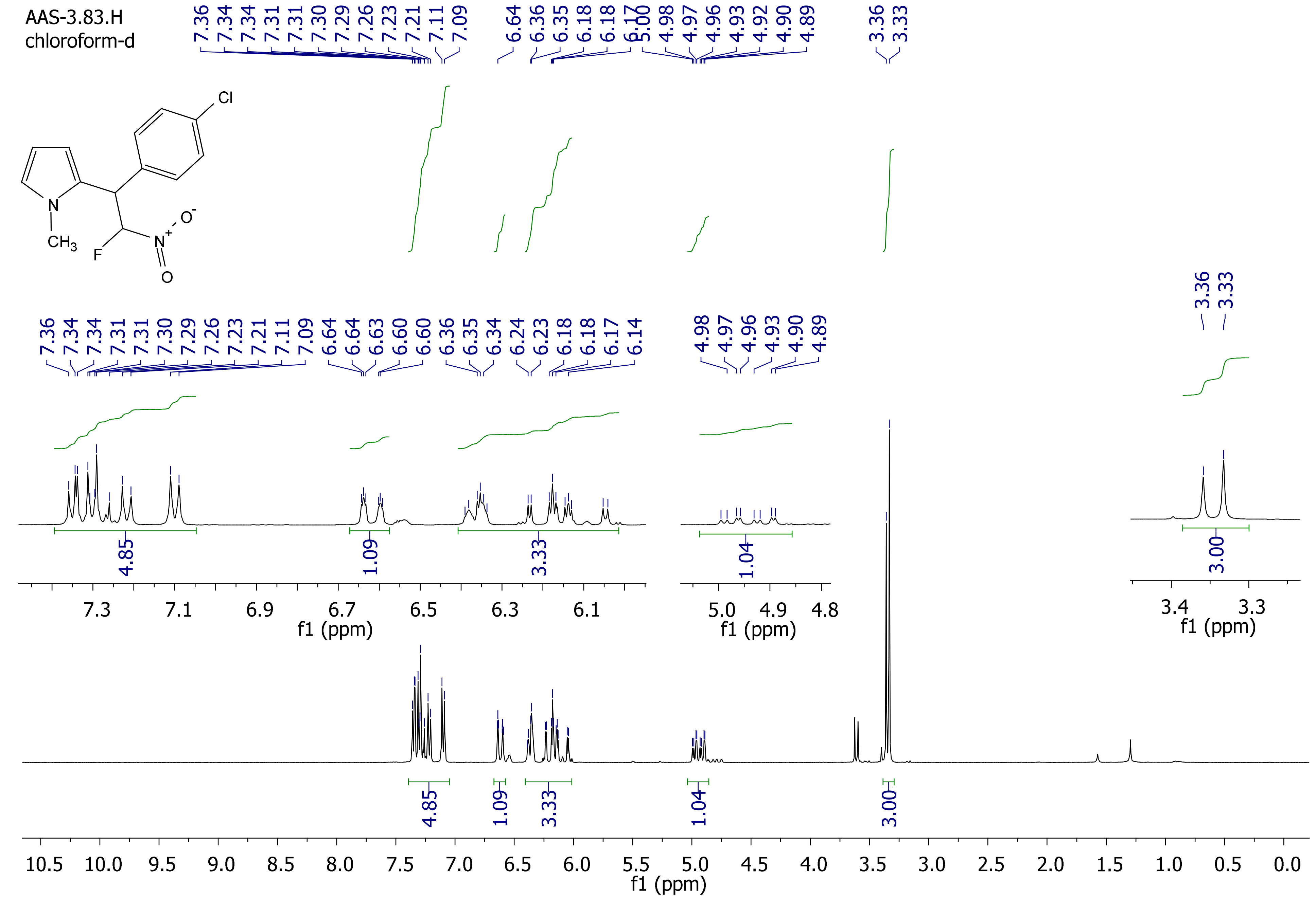

^{1}H NMR spectrum of 2-(1-(4-chlorophenyl)-2-fluoro-2-nitroethyl)-1-methyl-1*H*-pyrrole (**3p**)

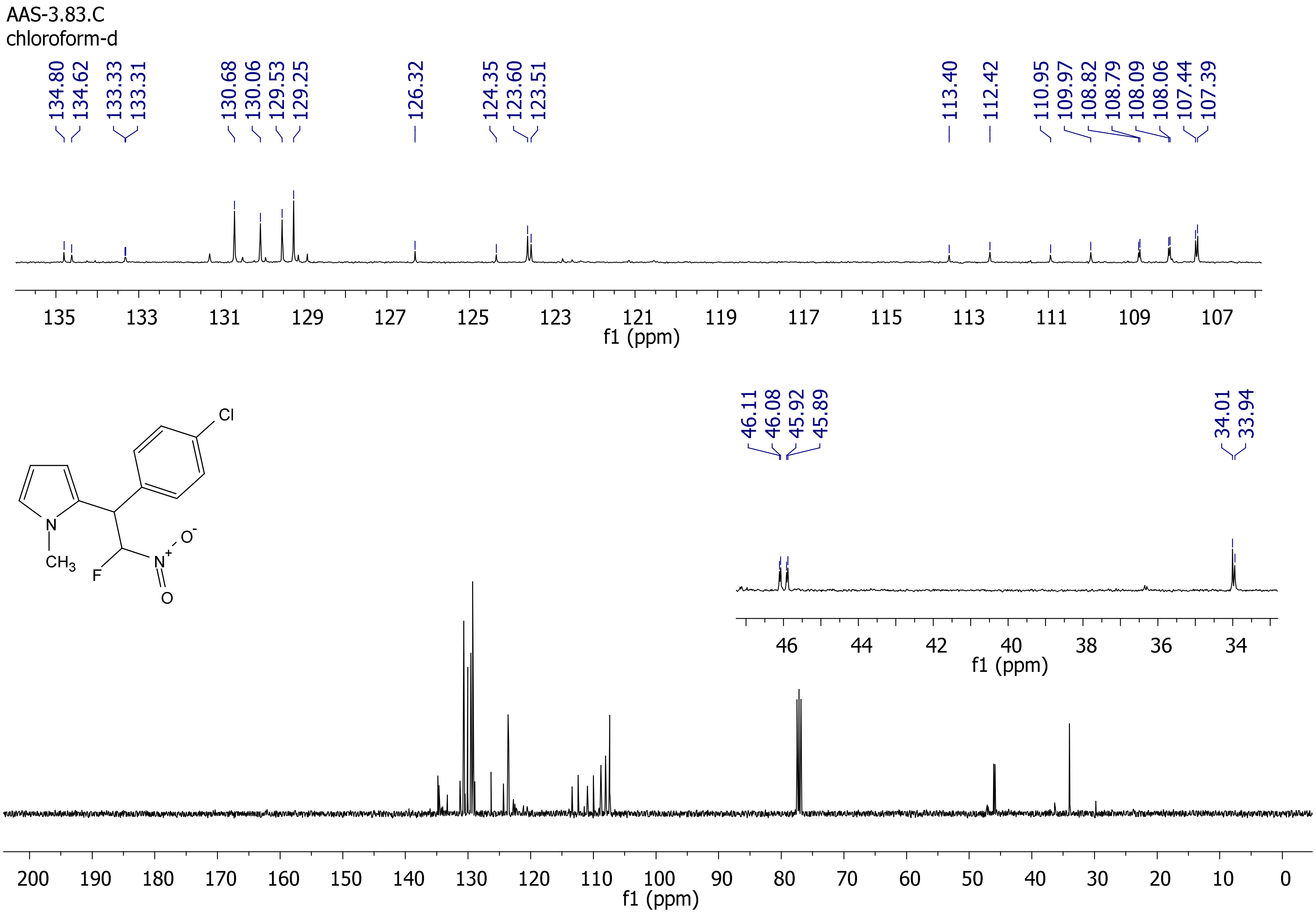

^{13}C NMR spectrum of 2-(1-(4-chlorophenyl)-2-fluoro-2-nitroethyl)-1-methyl-1*H*-pyrrole (**3p**)

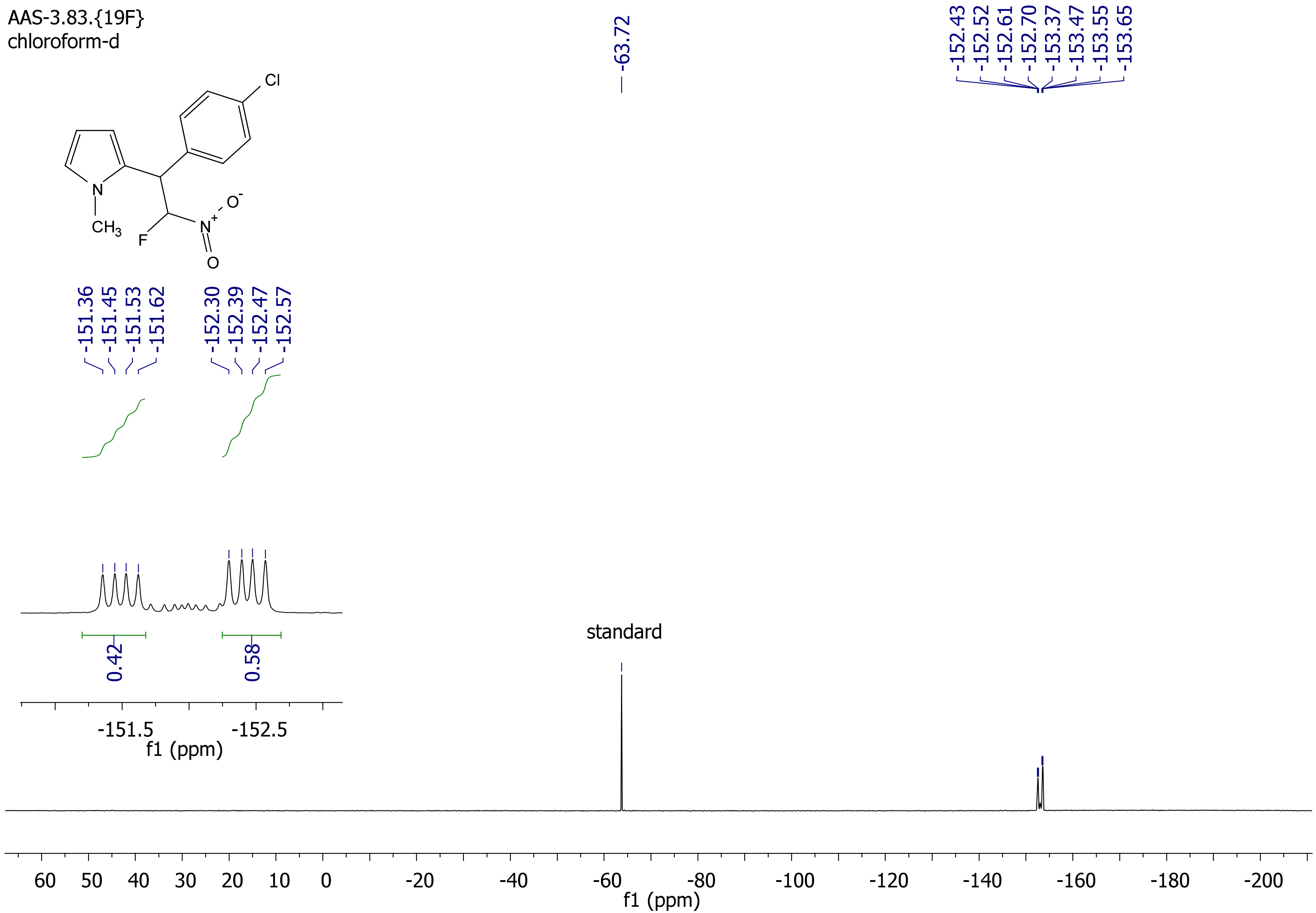

^{19}F NMR spectrum of 2-(1-(4-chlorophenyl)-2-fluoro-2-nitroethyl)-1-methyl-1*H*-pyrrole (**3p**)

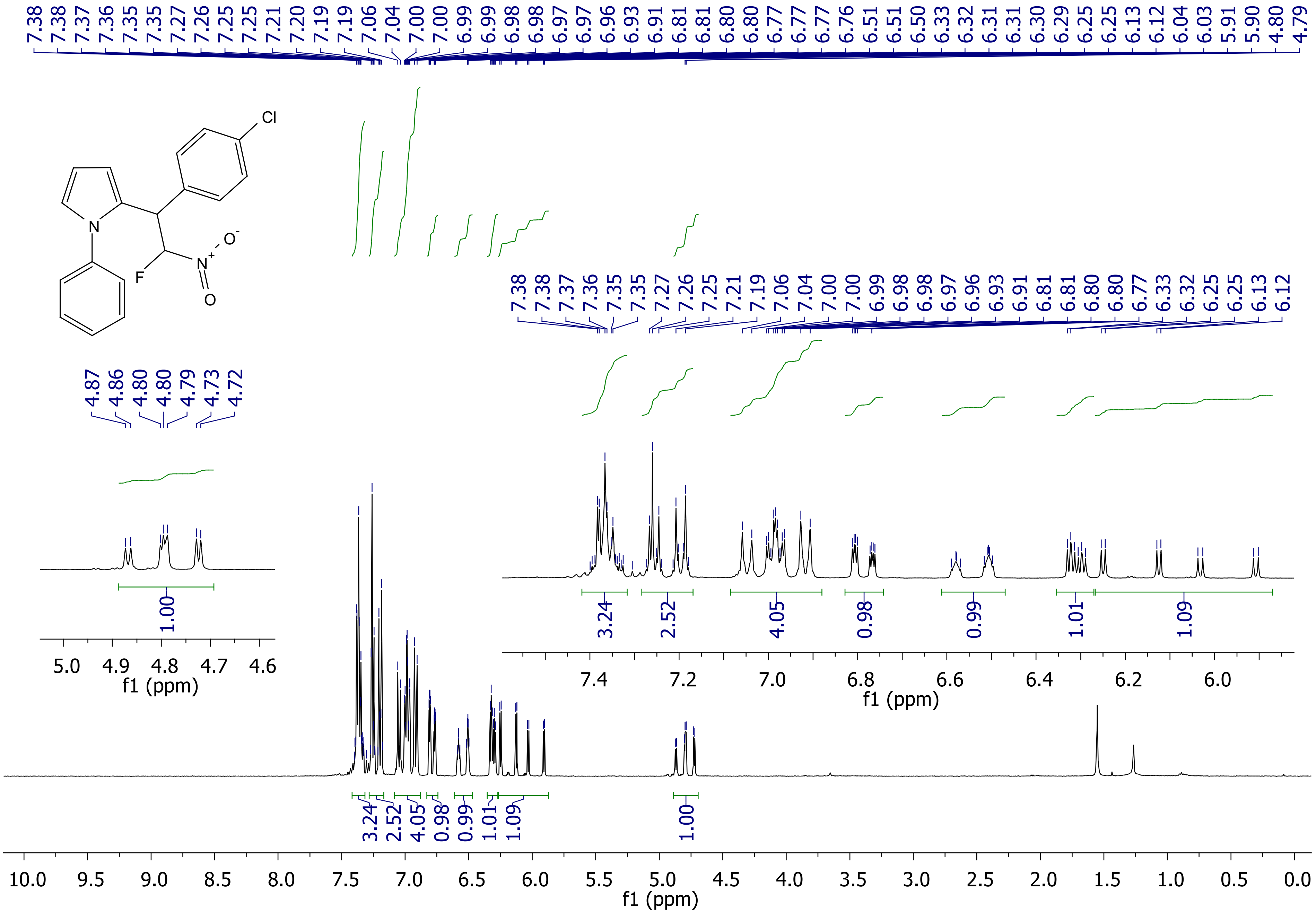

^{1}H NMR spectrum of 2-(1-(4-chlorophenyl)-2-fluoro-2-nitroethyl)-1-phenyl-1*H*-pyrrole (**3q**)

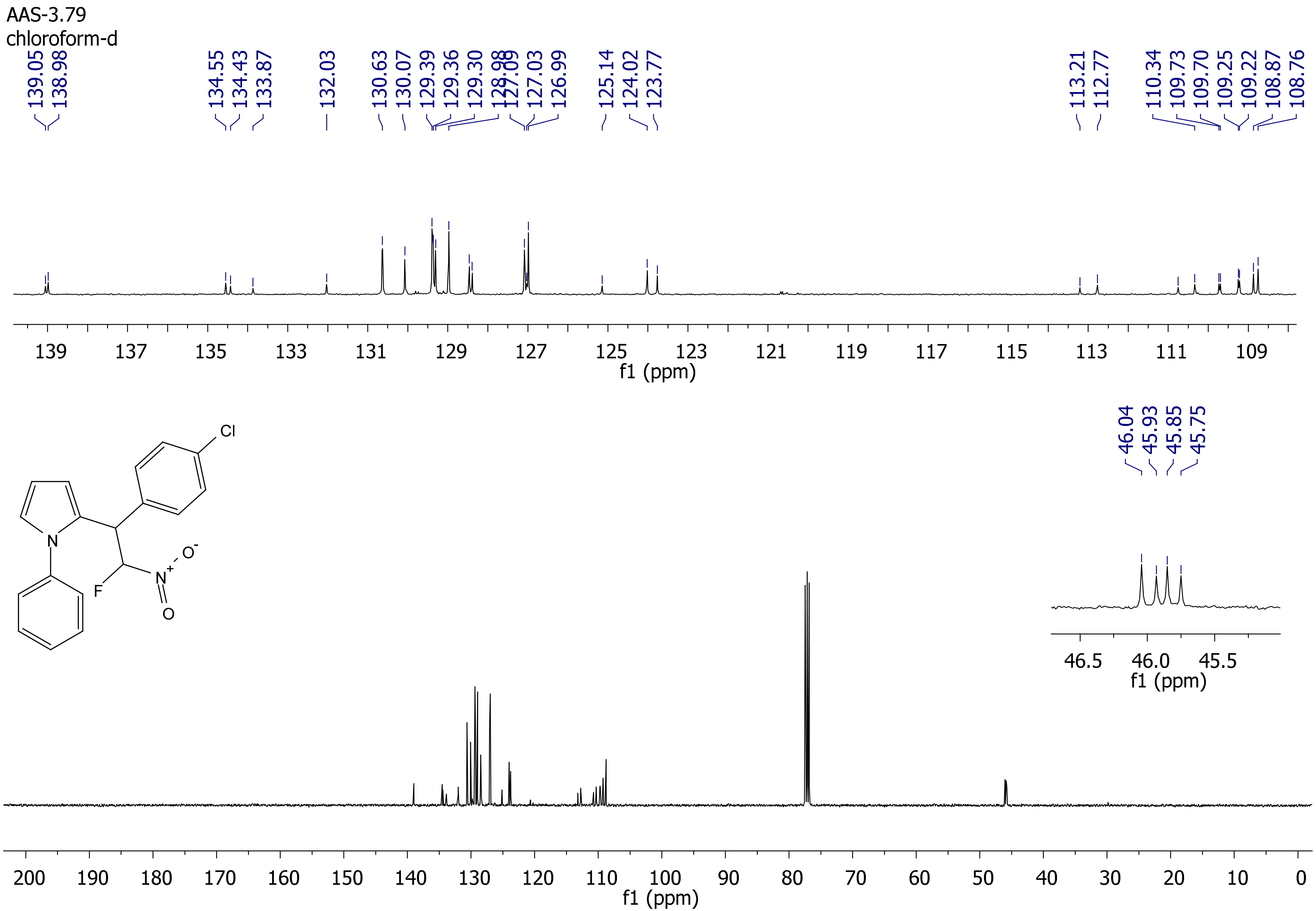

^{13}C NMR spectrum of 2-(1-(4-chlorophenyl)-2-fluoro-2-nitroethyl)-1-phenyl-1*H*-pyrrole (**3q**)

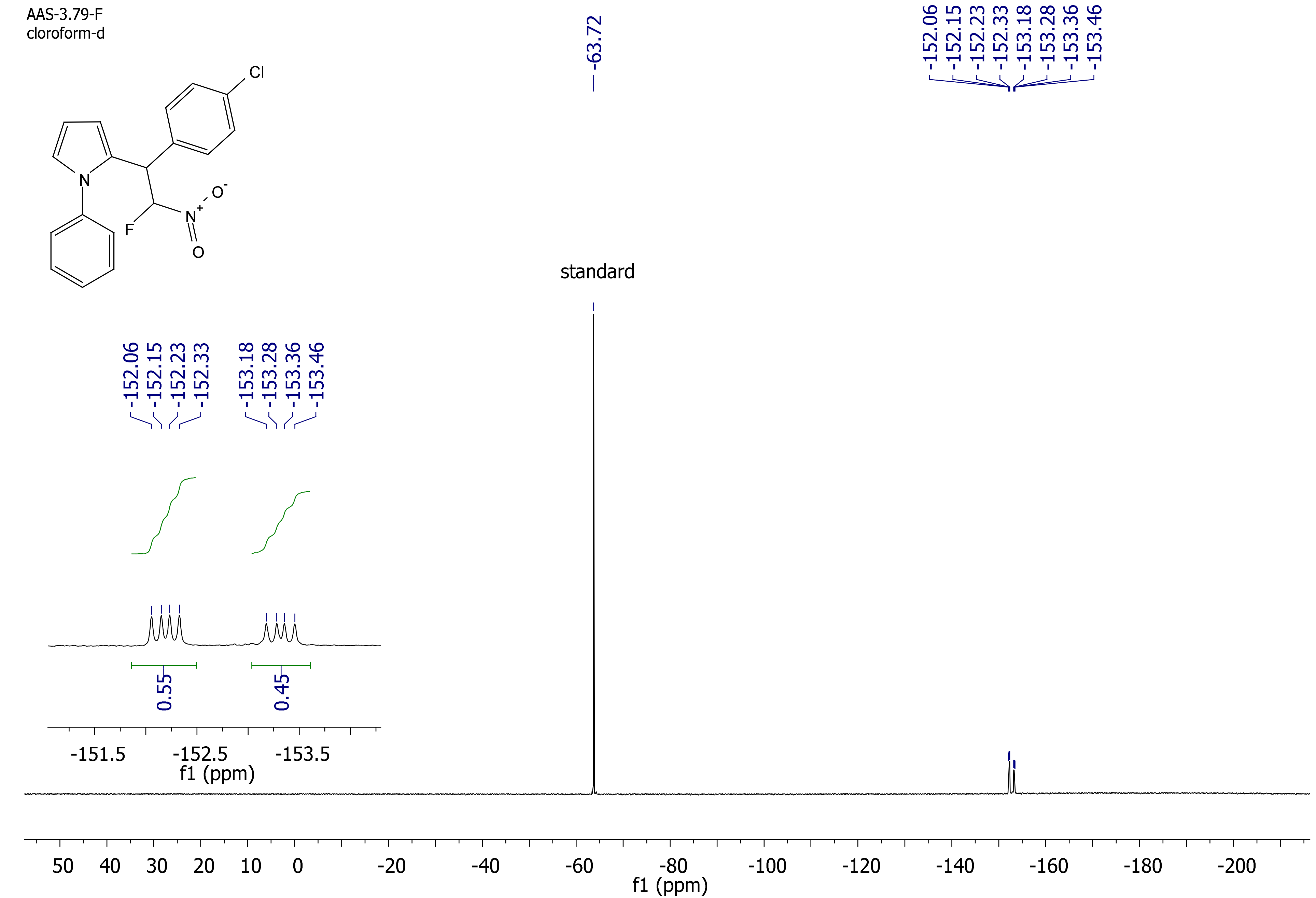

^{19}F NMR spectrum of 2-(1-(4-chlorophenyl)-2-fluoro-2-nitroethyl)-1-phenyl-1*H*-pyrrole (**3q**)

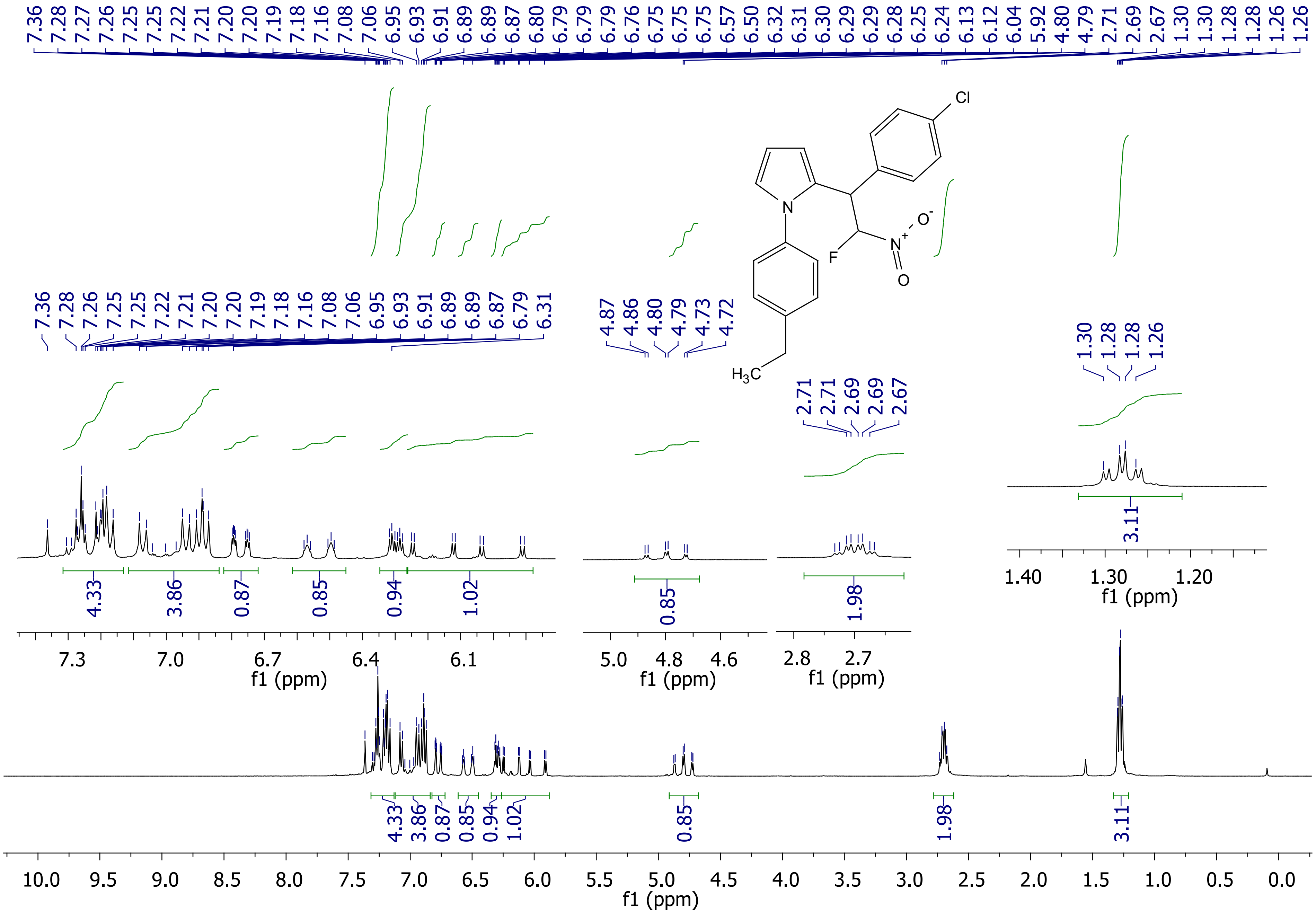

^{1}H NMR spectrum of 2-(1-(4-chlorophenyl)-2-fluoro-2-nitroethyl)-1-(4-ethylphenyl)-1*H*-pyrrole (**3r**)

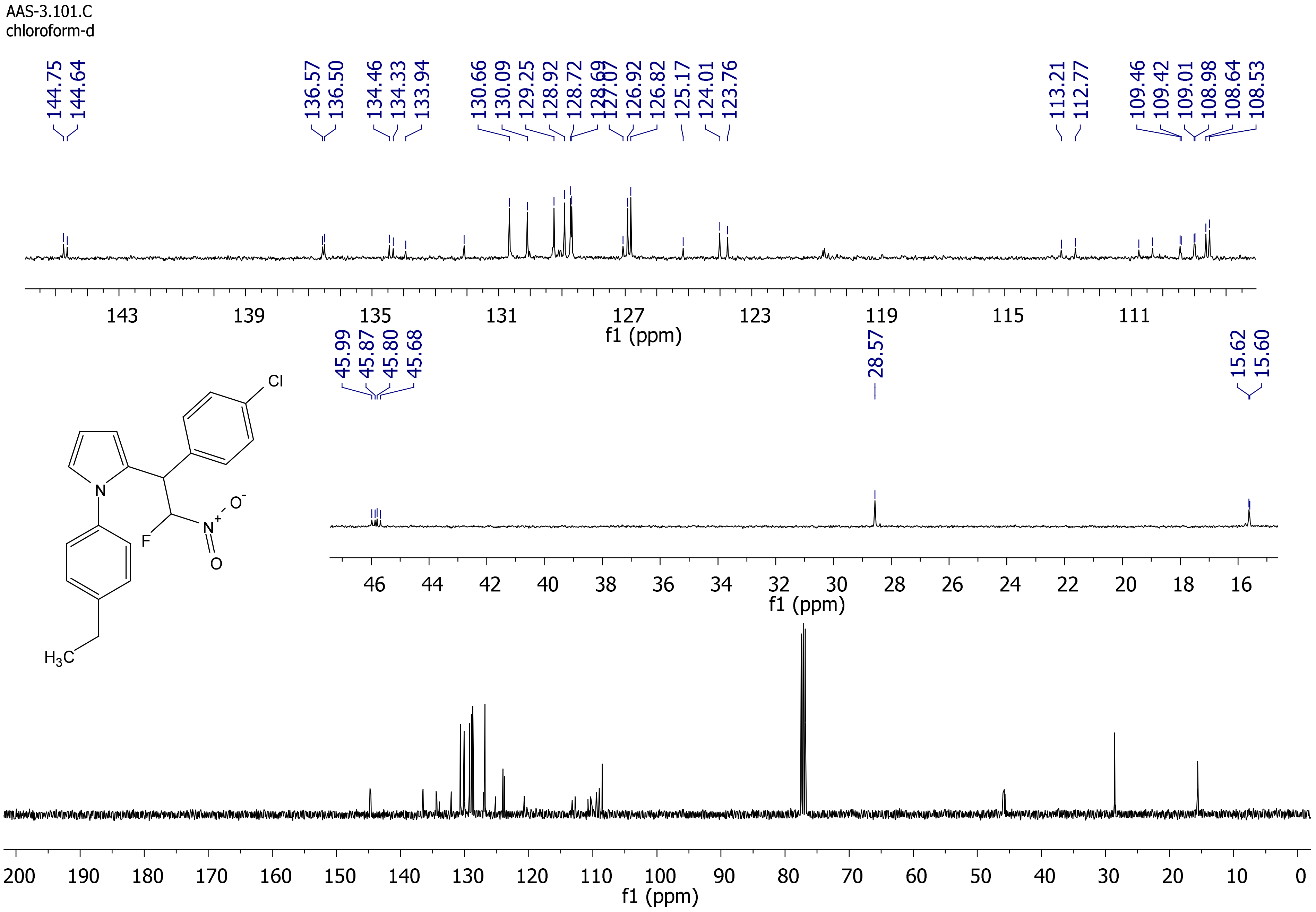

^{13}C NMR spectrum of 2-(1-(4-chlorophenyl)-2-fluoro-2-nitroethyl)-1-(4-ethylphenyl)-1*H*-pyrrole (**3r**)

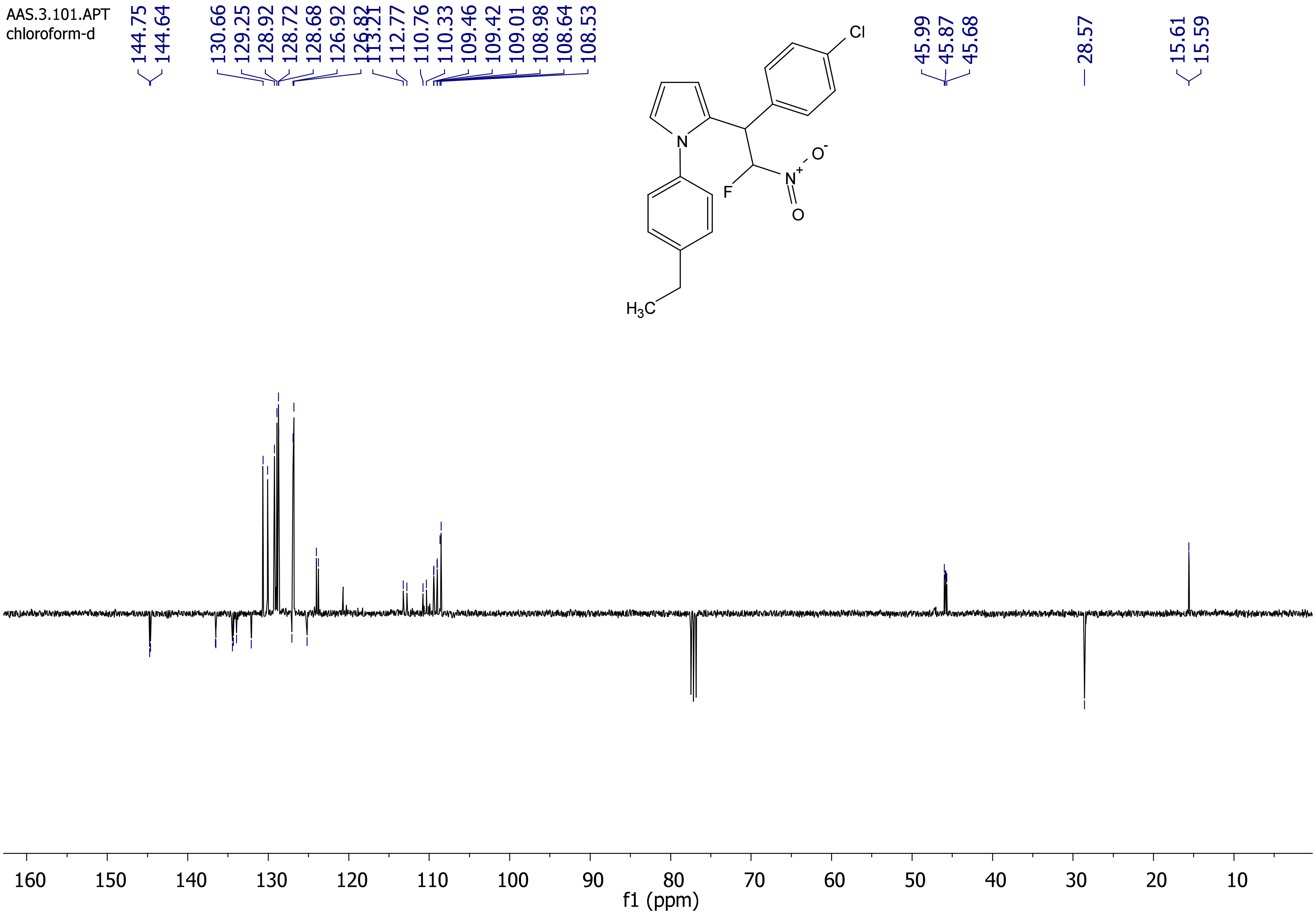

^{13}C APT NMR spectrum of 2-(1-(4-chlorophenyl)-2-fluoro-2-nitroethyl)-1-(4-ethylphenyl)-1*H*-pyrrole (**3r**)

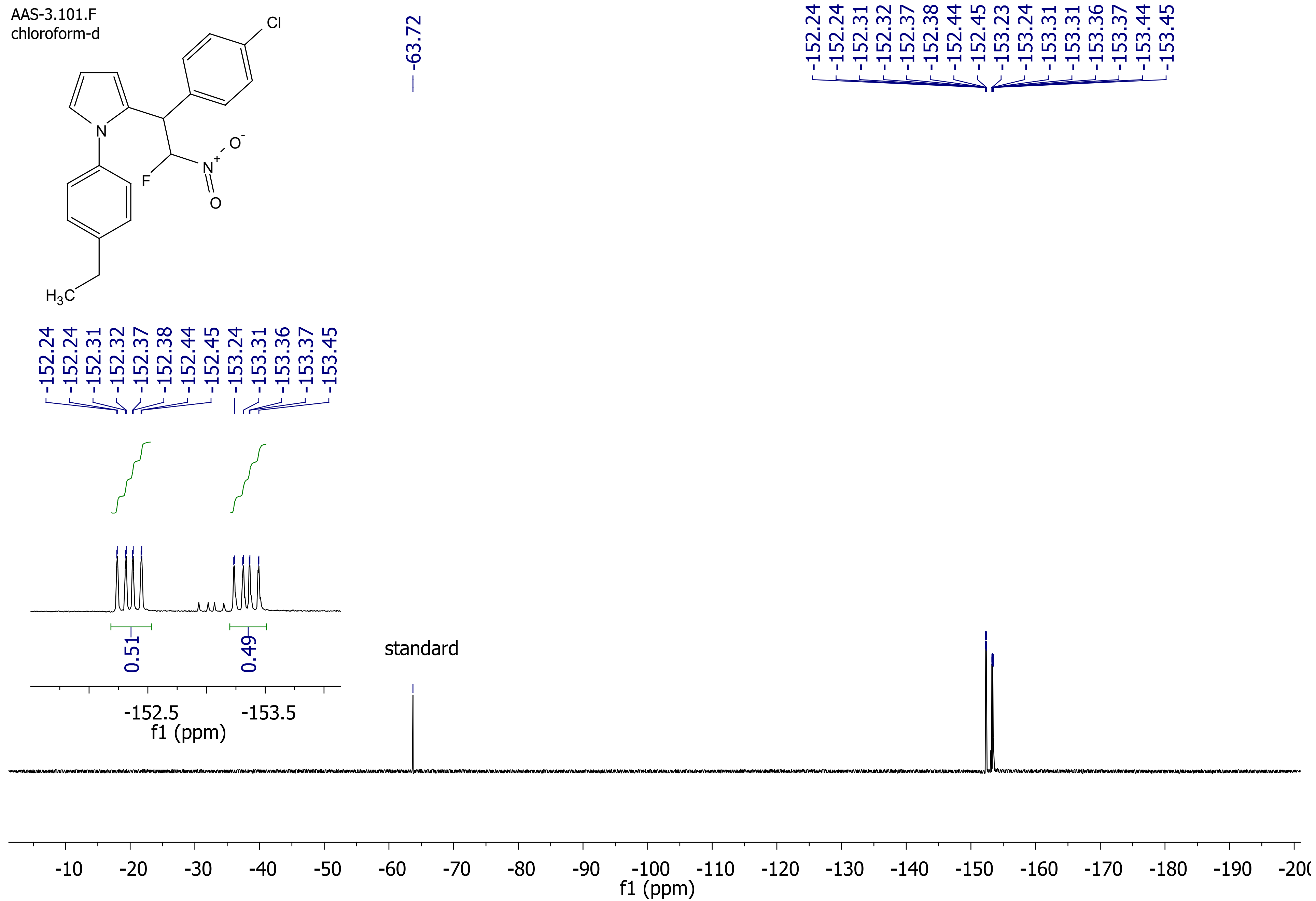

^{19}F NMR spectrum of 2-(1-(4-chlorophenyl)-2-fluoro-2-nitroethyl)-1-(4-ethylphenyl)-1*H*-pyrrole (**3r**)

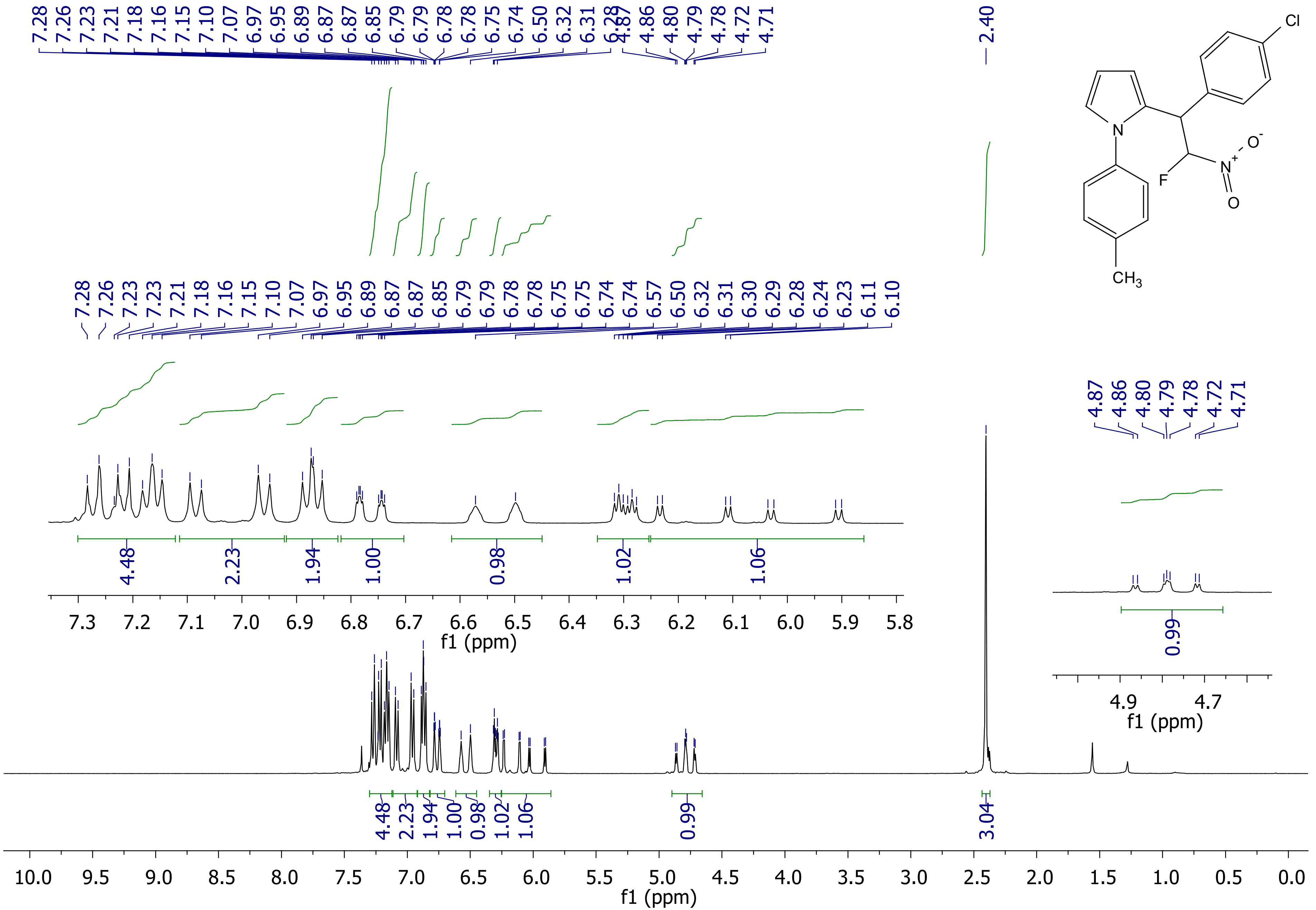

^{1}H NMR spectrum of 2-(1-(4-chlorophenyl)-2-fluoro-2-nitroethyl)-1-(p-tolyl)-1*H*-pyrrole (**3s**)

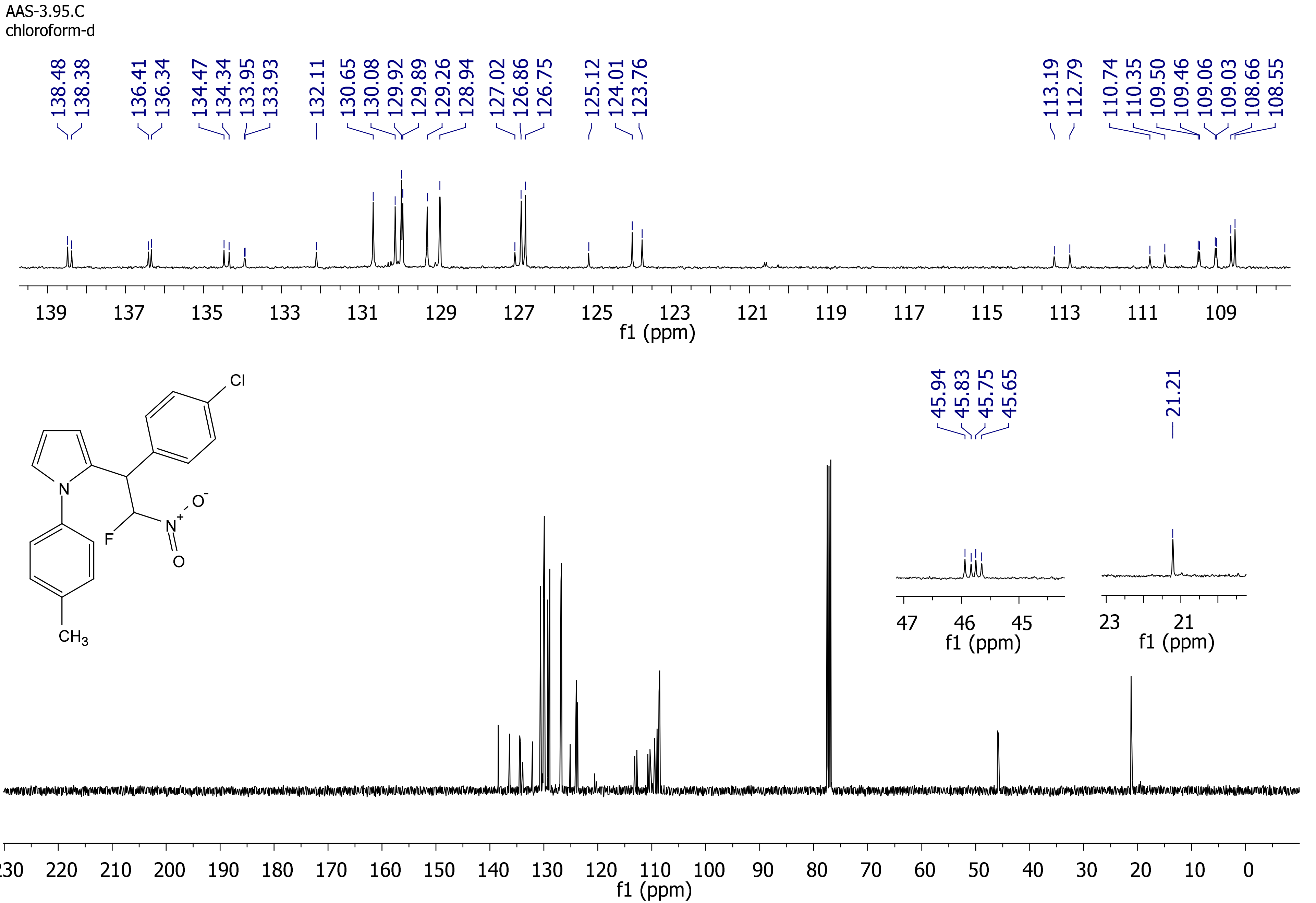

^{13}C NMR spectrum of 2-(1-(4-chlorophenyl)-2-fluoro-2-nitroethyl)-1-(p-tolyl)-1*H*-pyrrole (**3s**)

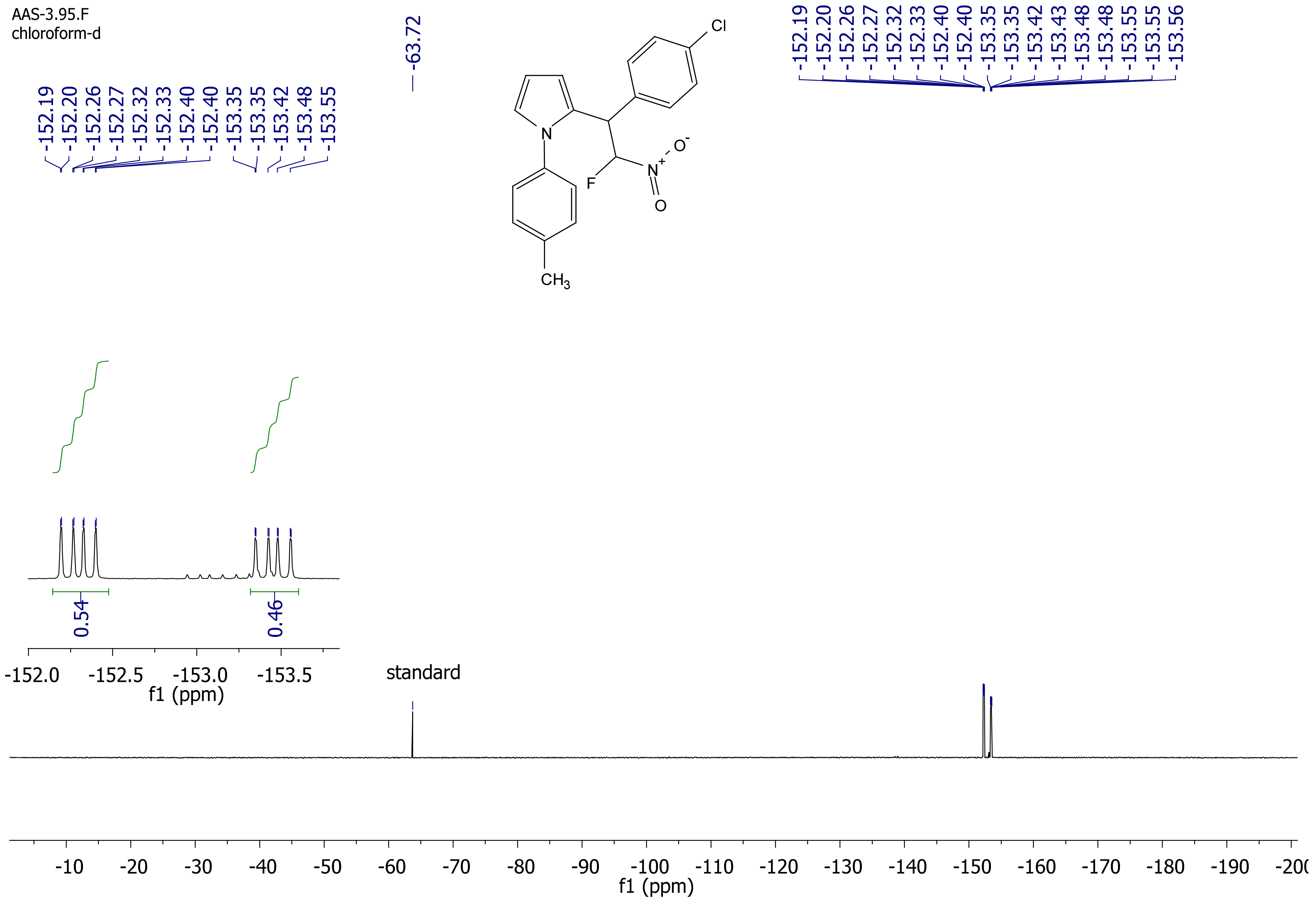

^{19}F NMR spectrum of 2-(1-(4-chlorophenyl)-2-fluoro-2-nitroethyl)-1-(p-tolyl)-1*H*-pyrrole (**3s**)

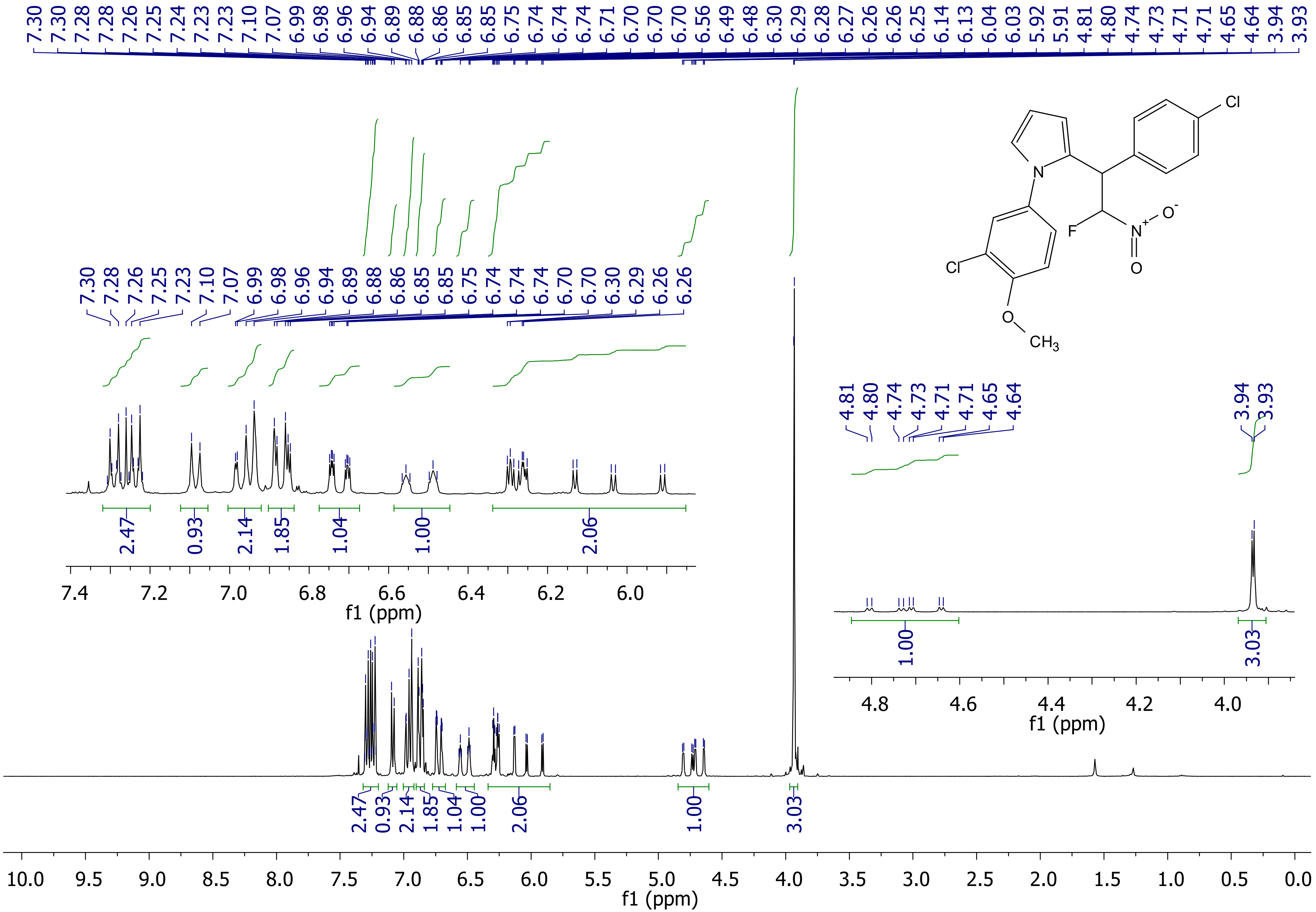

^{1}H NMR spectrum of 1-(3-chloro-4-methoxyphenyl)-2-(1-(4-chlorophenyl)-2-fluoro-2-nitroethyl)-1*H*-pyrrole (**3t**)

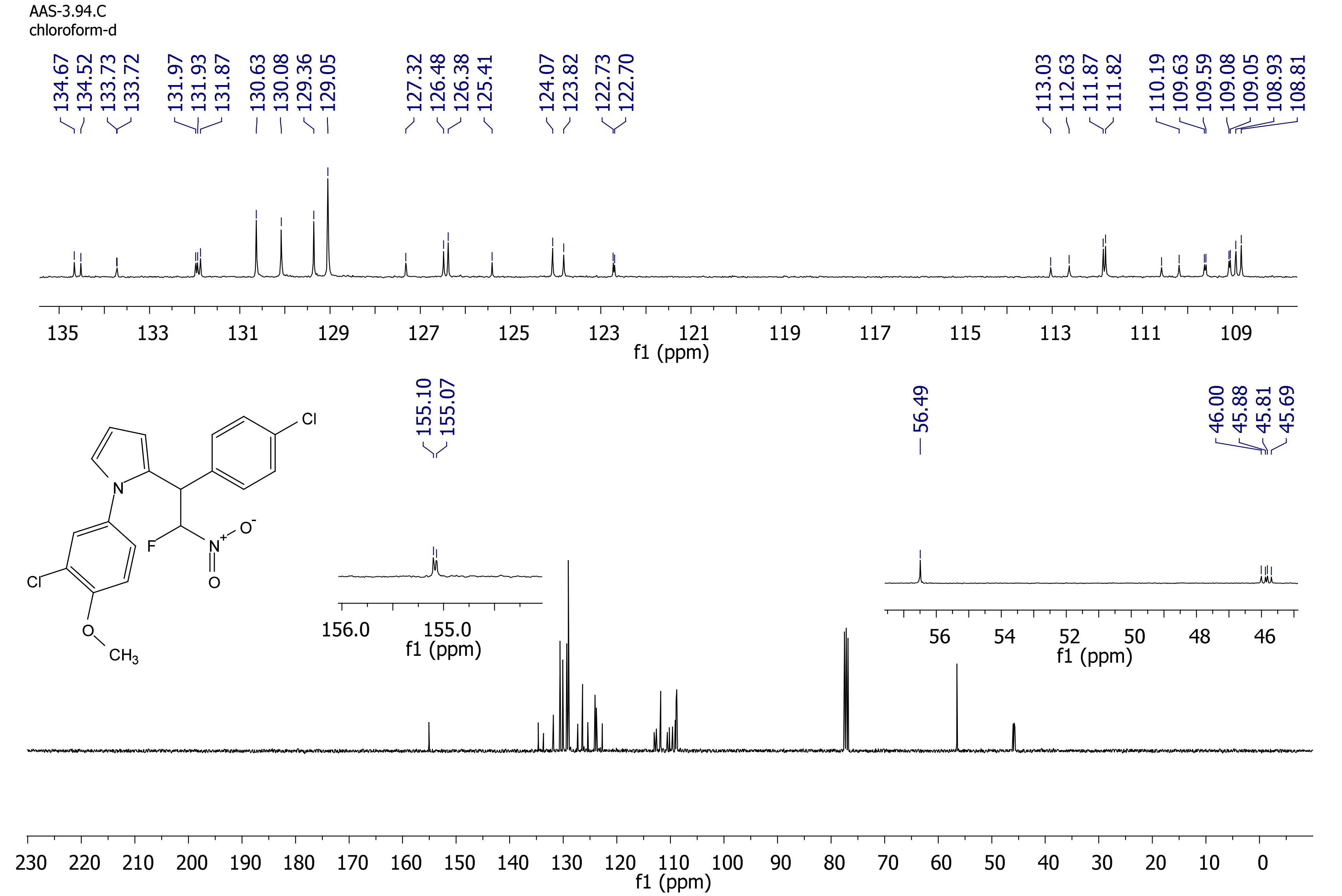

^{13}C NMR spectrum of 1-(3-chloro-4-methoxyphenyl)-2-(1-(4-chlorophenyl)-2-fluoro-2-nitroethyl)-1*H*-pyrrole (**3t**)

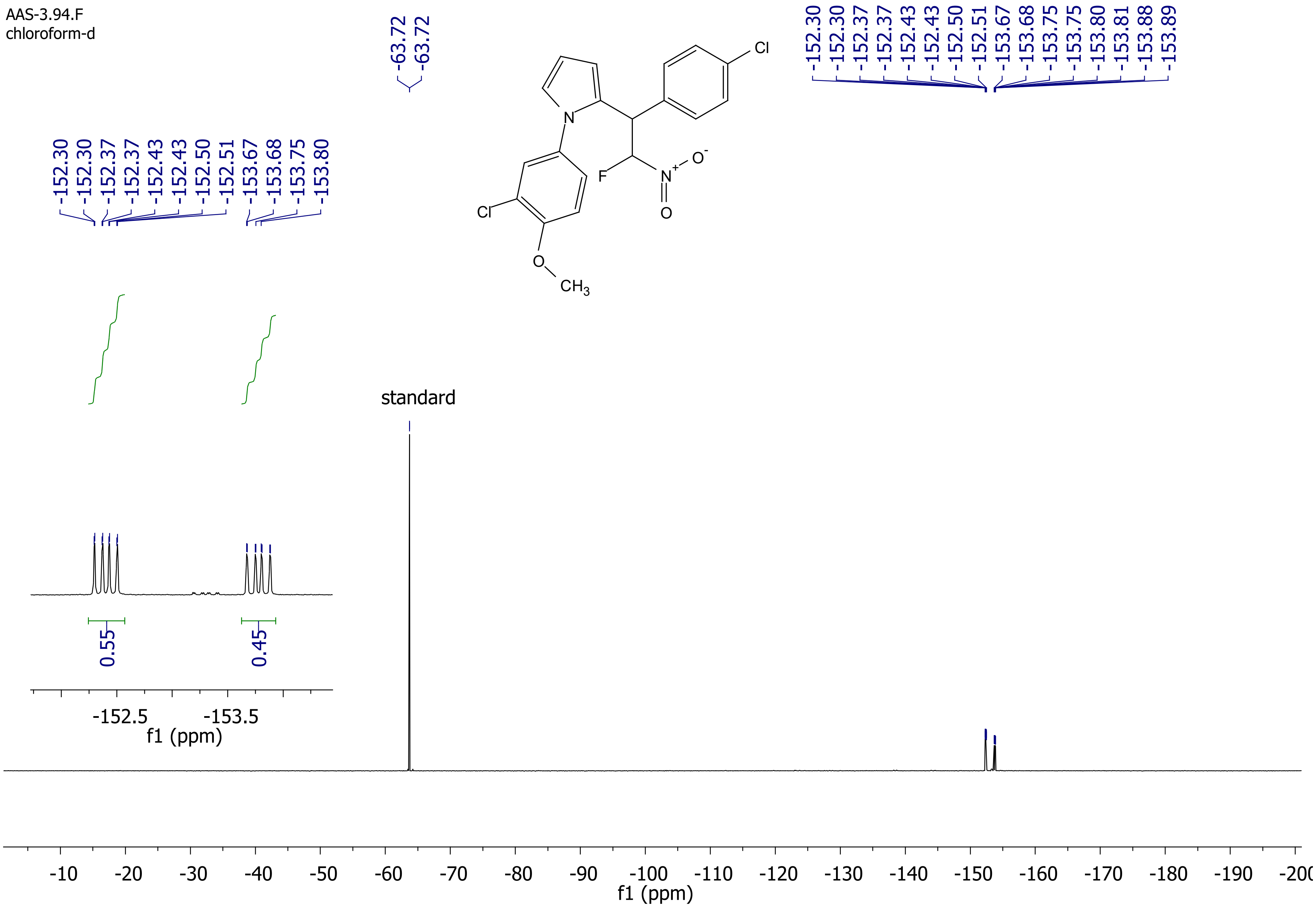

^{19}F NMR spectrum of 1-(3-chloro-4-methoxyphenyl)-2-(1-(4-chlorophenyl)-2-fluoro-2-nitroethyl)-1*H*-pyrrole (**3t**)

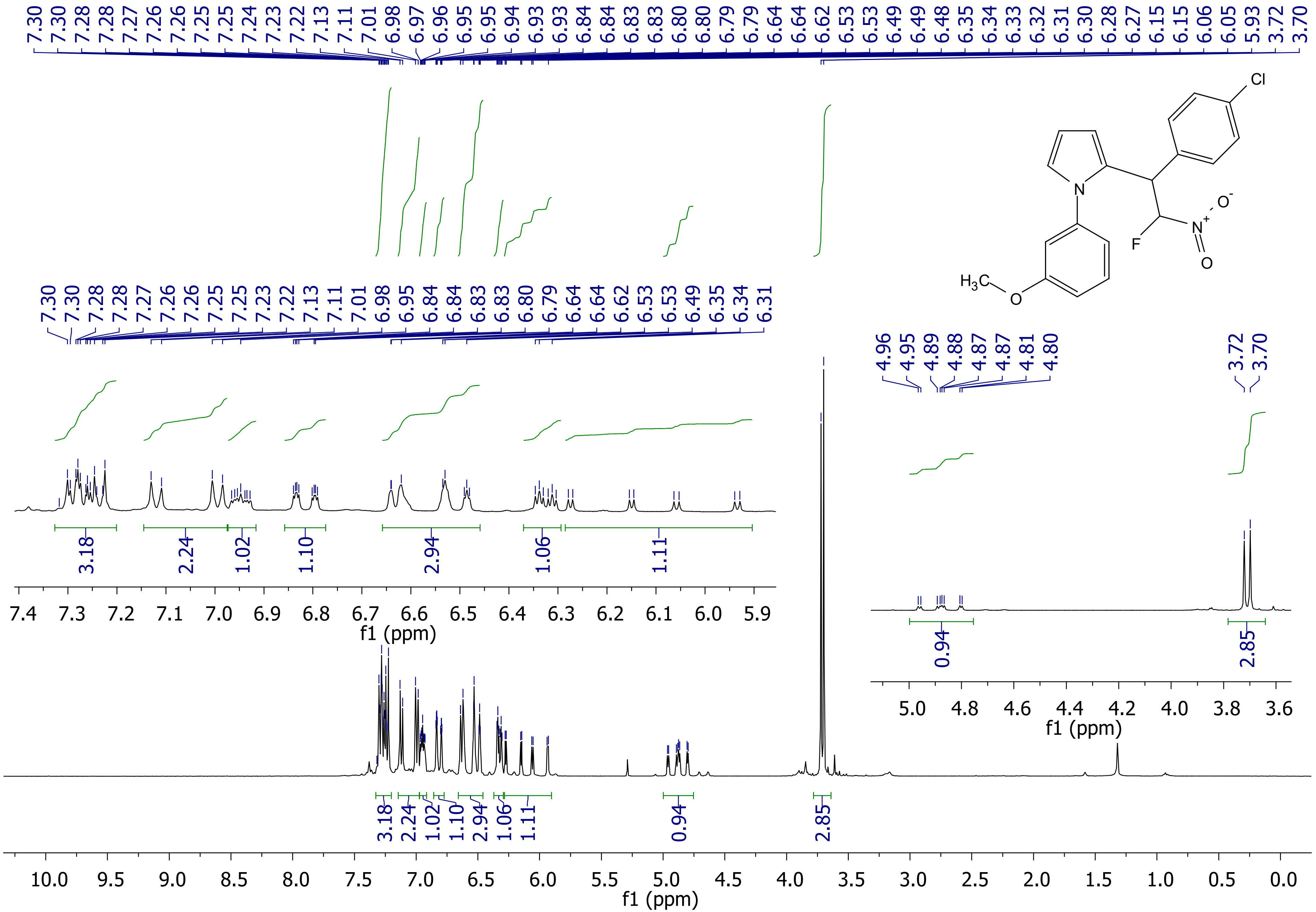

^{1}H NMR spectrum of 2-(1-(4-chlorophenyl)-2-fluoro-2-nitroethyl)-1-(3-methoxyphenyl)-1*H*-pyrrole (**3u**)

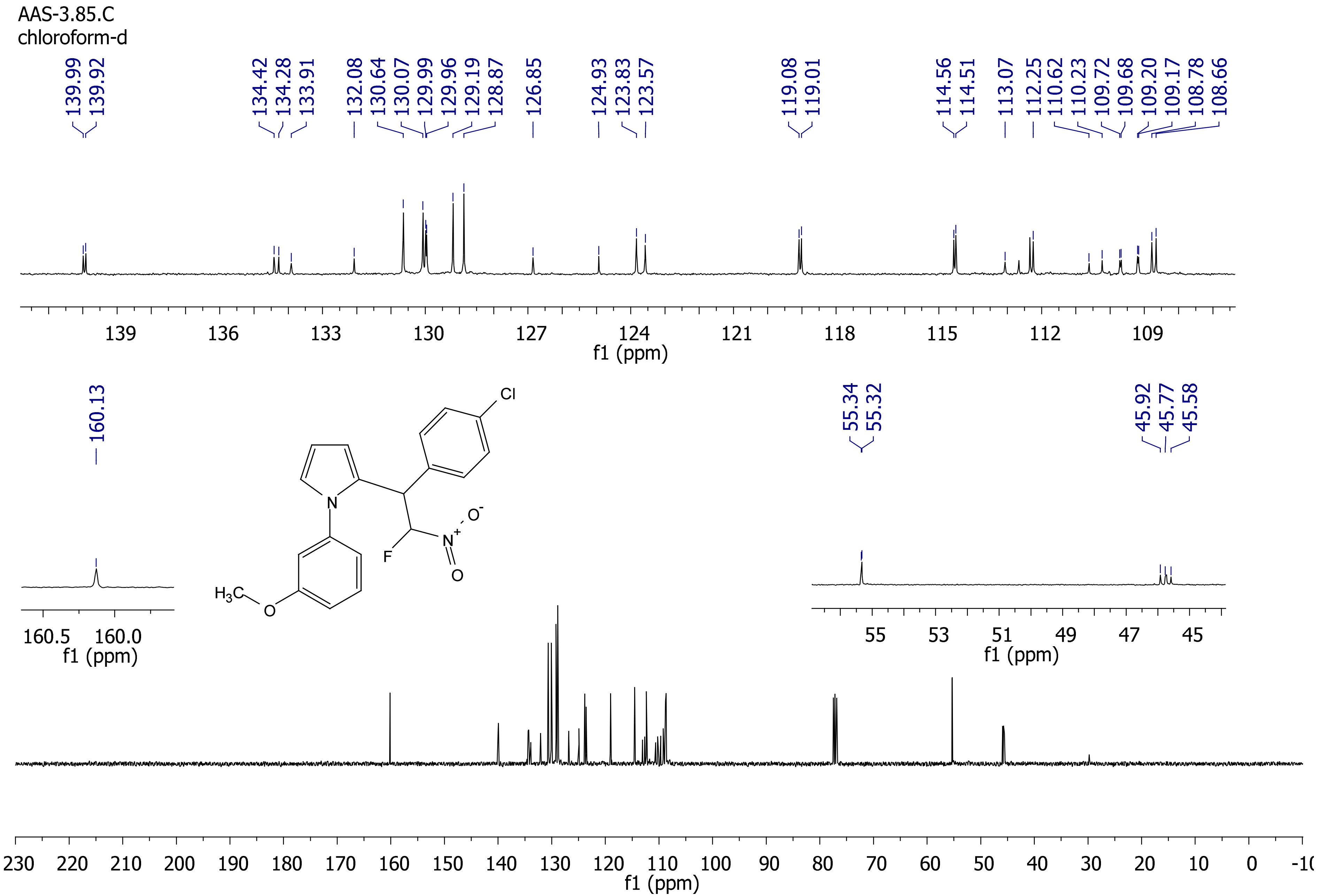

^{13}C NMR spectrum of 2-(1-(4-chlorophenyl)-2-fluoro-2-nitroethyl)-1-(3-methoxyphenyl)-1*H*-pyrrole (**3u**)

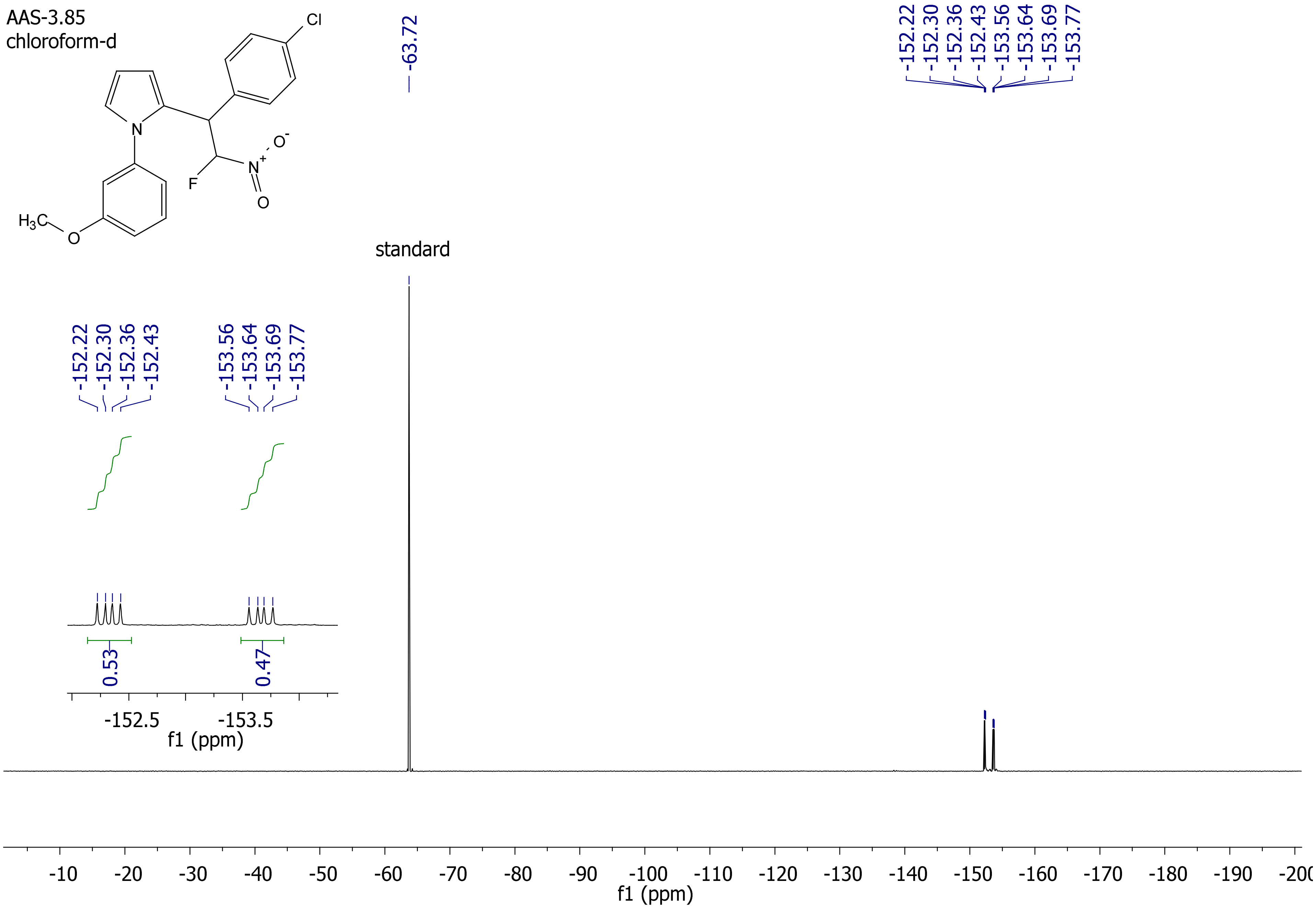

^{19}F NMR spectrum of 2-(1-(4-chlorophenyl)-2-fluoro-2-nitroethyl)-1-(3-methoxyphenyl)-1*H*-pyrrole (**3u**)

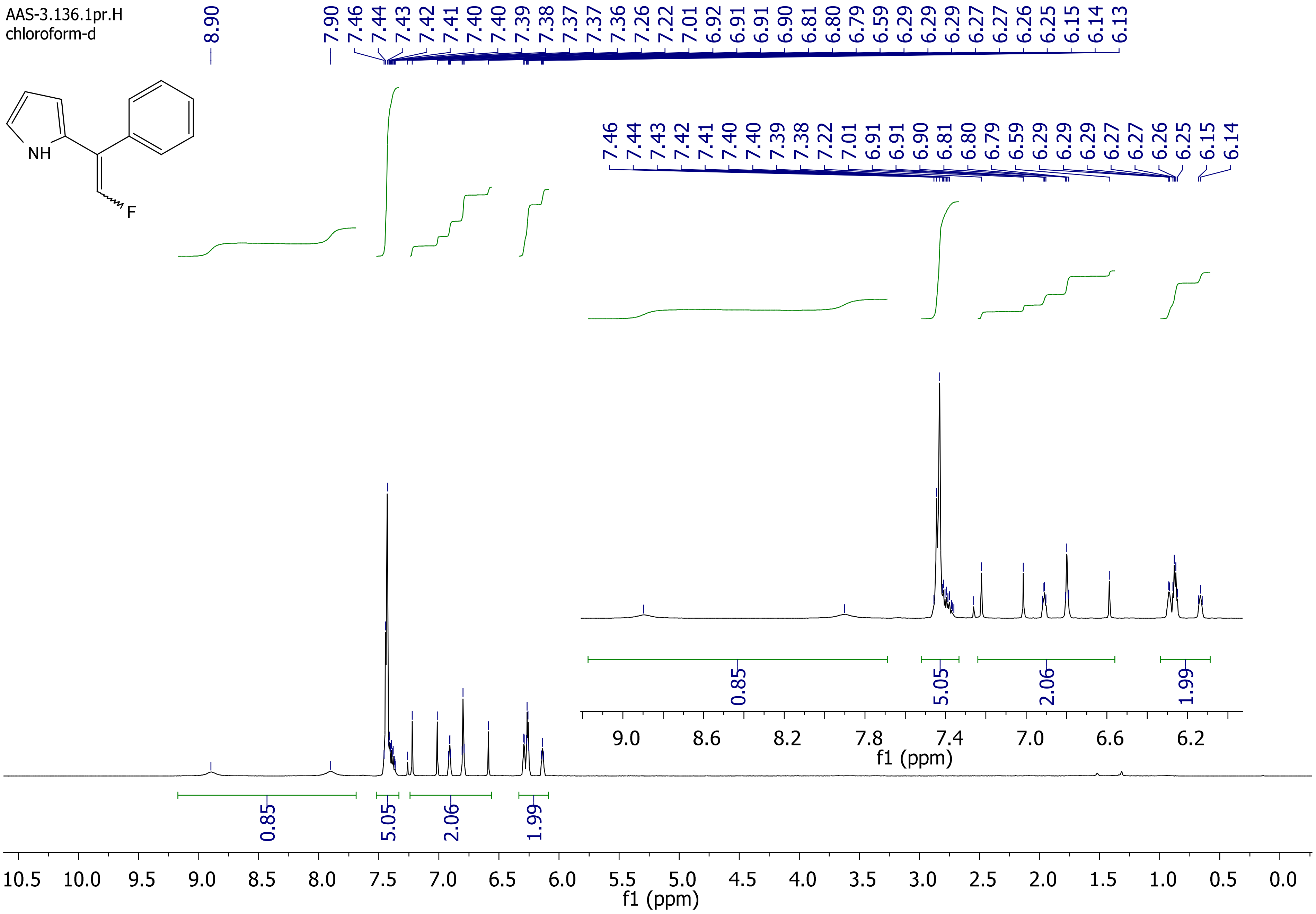

^{1}H NMR spectrum of 2-(2-fluoro-1-phenylvinyl)-1*H*-pyrrole (**4a**)

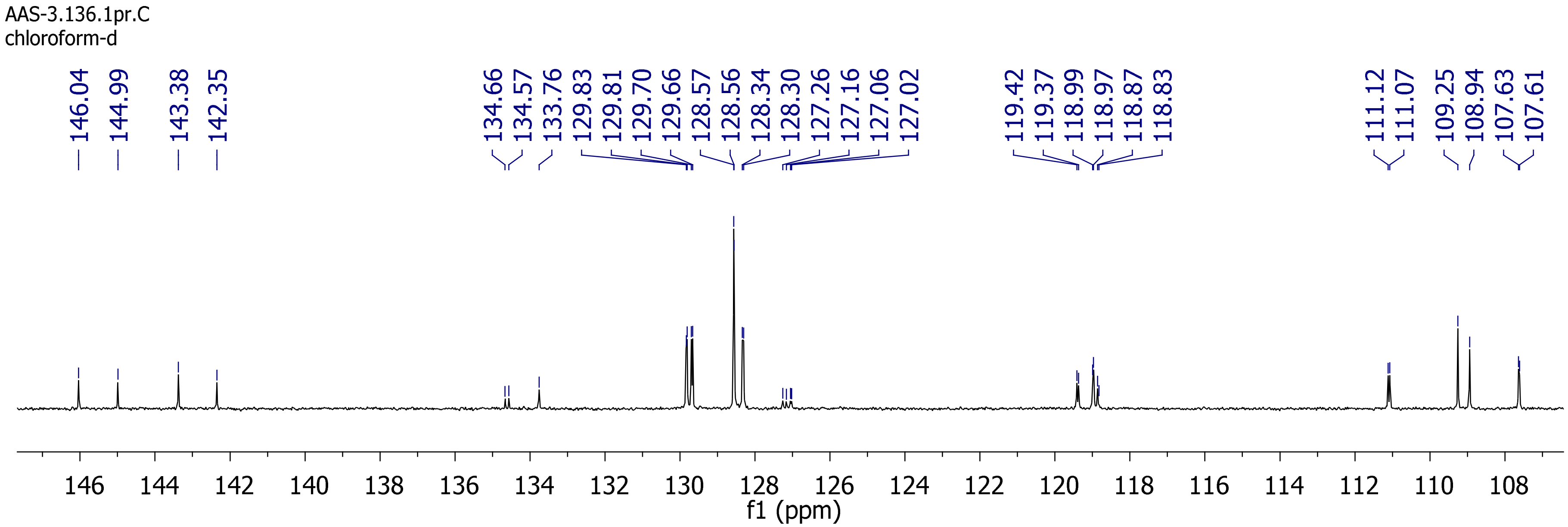

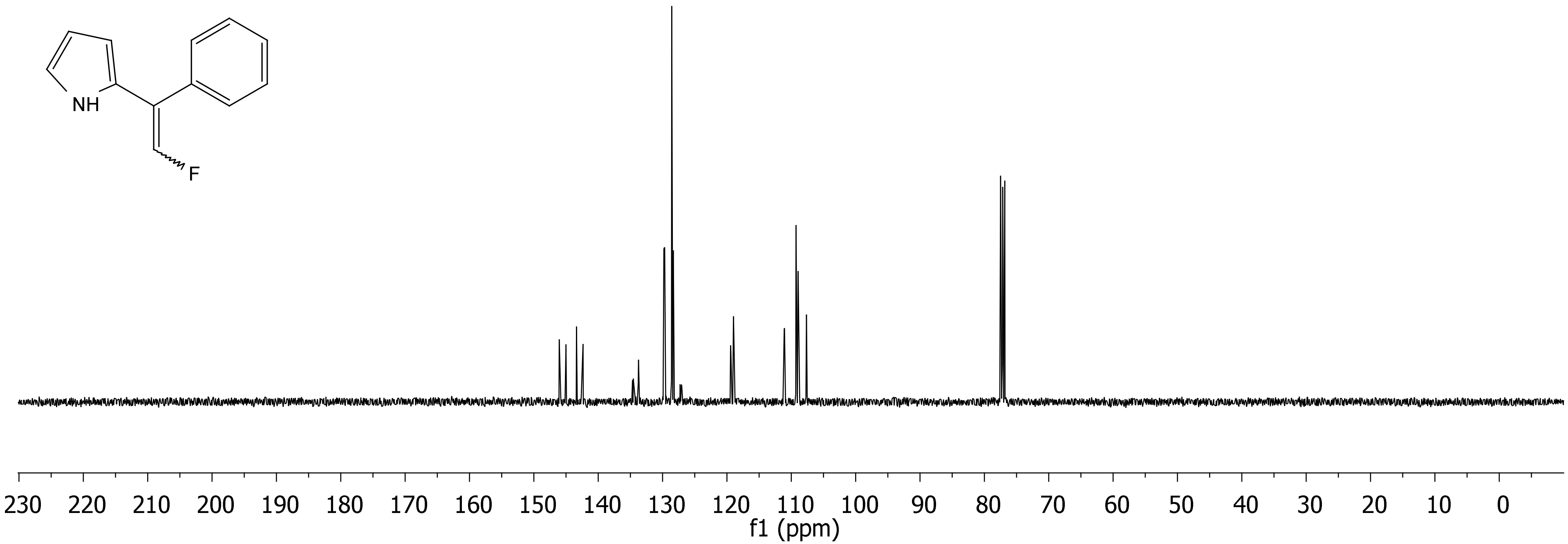

^{13}C NMR spectrum of 2-(2-fluoro-1-phenylvinyl)-1*H*-pyrrole (**4a**)

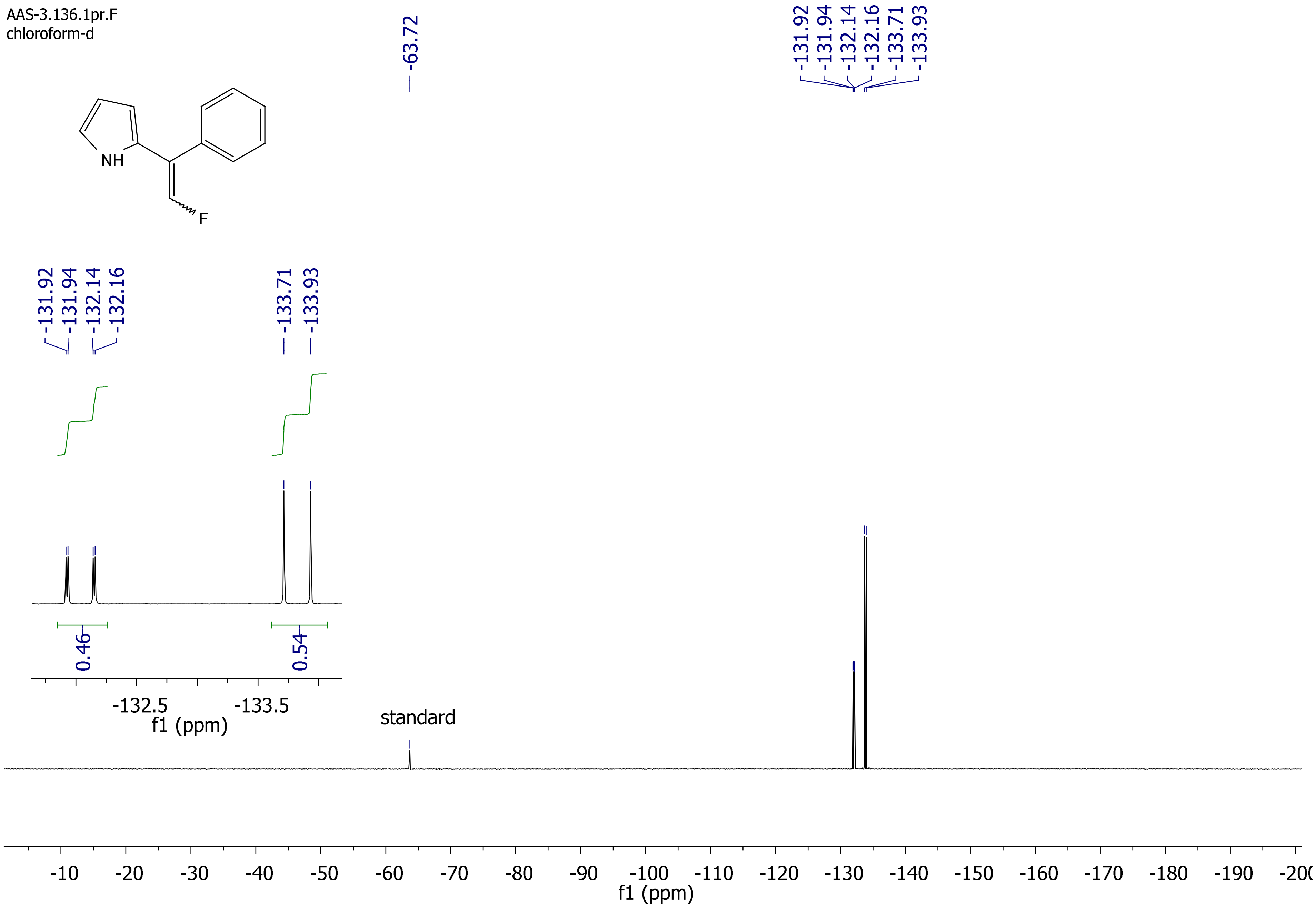

^{19}F NMR spectrum of 2-(2-fluoro-1-phenylvinyl)-1*H*-pyrrole (**4a**)

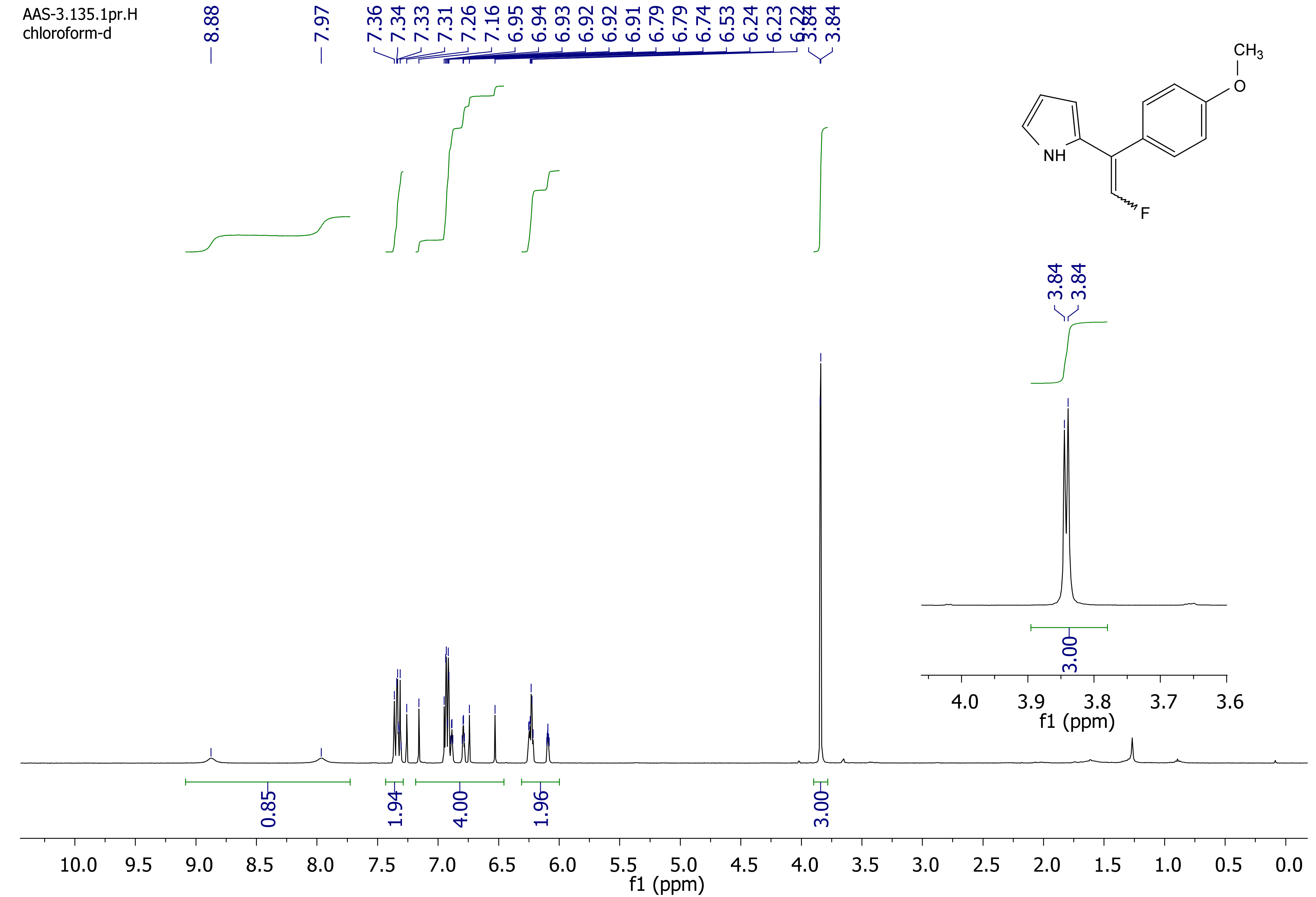

^{1}H NMR spectrum of 2-(2-fluoro-1-(4-methoxyphenyl)vinyl)-1*H*-pyrrole (**4b**)

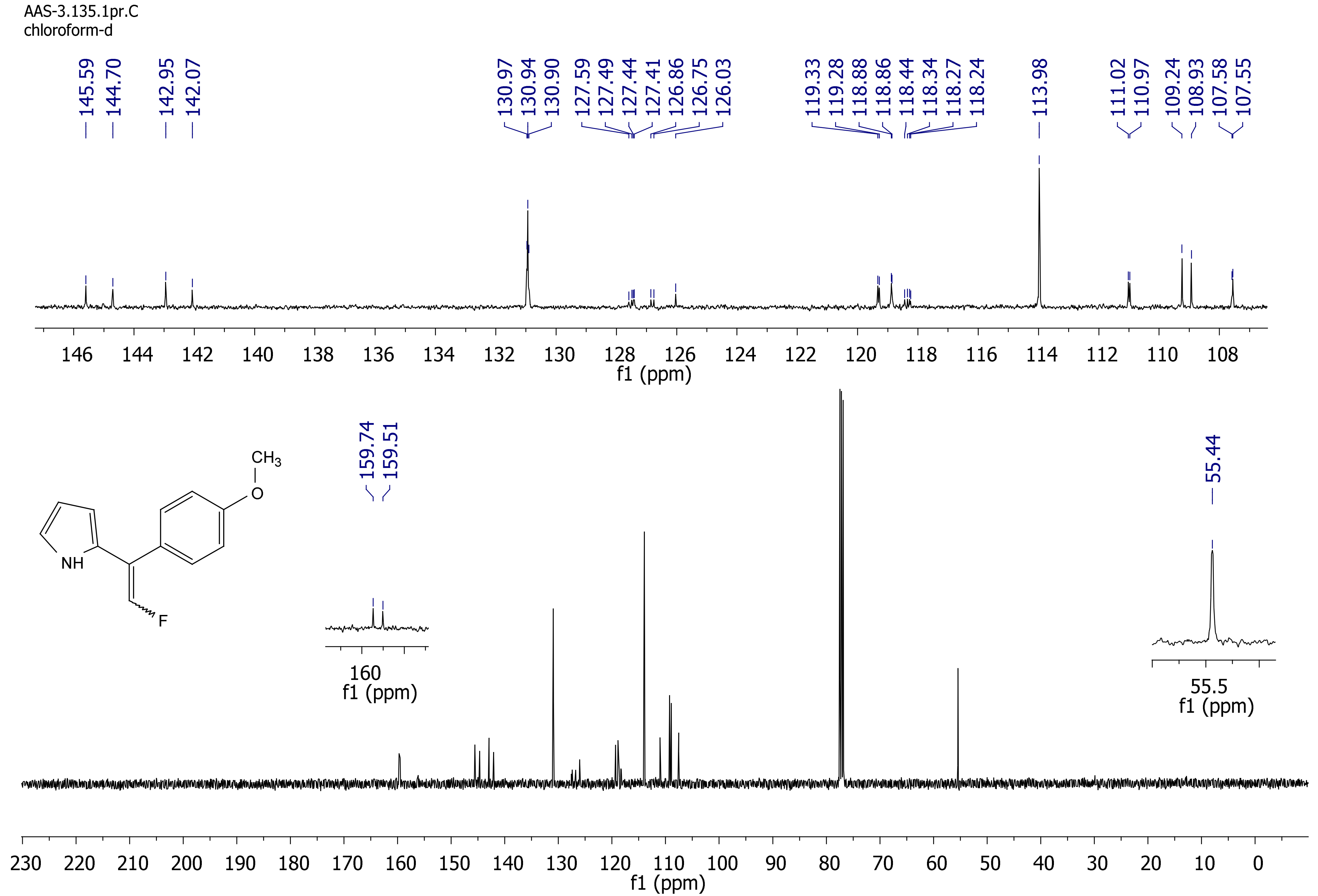

^{13}C NMR spectrum of 2-(2-fluoro-1-(4-methoxyphenyl)vinyl)-1*H*-pyrrole (**4b**)

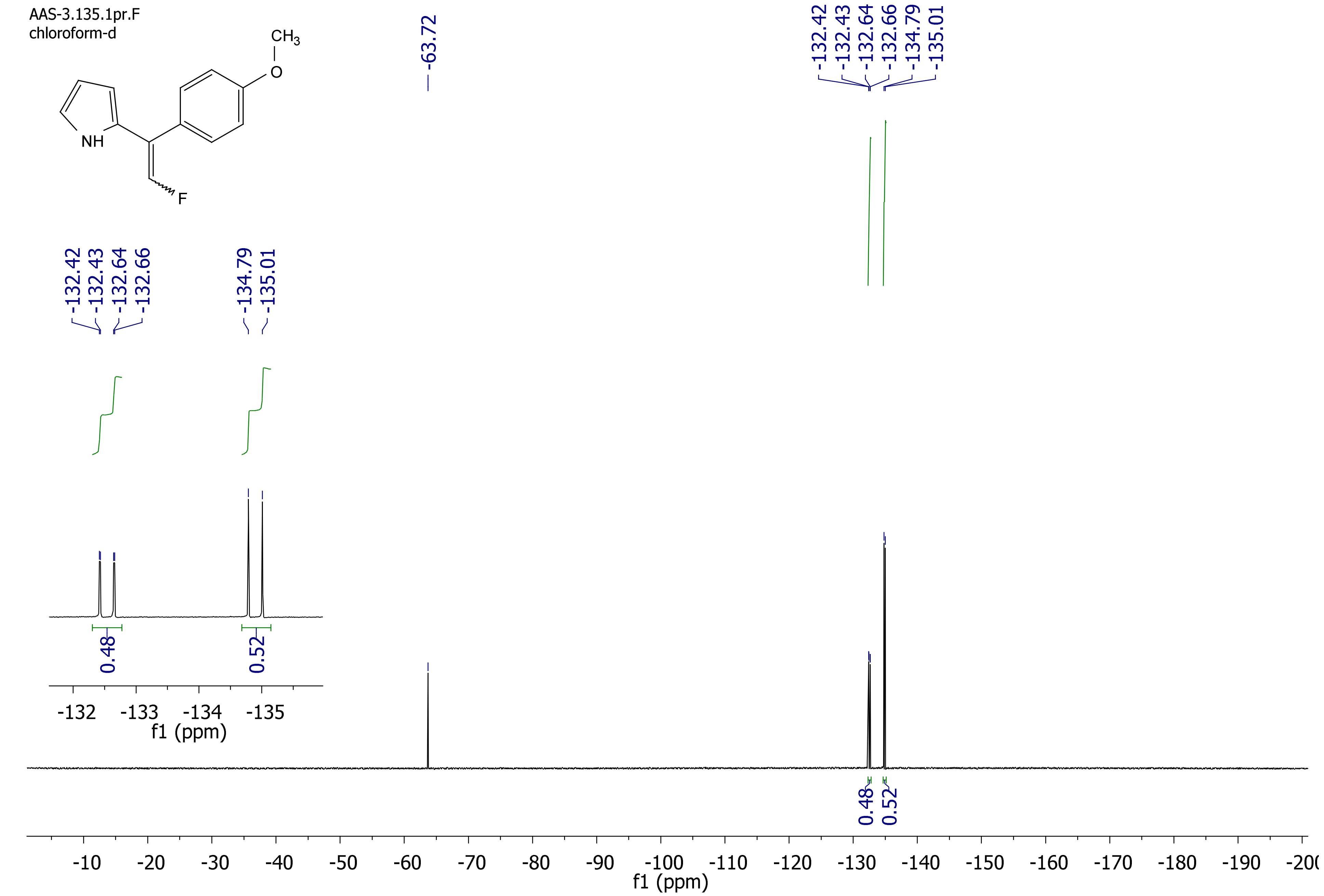

^{19}F NMR spectrum of 2-(2-fluoro-1-(4-methoxyphenyl)vinyl)-1*H*-pyrrole (**4b**)

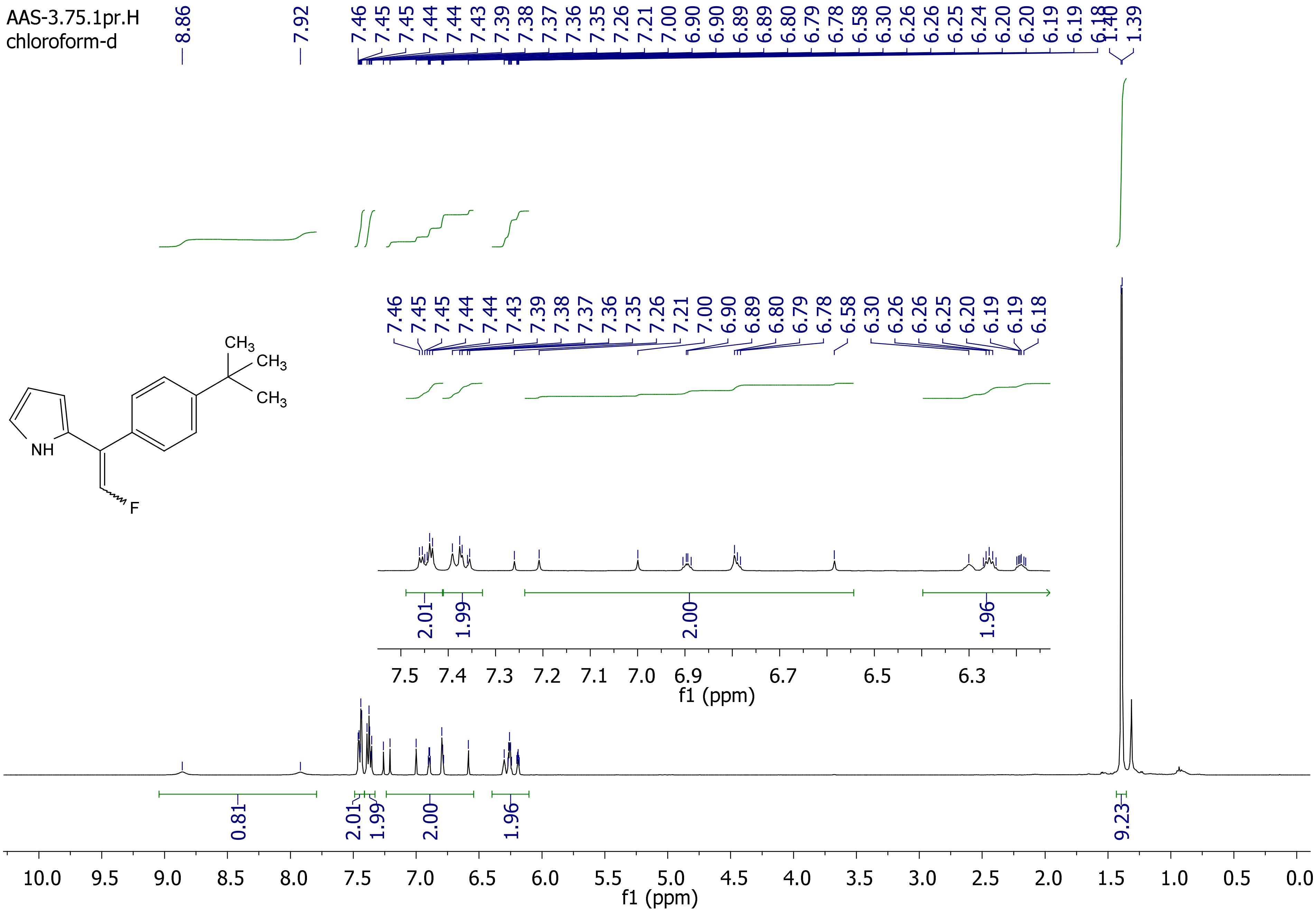

^{1}H NMR spectrum of 2-(1-(4-(*tert*-butyl)phenyl)-2-fluorovinyl)-1*H*-pyrrole (**4c**)

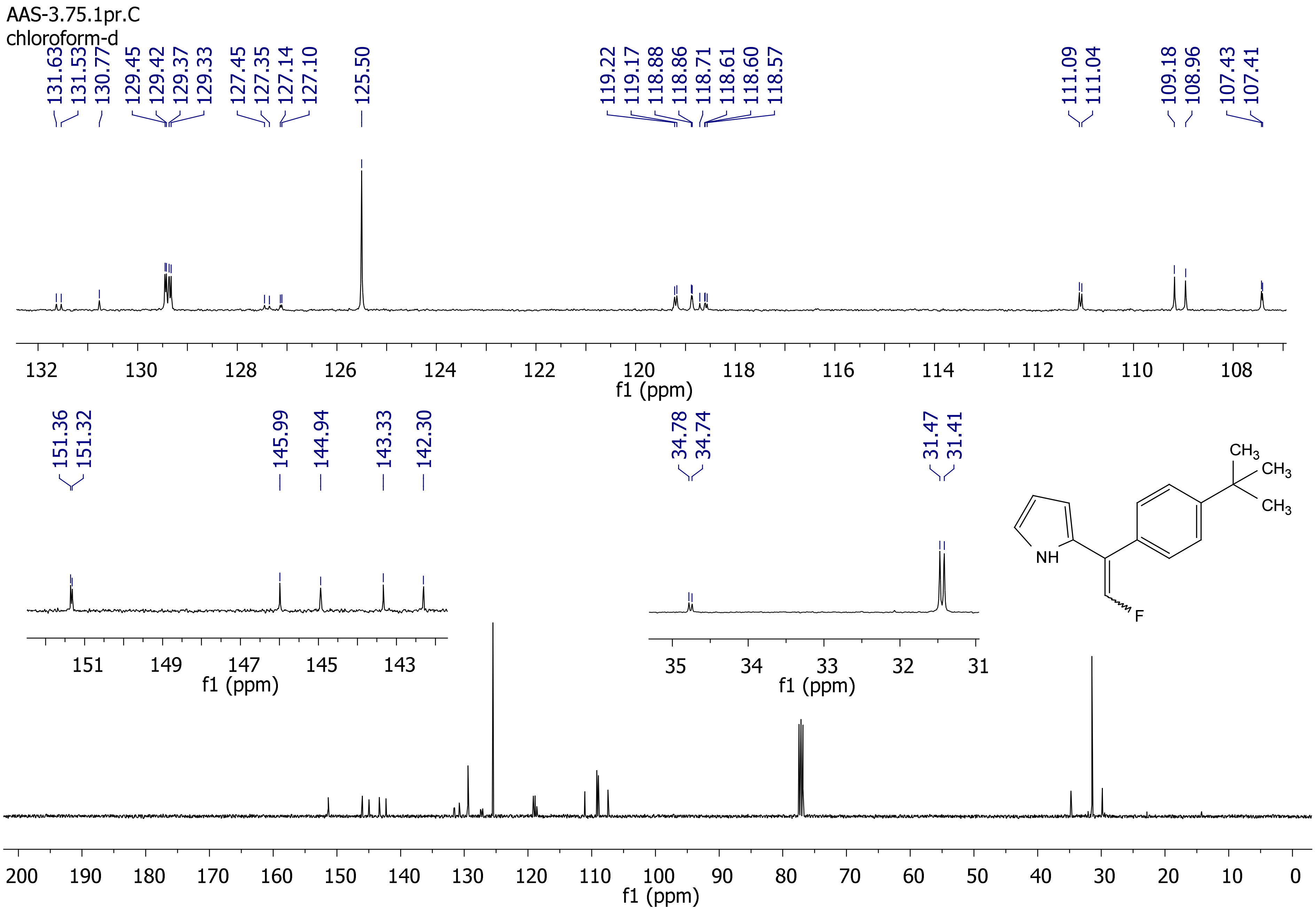

^{13}C NMR spectrum of 2-(1-(4-(*tert*-butyl)phenyl)-2-fluorovinyl)-1*H*-pyrrole (**4c**)

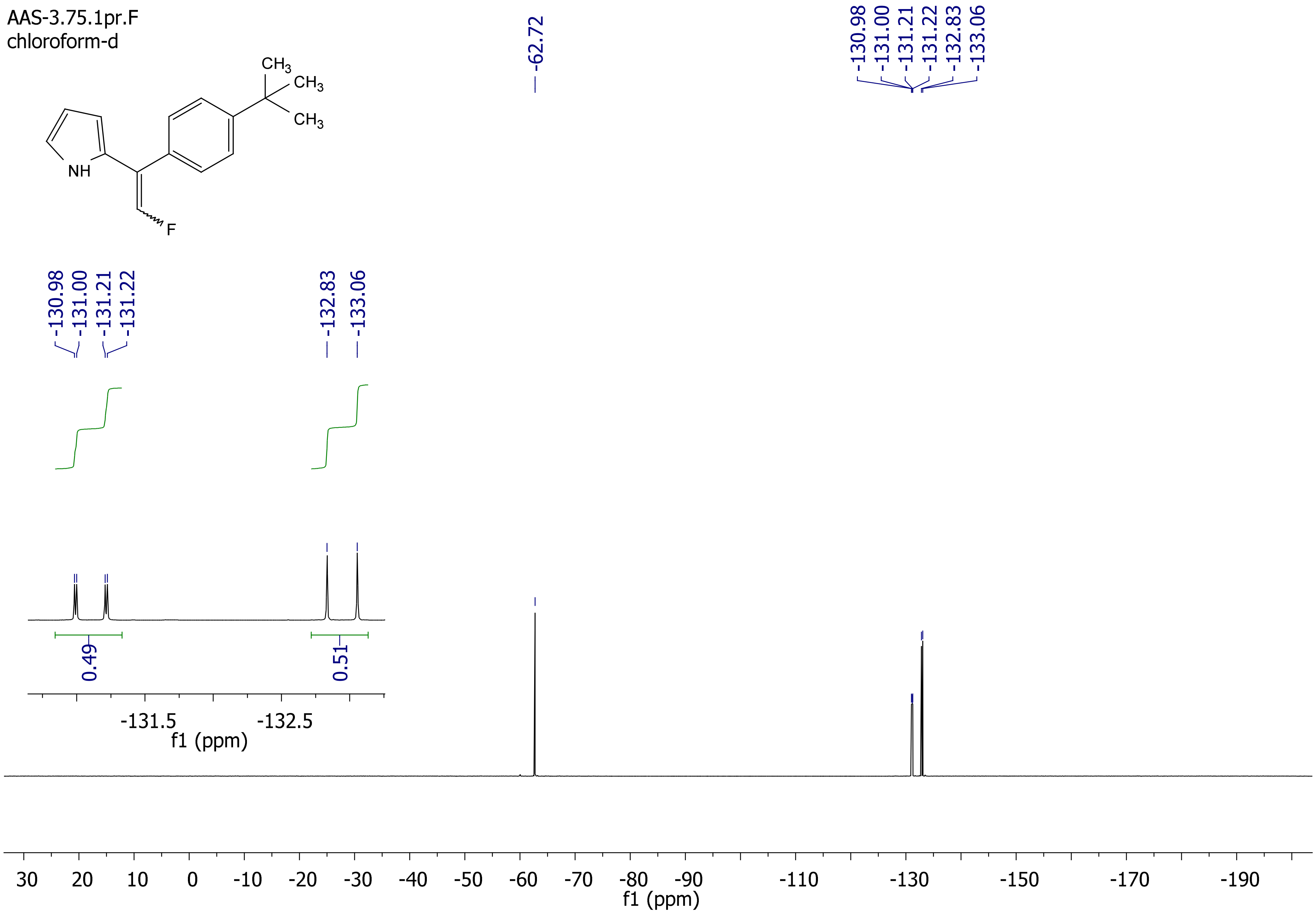

^{19}F NMR spectrum of 2-(1-(4-(tert-butyl)phenyl)-2-fluorovinyl)-1*H*-pyrrole (**4c**)

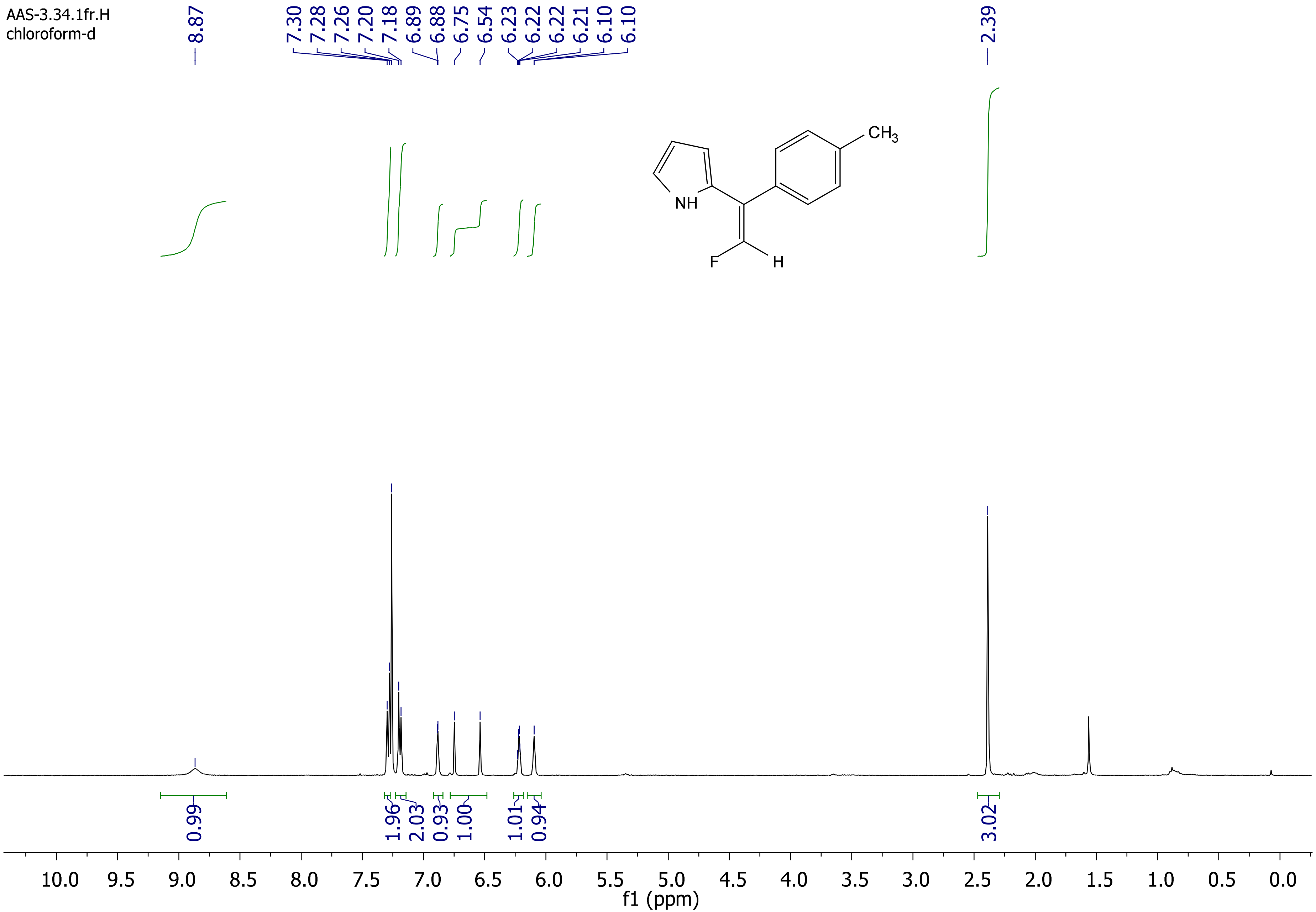

^{1}H NMR spectrum of (*Z*)-2-(2-fluoro-1-(p-tolyl)vinyl)-1*H*-pyrrole (*Z*-**4d**)

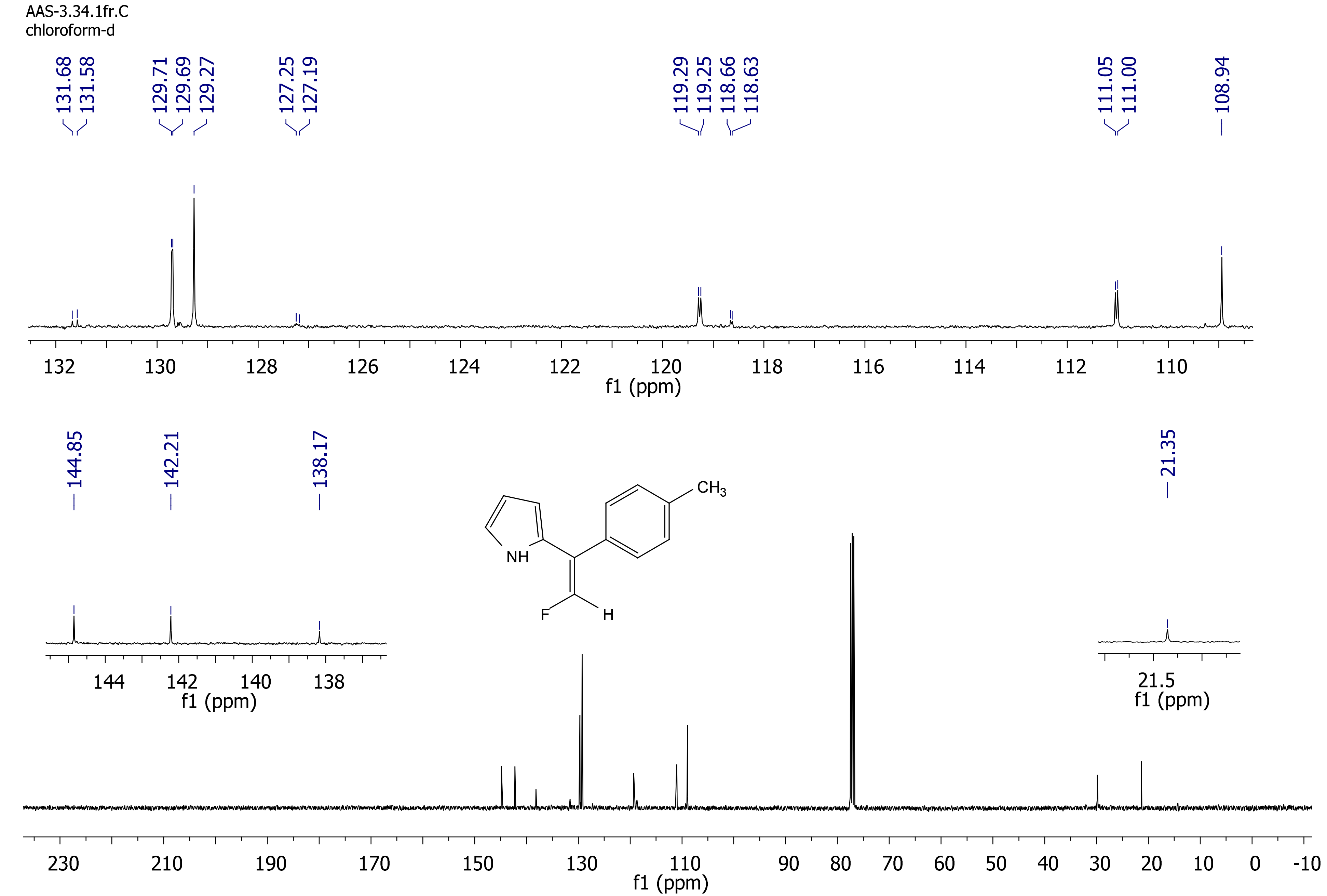

^{13}C NMR spectrum of (*Z*)-2-(2-fluoro-1-(p-tolyl)vinyl)-1*H*-pyrrole (*Z*-**4d**)

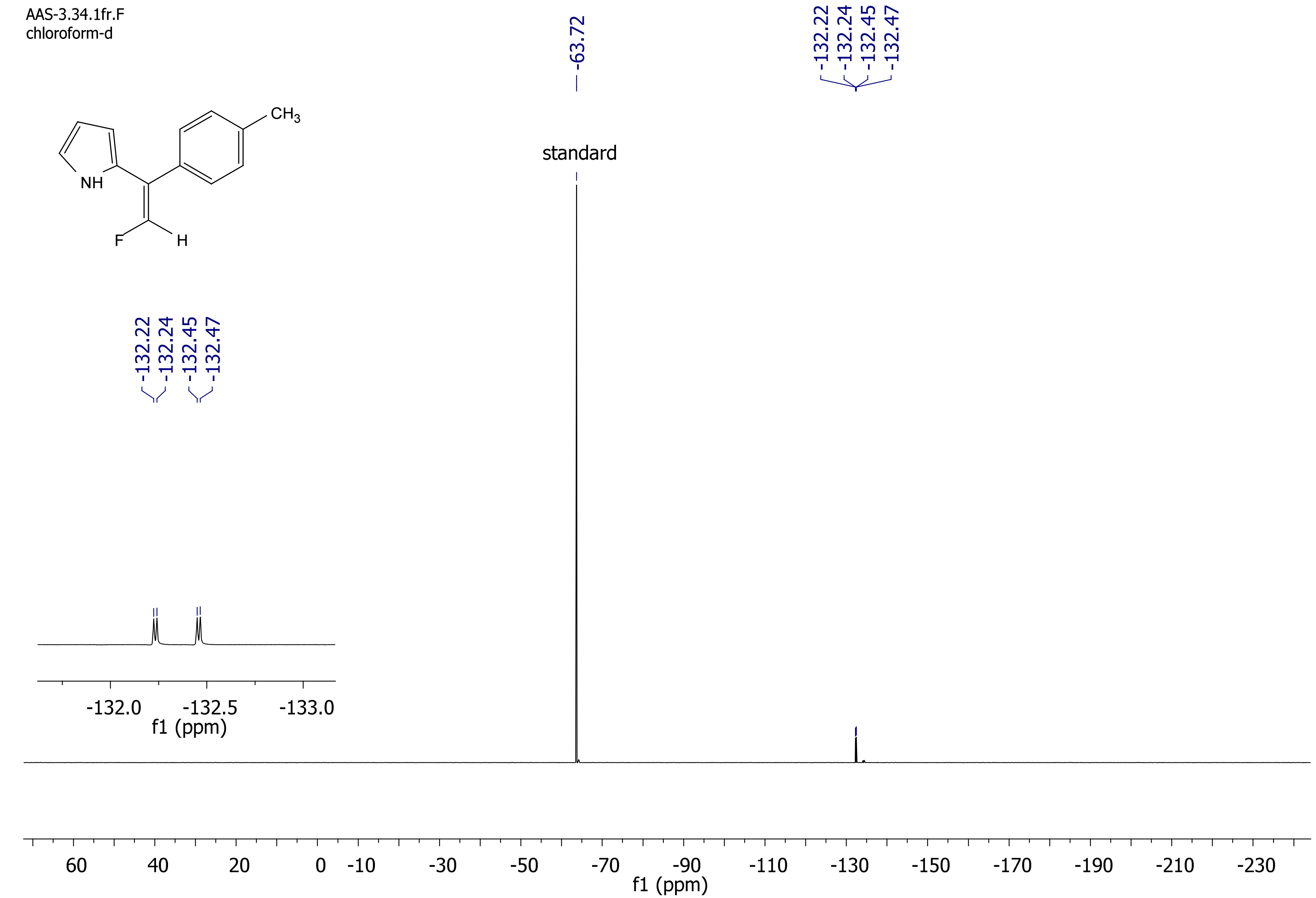

^{19}F NMR spectrum of (*Z*)-2-(2-fluoro-1-(p-tolyl)vinyl)-1*H*-pyrrole (*Z*-**4d**)

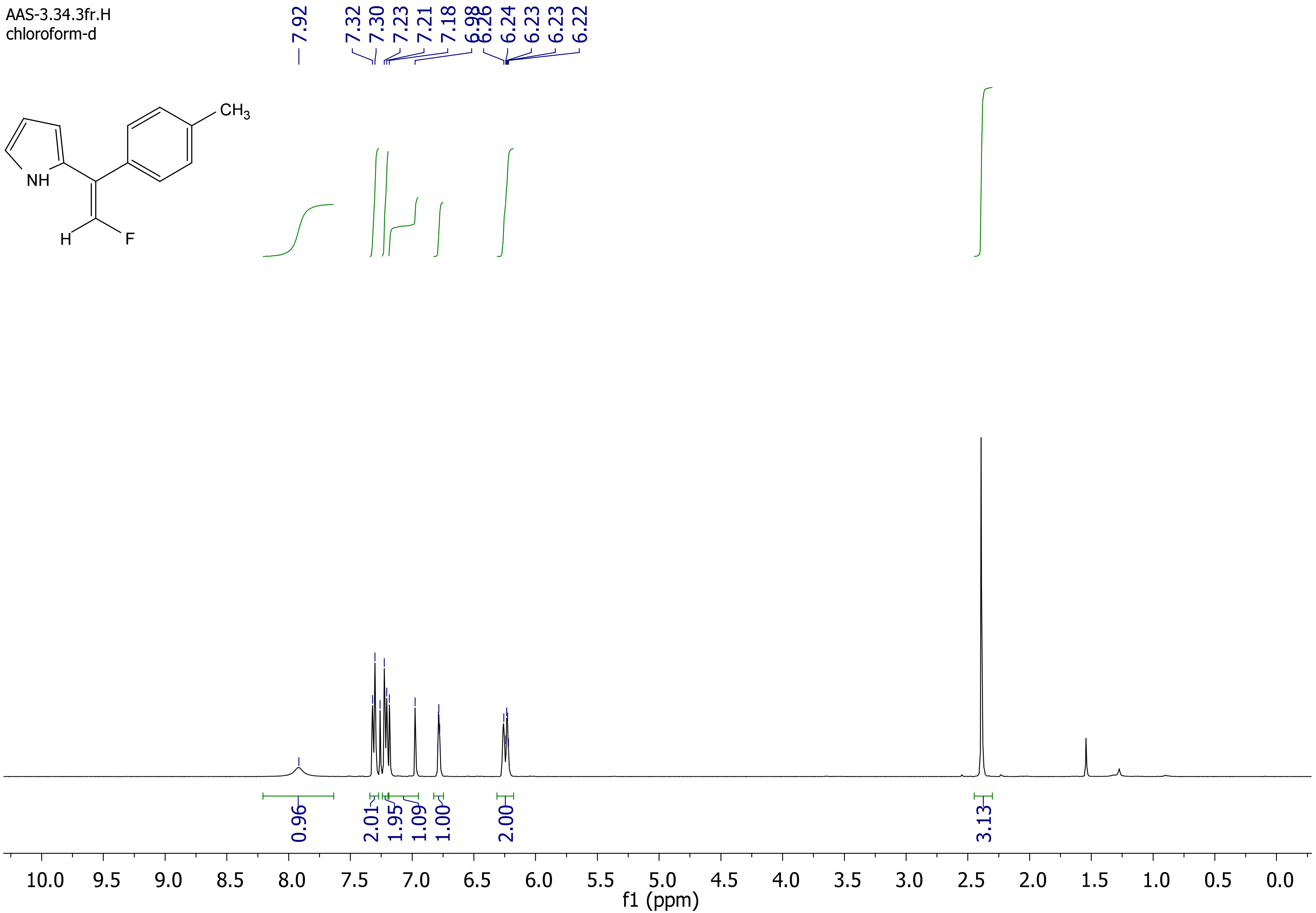

^{1}H NMR spectrum of (*E*)-2-(2-fluoro-1-(p-tolyl)vinyl)-1*H*-pyrrole (*E*-**4d**)

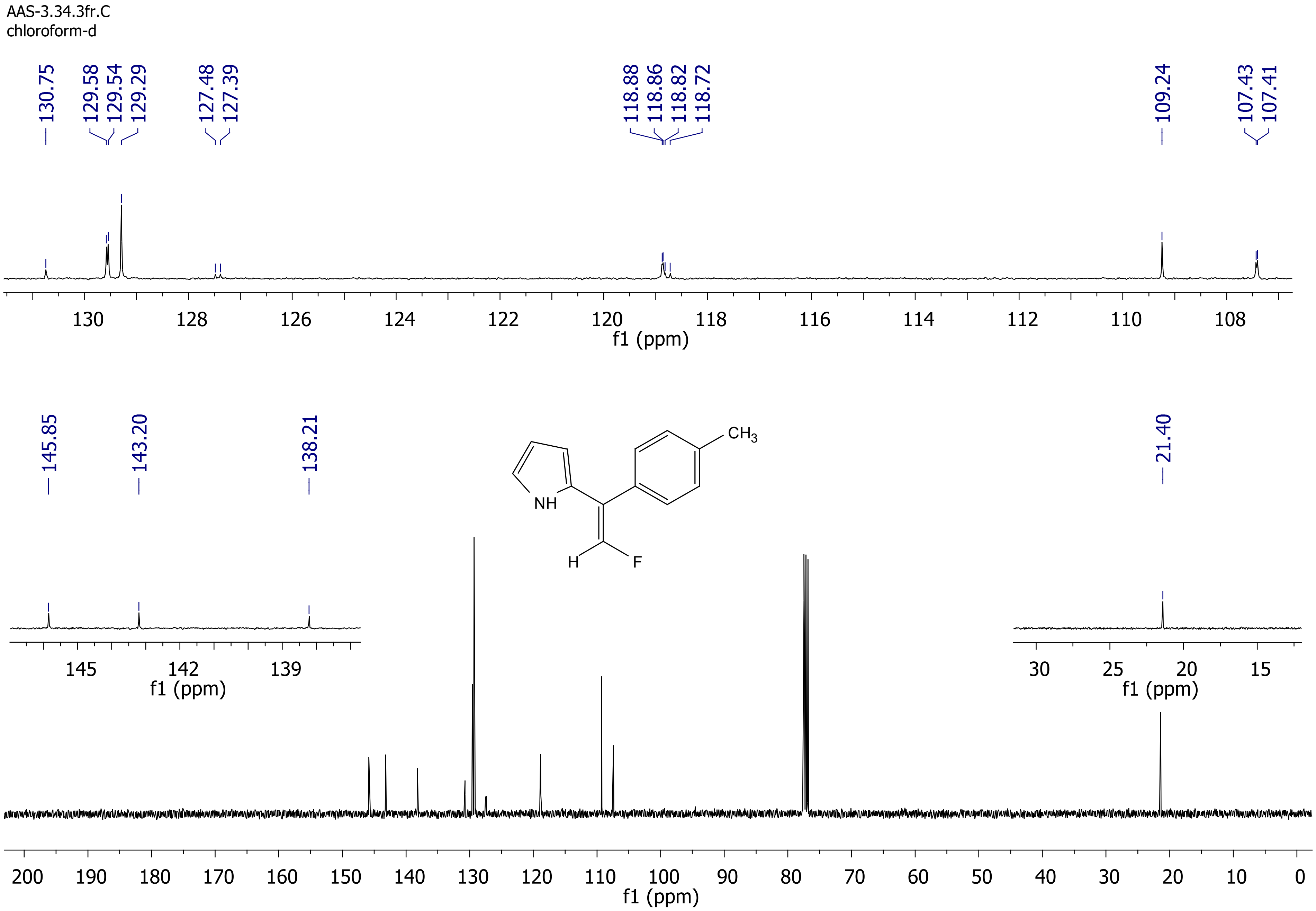

^{13}C NMR spectrum of spectrum of (*E*)-2-(2-fluoro-1-(p-tolyl)vinyl)-1*H*-pyrrole (*E*-**4d**)

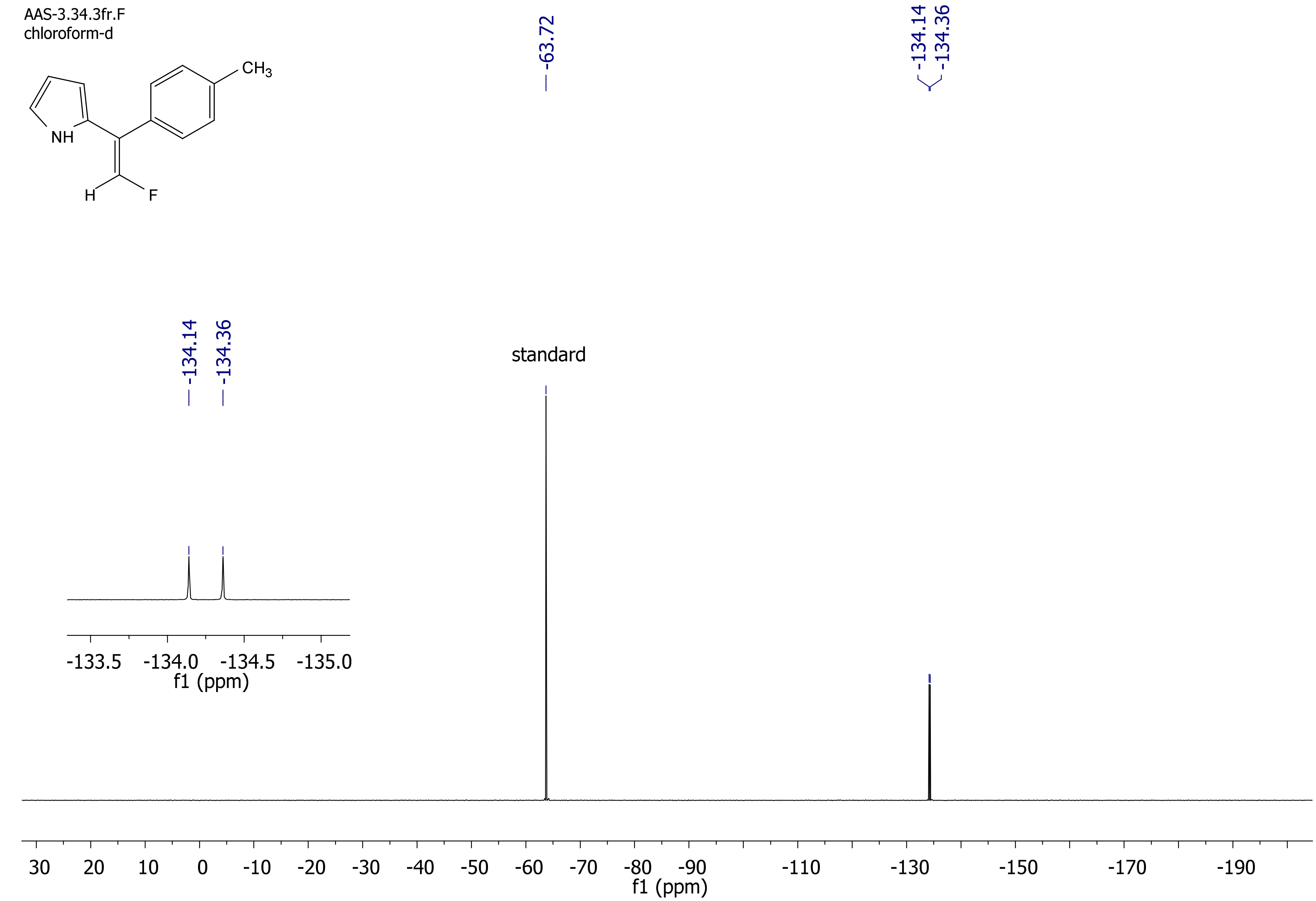

^{19}F NMR spectrum of (*E*)-2-(2-fluoro-1-(p-tolyl)vinyl)-*1H*-pyrrole (*E*-**4d**)

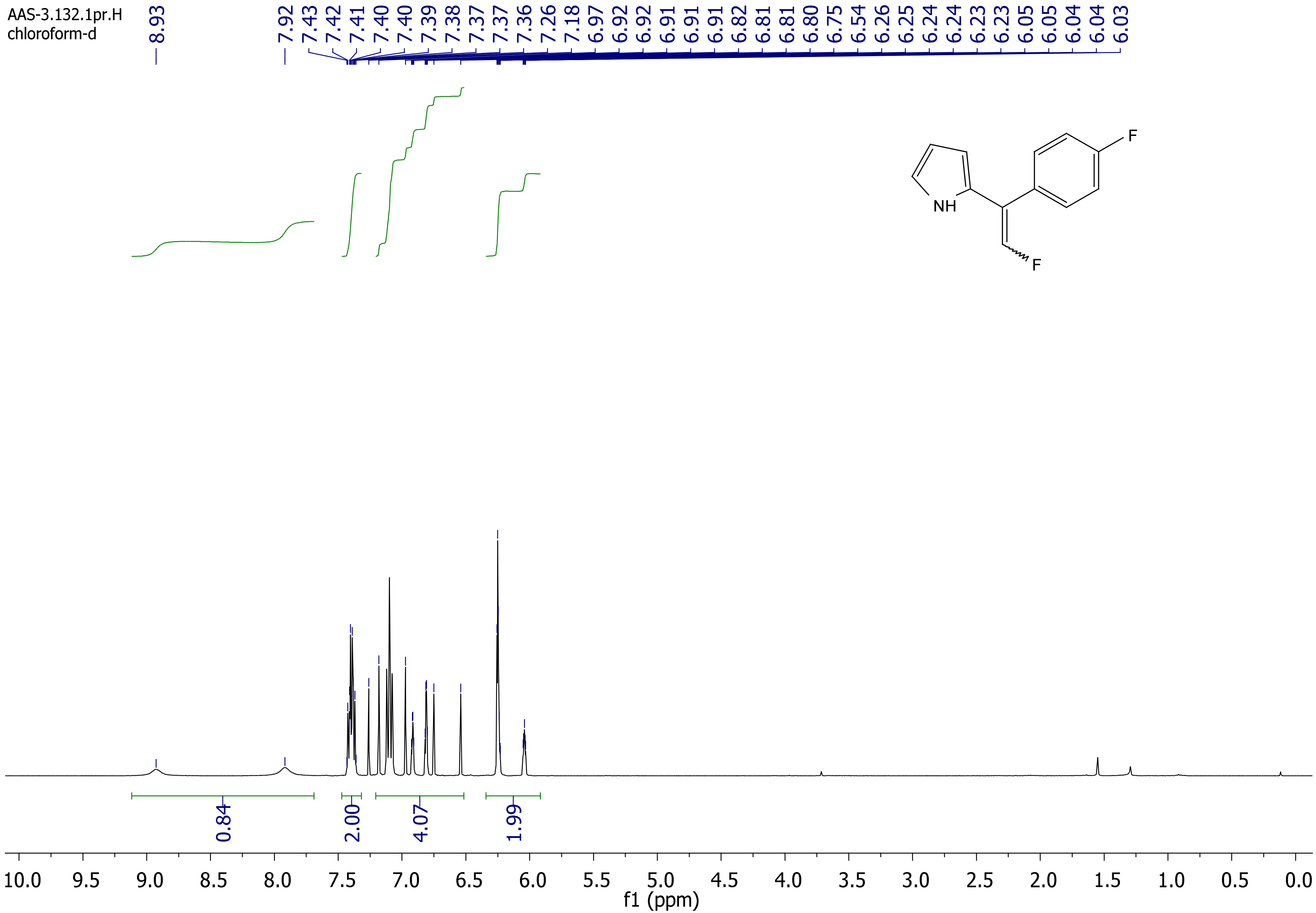

^{1}H NMR spectrum of 2-(2-fluoro-1-(4-fluorophenyl)vinyl)-1*H*-pyrrole (**4e**)

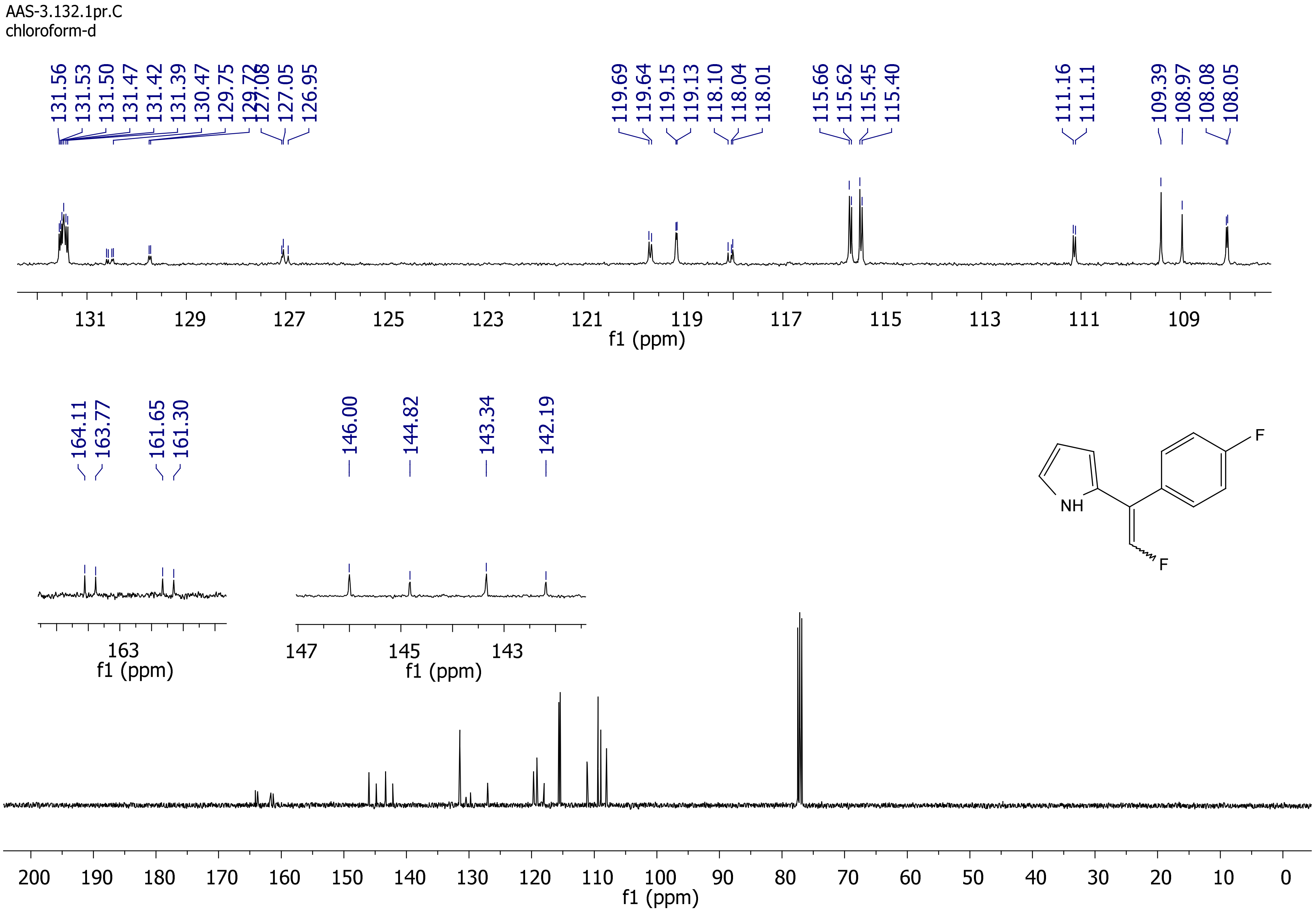

^{13}C NMR spectrum of spectrum of 2-(2-fluoro-1-(4-fluorophenyl)vinyl)-1*H*-pyrrole (**4e**)

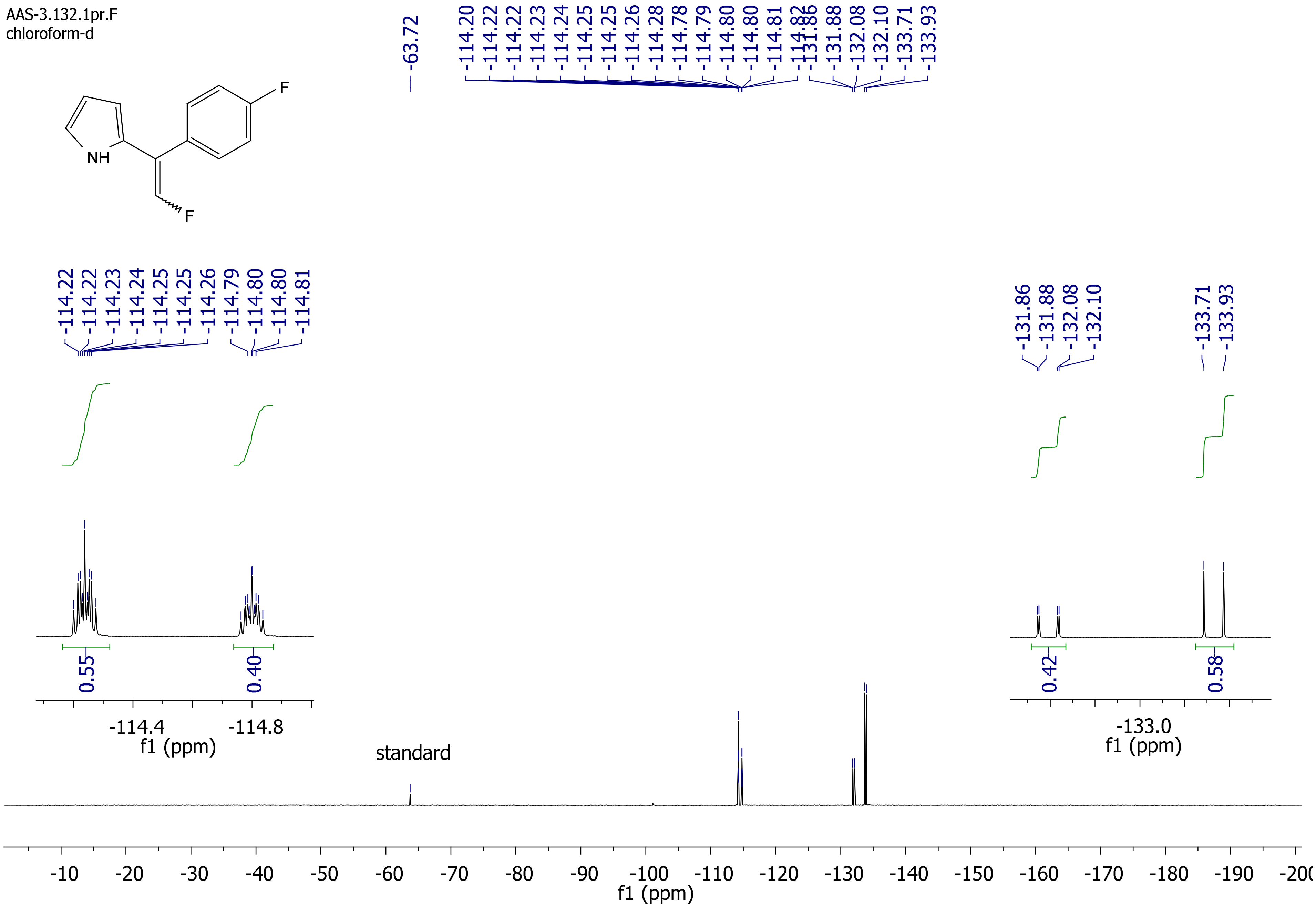

^{19}F NMR spectrum of 2-(2-fluoro-1-(4-fluorophenyl)vinyl)-1*H*-pyrrole (**4e**)

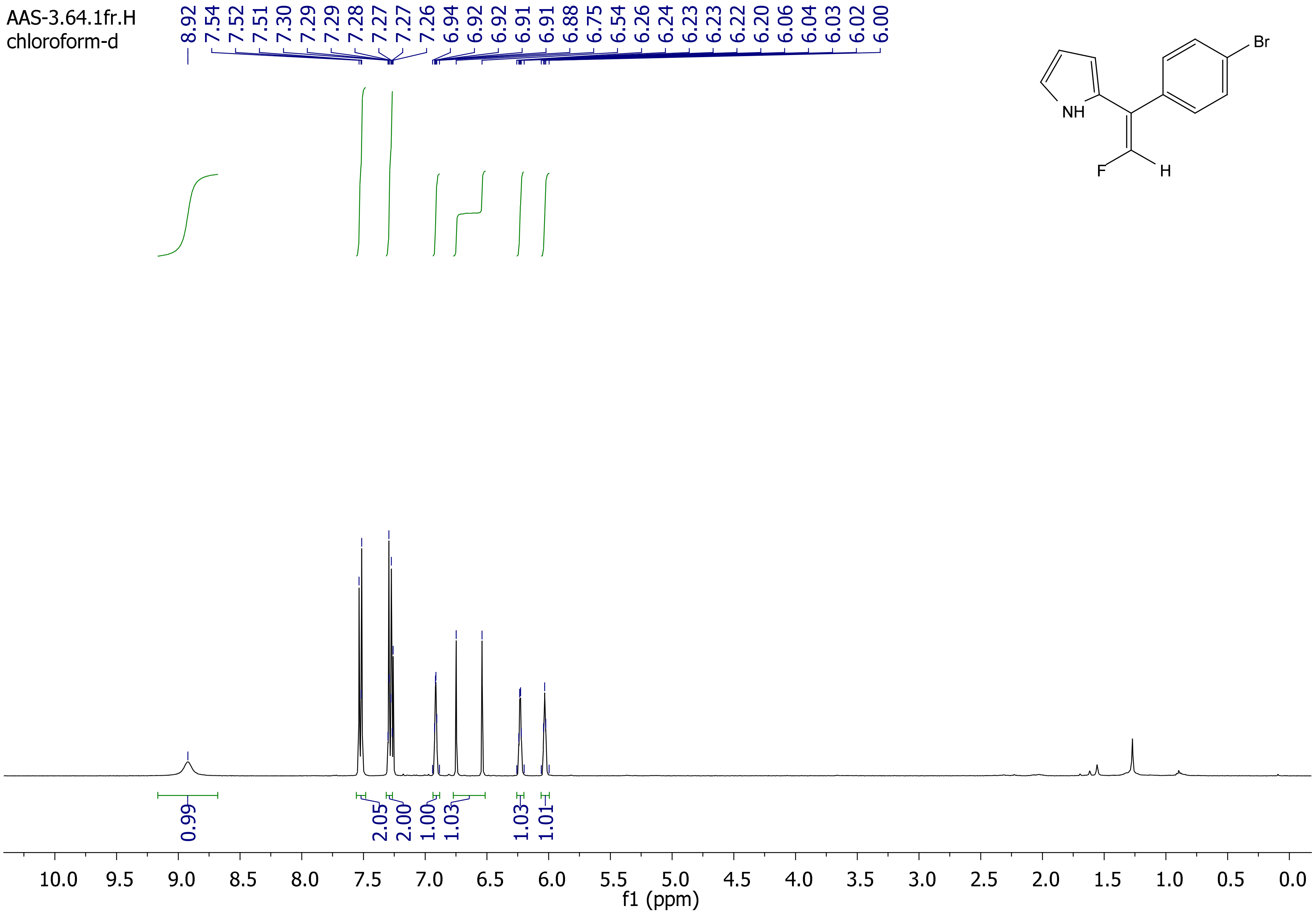

^{1}H NMR spectrum of (*Z*)-2-(1-(4-bromophenyl)-2-fluorovinyl)-1*H*-pyrrole (*Z*-**4f**)

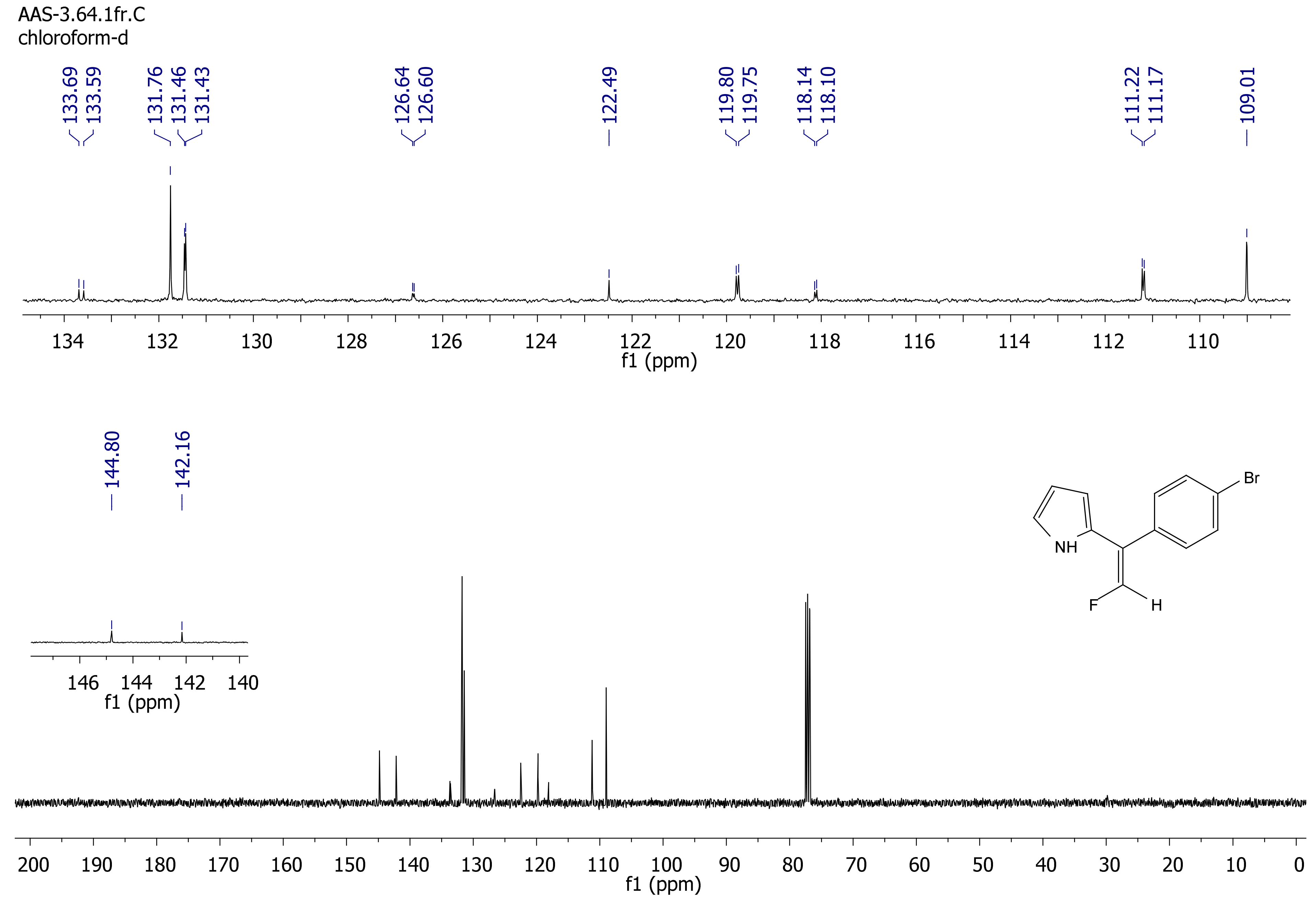

^{13}C NMR spectrum of (*Z*)-2-(1-(4-bromophenyl)-2-fluorovinyl)-1*H*-pyrrole (*Z*-**4f**)

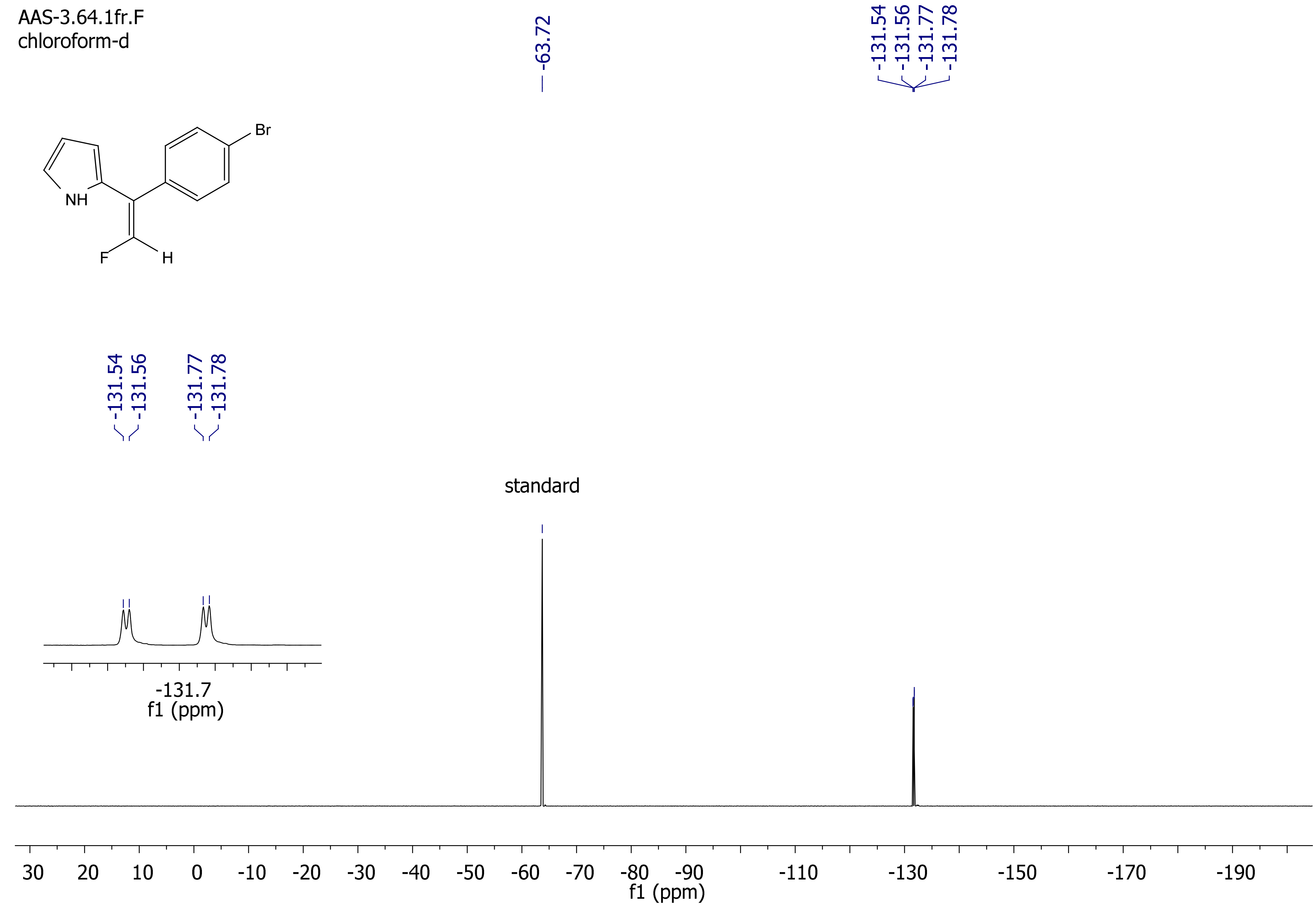

^{19}F NMR spectrum of (*Z*)-2-(1-(4-bromophenyl)-2-fluorovinyl)-1*H*-pyrrole (*Z*-**4f**)

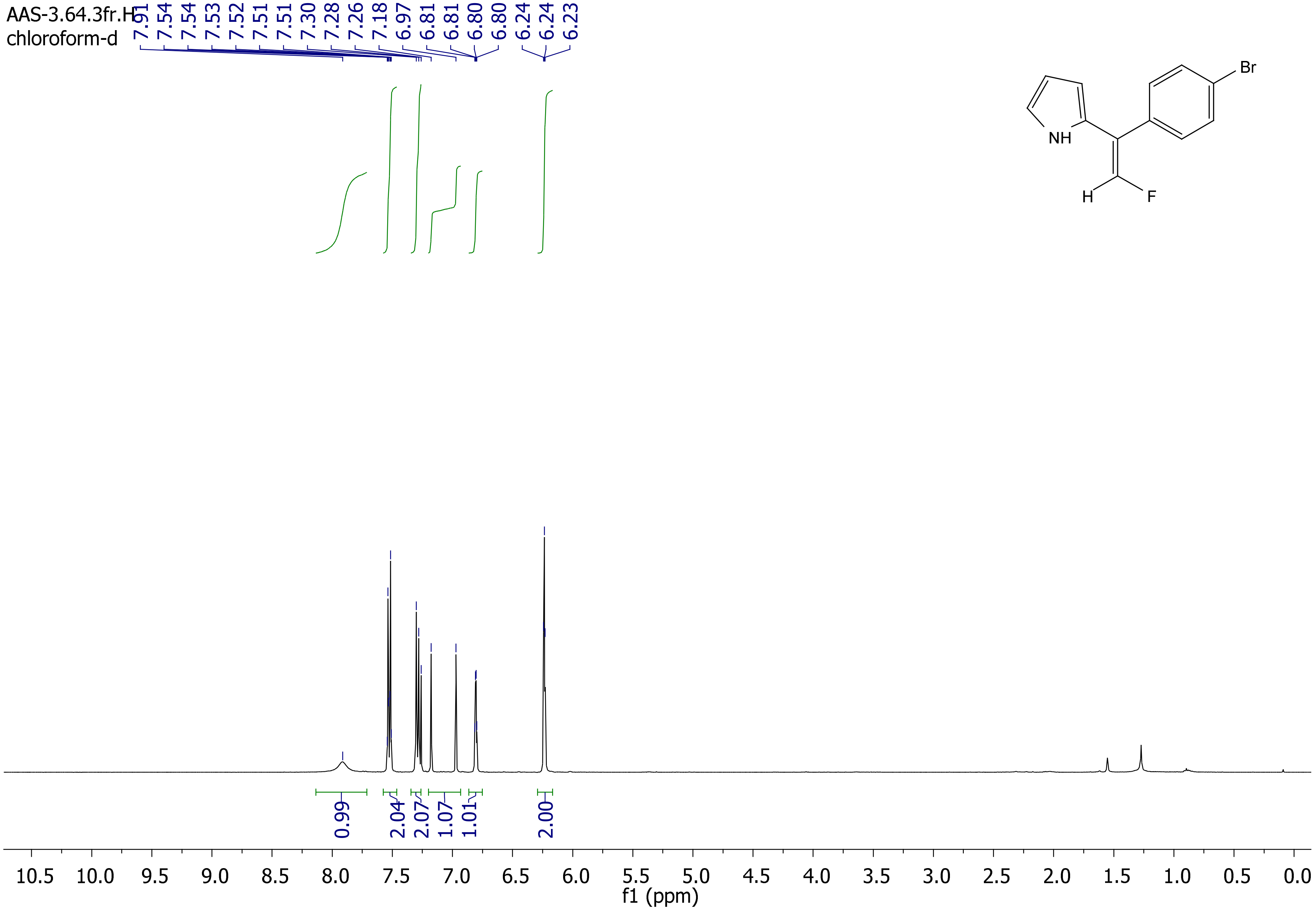

^{1}H NMR spectrum of (*E*)-2-(1-(4-bromophenyl)-2-fluorovinyl)-1*H*-pyrrole (*E*-**4f**)

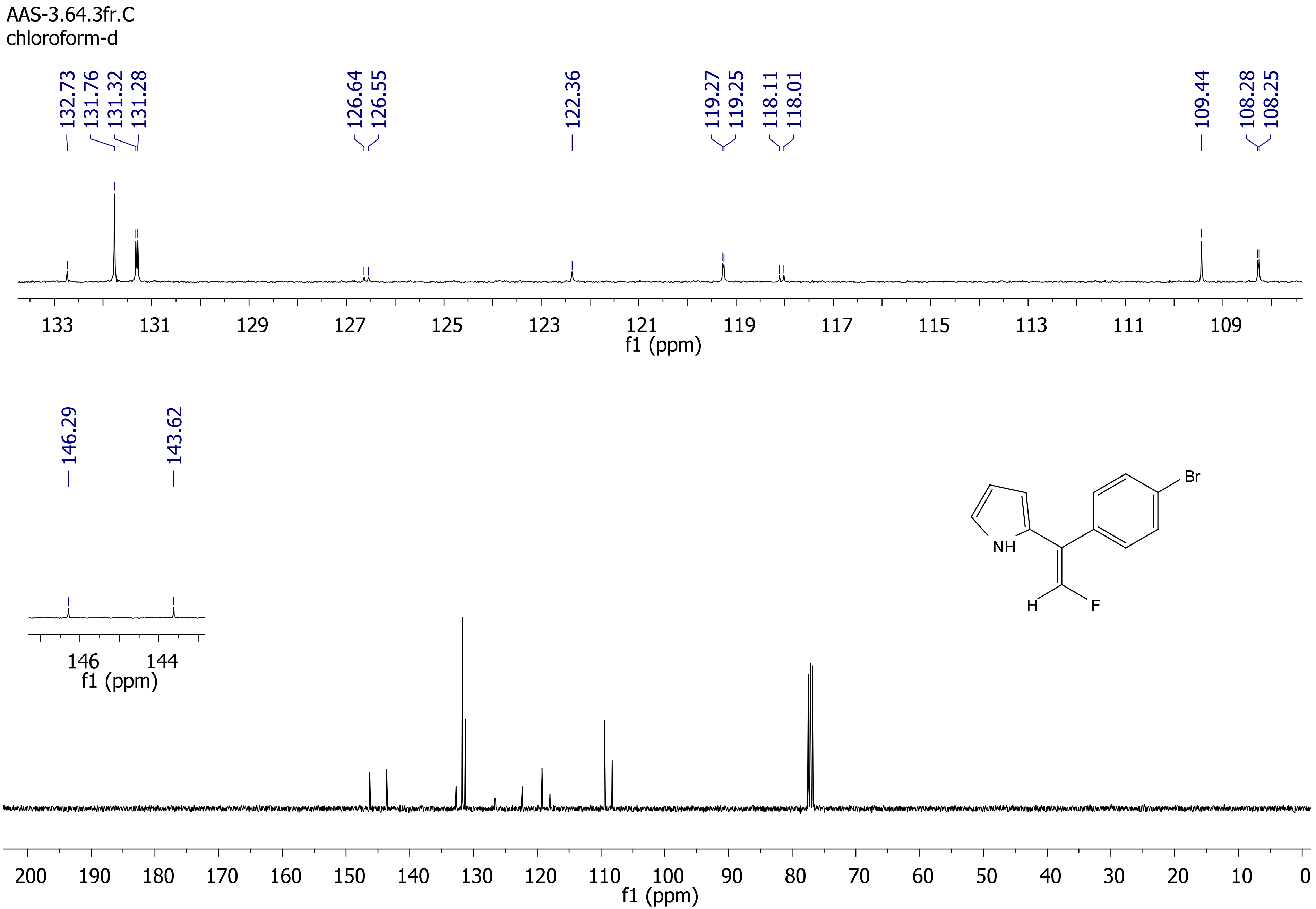

^{13}C NMR spectrum of (*E*)-2-(1-(4-bromophenyl)-2-fluorovinyl)-1*H*-pyrrole (*E*-**4f**)

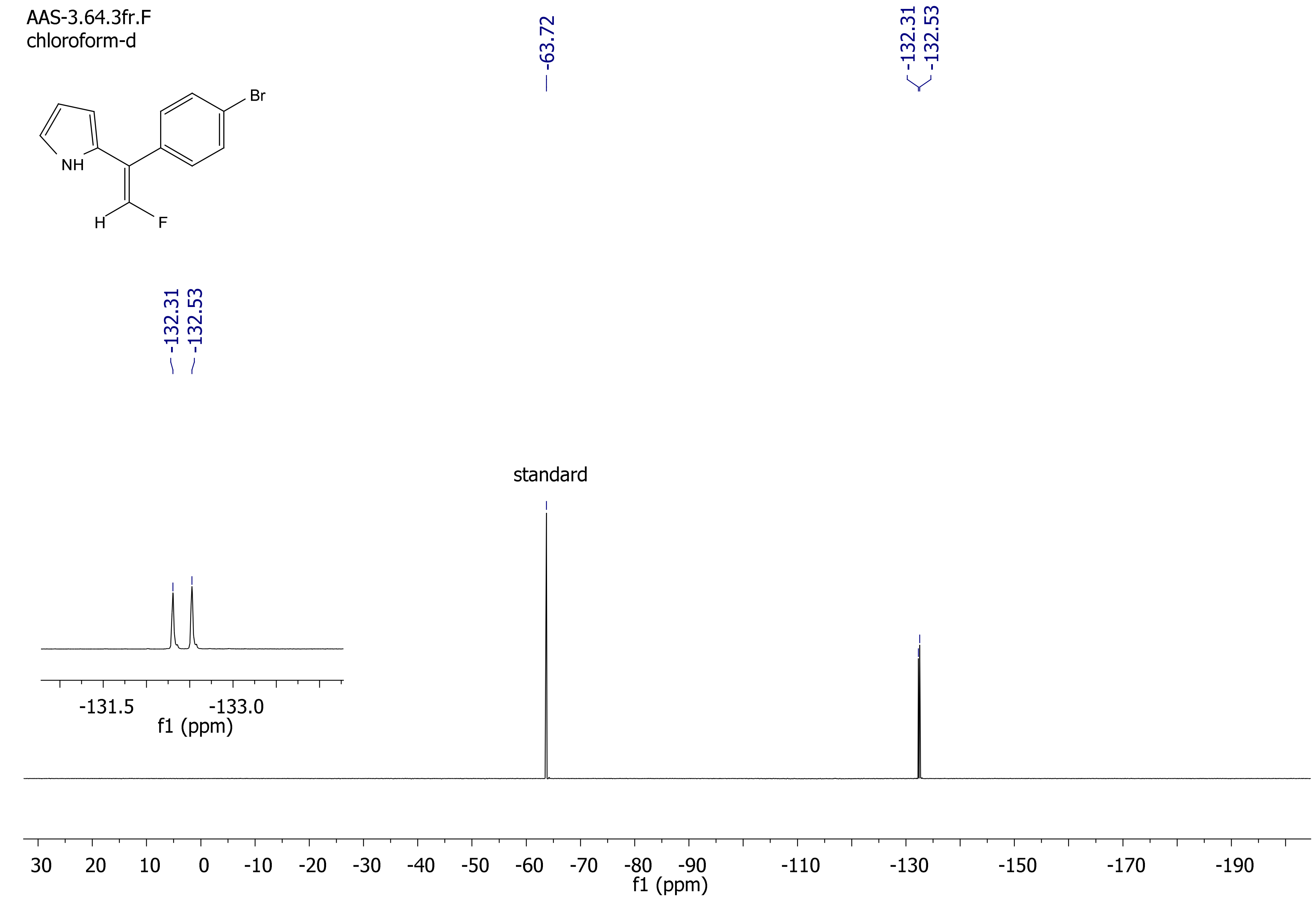

^{19}F NMR spectrum of (*E*)-2-(1-(4-bromophenyl)-2-fluorovinyl)-1*H*-pyrrole (*E*-**4f**)

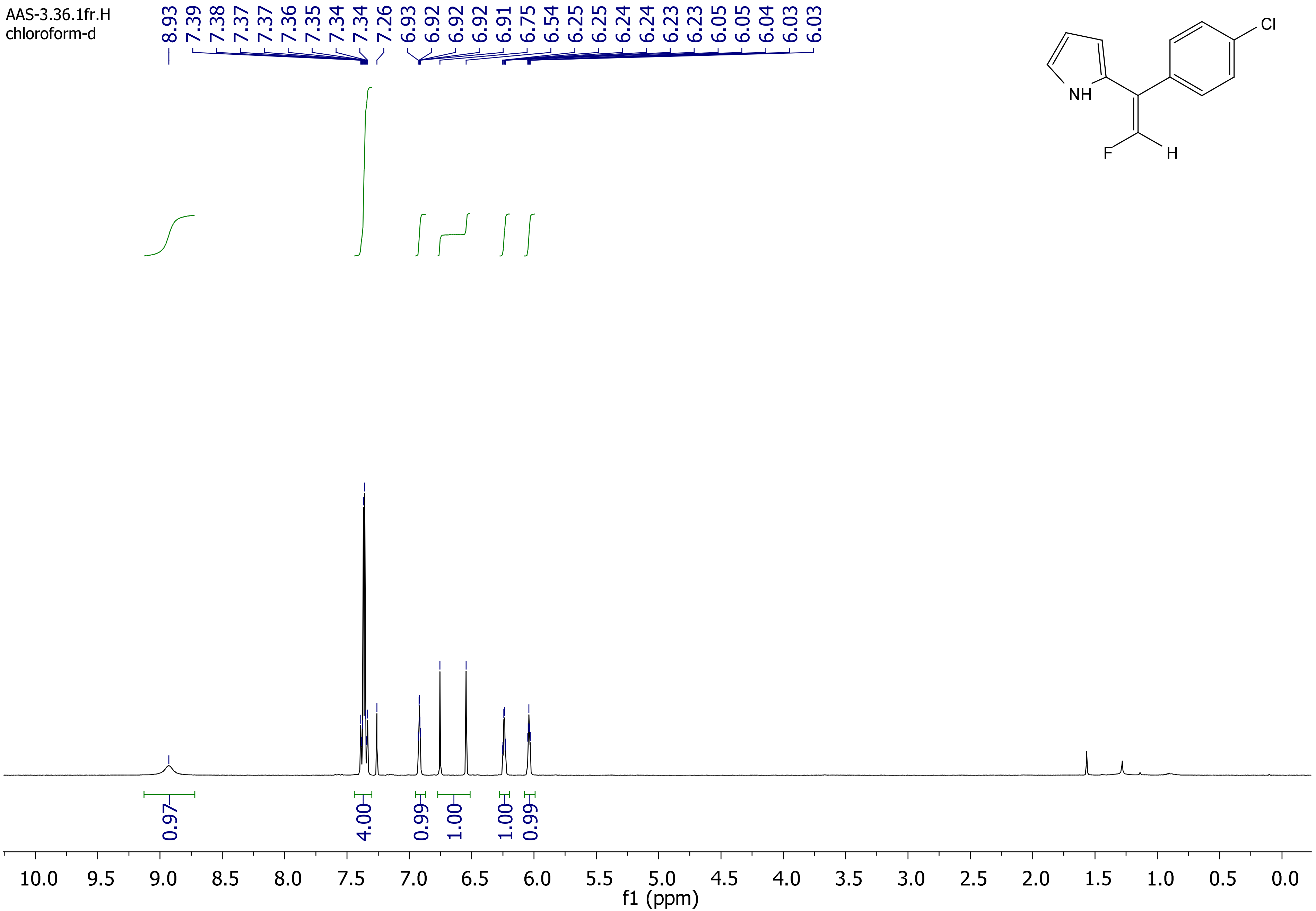

^{1}H NMR spectrum of (*Z*)-2-(1-(4-chlorophenyl)-2-fluorovinyl)-1*H*-pyrrole (*Z*-**4g**)

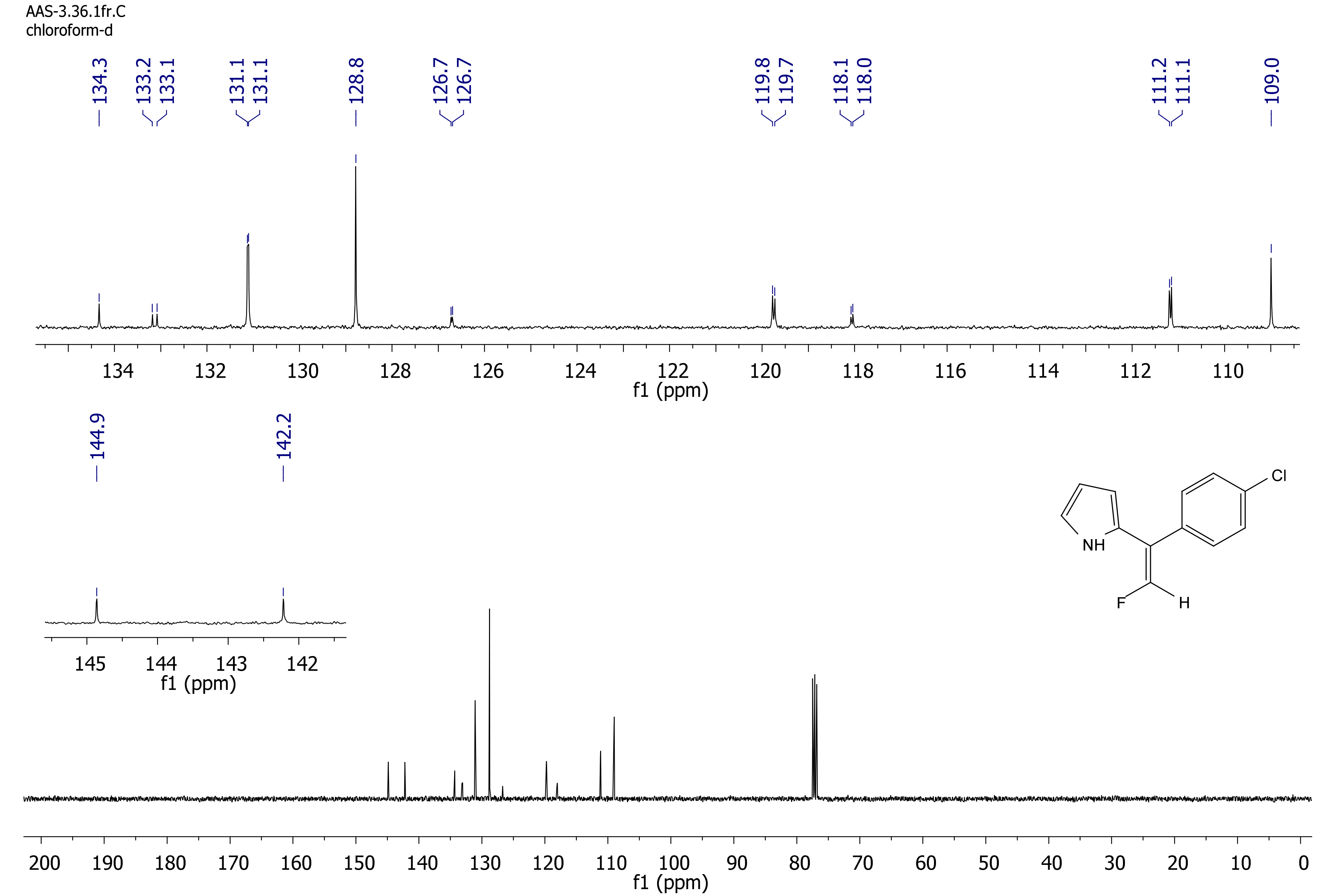

^{13}C NMR spectrum of (*Z*)-2-(1-(4-chlorophenyl)-2-fluorovinyl)-1*H*-pyrrole (*Z*-**4g**)

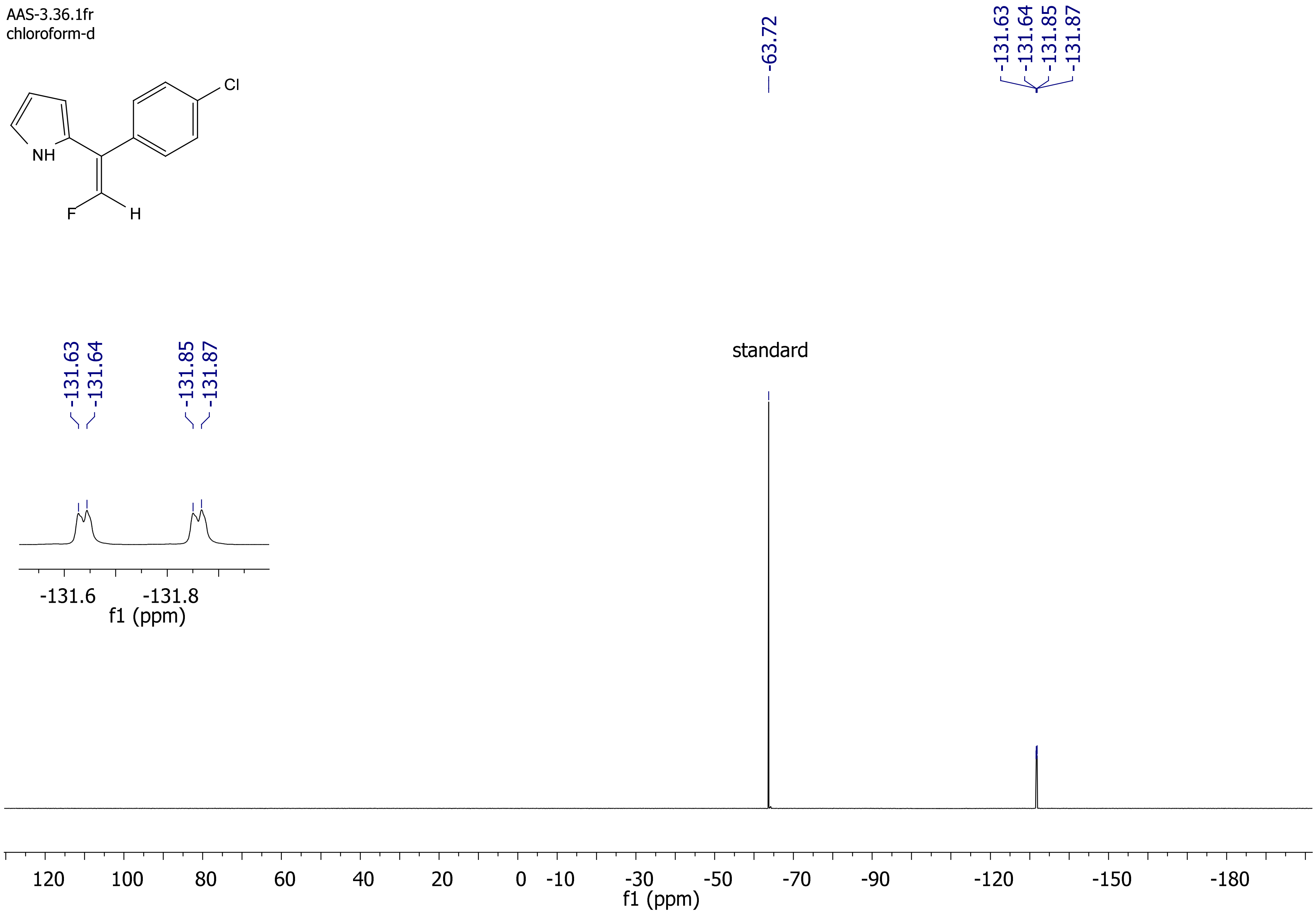

^{19}F NMR spectrum of (*Z*)-2-(1-(4-chlorophenyl)-2-fluorovinyl)-1*H*-pyrrole (*Z*-**4g**)

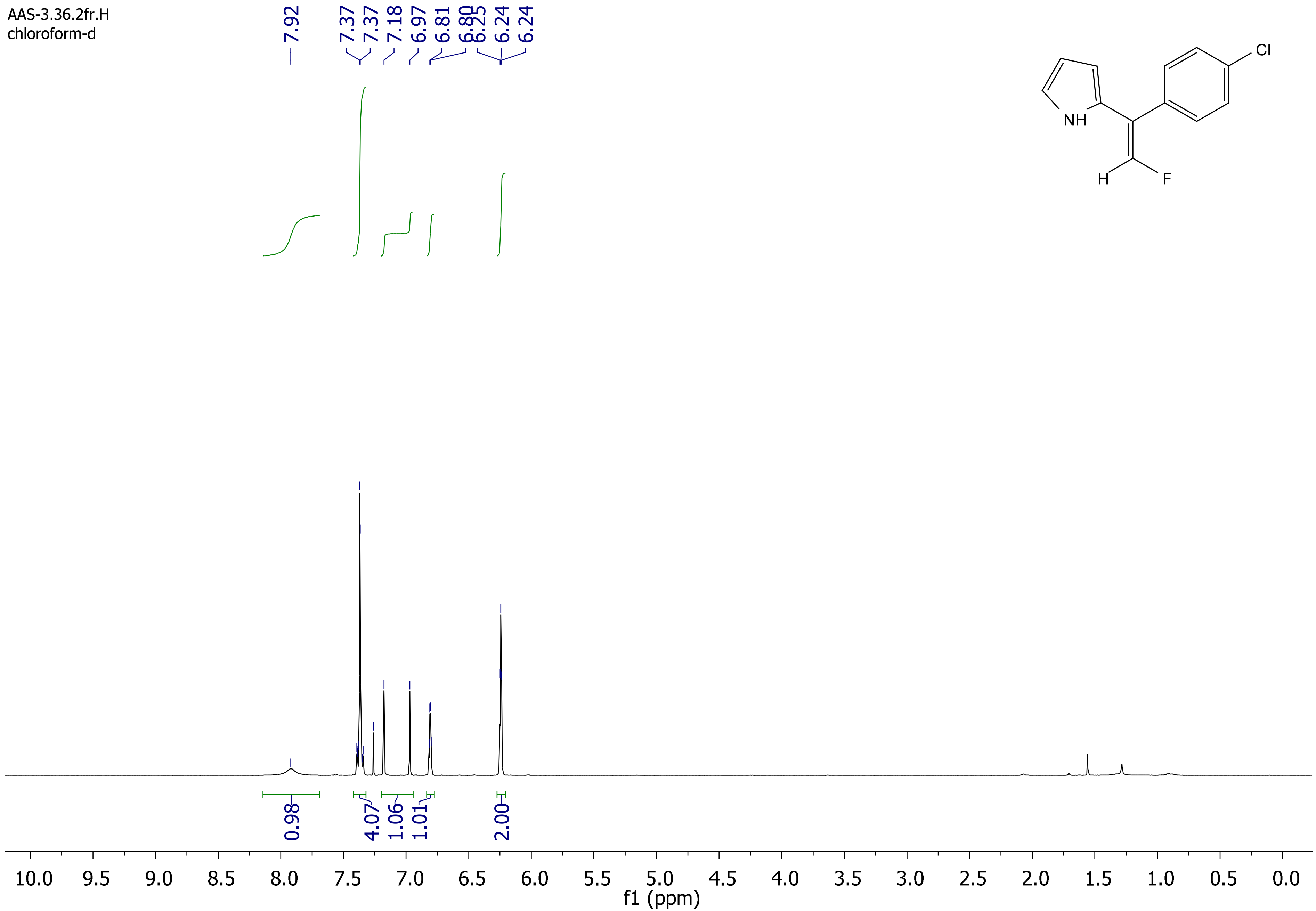

^{1}H NMR spectrum of (*E*)-2-(1-(4-chlorophenyl)-2-fluorovinyl)-1*H*-pyrrole (*E*-**4g**)

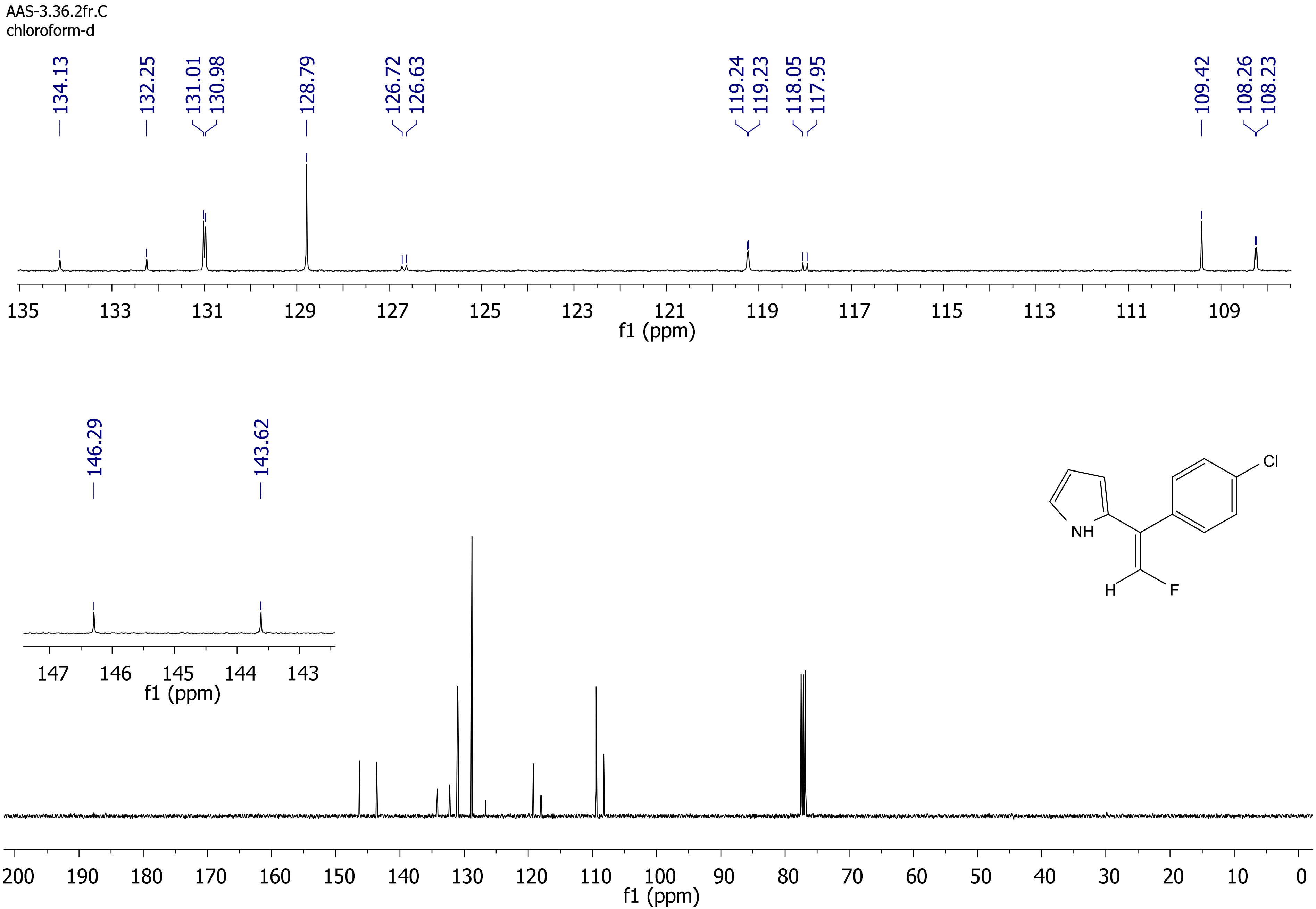

^{13}C NMR spectrum of (*E*)-2-(1-(4-chlorophenyl)-2-fluorovinyl)-1*H*-pyrrole (*E*-**4g**)

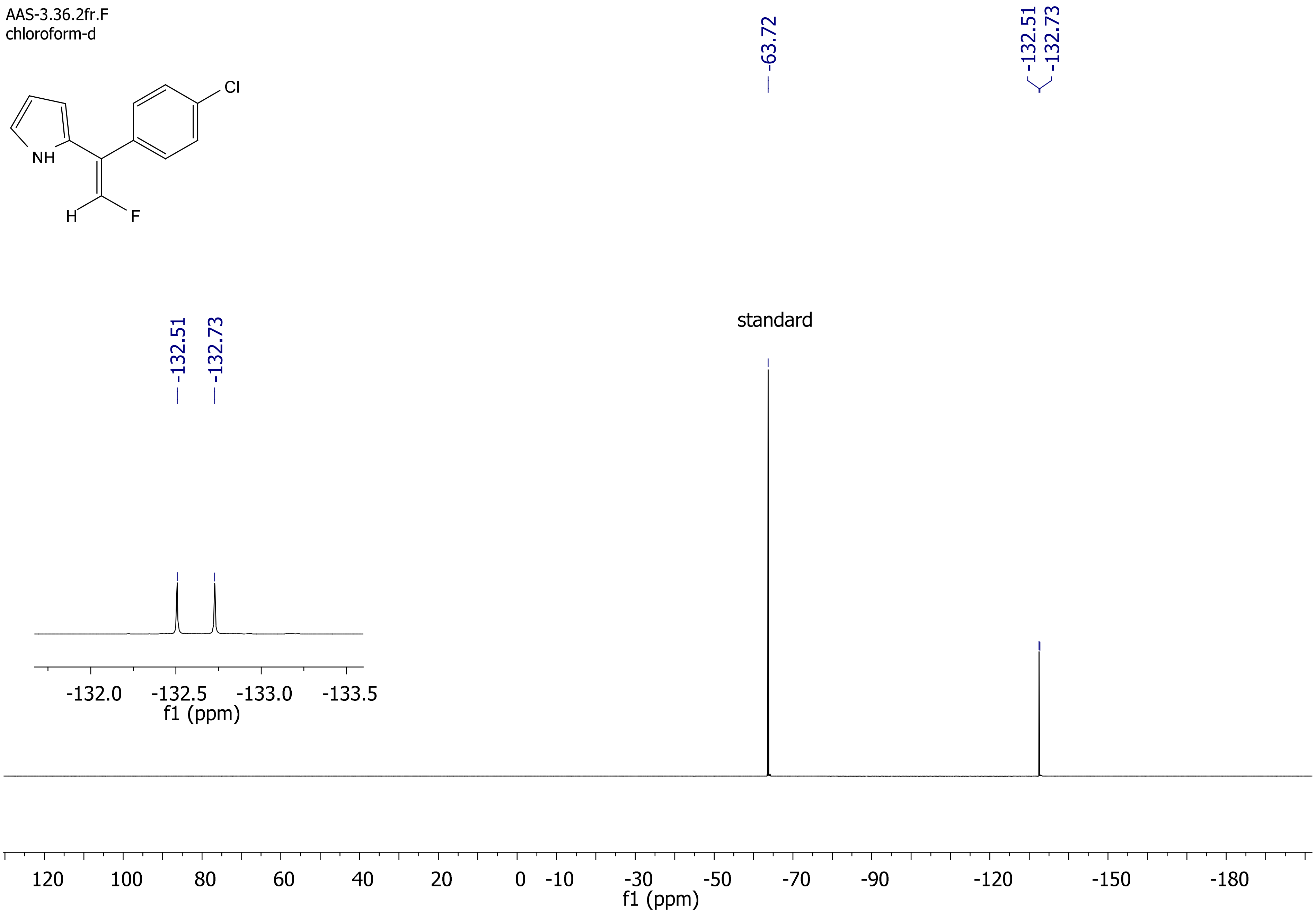

^{19}F NMR spectrum of (*E*)-2-(1-(4-chlorophenyl)-2-fluorovinyl)-1*H*-pyrrole (*E*-**4g**)

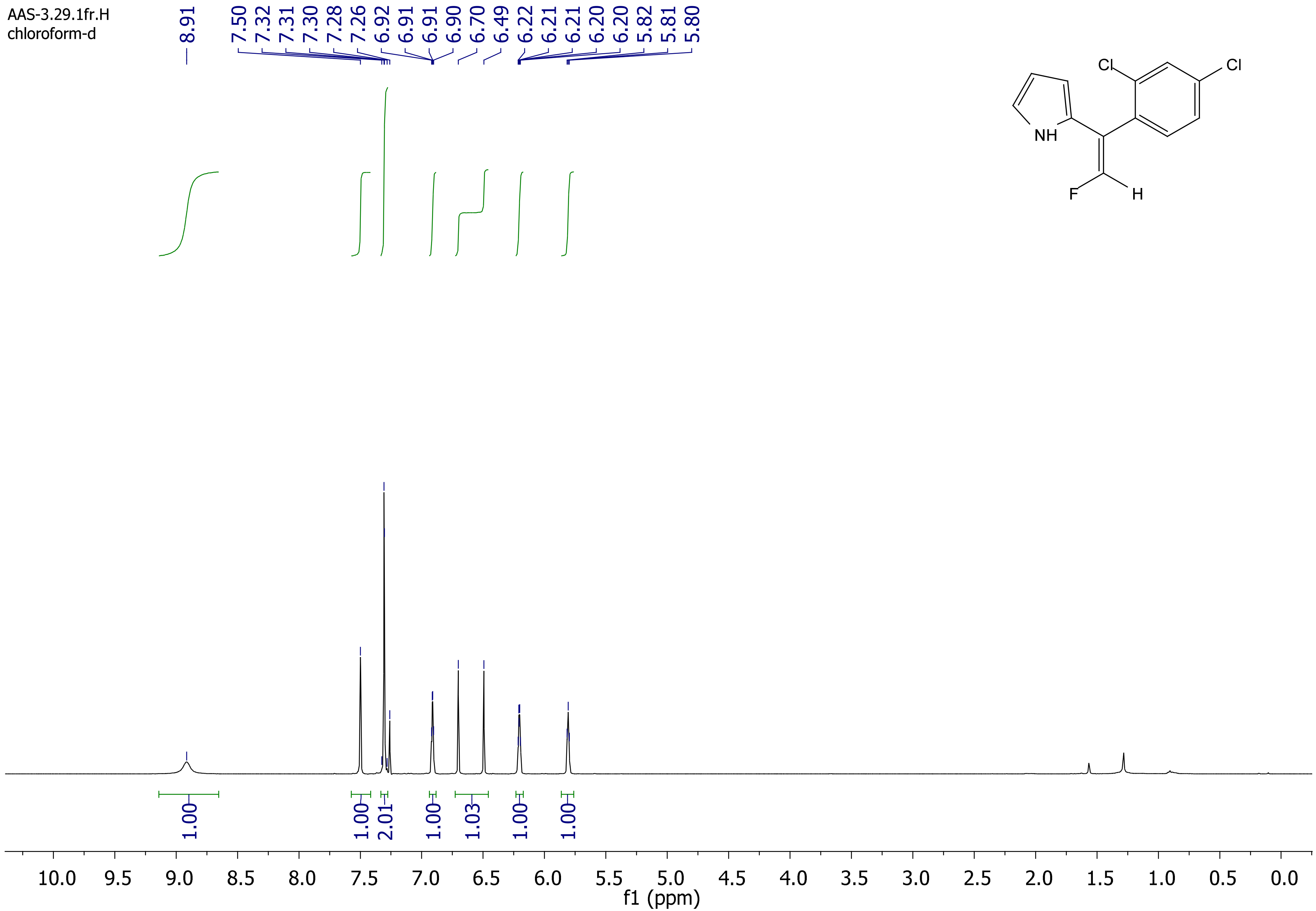

^{1}H NMR spectrum of (*Z*)-2-(1-(2,4-dichlorophenyl)-2-fluorovinyl)-1*H*-pyrrole (*Z*-**4h**)

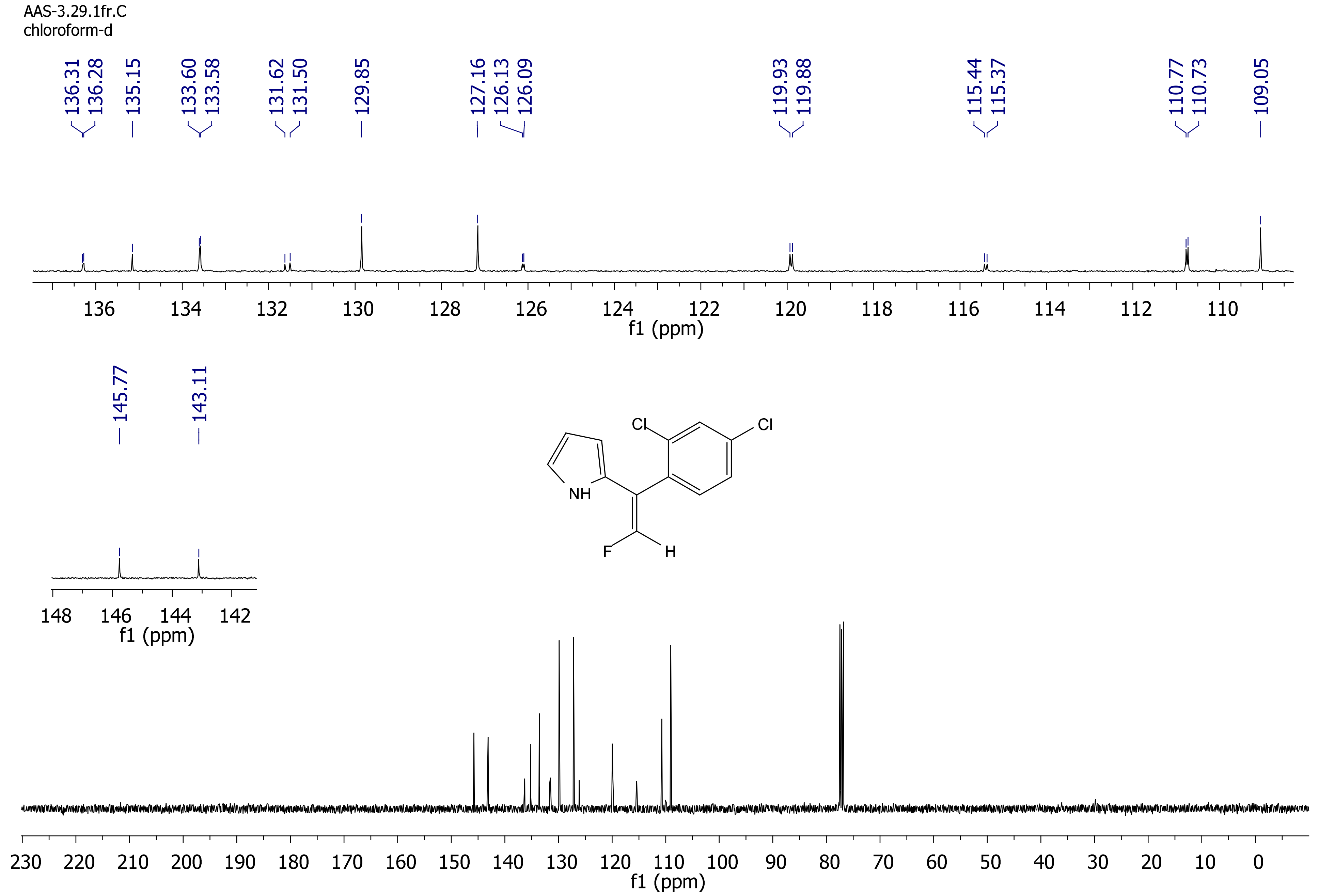

^{13}C NMR spectrum of (*Z*)-2-(1-(2,4-dichlorophenyl)-2-fluorovinyl)-1*H*-pyrrole (*Z*-**4h**)

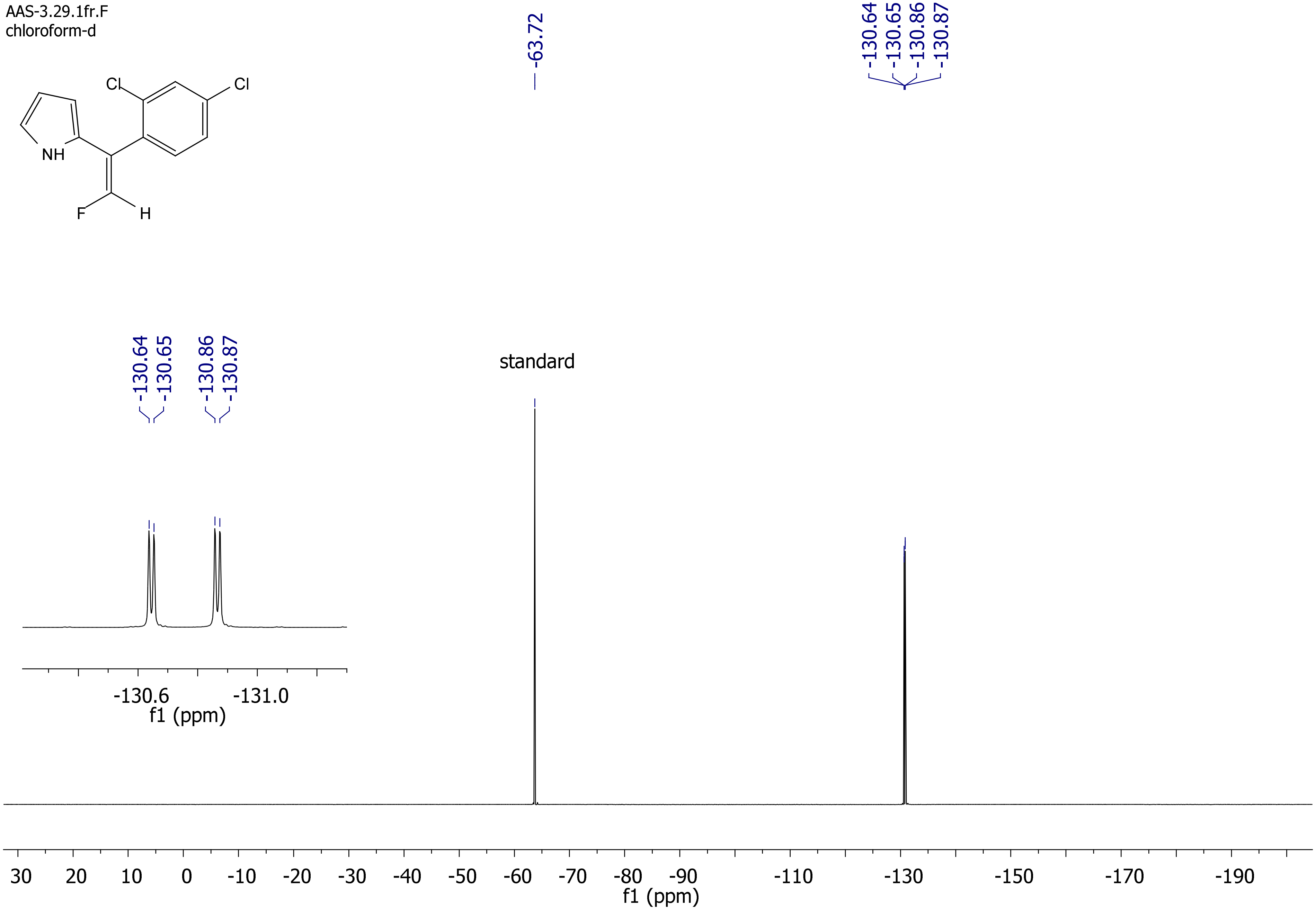

^{19}F NMR spectrum of (*Z*)-2-(1-(2,4-dichlorophenyl)-2-fluorovinyl)-1*H*-pyrrole (*Z*-**4h**)

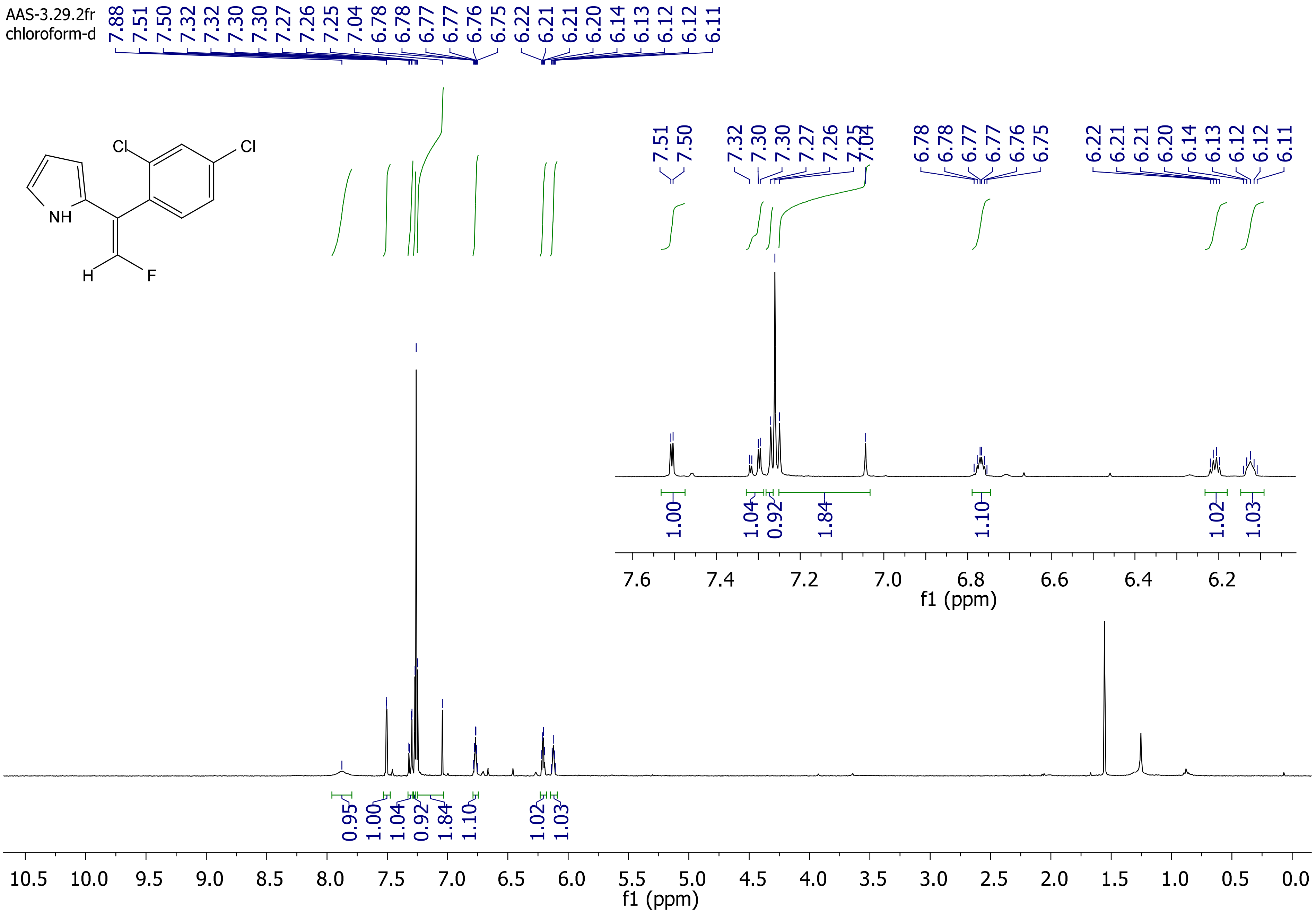

^{1}H NMR spectrum of (*E*)-2-(1-(2,4-dichlorophenyl)-2-fluorovinyl)-1*H*-pyrrole (*E*-**4h**)

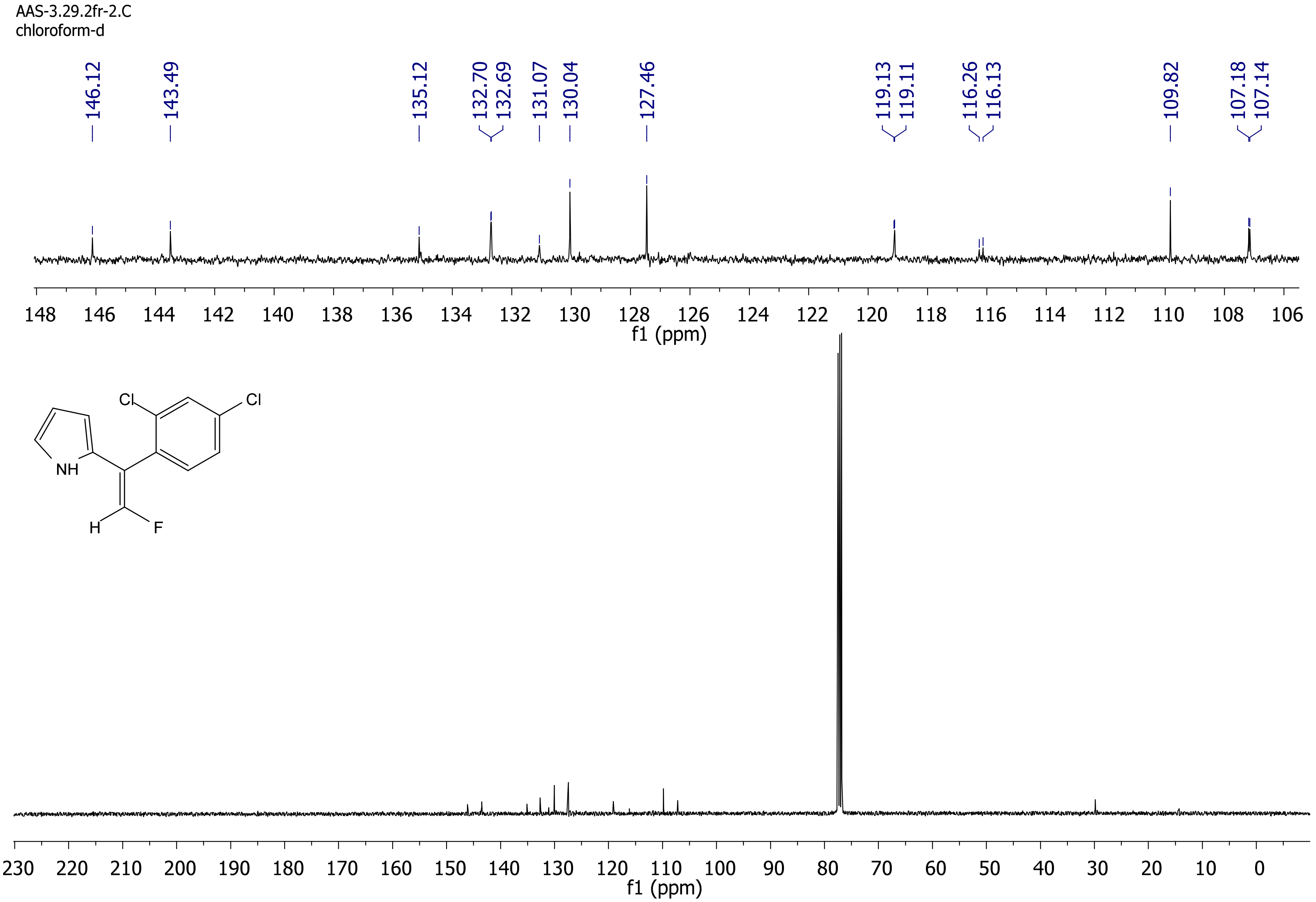

^{13}C NMR spectrum of (*E*)-2-(1-(2,4-dichlorophenyl)-2-fluorovinyl)-1*H*-pyrrole (*E*-**4h**)

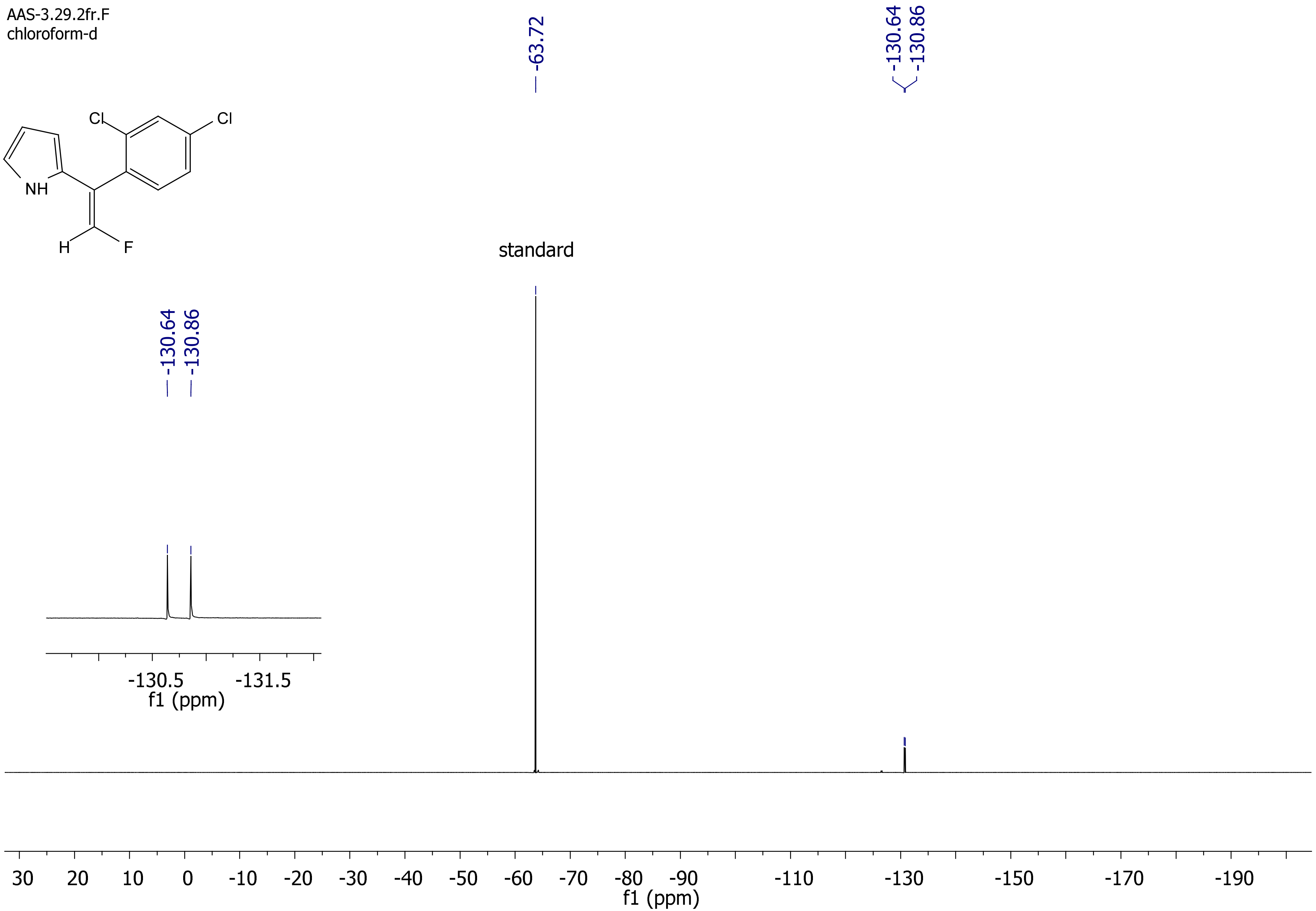

^{19}F NMR spectrum of (*E*)-2-(1-(2,4-dichlorophenyl)-2-fluorovinyl)-1*H*-pyrrole (*E*-**4h**)

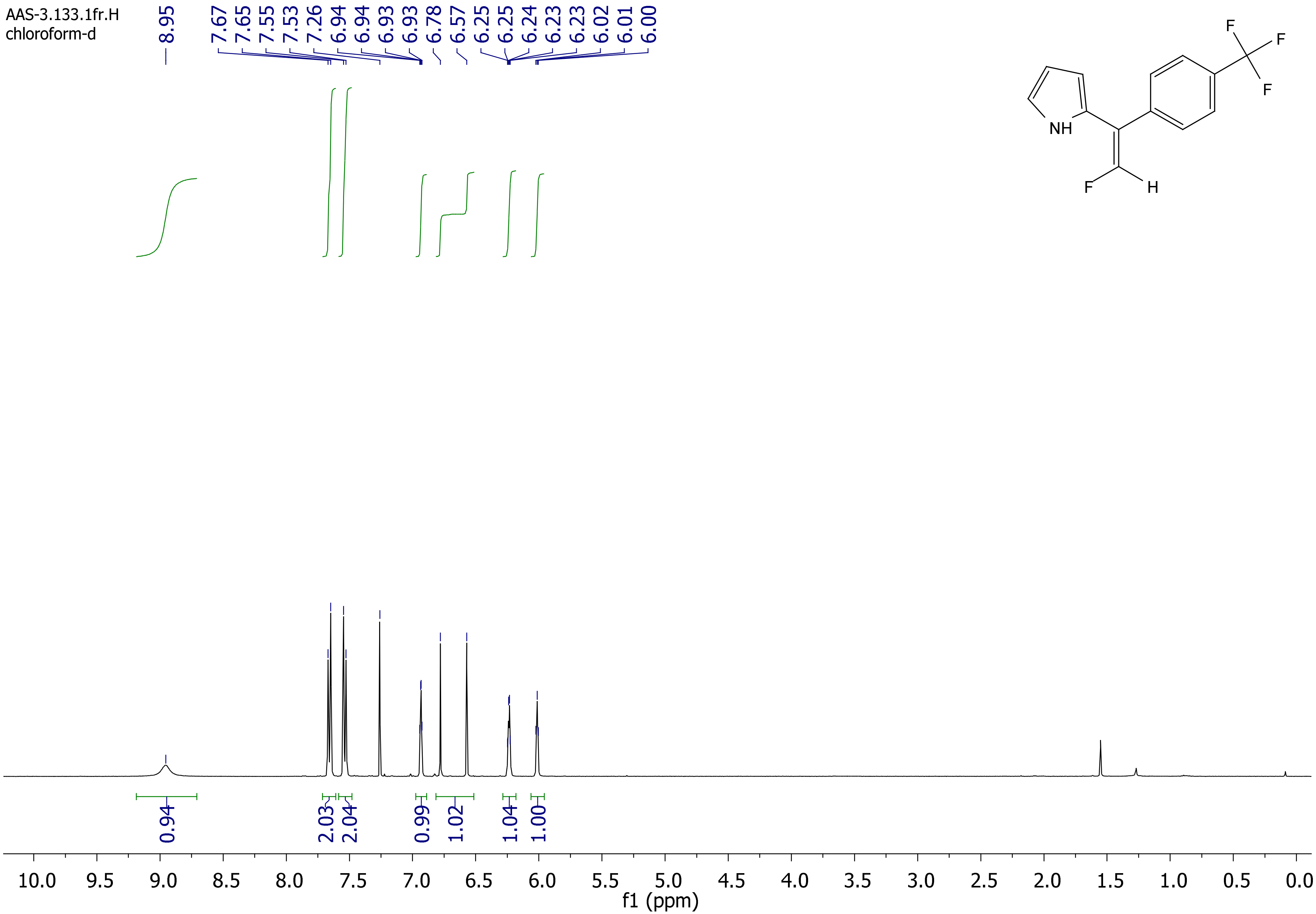

^{1}H NMR spectrum of (*Z*)-2-(2-fluoro-1-(4-(trifluoromethyl)phenyl)vinyl)-1*H*-pyrrole (*Z*-**4i**)

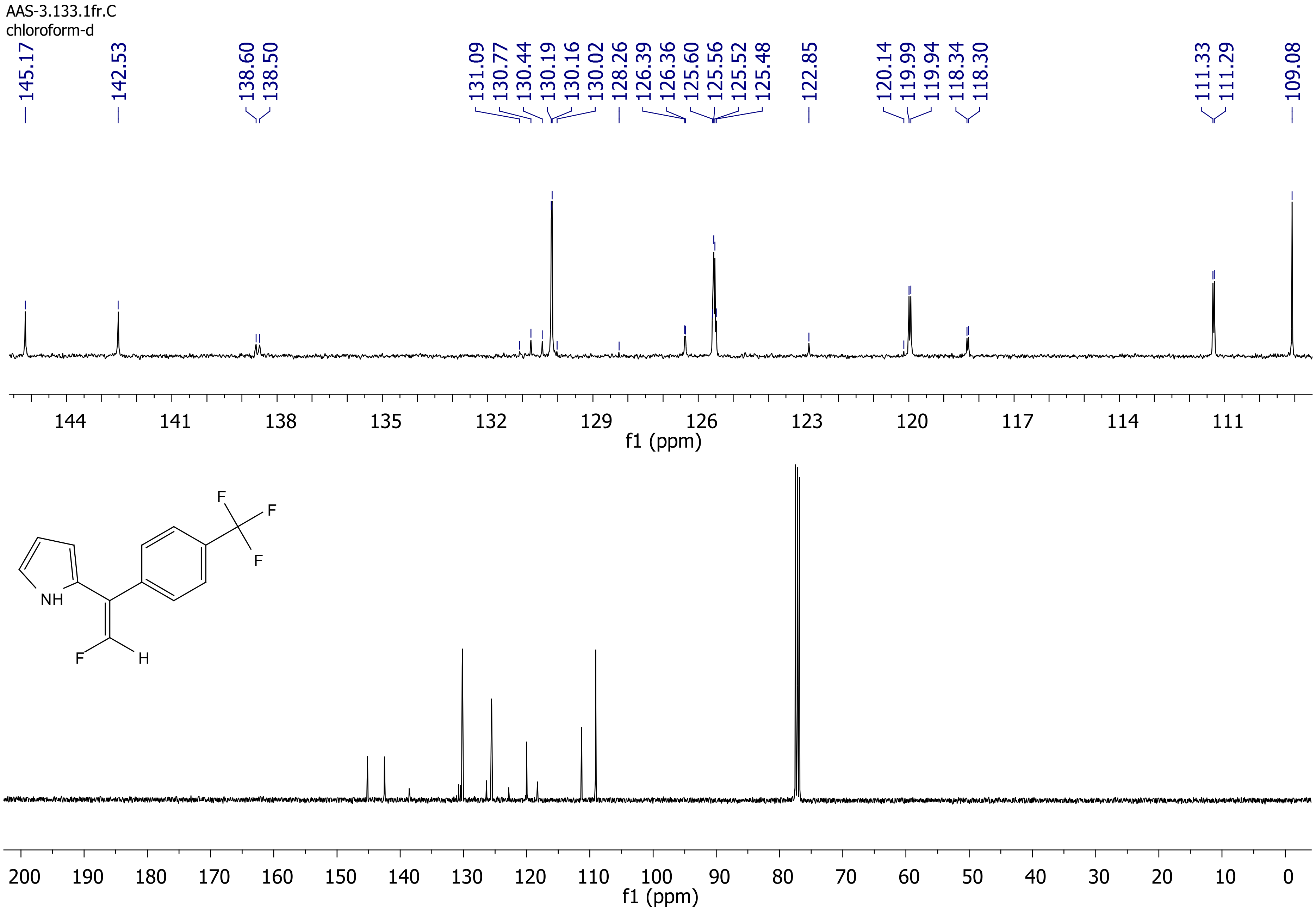

^{13}C NMR spectrum of (*Z*)-2-(2-fluoro-1-(4-(trifluoromethyl)phenyl)vinyl)-1*H*-pyrrole (*Z*-**4i**)

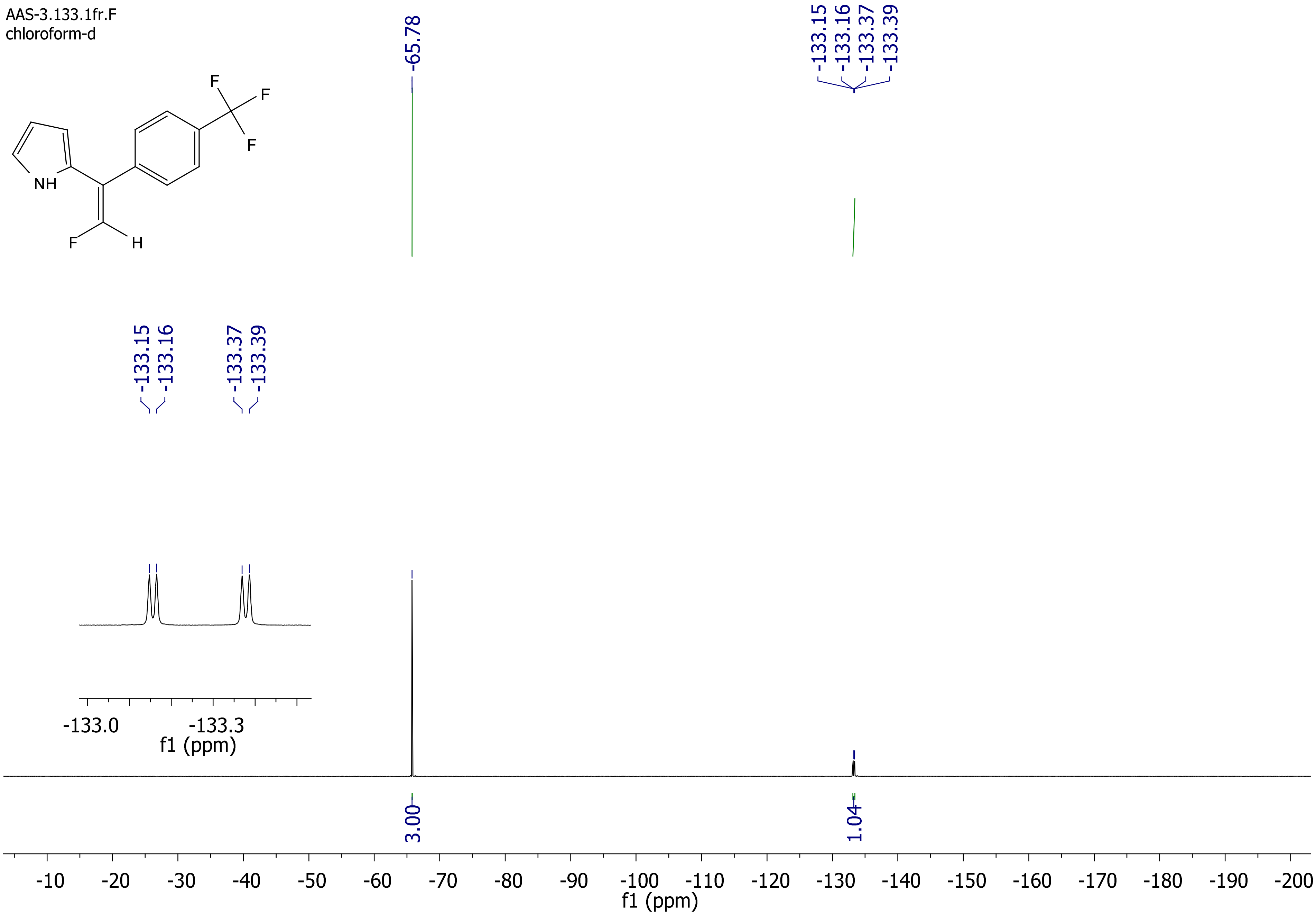

^{19}F NMR spectrum of (*Z*)-2-(2-fluoro-1-(4-(trifluoromethyl)phenyl)vinyl)-1*H*-pyrrole (*Z*-**4i**)

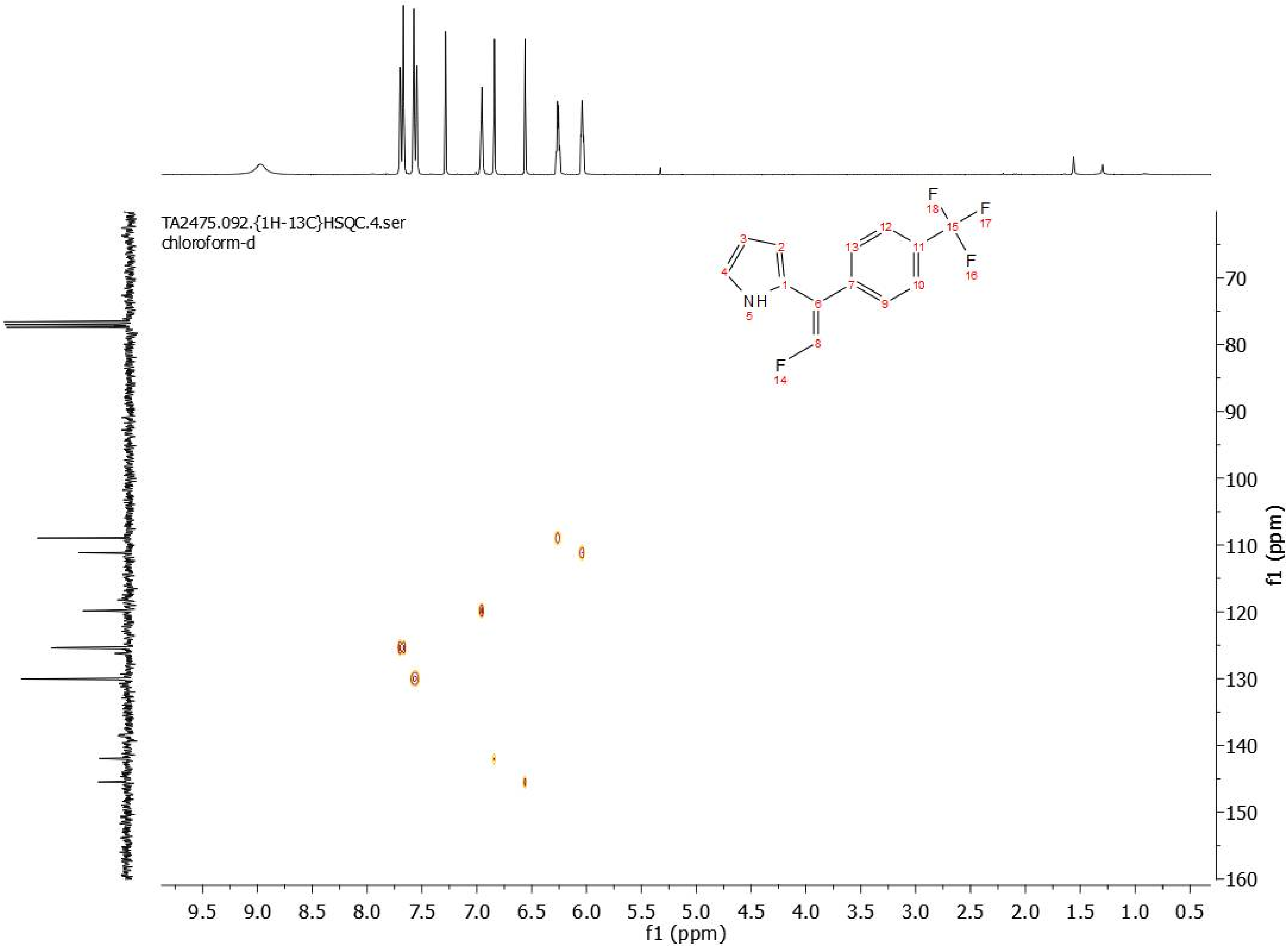

HSQC ^{1}H-^{13}C spectra of (*Z*)-2-(2-fluoro-1-(4-(trifluoromethyl)phenyl)vinyl)-1*H*-pyrrole (*Z*-**4i**)

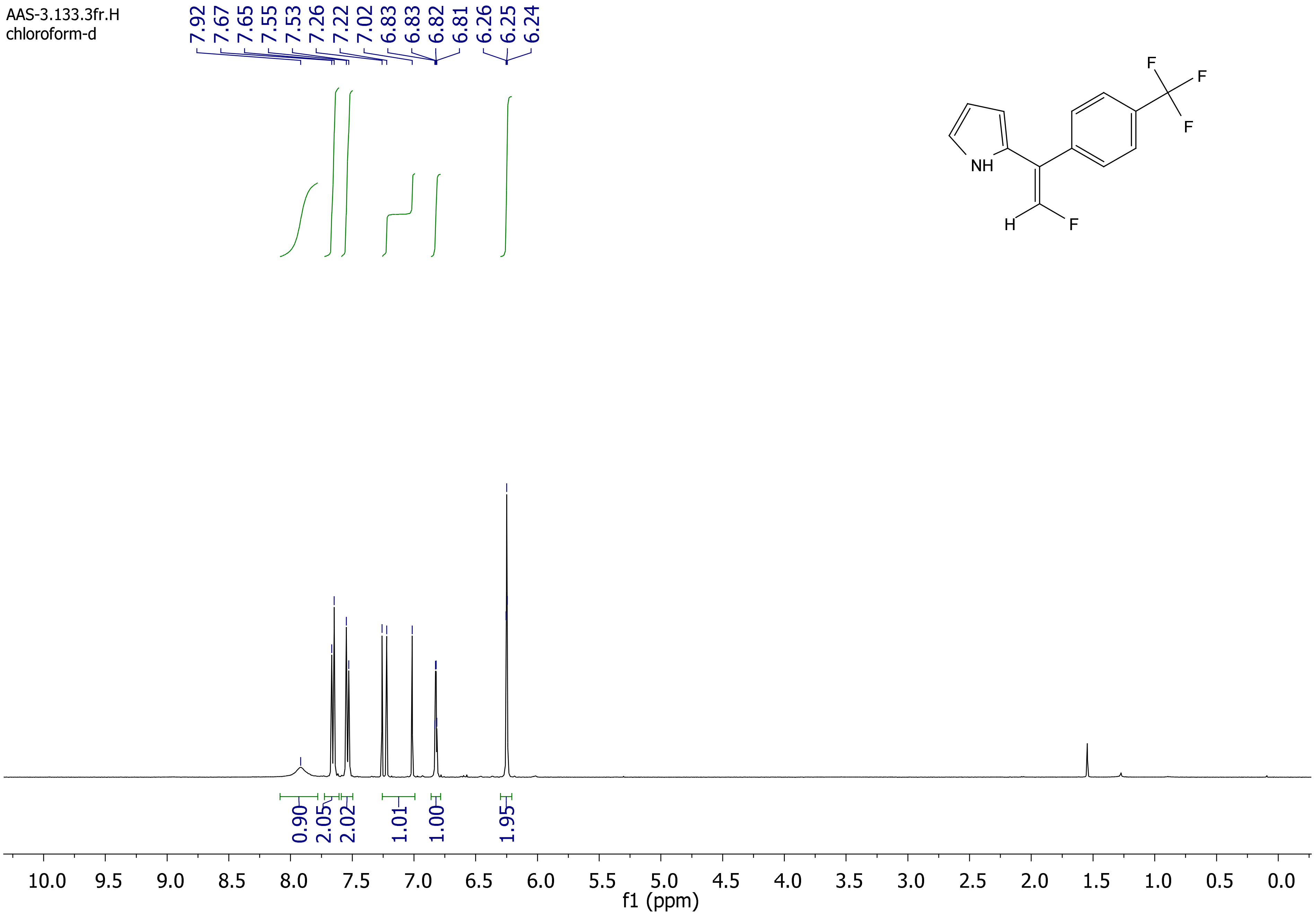

^{1}H NMR spectrum of (*E*)-2-(2-fluoro-1-(4-(trifluoromethyl)phenyl)vinyl)-1*H*-pyrrole (*E*-**4i**)

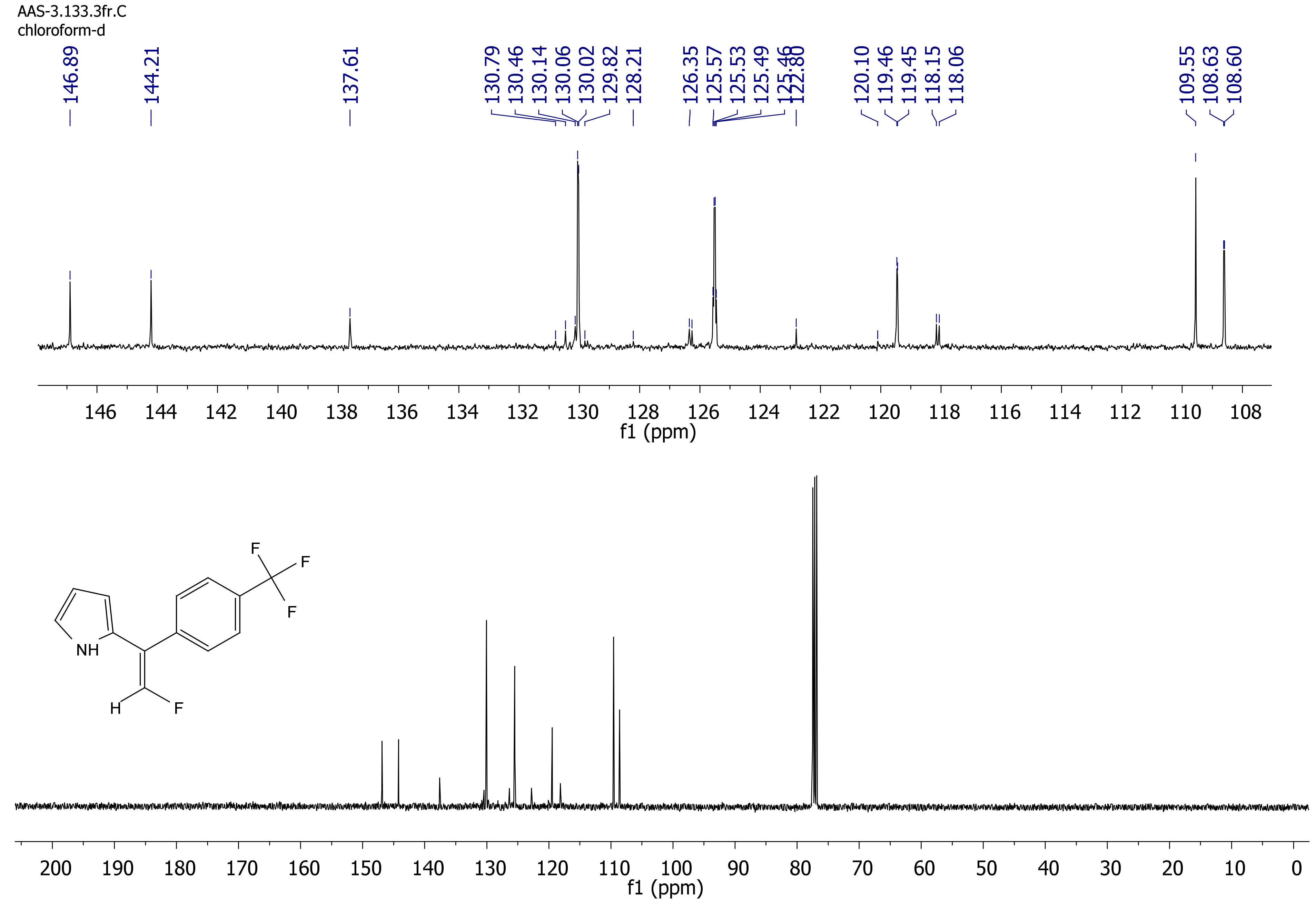

^{13}C NMR spectrum of (*E*)-2-(2-fluoro-1-(4-(trifluoromethyl)phenyl)vinyl)-1*H*-pyrrole (*E*-**4i**)

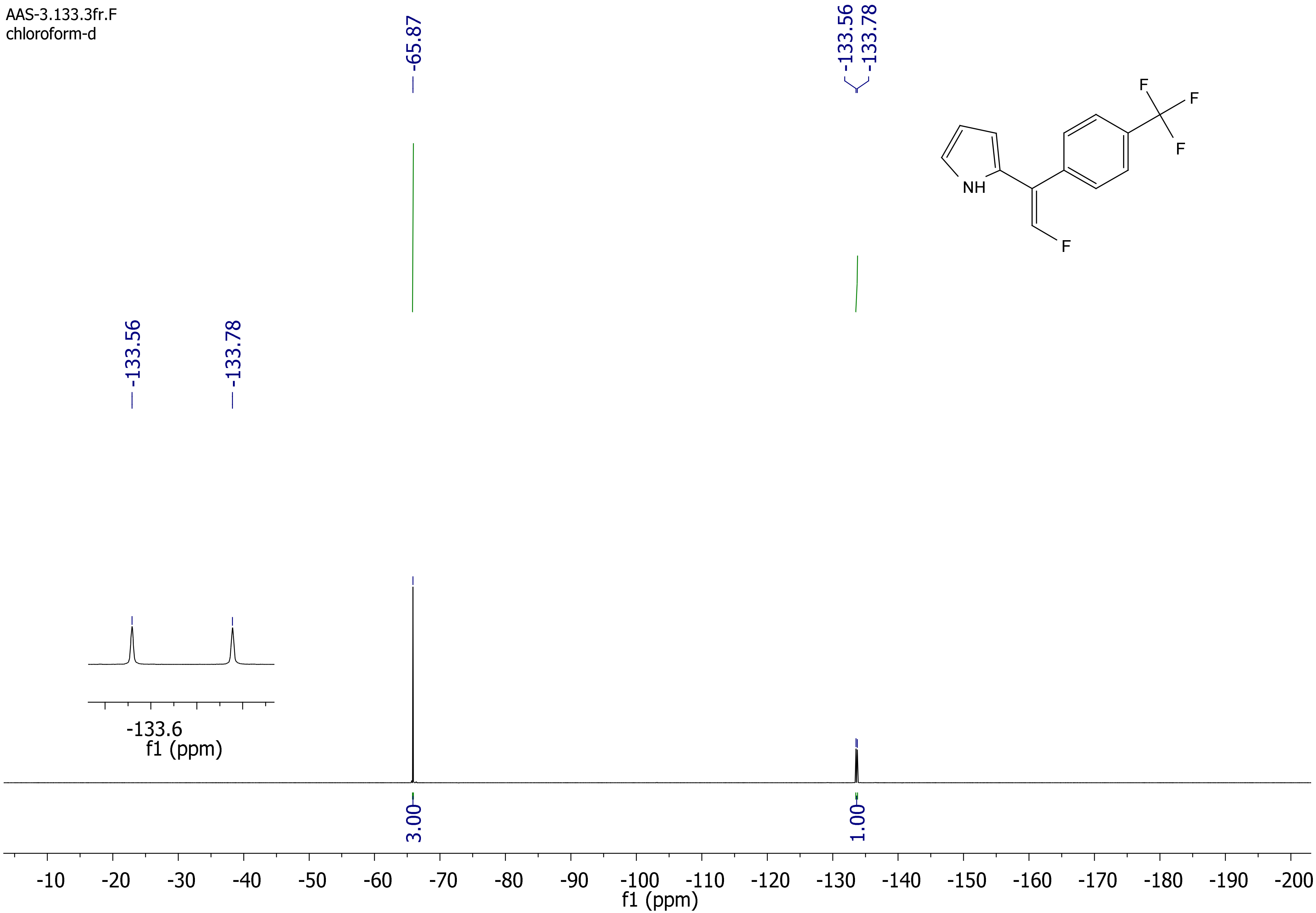

^{19}F NMR spectrum of (*E*)-2-(2-fluoro-1-(4-(trifluoromethyl)phenyl)vinyl)-1*H*-pyrrole (*E*-**4i**)

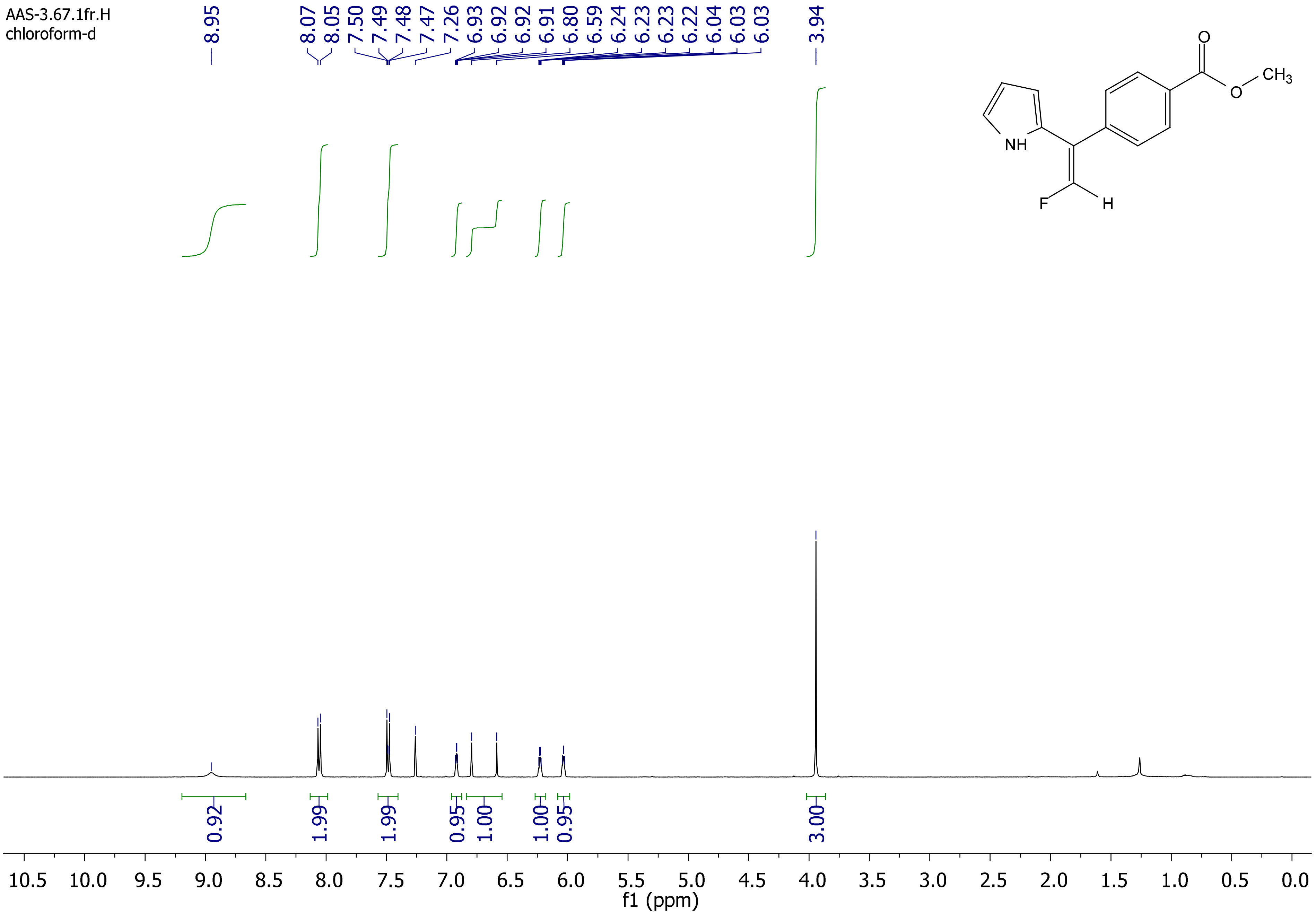

^{1}H NMR spectrum of (*Z*)-methyl 4-(2-fluoro-1-(1*H*-pyrrol-2-yl)vinyl)benzoate (Z-**4j**)

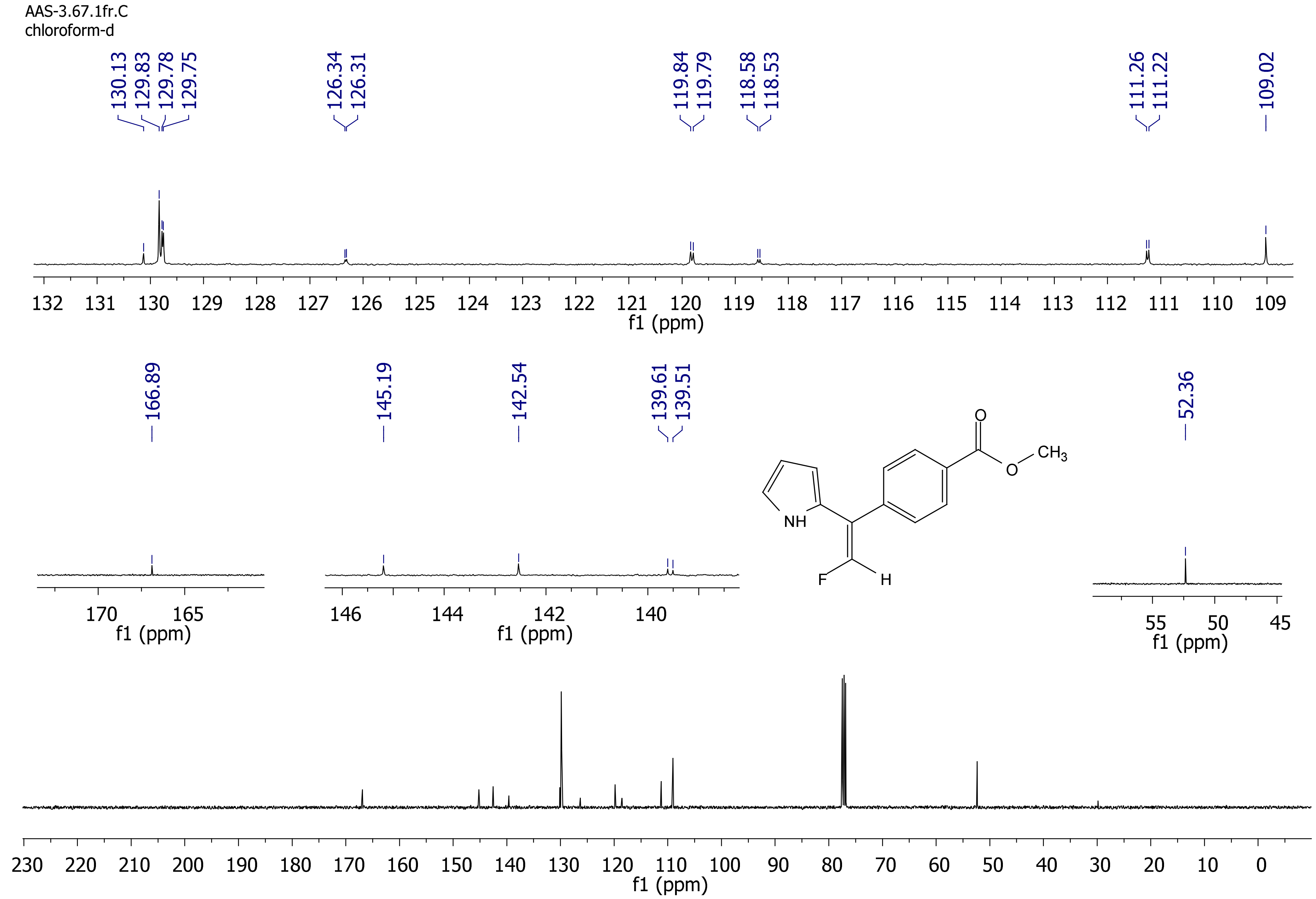

^{13}C NMR spectrum of (*Z*)-methyl 4-(2-fluoro-1-(1*H*-pyrrol-2-yl)vinyl)benzoate (Z-**4j**)

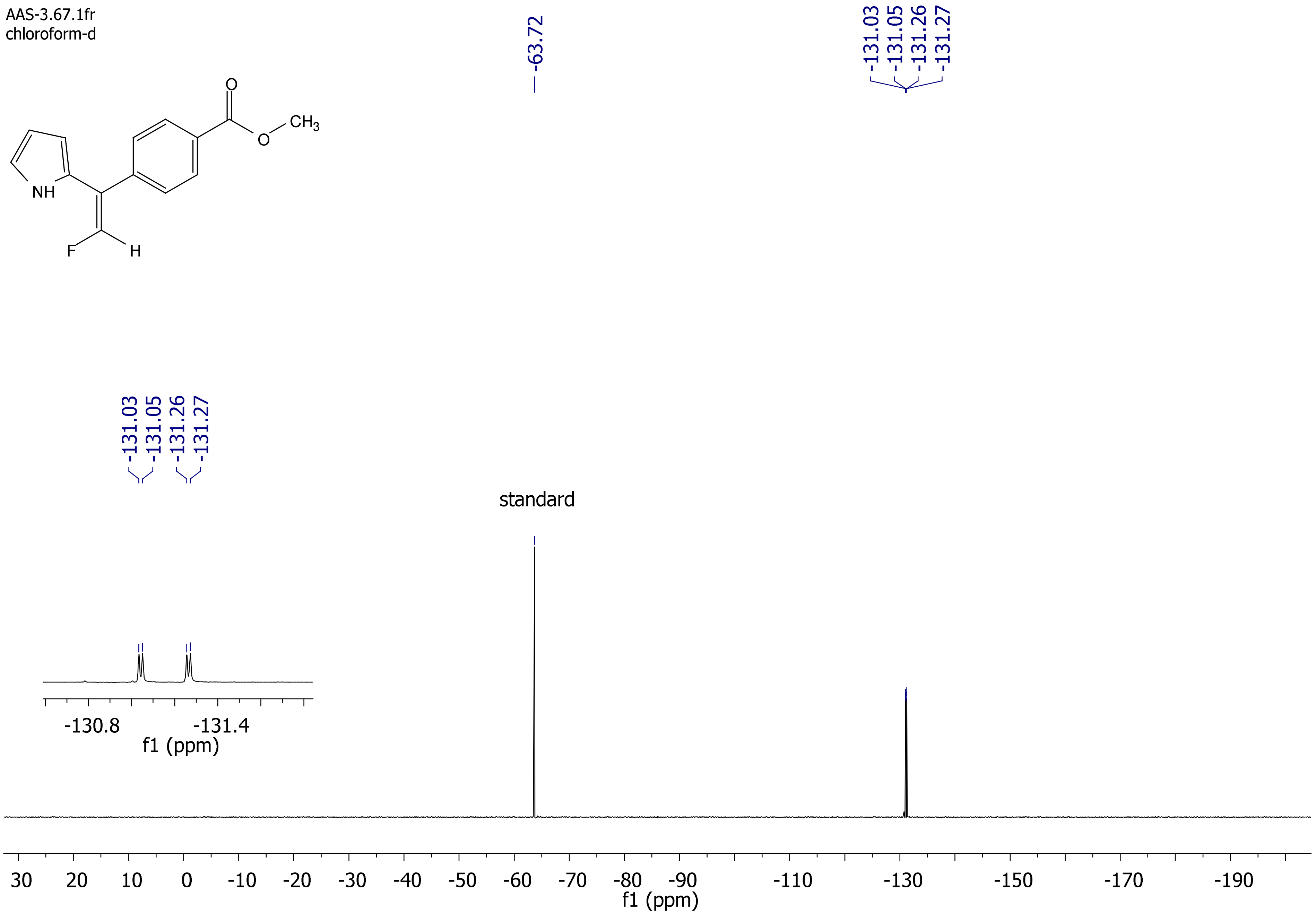

^{19}F NMR spectrum of (*Z*)-methyl 4-(2-fluoro-1-(1*H*-pyrrol-2-yl)vinyl)benzoate (Z-**4j**)

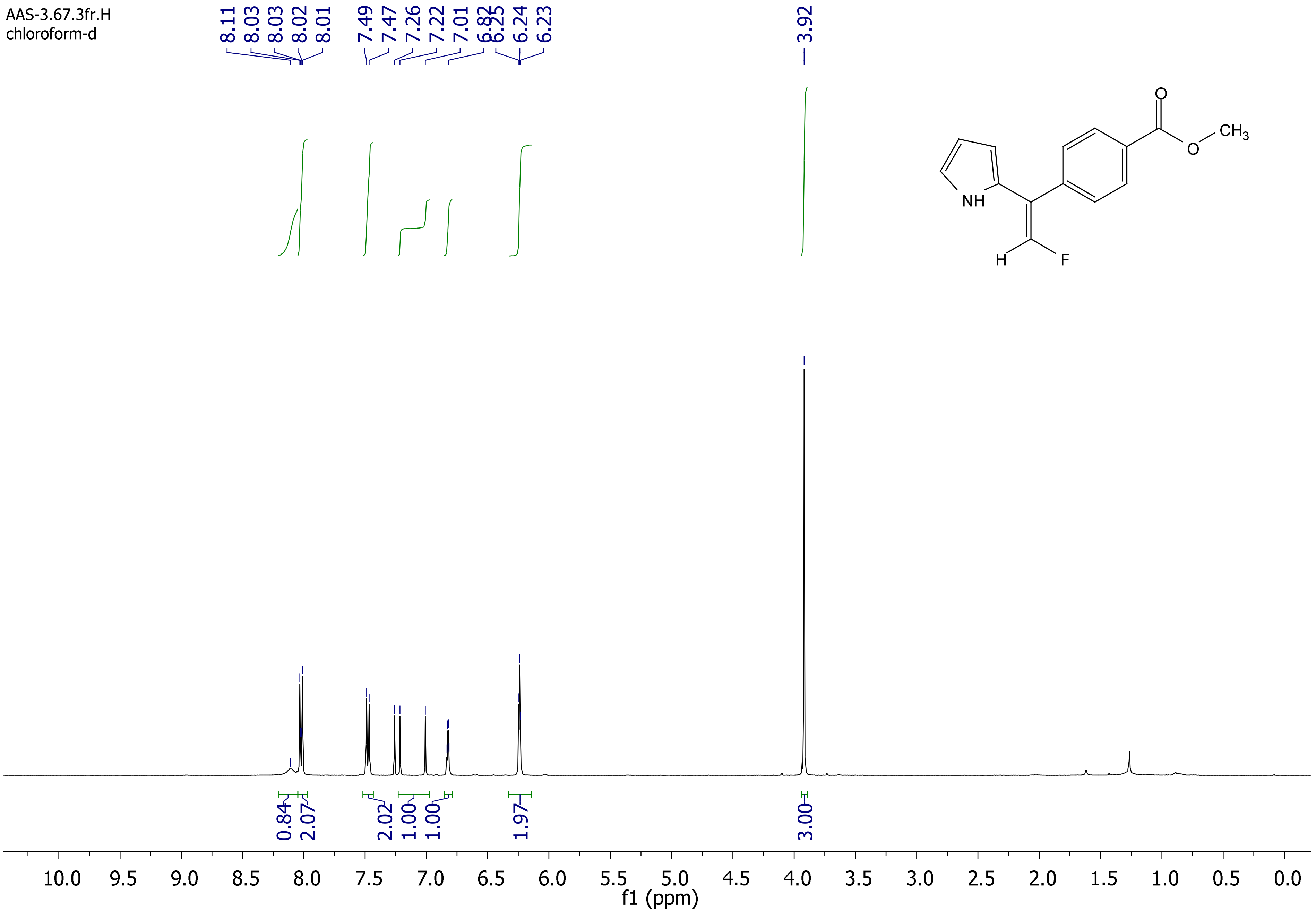

^{1}H NMR spectrum of (*E*)-methyl 4-(2-fluoro-1-(1*H*-pyrrol-2-yl)vinyl)benzoate (*E*-**4j**)

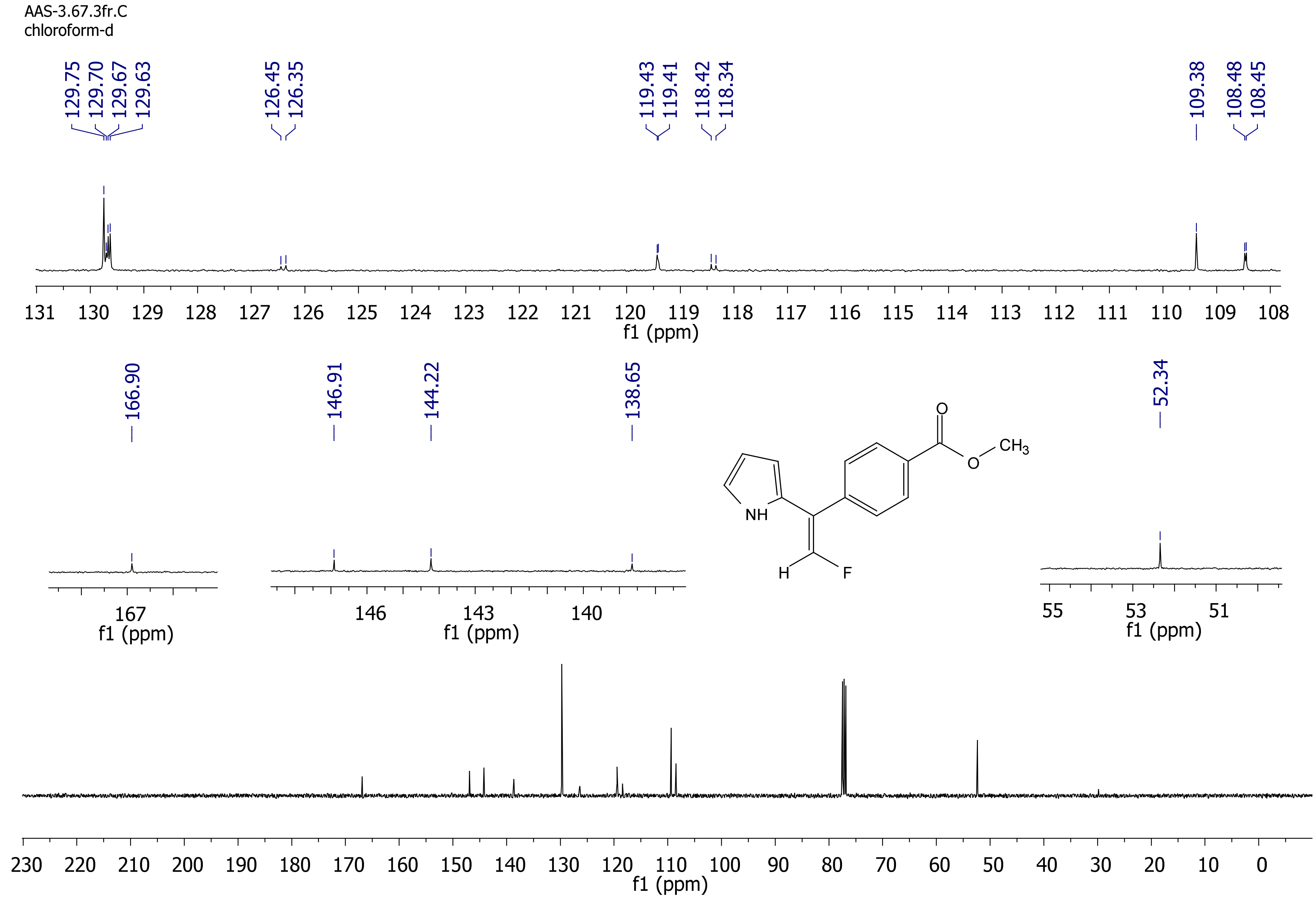

^{13}C NMR spectrum of (*E*)-methyl 4-(2-fluoro-1-(1*H*-pyrrol-2-yl)vinyl)benzoate (*E*-**4j**)

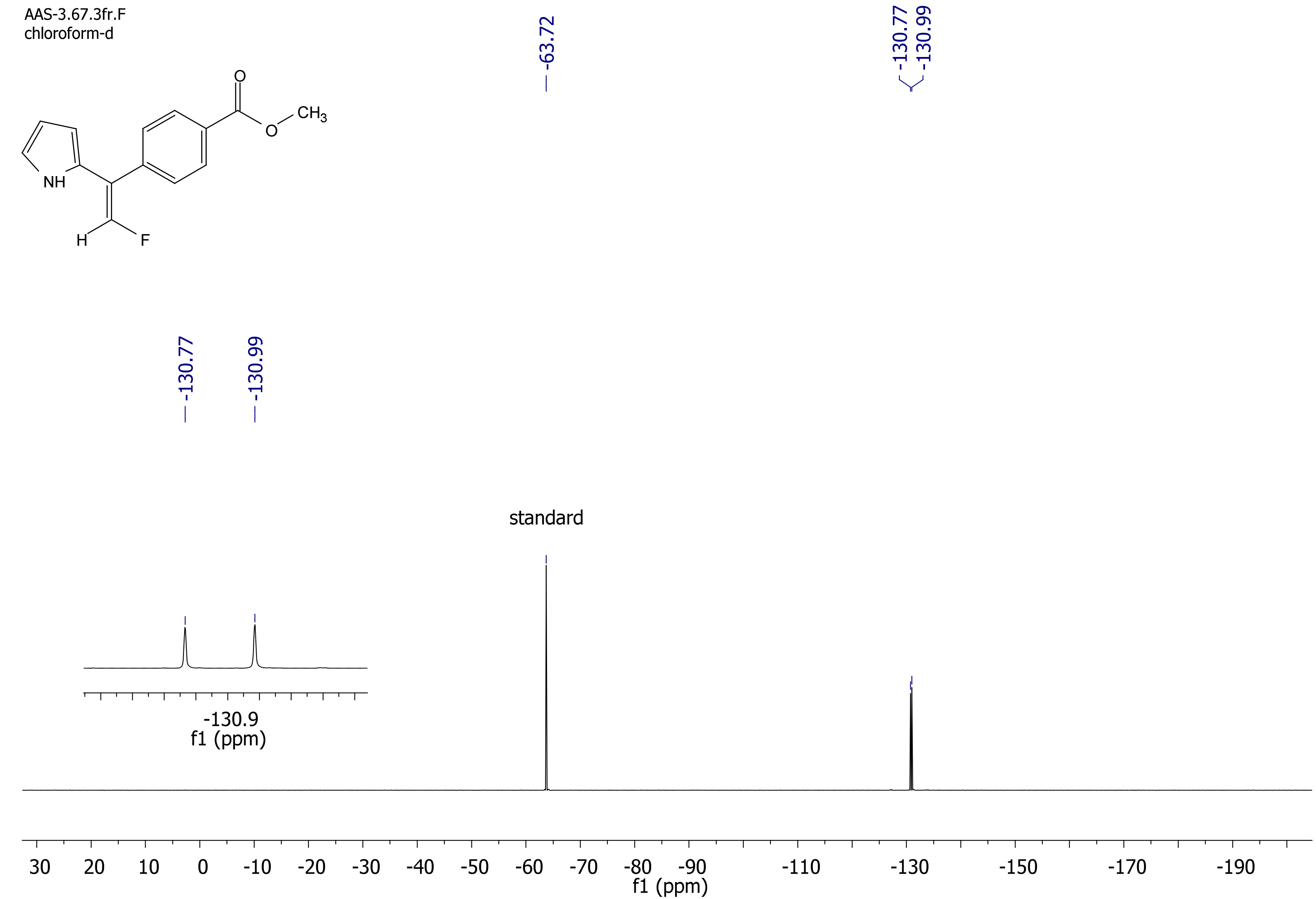

^{19}F NMR spectrum of (*E*)-methyl 4-(2-fluoro-1-(1*H*-pyrrol-2-yl)vinyl)benzoate (*E*-**4j**)

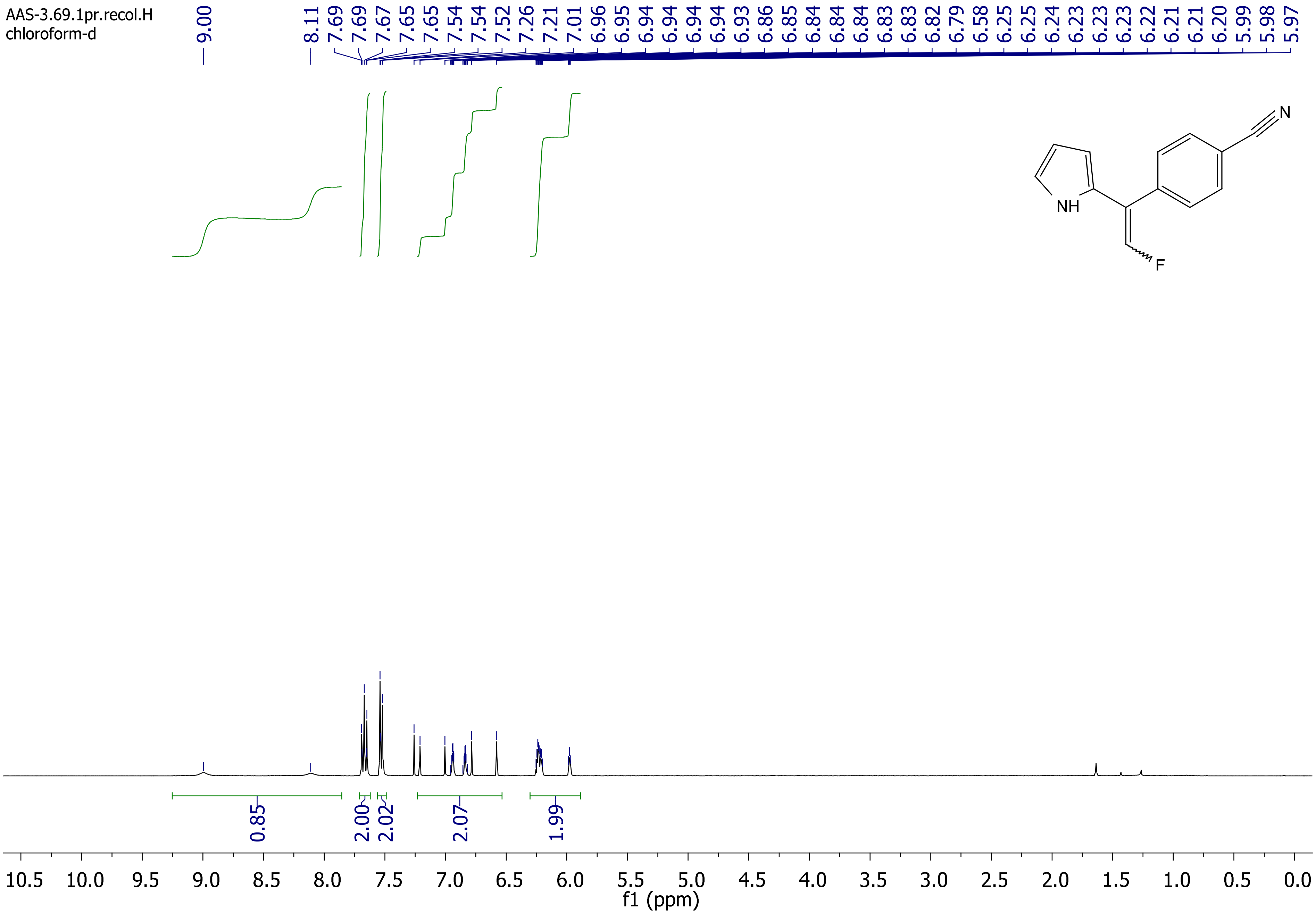

^{1}H NMR spectrum of 4-(2-fluoro-1-(1*H*-pyrrol-2-yl)vinyl)benzonitrile (**4k**)

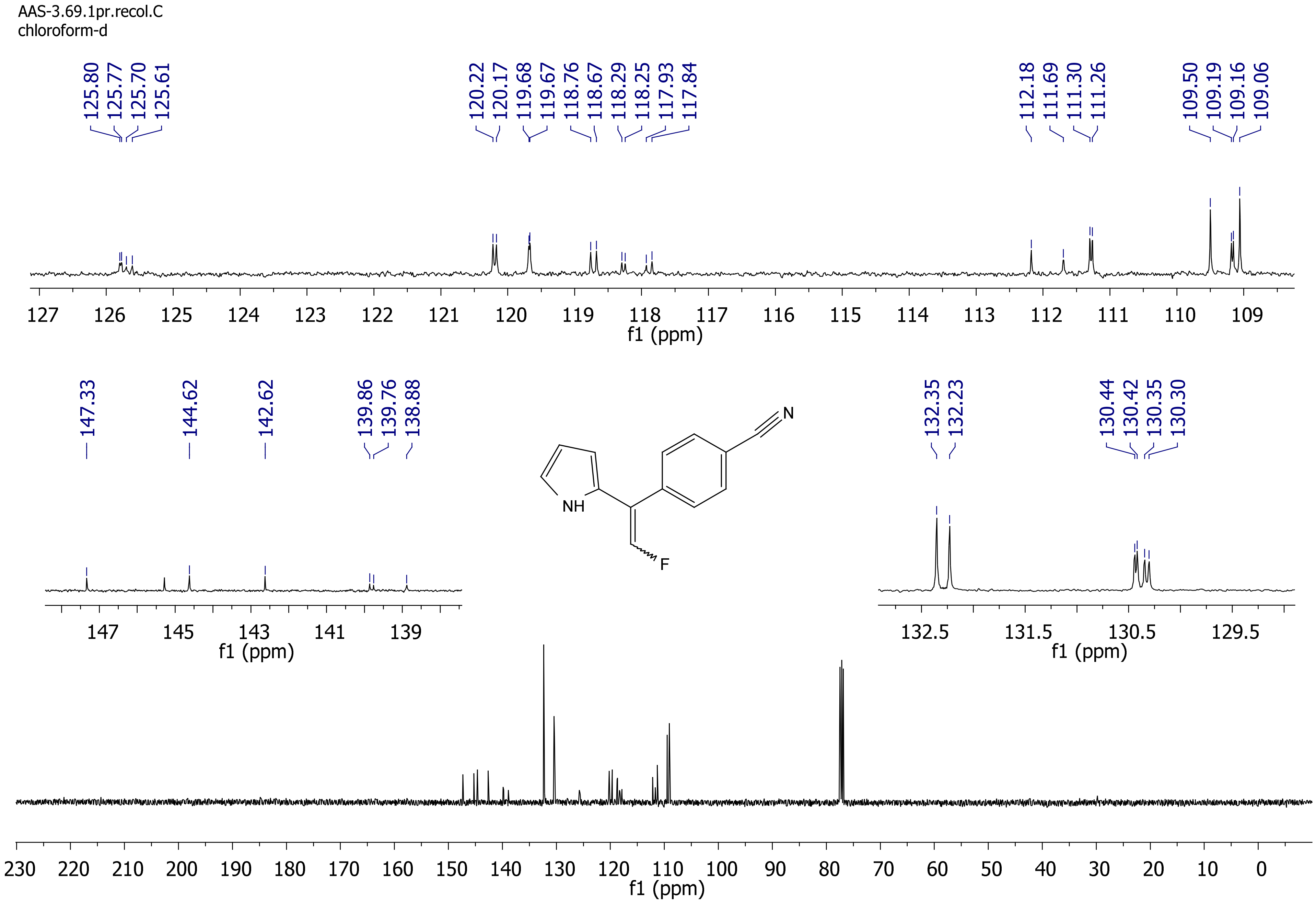

^{13}C NMR spectrum of 4-(2-fluoro-1-(1*H*-pyrrol-2-yl)vinyl)benzonitrile (**4k**)

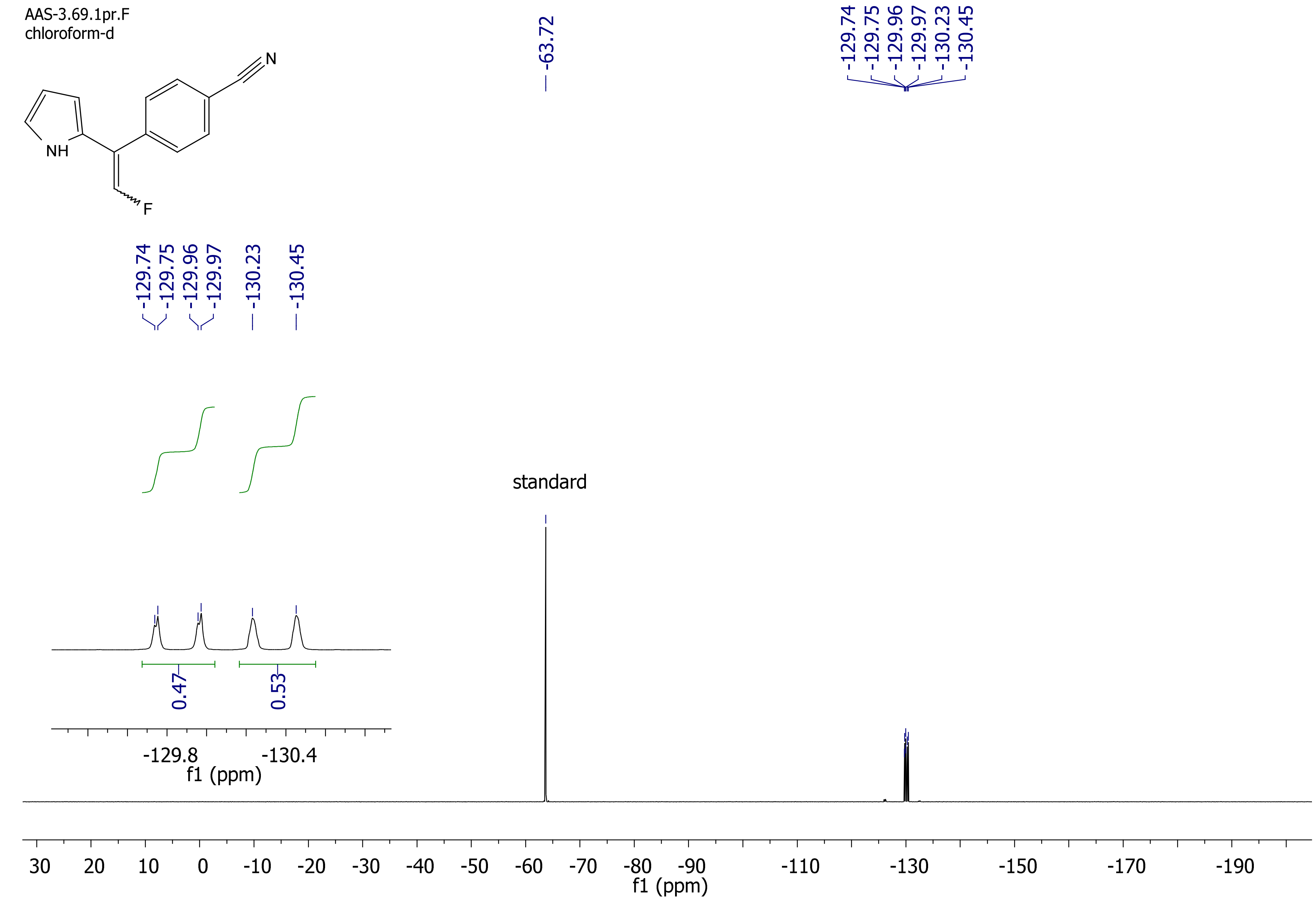

^{19}F NMR spectrum of 4-(2-fluoro-1-(1*H*-pyrrol-2-yl)vinyl)benzonitrile (**4k**)

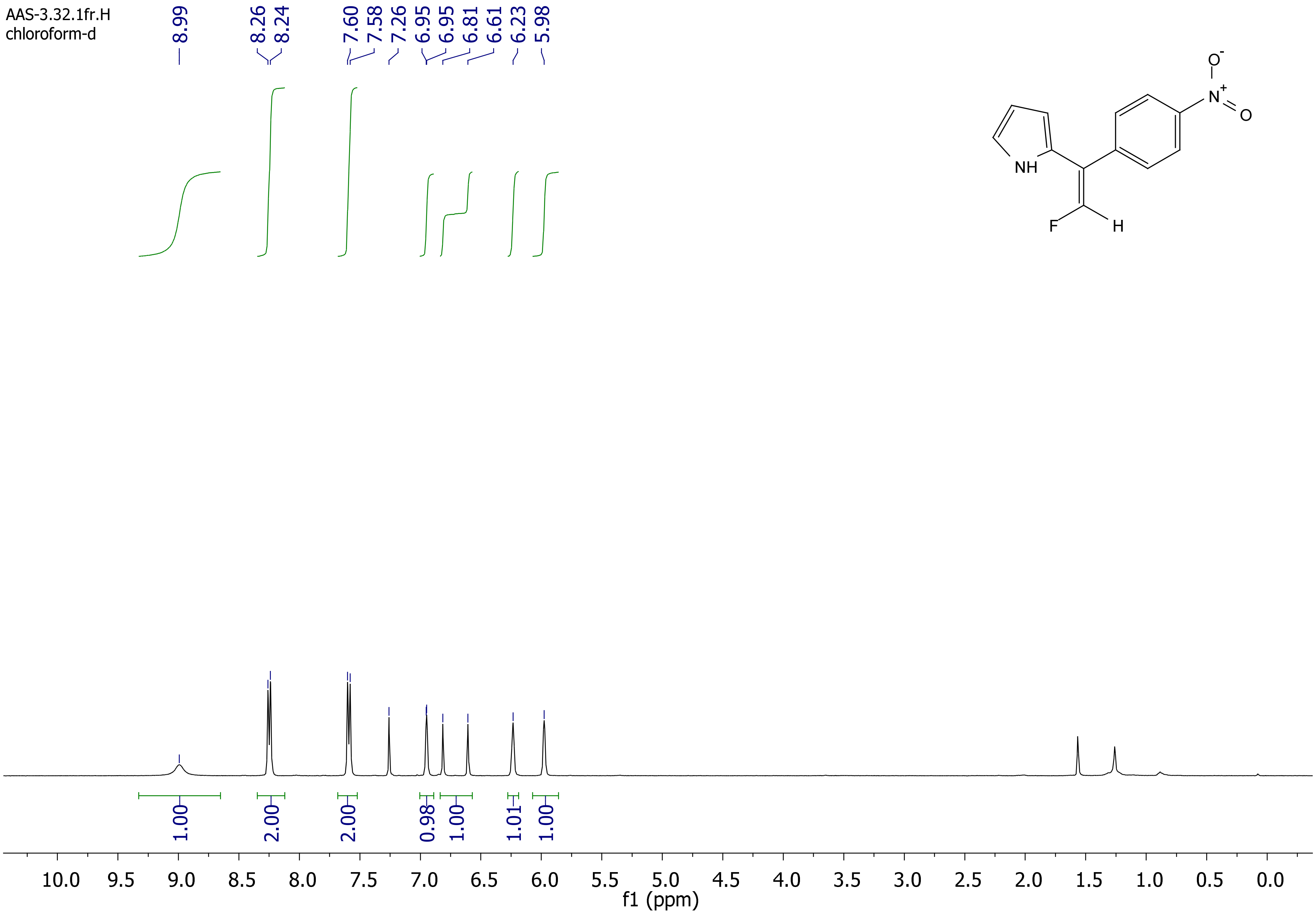

^{1}H NMR spectrum of (*Z*)-2-(2-fluoro-1-(4-nitrophenyl)vinyl)-1*H*-pyrrole (*Z*-**4l**)

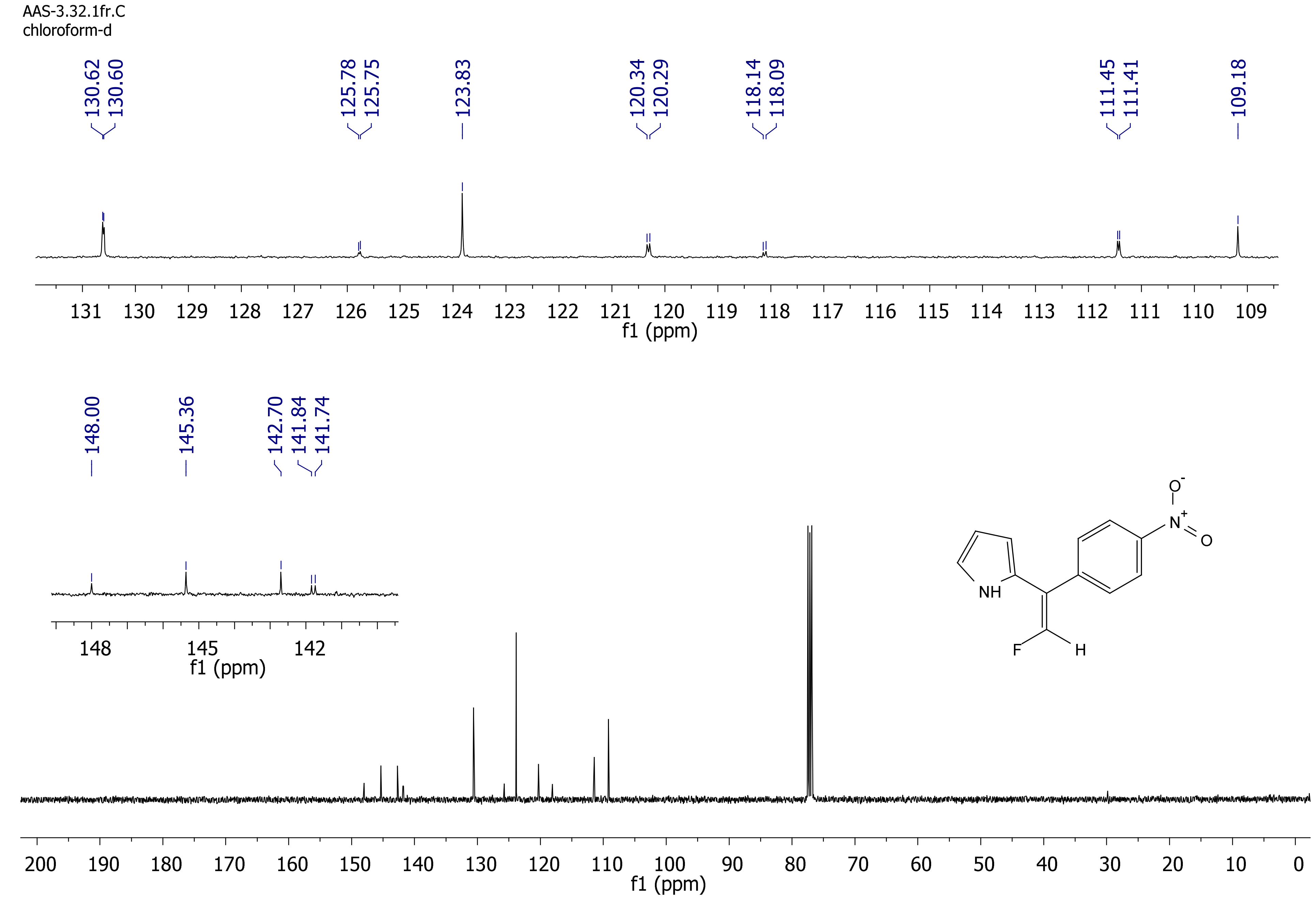

^{13}C NMR spectrum of (*Z*)-2-(2-fluoro-1-(4-nitrophenyl)vinyl)-1*H*-pyrrole (*Z*-**4l**)

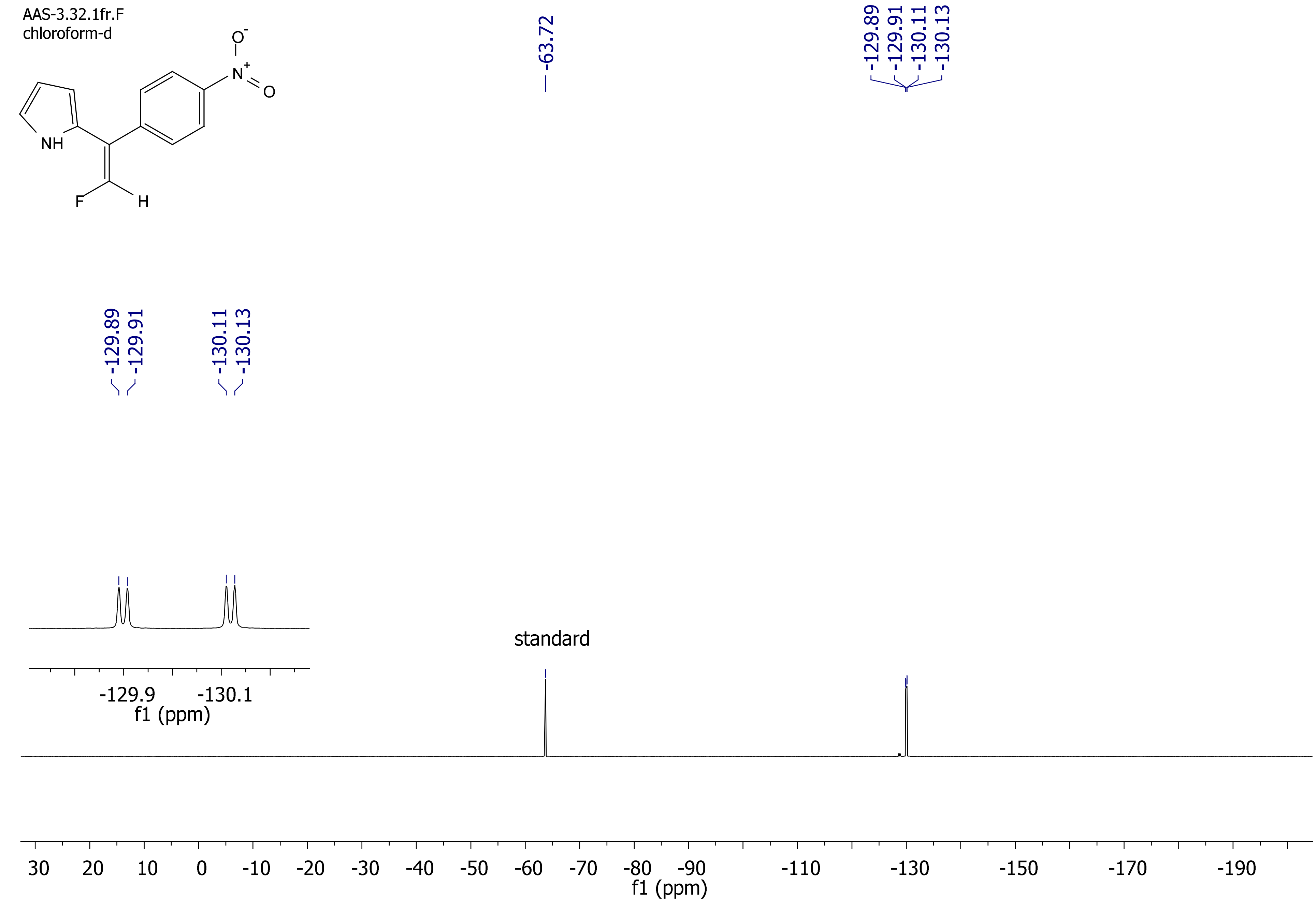

^{19}F NMR spectrum of (*Z*)-2-(2-fluoro-1-(4-nitrophenyl)vinyl)-1*H*-pyrrole (*Z*-**4l**)

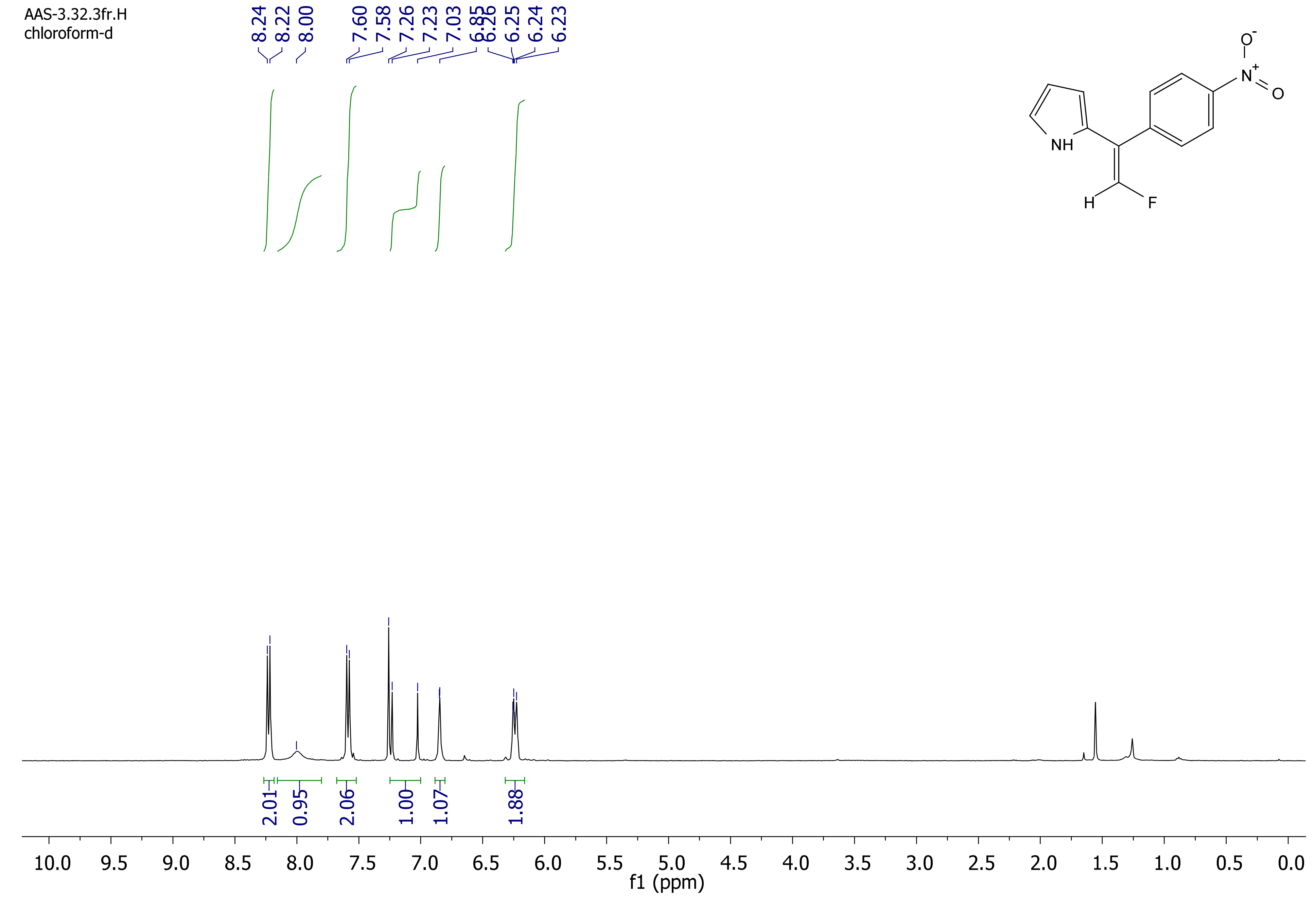

^{1}H NMR spectrum of (*E*)-2-(2-fluoro-1-(4-nitrophenyl)vinyl)-1*H*-pyrrole (*E*-**4l**)

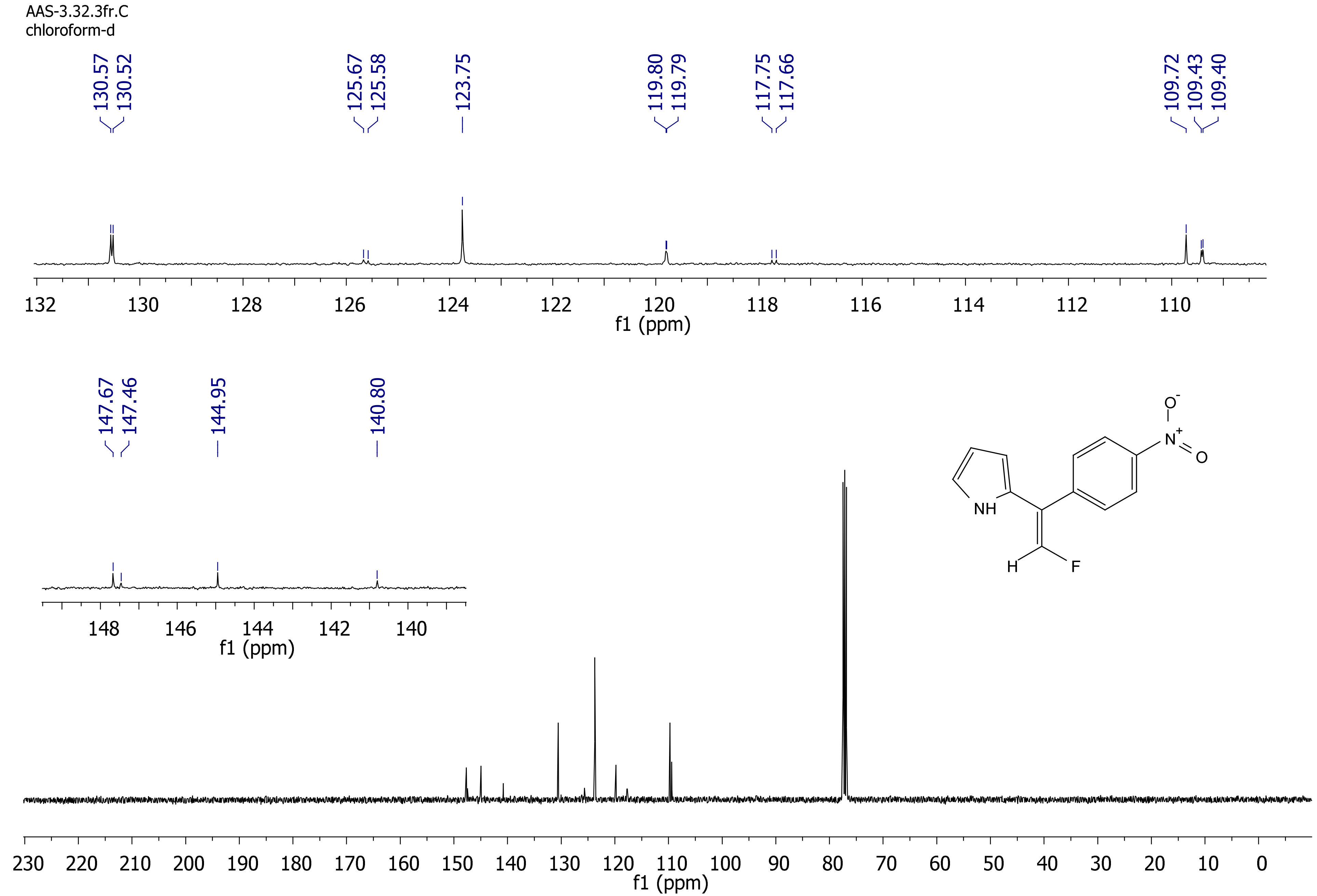

^{13}C NMR spectrum of (*E*)-2-(2-fluoro-1-(4-nitrophenyl)vinyl)-1*H*-pyrrole (*E*-**4l**)

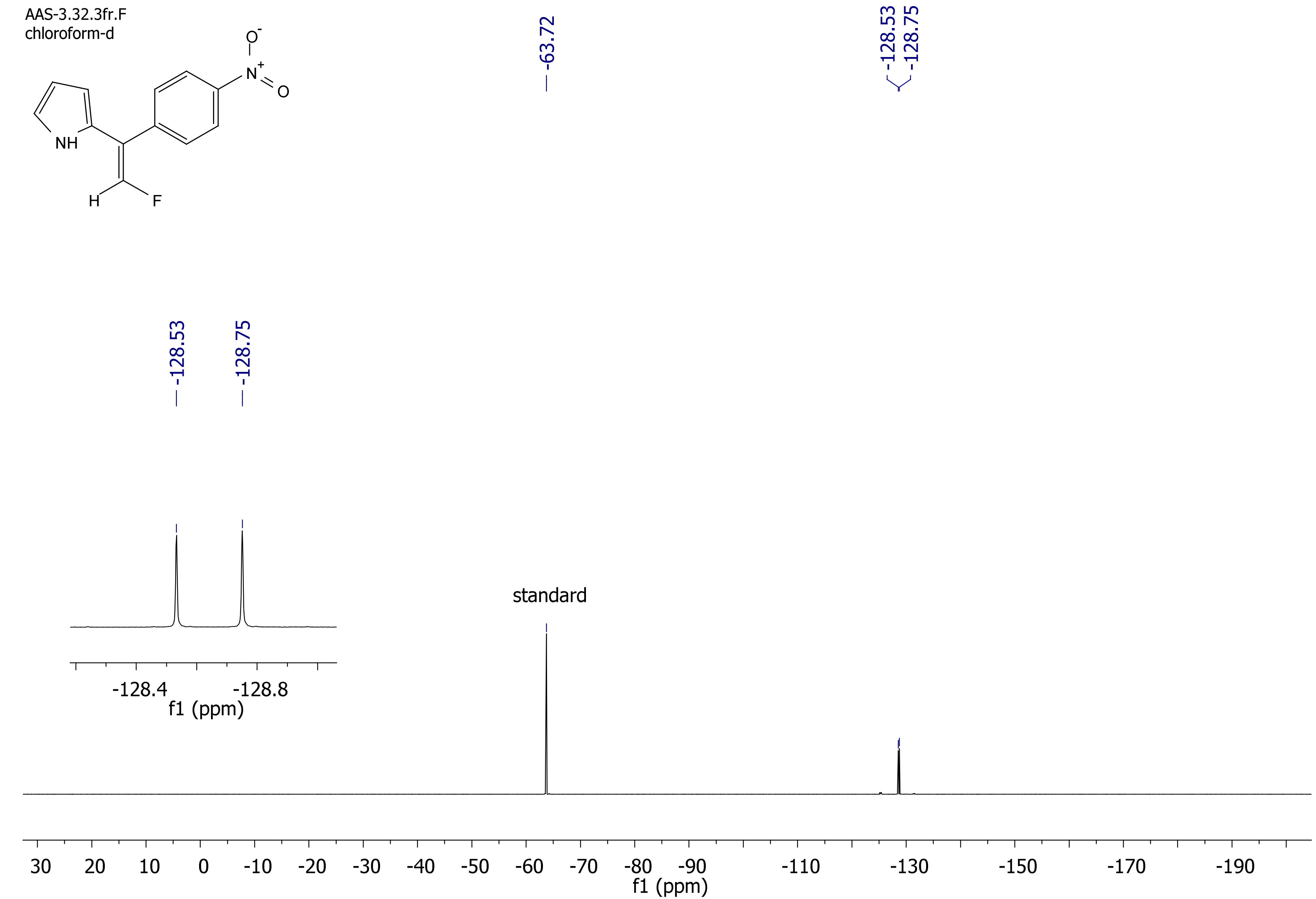

^{19}F NMR spectrum of (*E*)-2-(2-fluoro-1-(4-nitrophenyl)vinyl)-1*H*-pyrrole (*E*-**4l**)

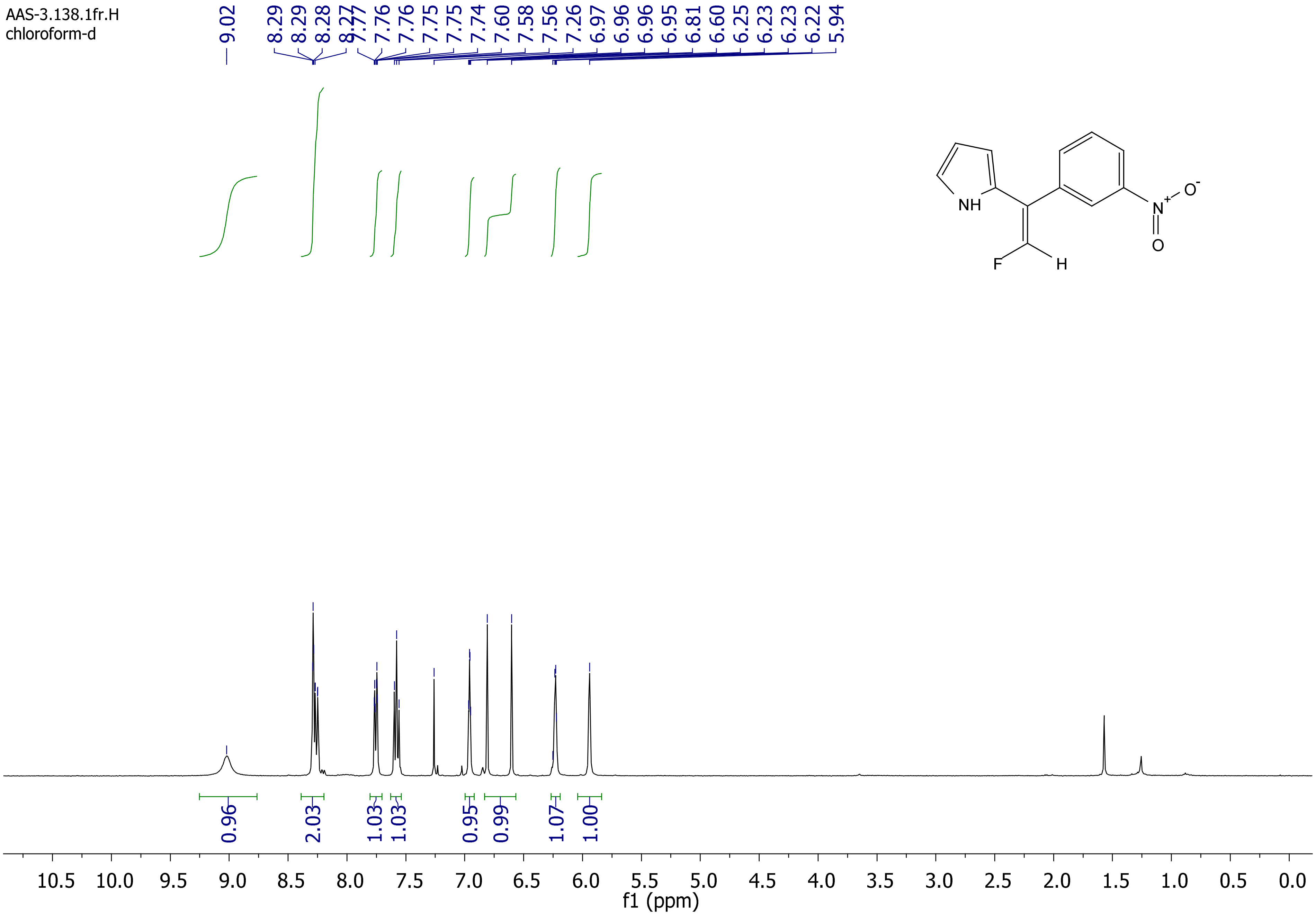

^{1}H NMR spectrum of (*Z*)-2-(2-fluoro-1-(3-nitrophenyl)vinyl)-1*H*-pyrrole (*Z*-**4m**)

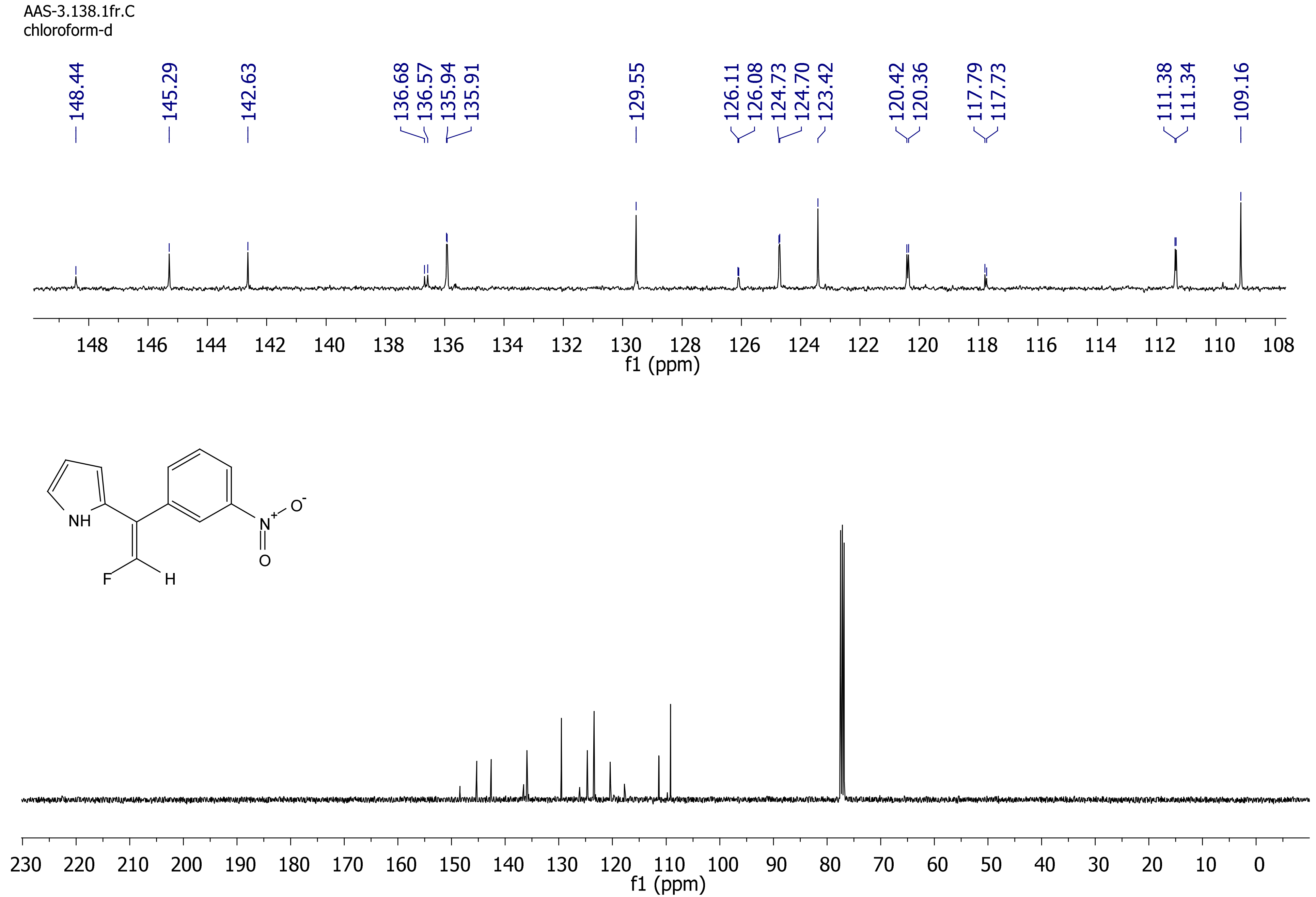

^{13}C NMR spectrum of (*Z*)-2-(2-fluoro-1-(3-nitrophenyl)vinyl)-1*H*-pyrrole (*Z*-**4m**)

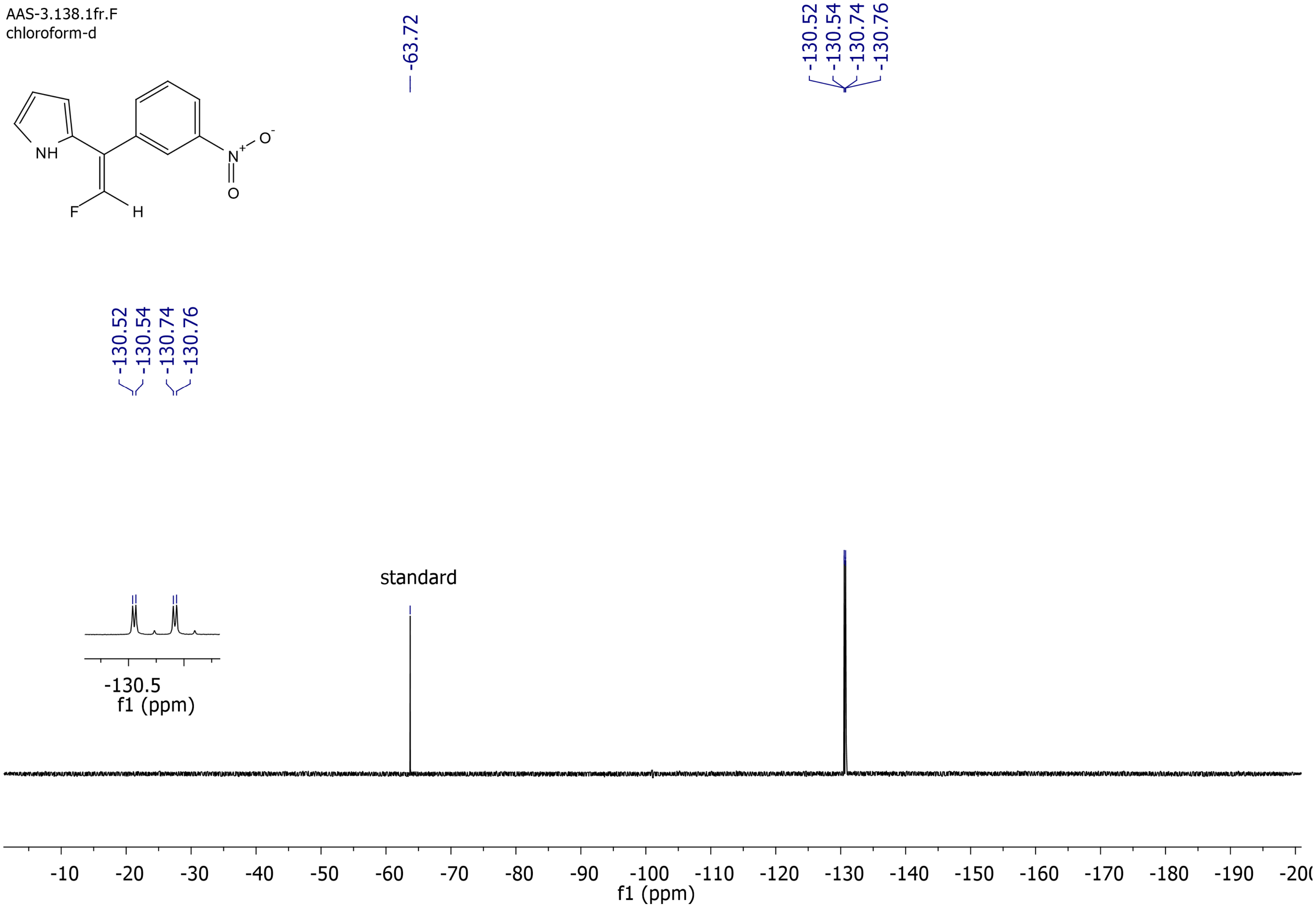

^{19}F NMR spectrum of (*Z*)-2-(2-fluoro-1-(3-nitrophenyl)vinyl)-1*H*-pyrrole (*Z*-**4m**)

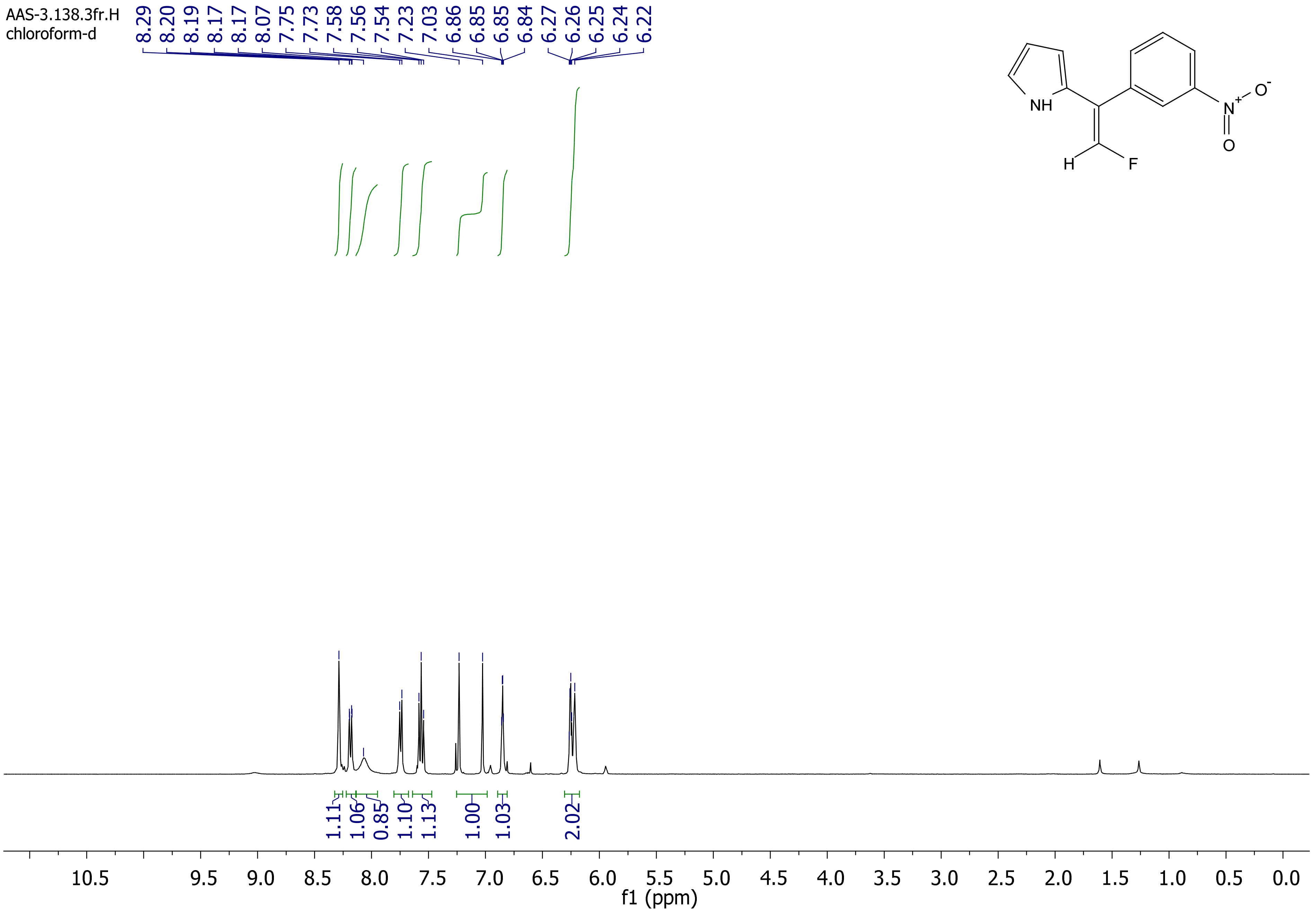

^{1}H NMR spectrum of (*E*)-2-(2-fluoro-1-(3-nitrophenyl)vinyl)-1*H*-pyrrole (*E*-**4m**)

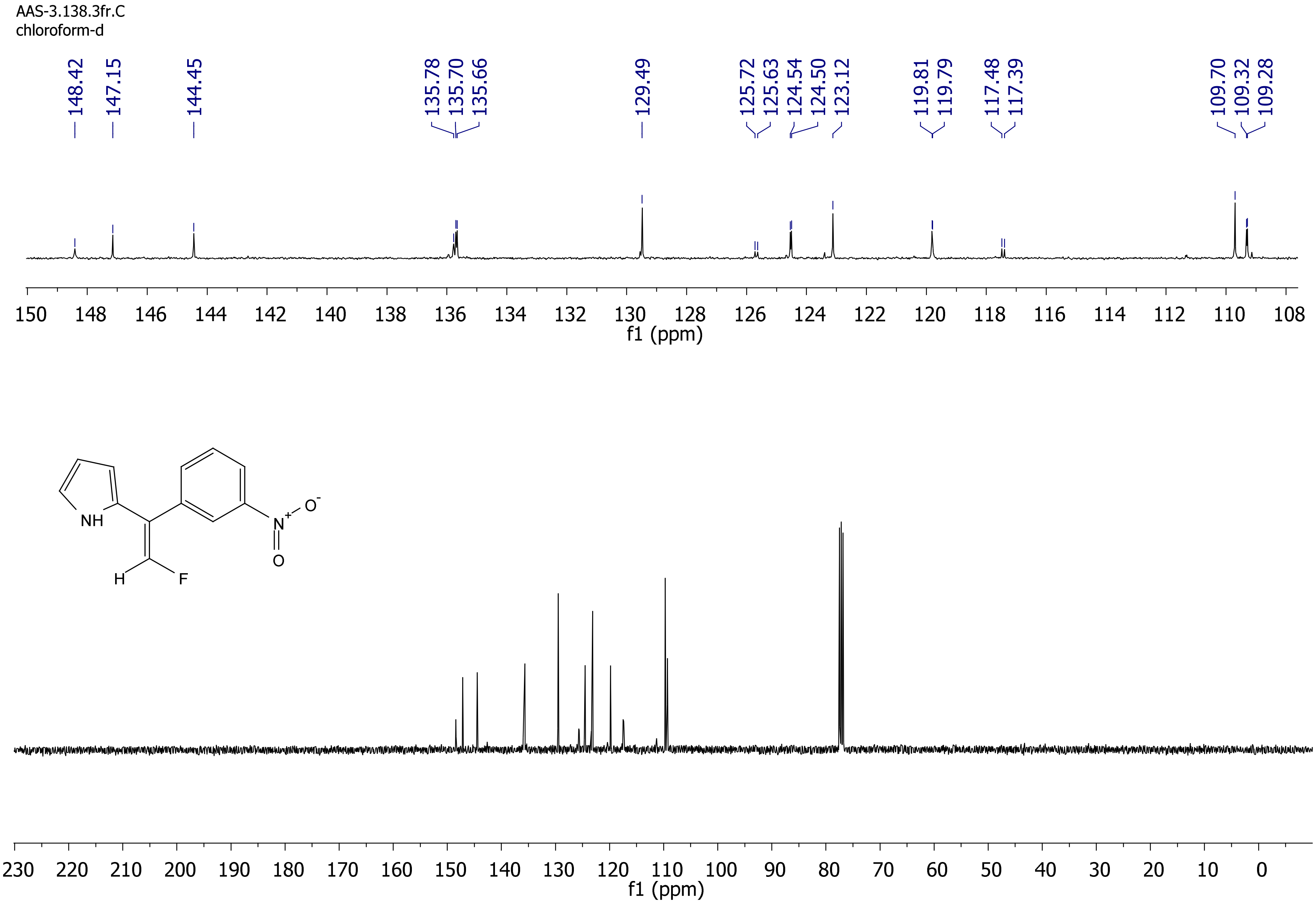

^{13}C NMR spectrum of (*E*)-2-(2-fluoro-1-(3-nitrophenyl)vinyl)-1*H*-pyrrole (*E*-**4m**)

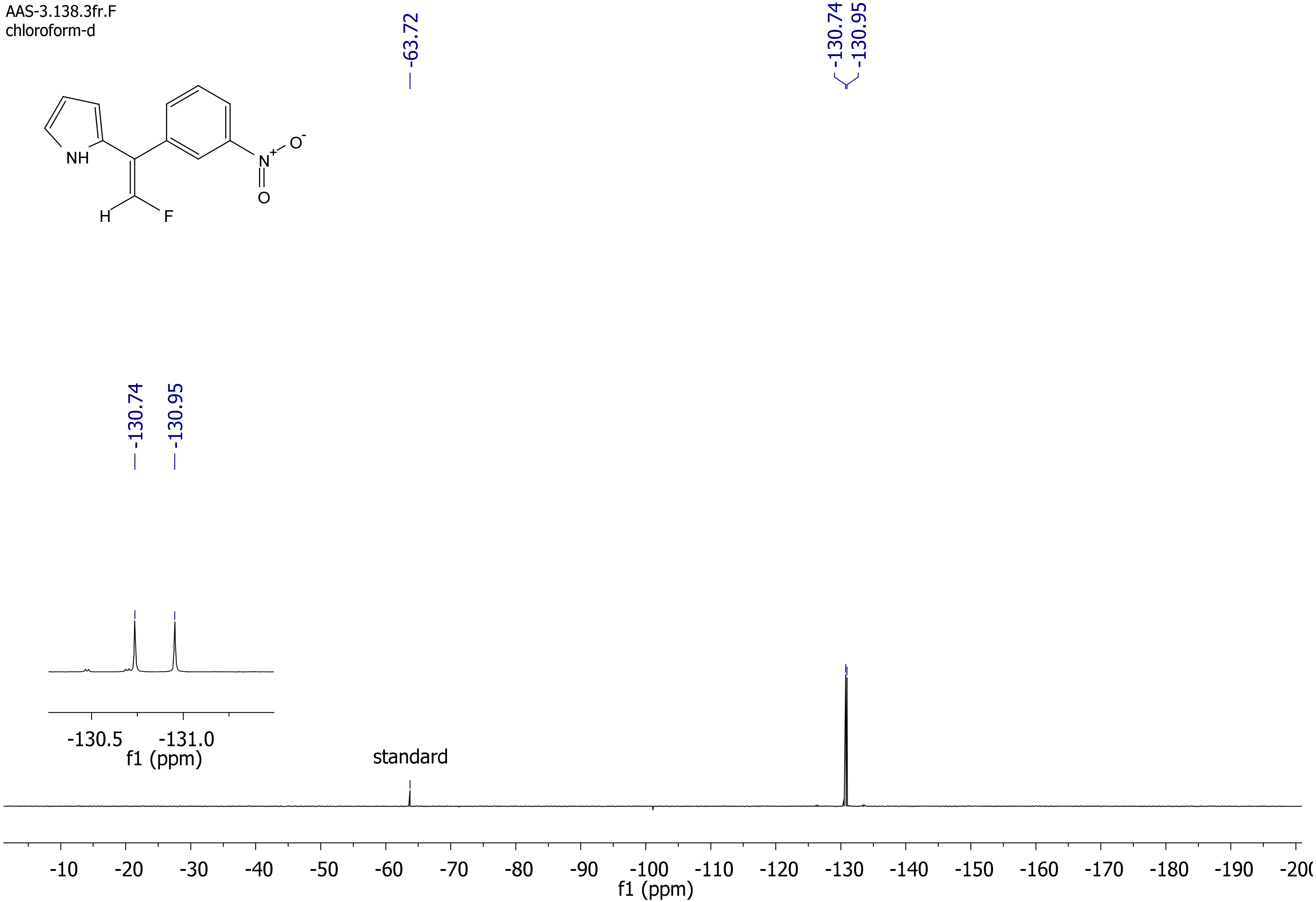

^{19}F NMR spectrum of (*E*)-2-(2-fluoro-1-(3-nitrophenyl)vinyl)-1*H*-pyrrole (*E*-**4m**)

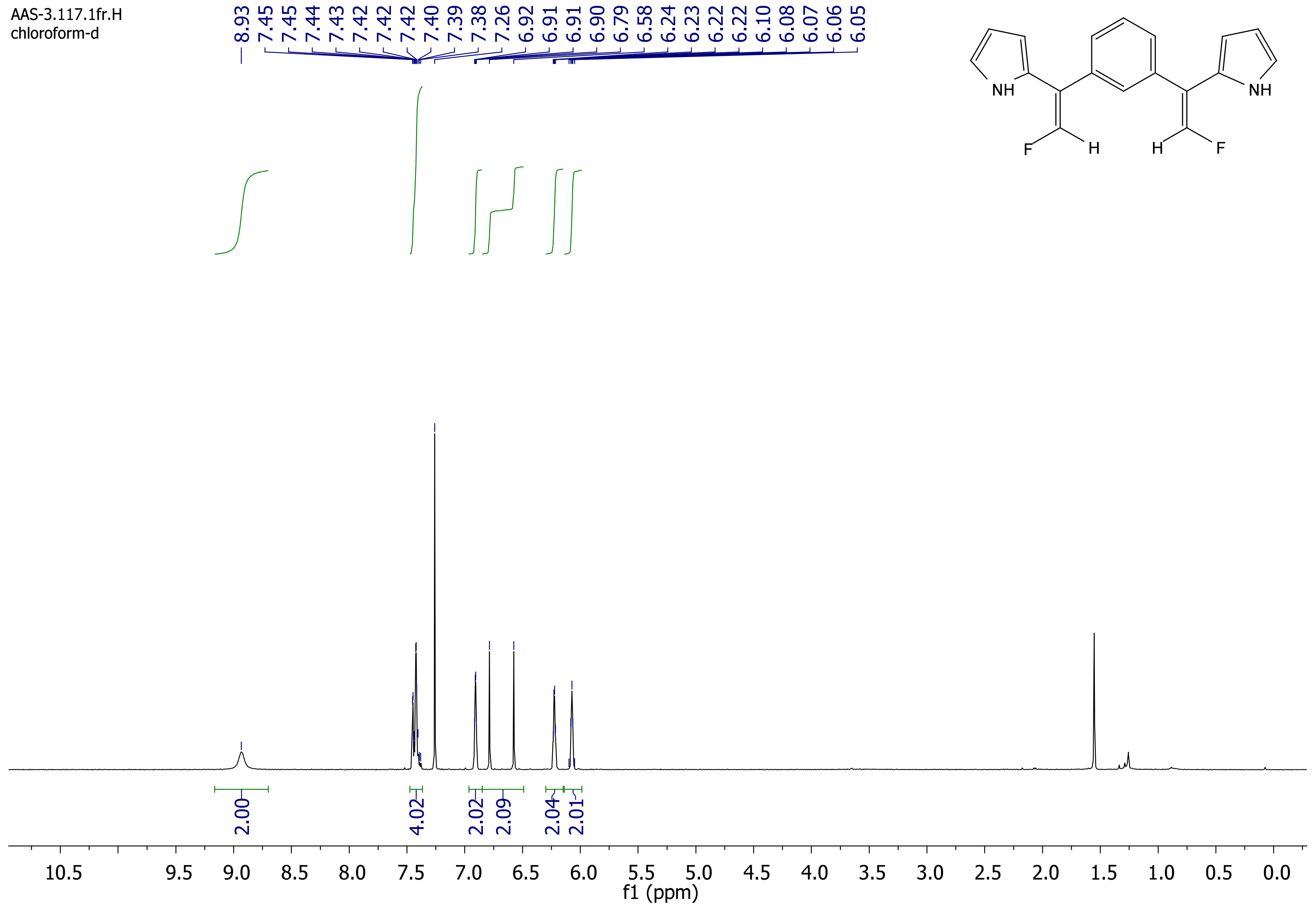

^{1}H NMR spectrum of 1,3-bis((*Z*)-2-fluoro-1-(1*H*-pyrrol-2-yl)vinyl)benzene (*Z*,*Z*-**4o**)

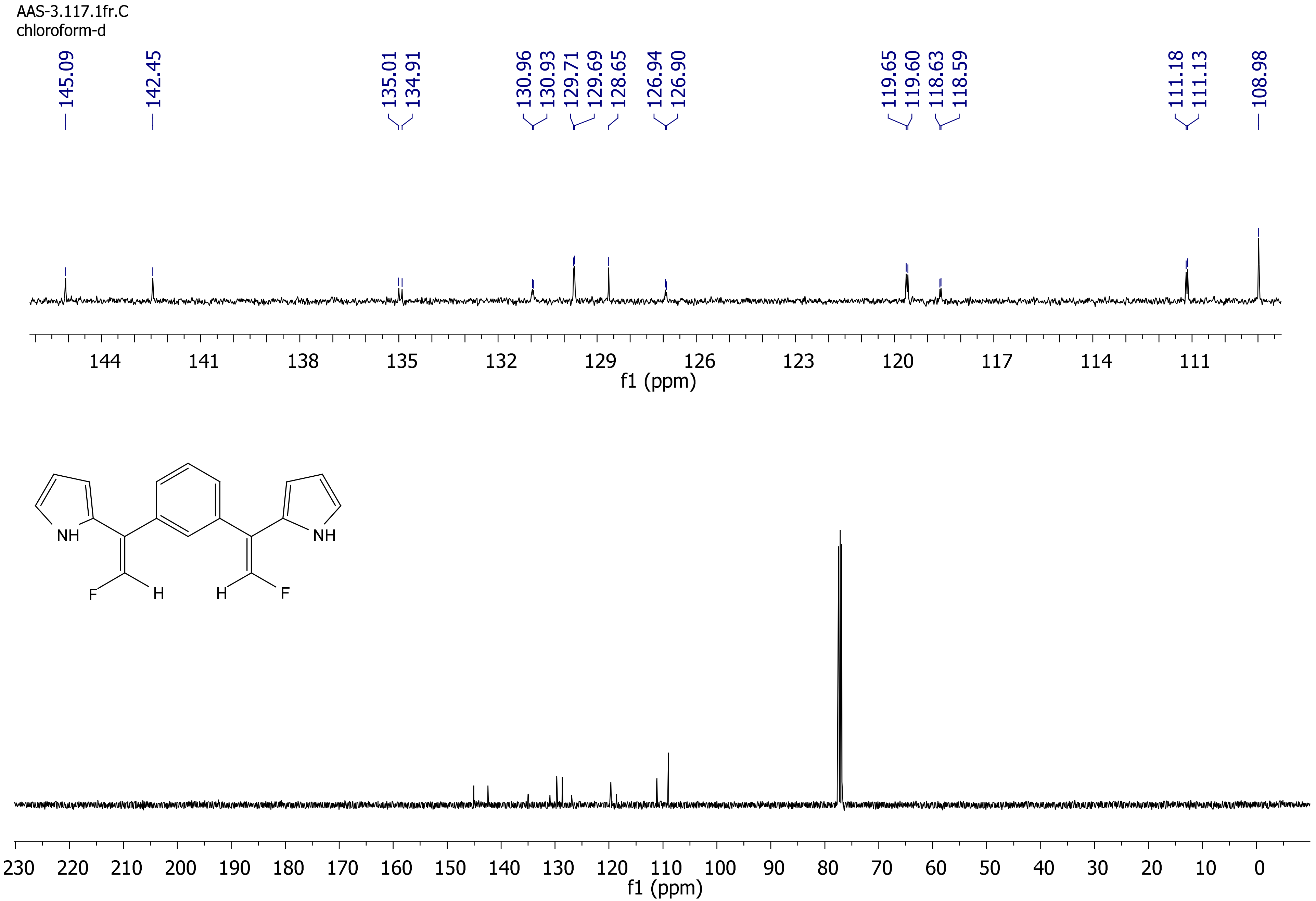

^{13}C NMR spectrum of 1,3-bis((*Z*)-2-fluoro-1-(1*H*-pyrrol-2-yl)vinyl)benzene (*Z*,*Z*-**4o**)

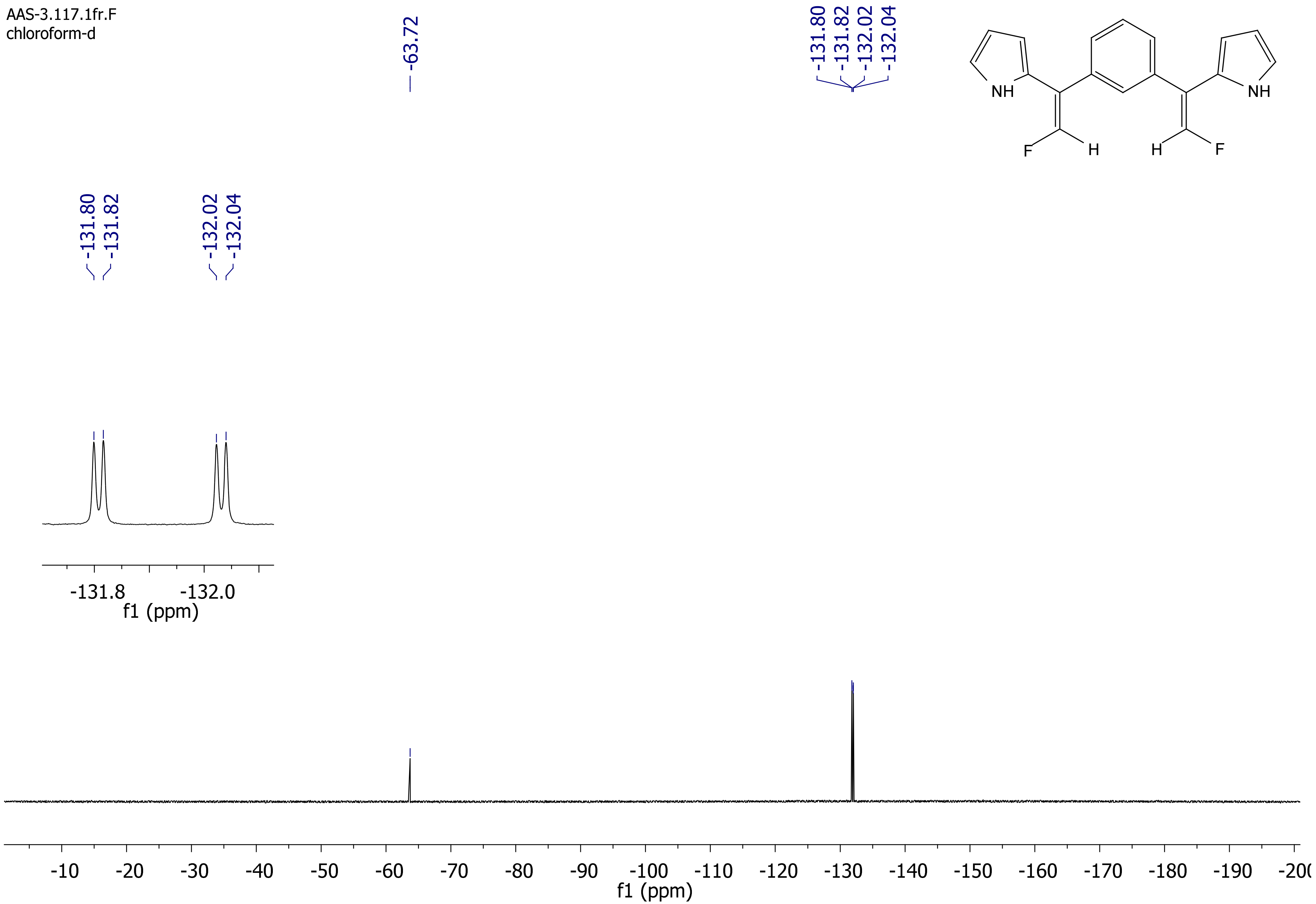

^{19}F NMR spectrum of 1,3-bis((*Z*)-2-fluoro-1-(1*H*-pyrrol-2-yl)vinyl)benzene (*Z*,*Z*-**4o**)

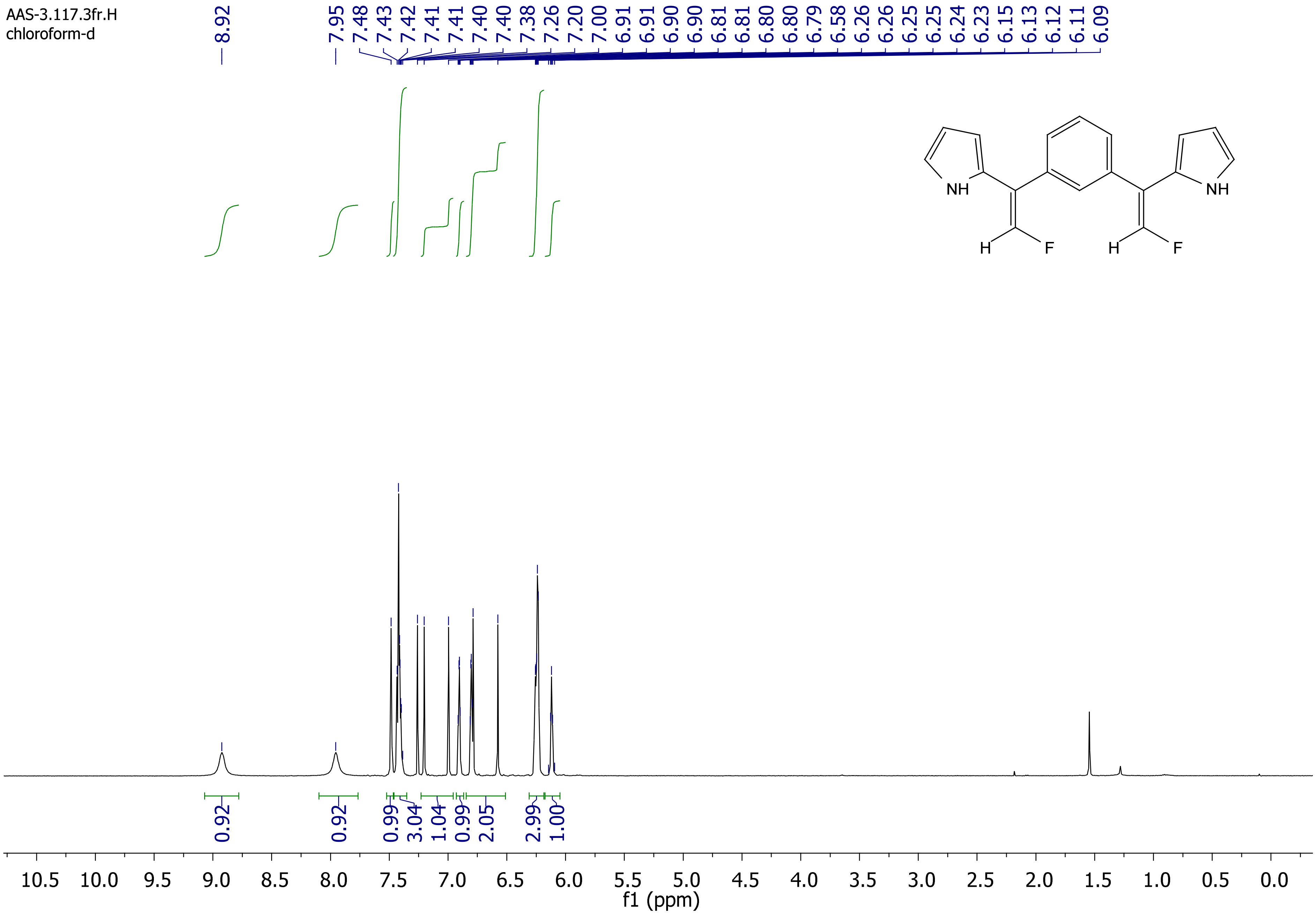

^{1}H NMR spectrum of 1-((*E*)-2-fluoro-1-(1*H*-pyrrol-2-yl)vinyl)-3-((*Z*)-2-fluoro-1-(1*H*-pyrrol-2-yl)vinyl)benzene (*E*,*Z*-**4o**)

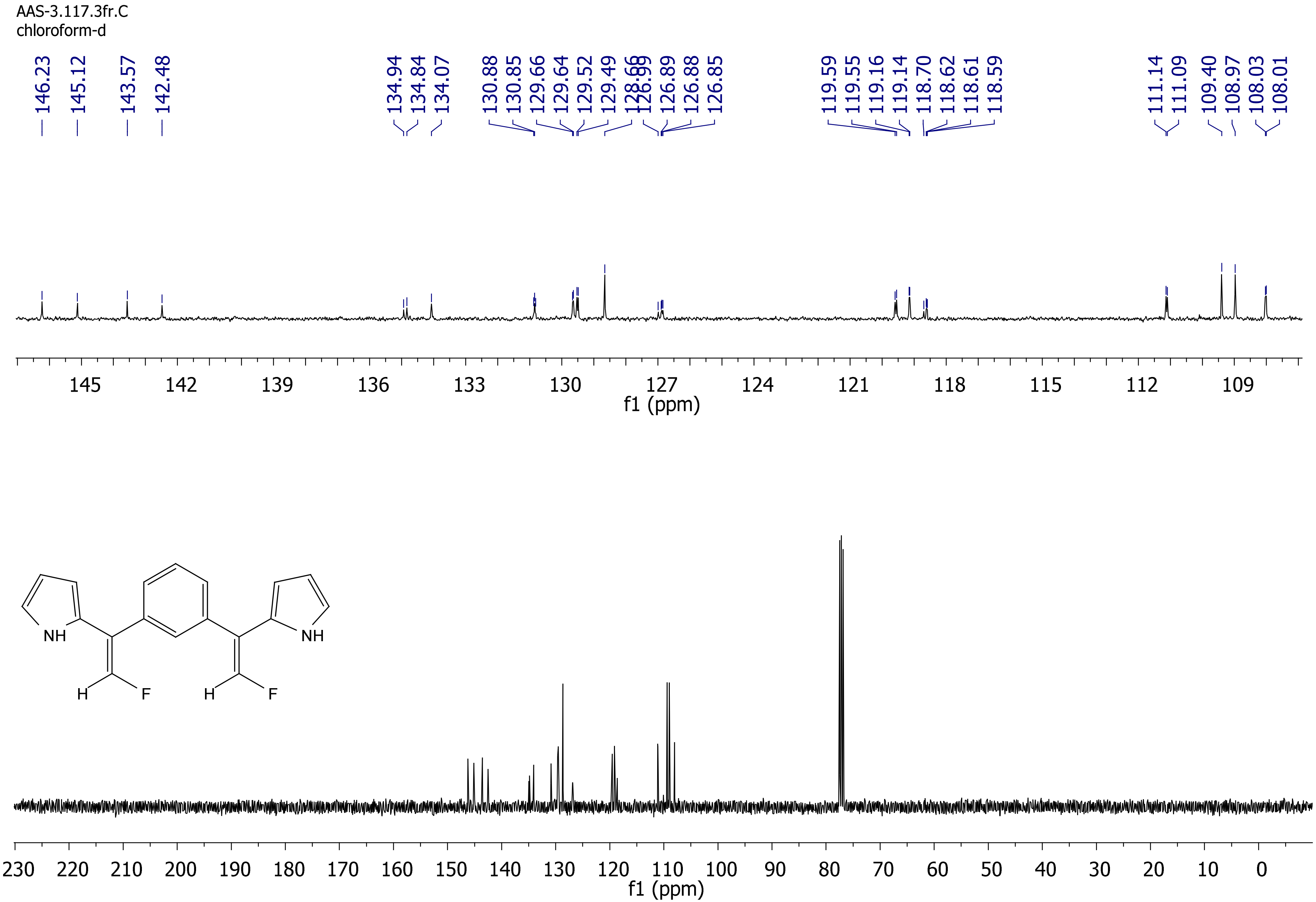

^{13}C NMR spectrum of 1-((*E*)-2-fluoro-1-(1*H*-pyrrol-2-yl)vinyl)-3-((*Z*)-2-fluoro-1-(1*H*-pyrrol-2-yl)vinyl)benzene (*E*,*Z*-**4o**)

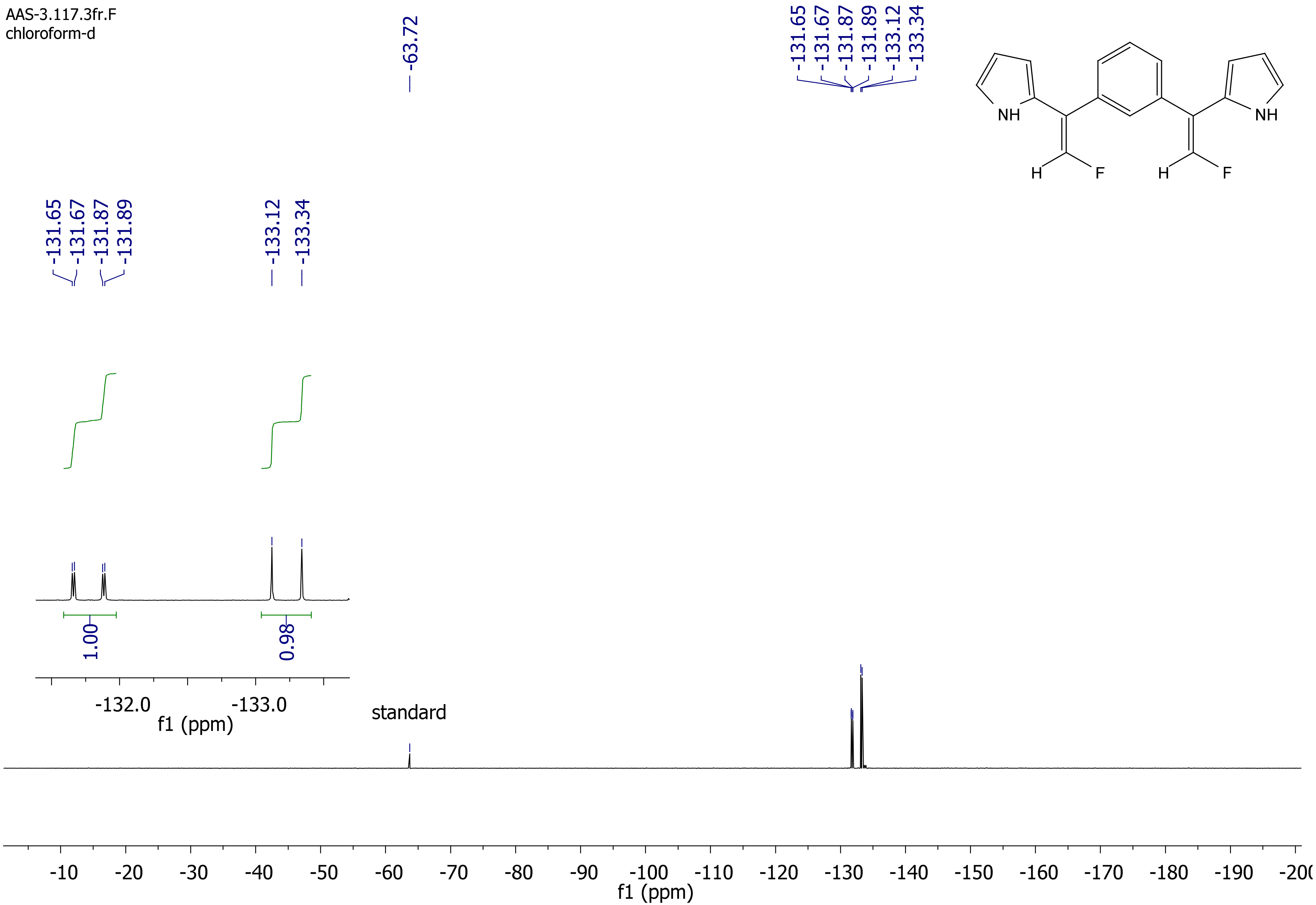

^{19}F NMR spectrum of 1-((*E*)-2-fluoro-1-(1*H*-pyrrol-2-yl)vinyl)-3-((*Z*)-2-fluoro-1-(1*H*-pyrrol-2-yl)vinyl)benzene (*E*,*Z*-**4o**)

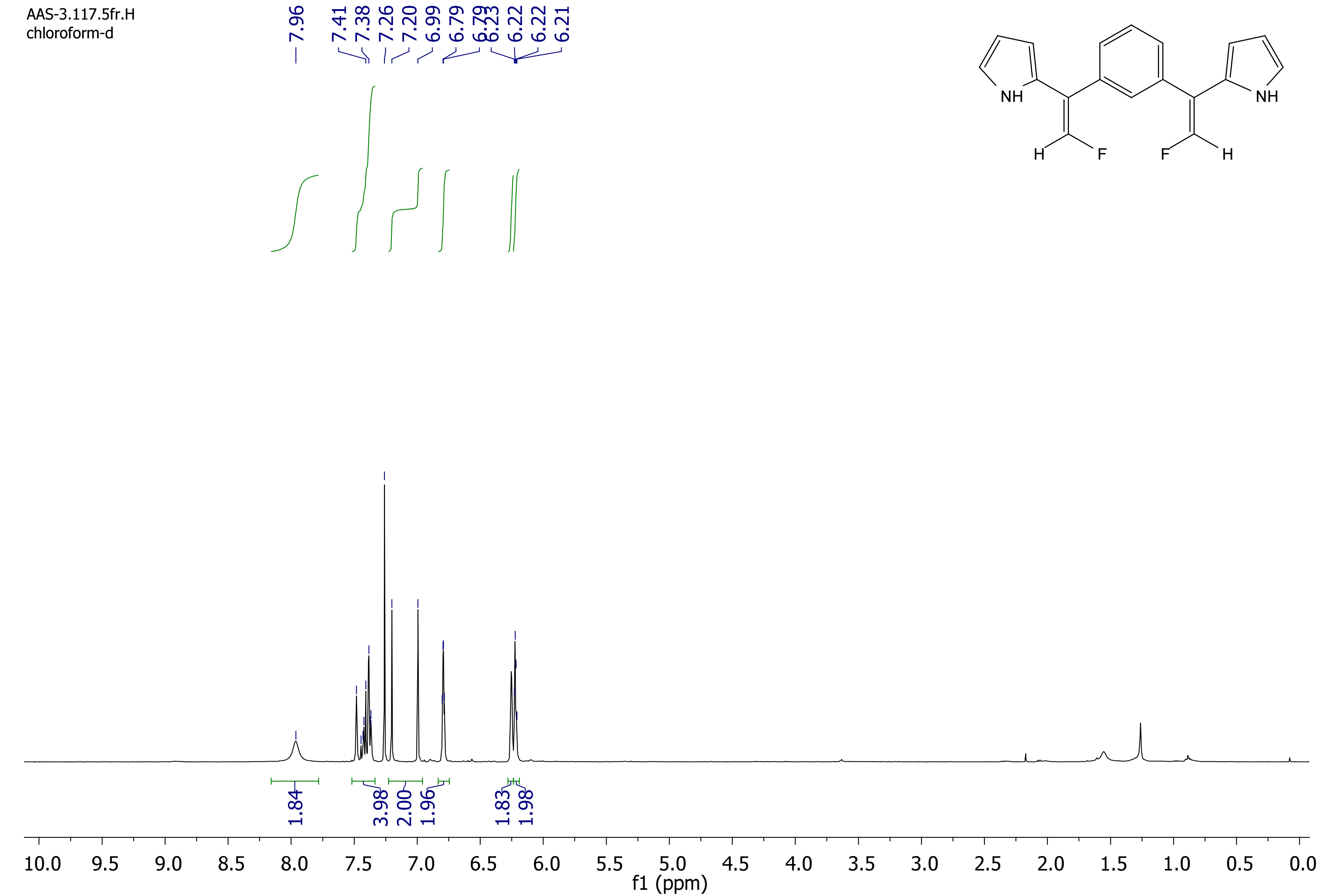

^{1}H NMR spectrum of 1,3-bis((*E*)-2-fluoro-1-(1*H*-pyrrol-2-yl)vinyl)benzene (*E*,*E*-**4o**)

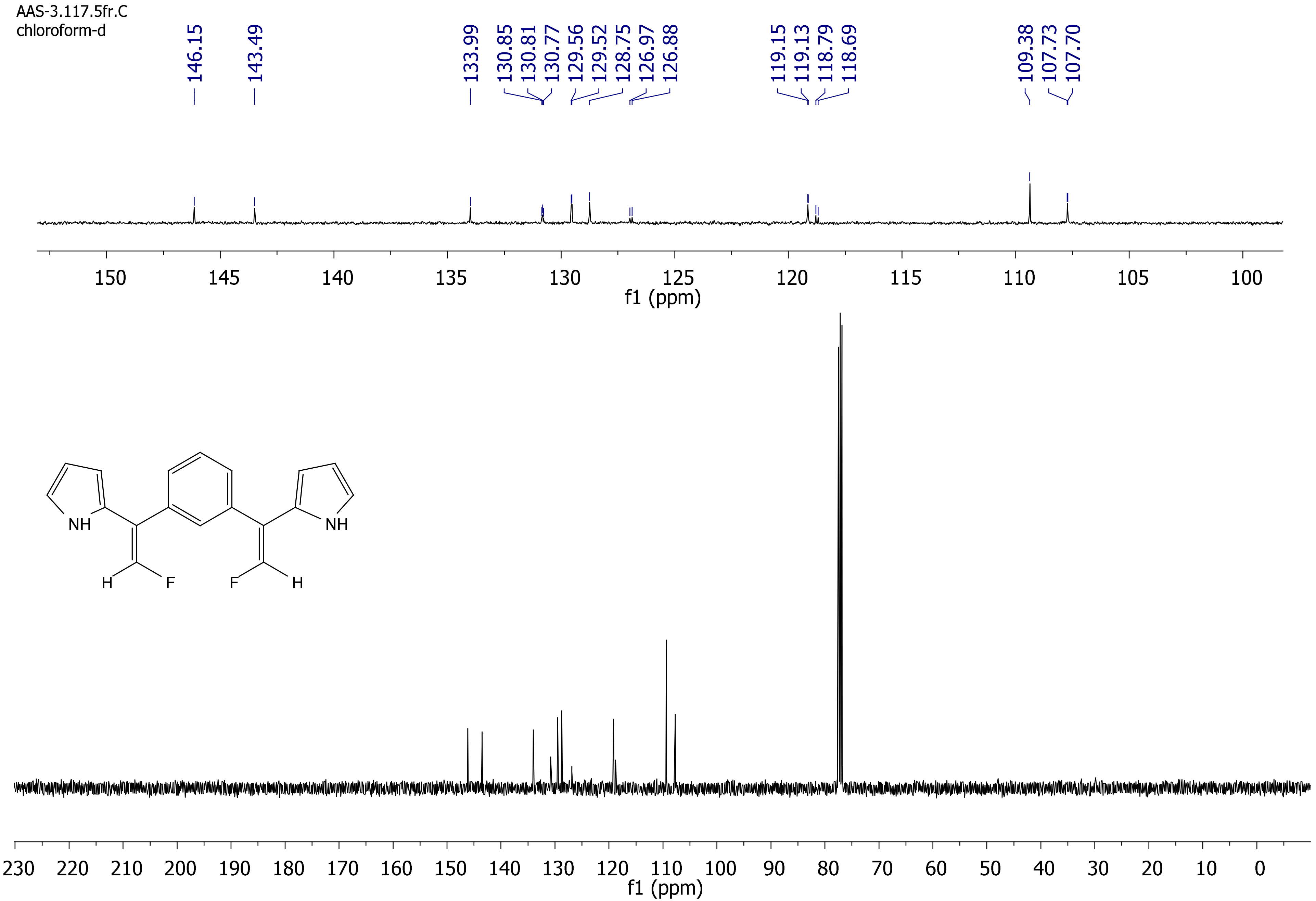

^{13}C NMR spectrum of 1,3-bis((*E*)-2-fluoro-1-(1*H*-pyrrol-2-yl)vinyl)benzene (*E*,*E*-**4o**)

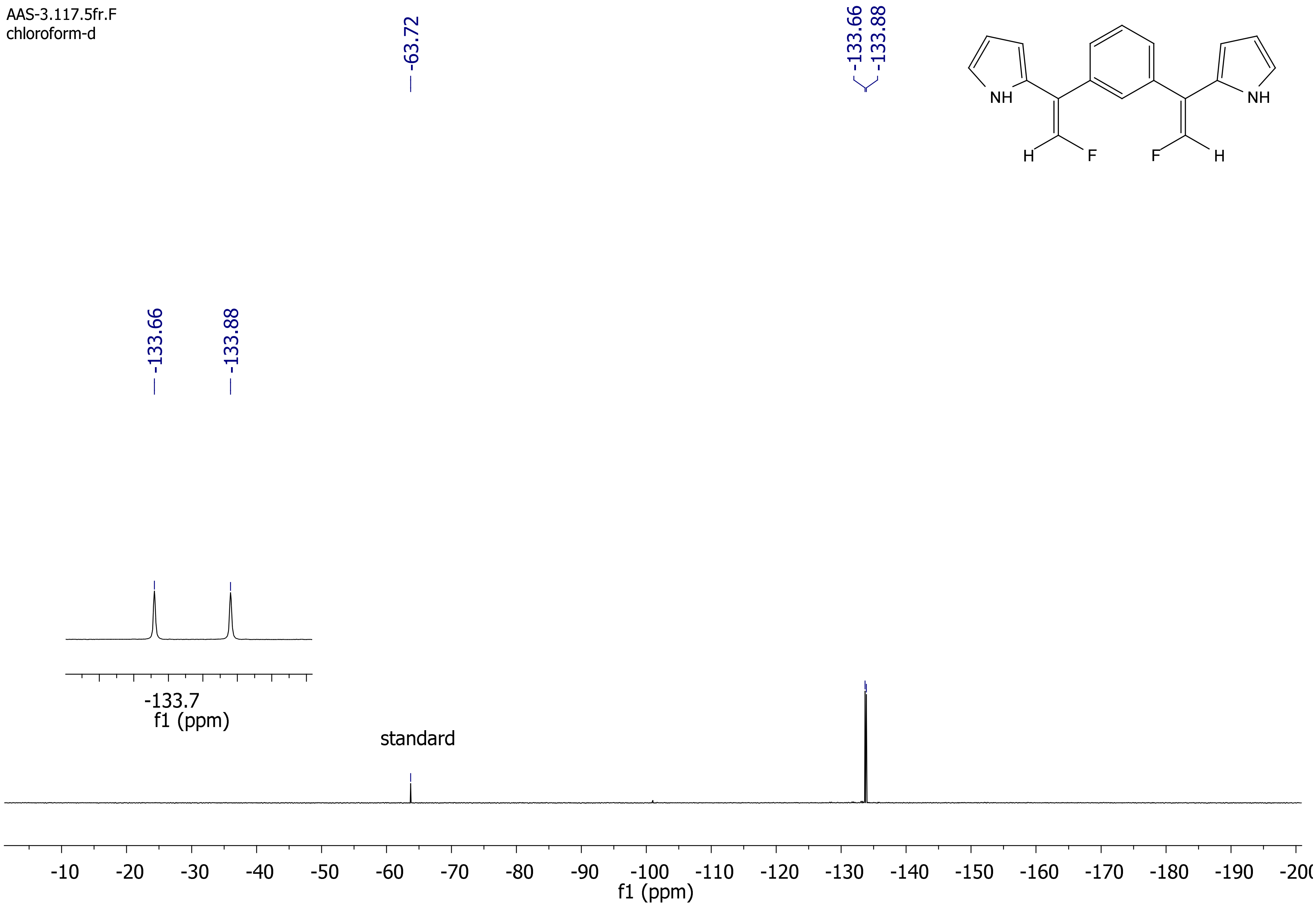

^{19}F NMR spectrum of 1,3-bis((*E*)-2-fluoro-1-(1*H*-pyrrol-2-yl)vinyl)benzene (*E*,*E*-**4o**)

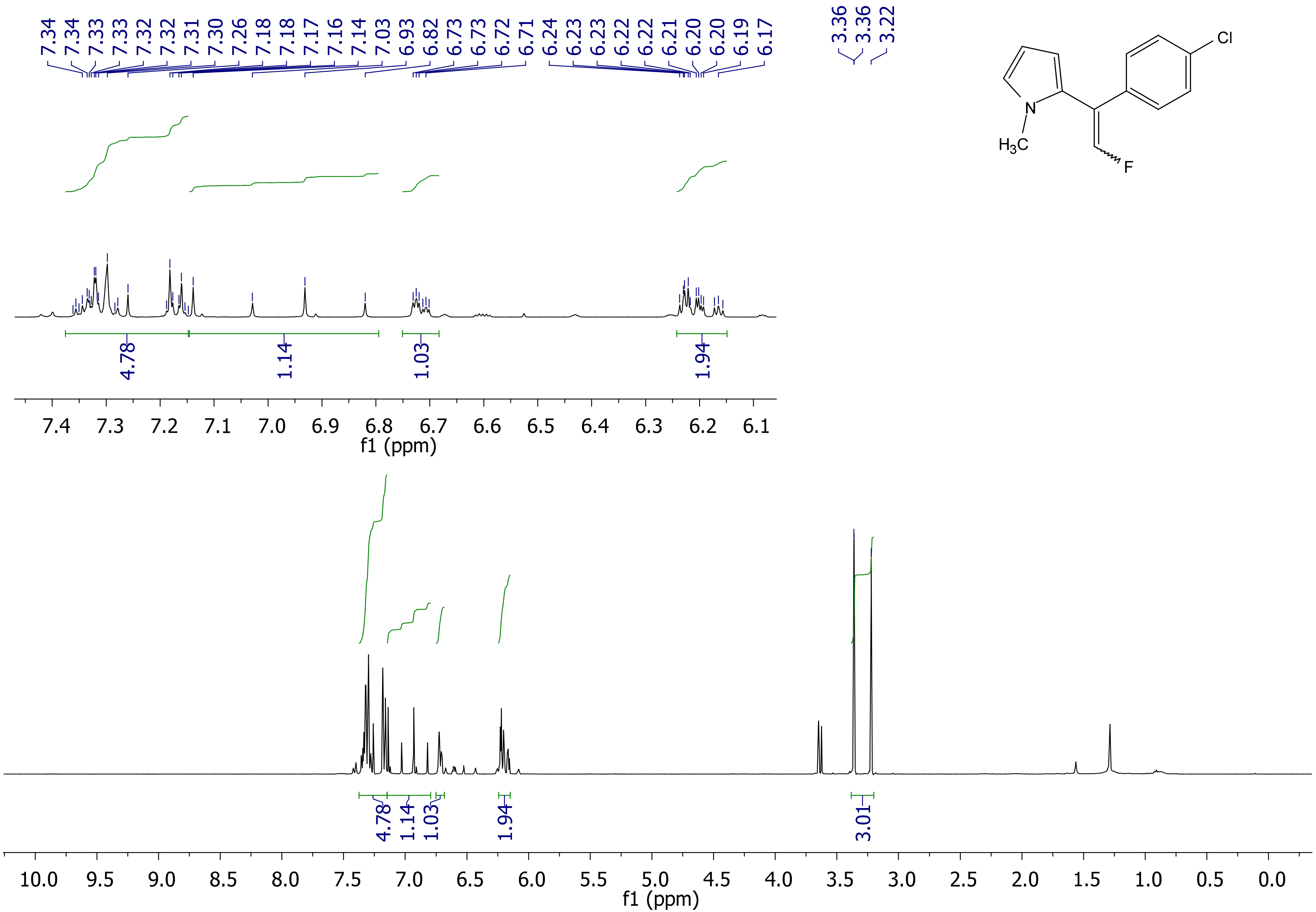

^{1}H NMR spectrum of 2-(1-(4-chlorophenyl)-2-fluorovinyl)-1-methyl-1*H*-pyrrole (**4p**)

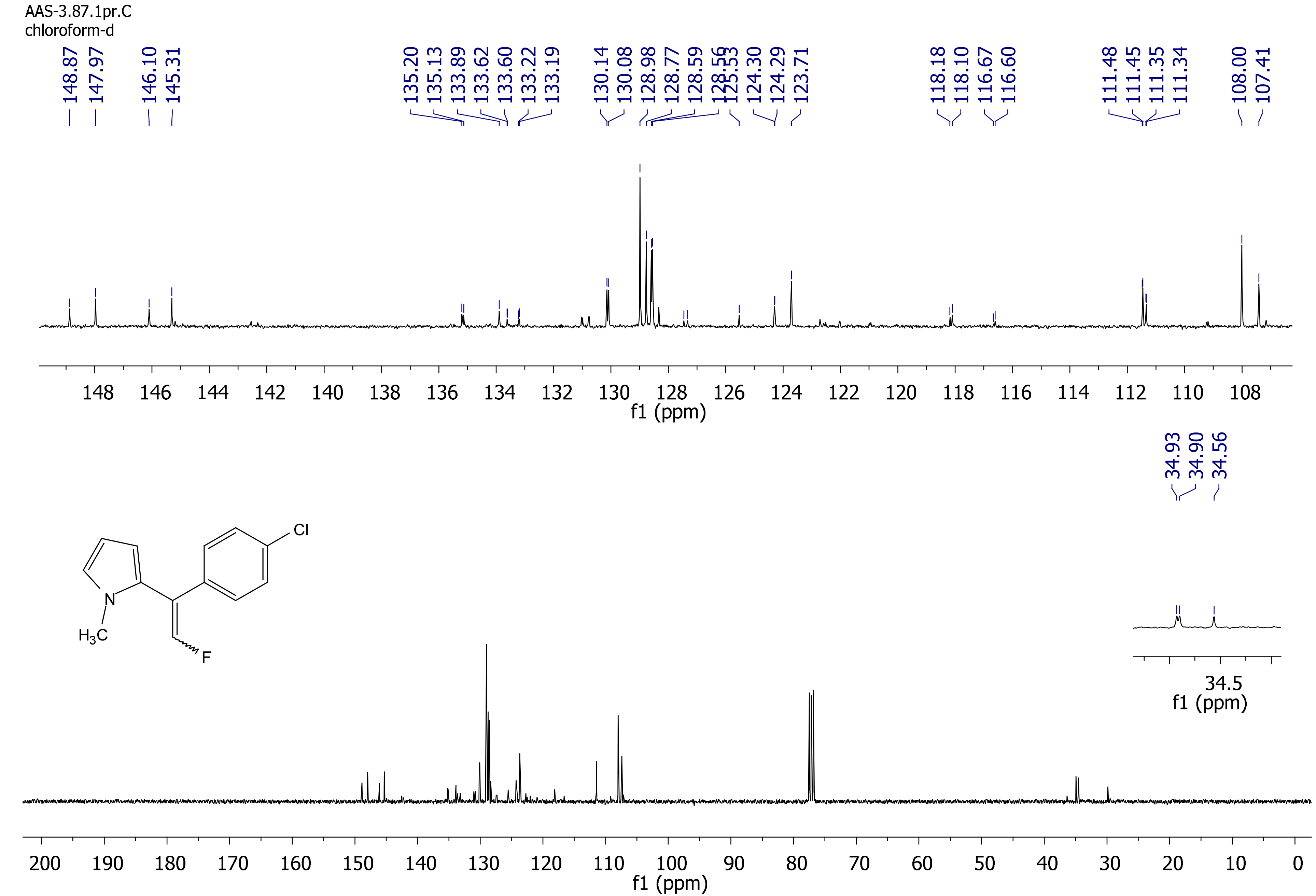

^{13}C NMR spectrum of 2-(1-(4-chlorophenyl)-2-fluorovinyl)-1-methyl-1*H*-pyrrole (**4p**)

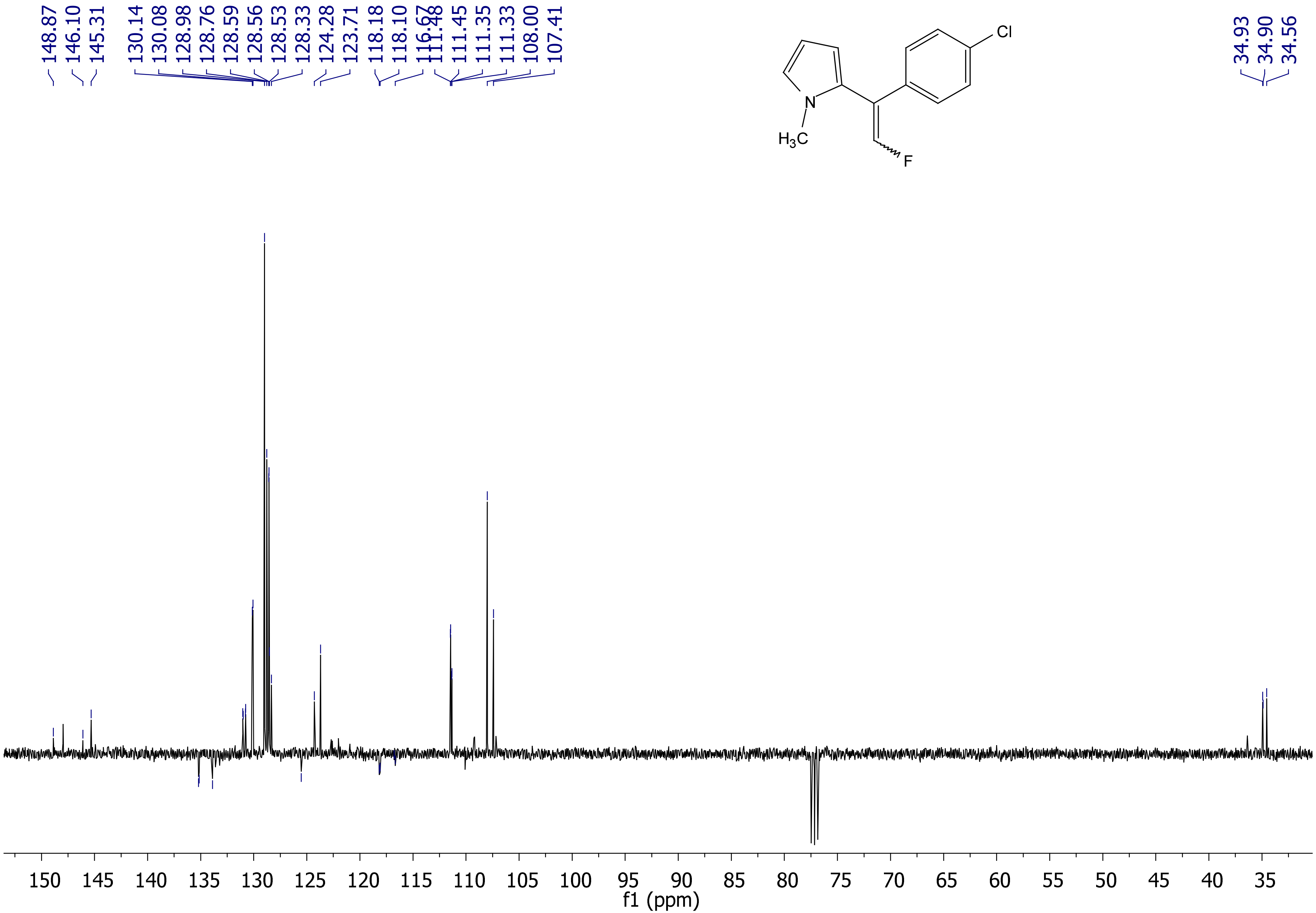

^{13}C NMR APT spectrum of 2-(1-(4-chlorophenyl)-2-fluorovinyl)-1-methyl-1*H*-pyrrole (**4p**)

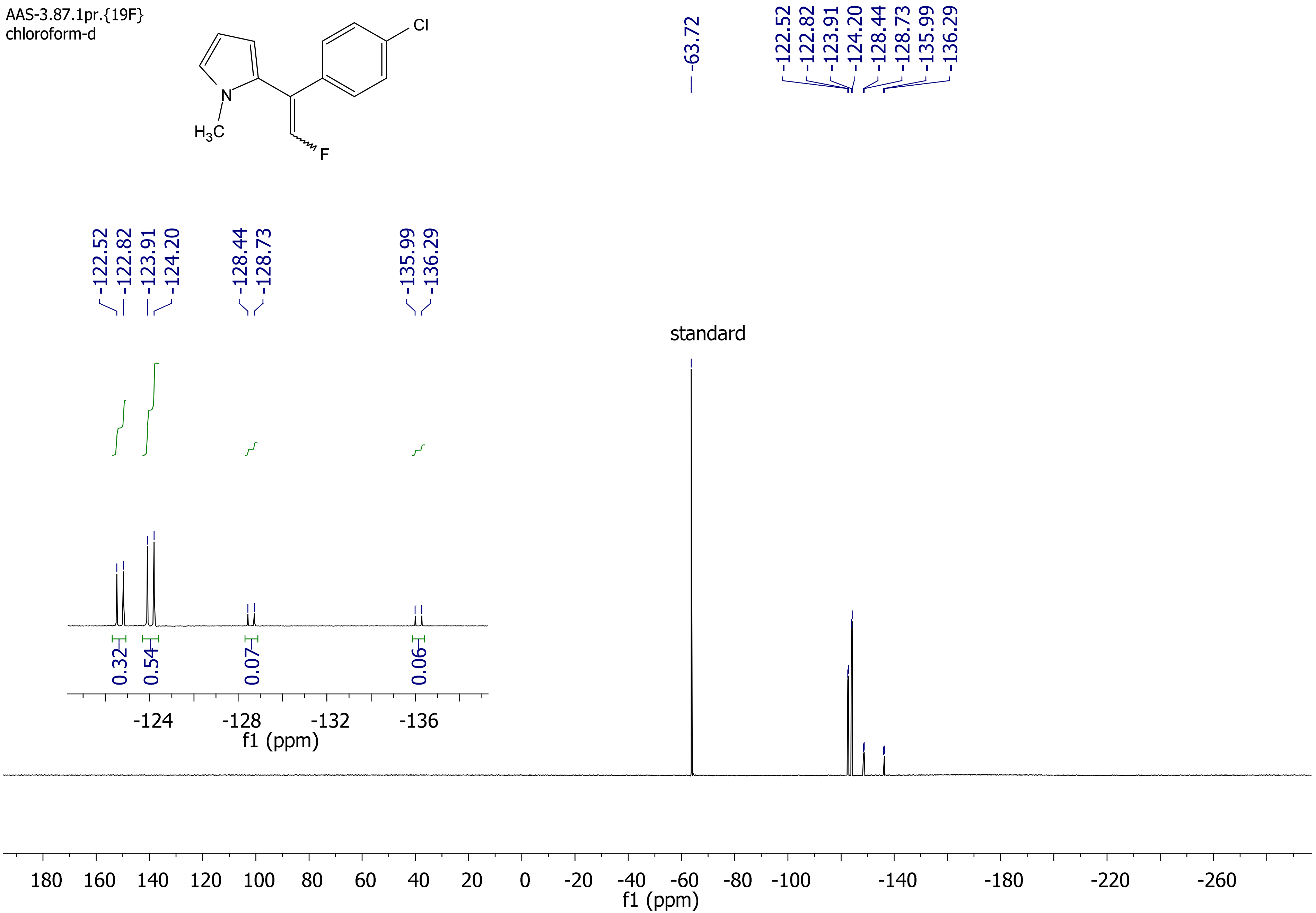

^{19}F NMR spectrum of of 2-(1-(4-chlorophenyl)-2-fluorovinyl)-1-methyl-1*H*-pyrrole (**4p**)

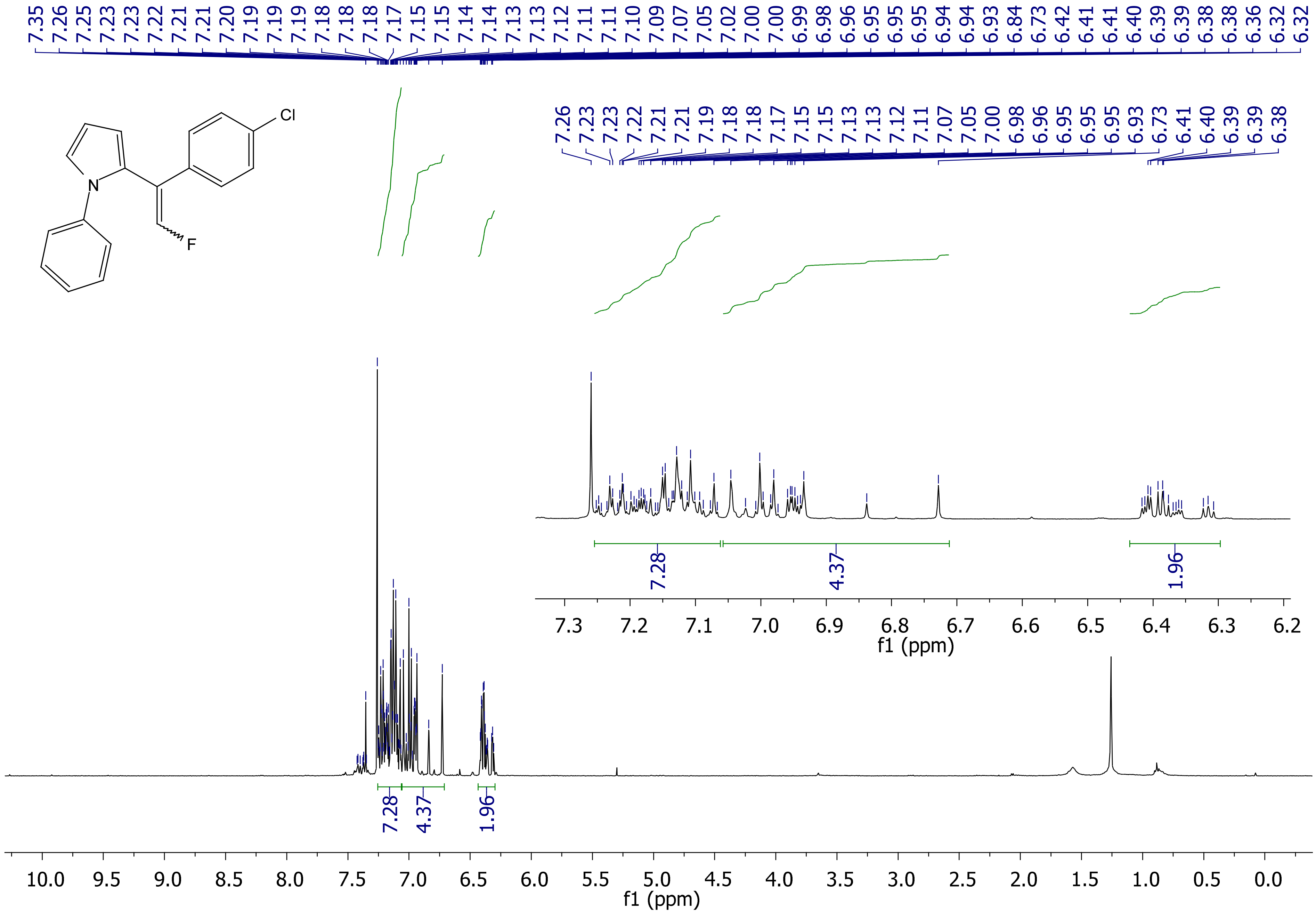

^{1}H NMR spectrum of 2-(1-(4-chlorophenyl)-2-fluorovinyl)-1-phenyl-1*H*-pyrrole (**4q**)

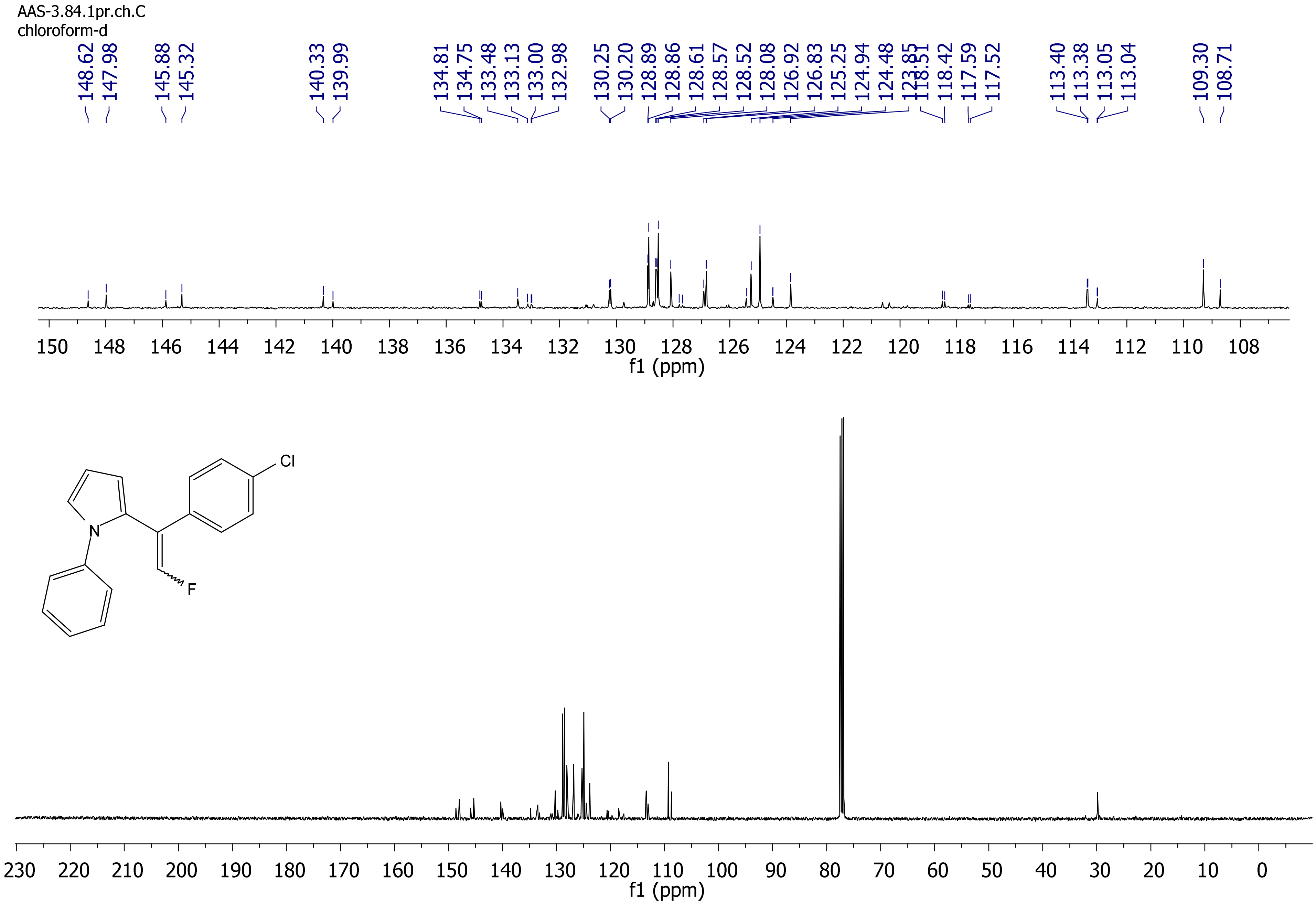

^{13}C NMR spectrum of 2-(1-(4-chlorophenyl)-2-fluorovinyl)-1-phenyl-1*H*-pyrrole (**4q**)

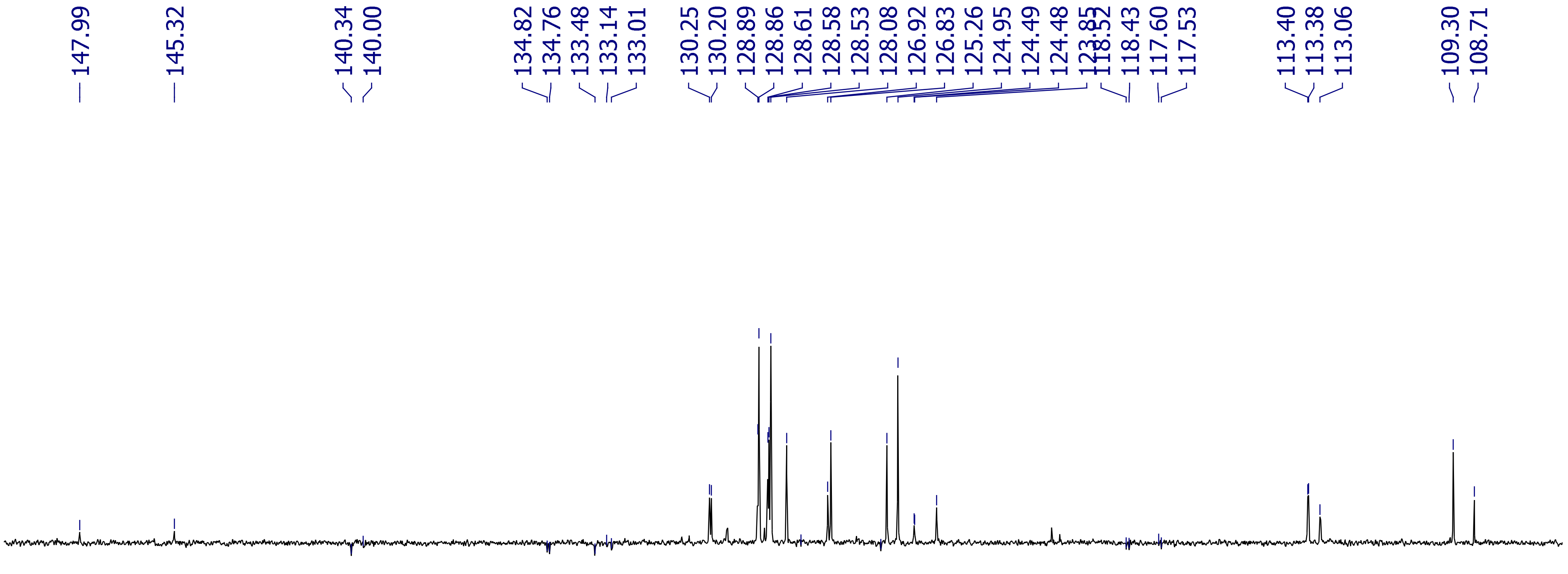

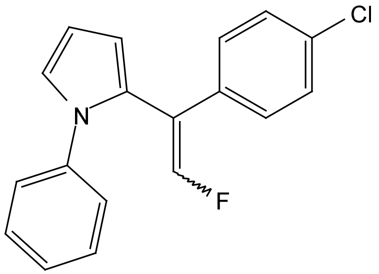

^{13}C NMR APT spectrum of 2-(1-(4-chlorophenyl)-2-fluorovinyl)-1-phenyl-1*H*-pyrrole (**4q**)

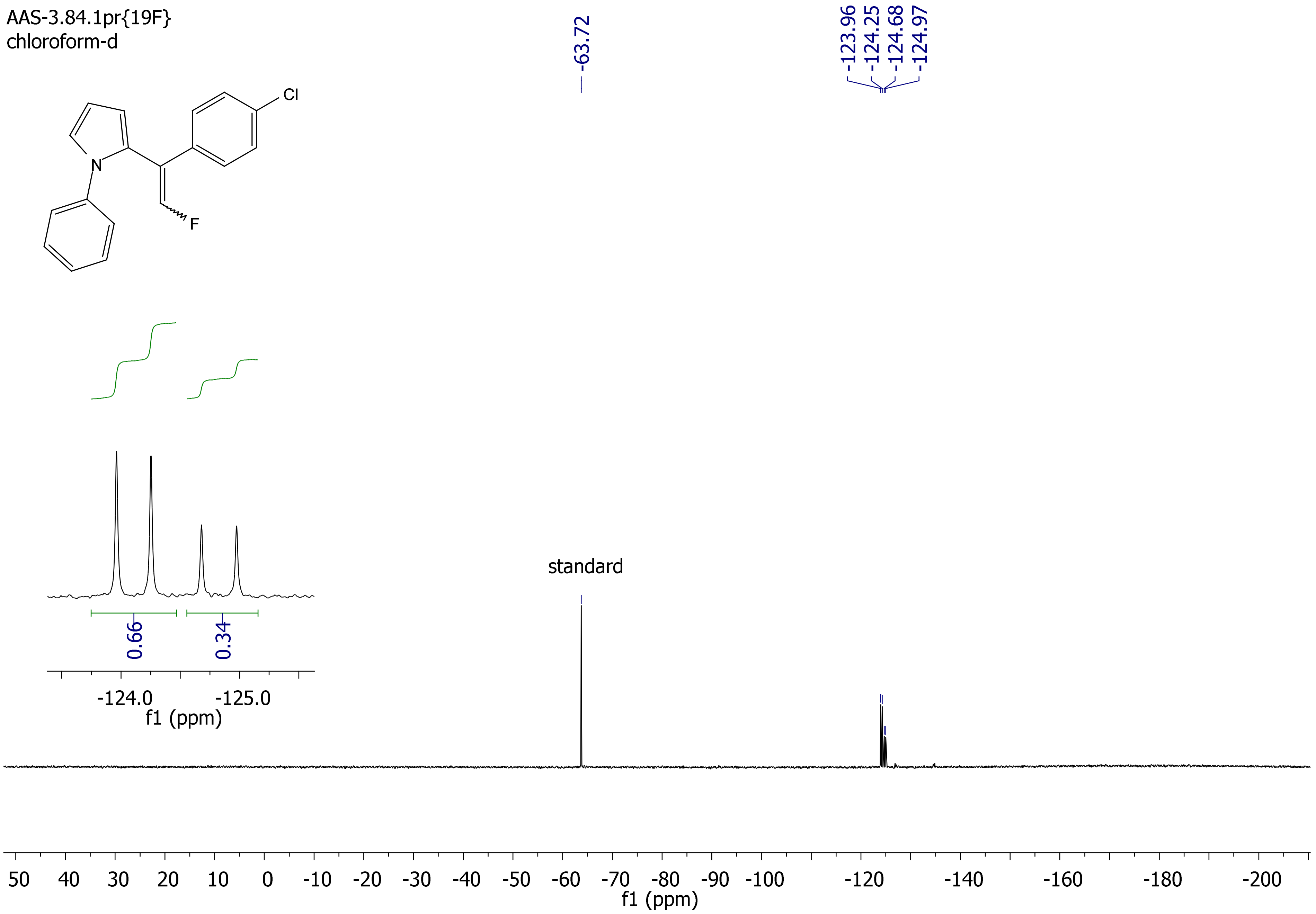

^{19}F NMR spectrum of 2-(1-(4-chlorophenyl)-2-fluorovinyl)-1-phenyl-1*H*-pyrrole (**4q**)

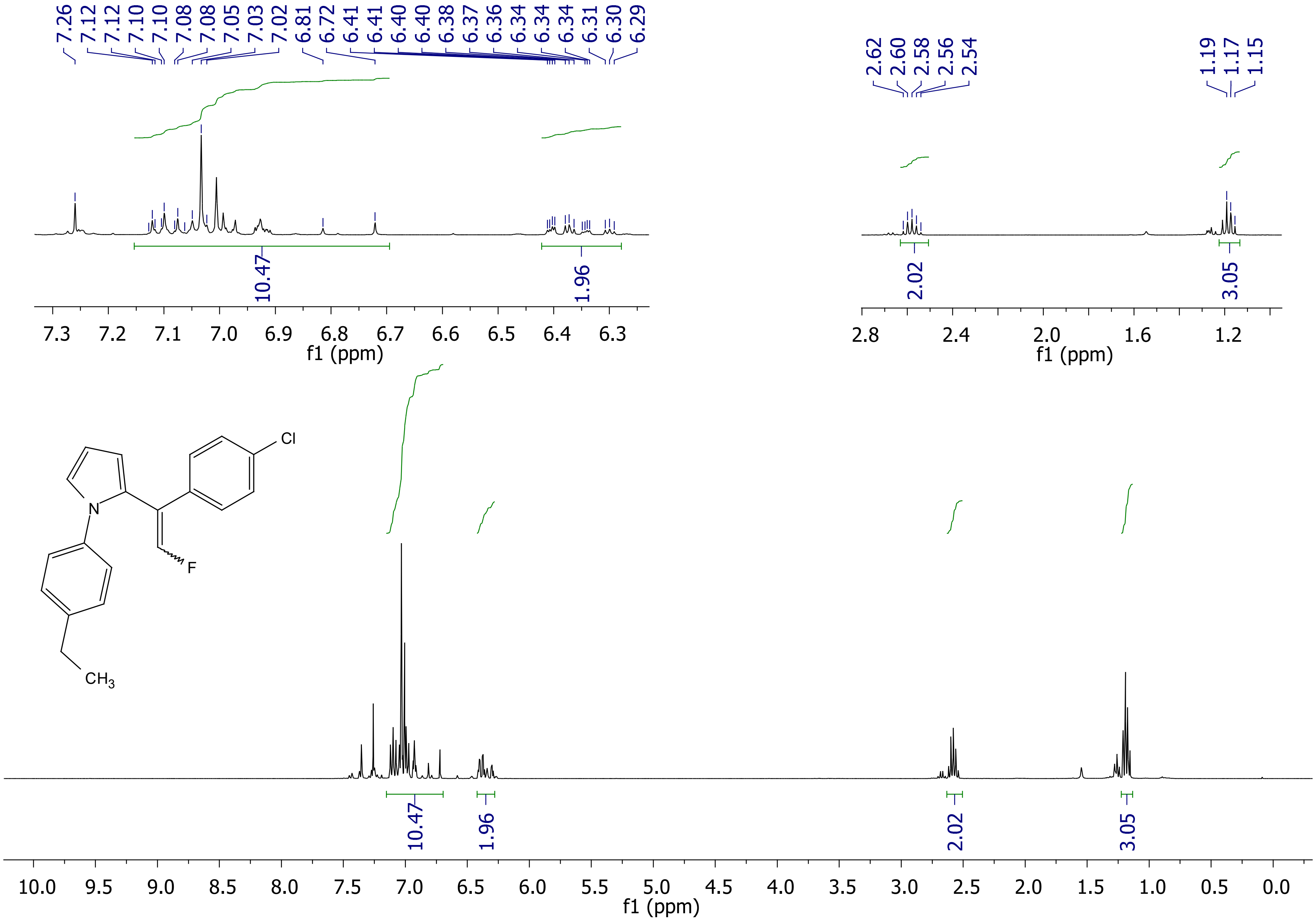

^{1}H NMR spectrum of 2-(1-(4-chlorophenyl)-2-fluorovinyl)-1-(4-ethylphenyl)-1*H*-pyrrole (**4r**)

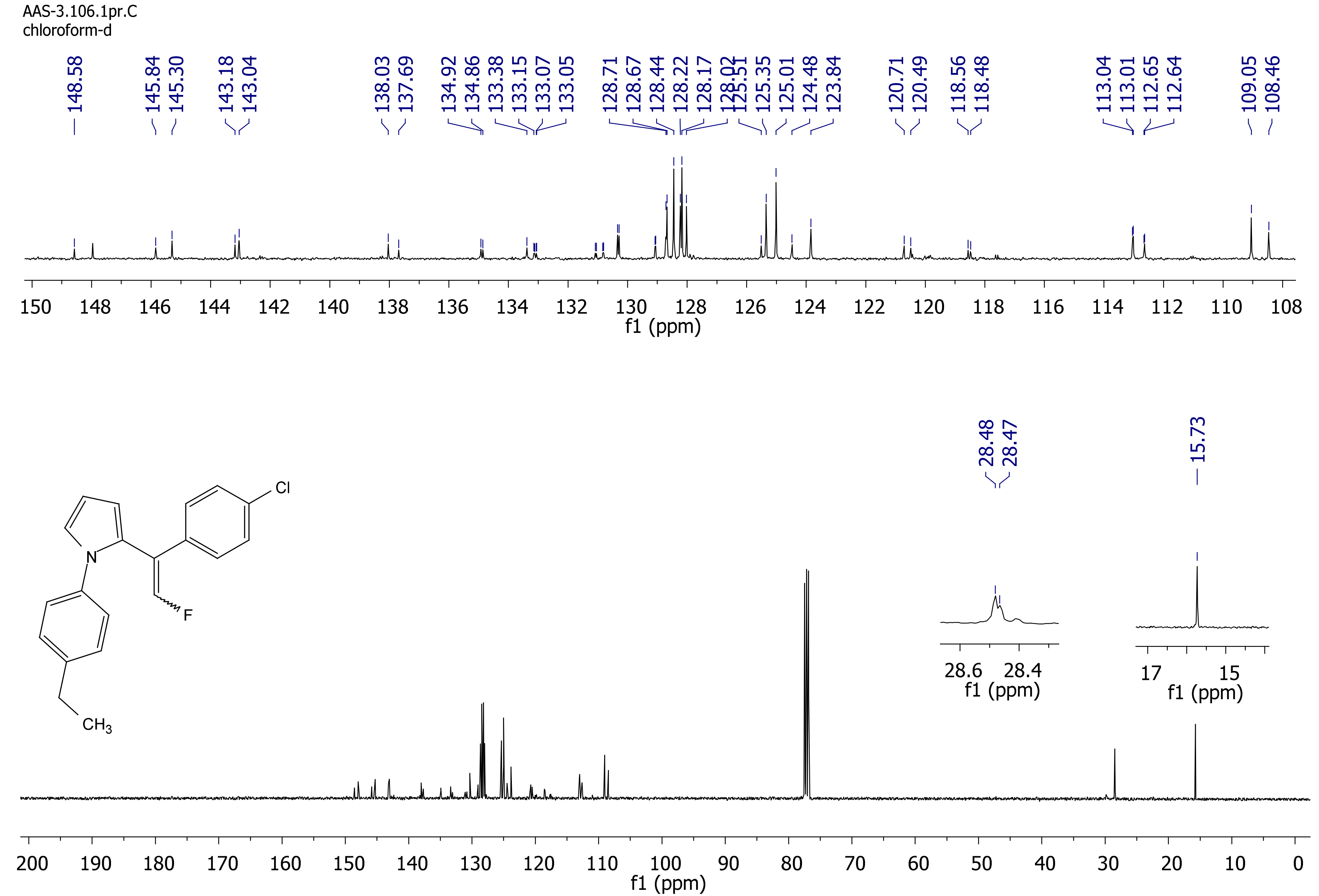

^{13}C NMR spectrum of 2-(1-(4-chlorophenyl)-2-fluorovinyl)-1-(4-ethylphenyl)-1*H*-pyrrole (**4r**)

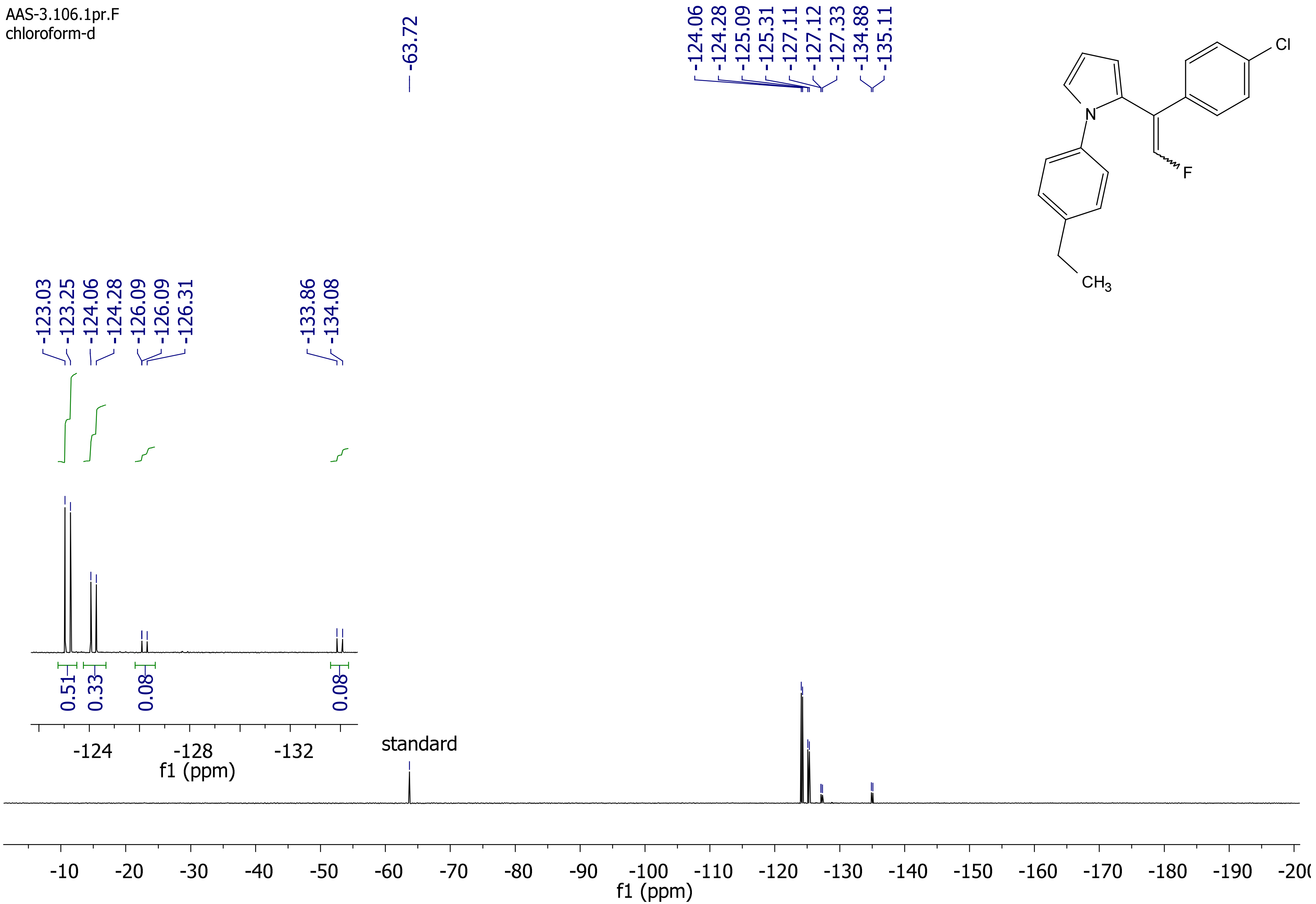

^{19}F NMR spectrum of 2-(1-(4-chlorophenyl)-2-fluorovinyl)-1-(4-ethylphenyl)-1*H*-pyrrole (**4r**)

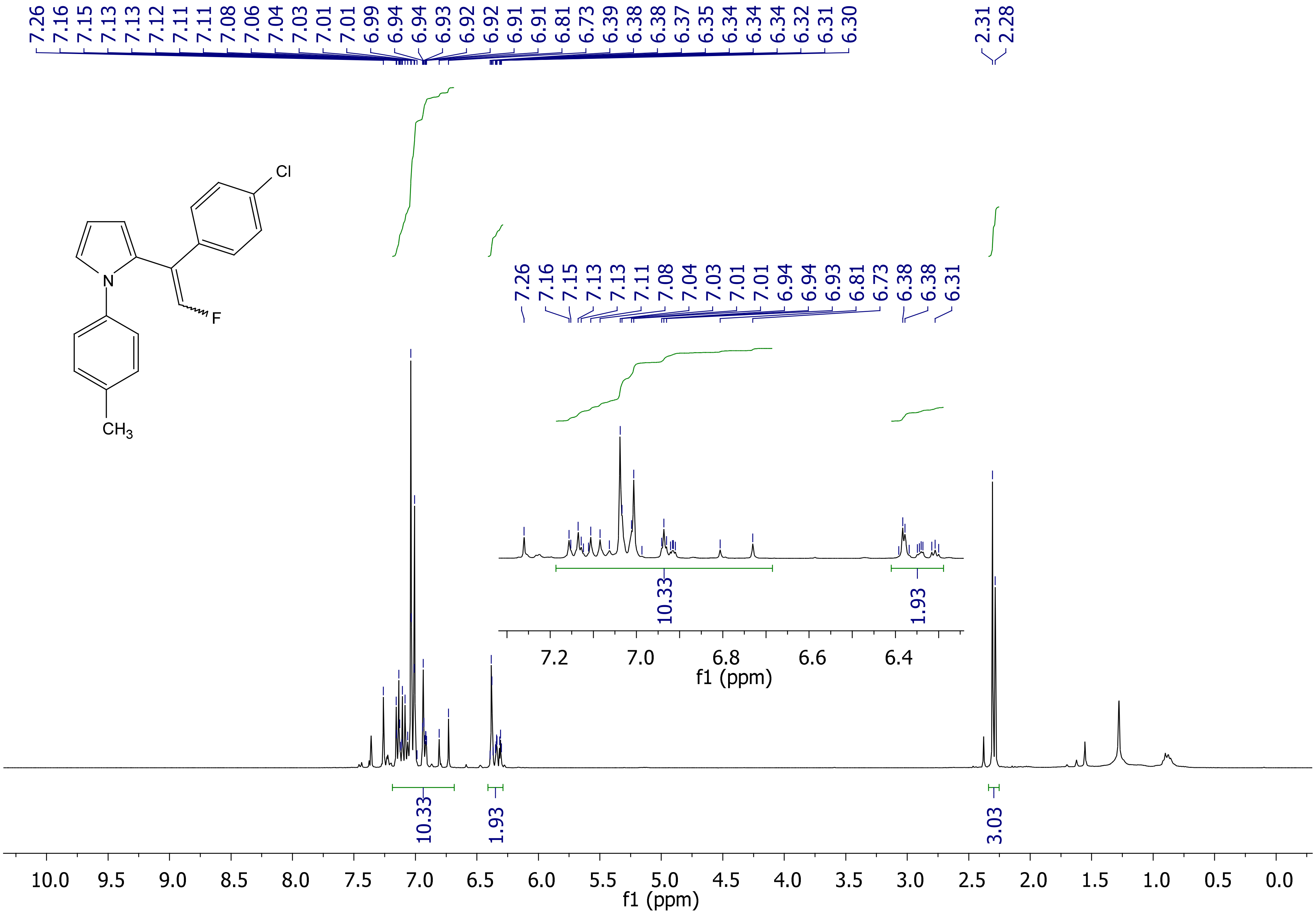

^{1}H NMR spectrum of 2-(1-(4-chlorophenyl)-2-fluorovinyl)-1-(p-tolyl)-1*H*-pyrrole (**4s**)

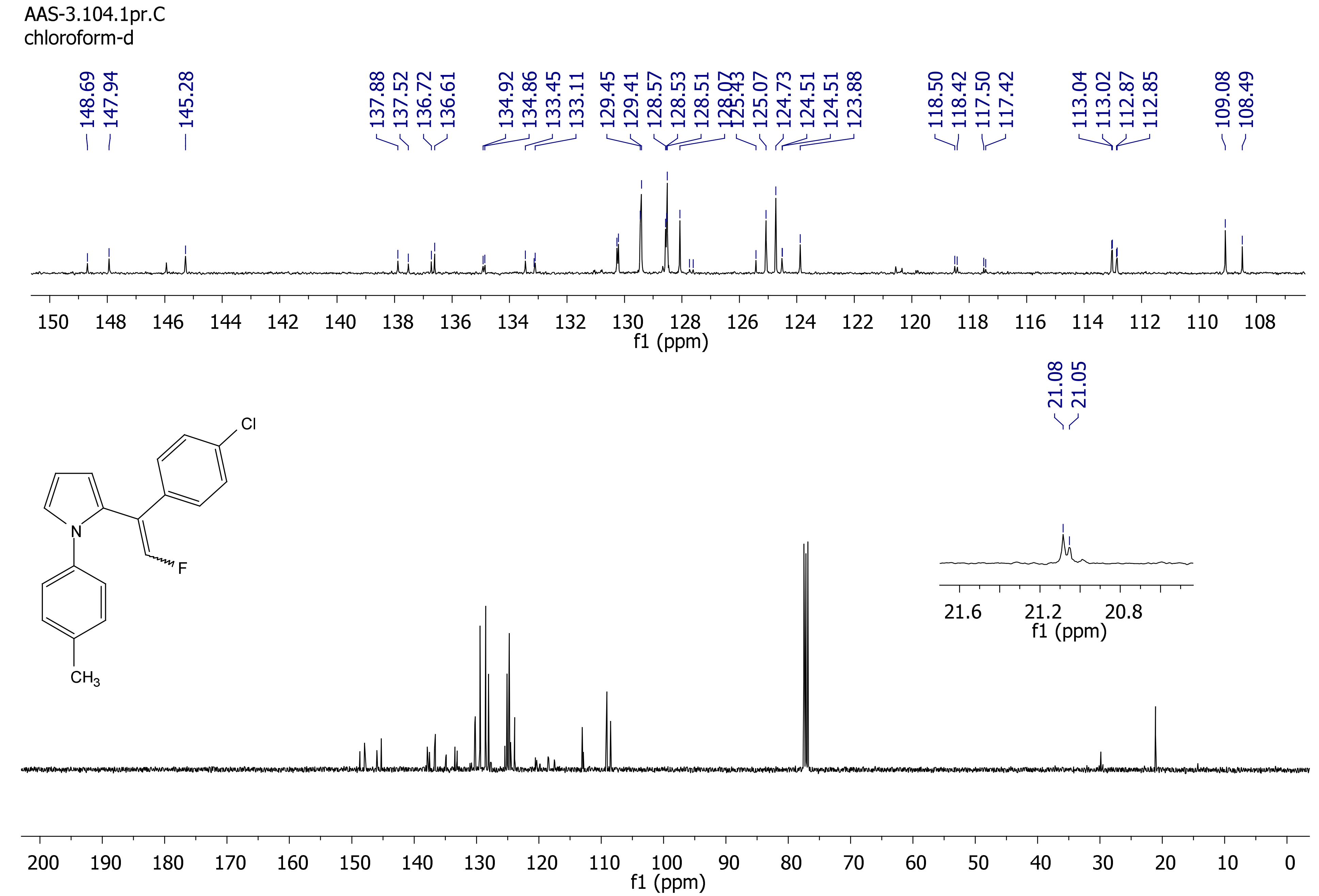

^{13}C NMR spectrum of 2-(1-(4-chlorophenyl)-2-fluorovinyl)-1-(p-tolyl)-1*H*-pyrrole (**4s**)

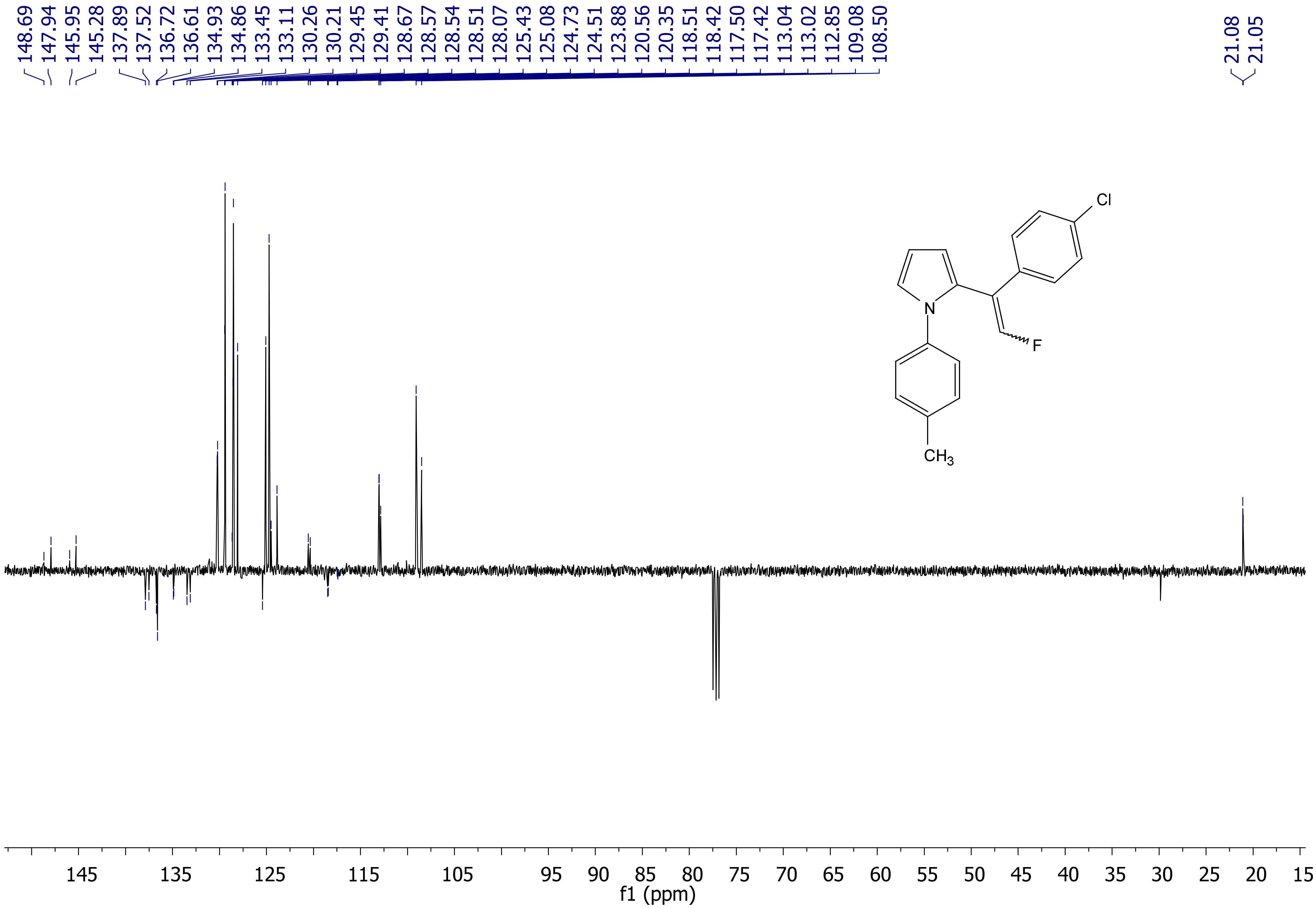

^{13}C NMR APT spectrum of 2-(1-(4-chlorophenyl)-2-fluorovinyl)-1-(p-tolyl)-1*H*-pyrrole (**4s**)

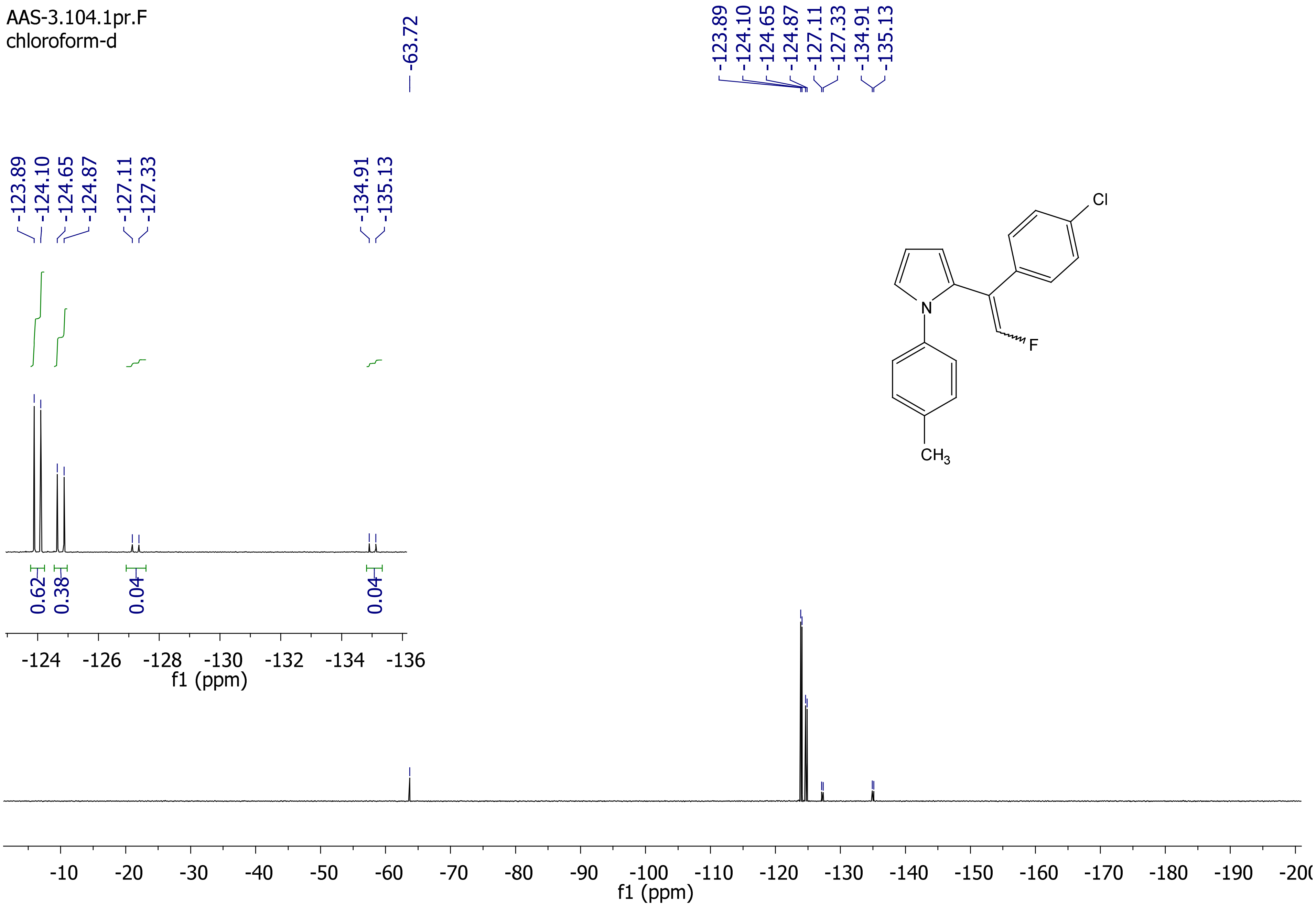

^{19}F NMR spectrum of 2-(1-(4-chlorophenyl)-2-fluorovinyl)-1-(p-tolyl)-1*H*-pyrrole (**4s**)

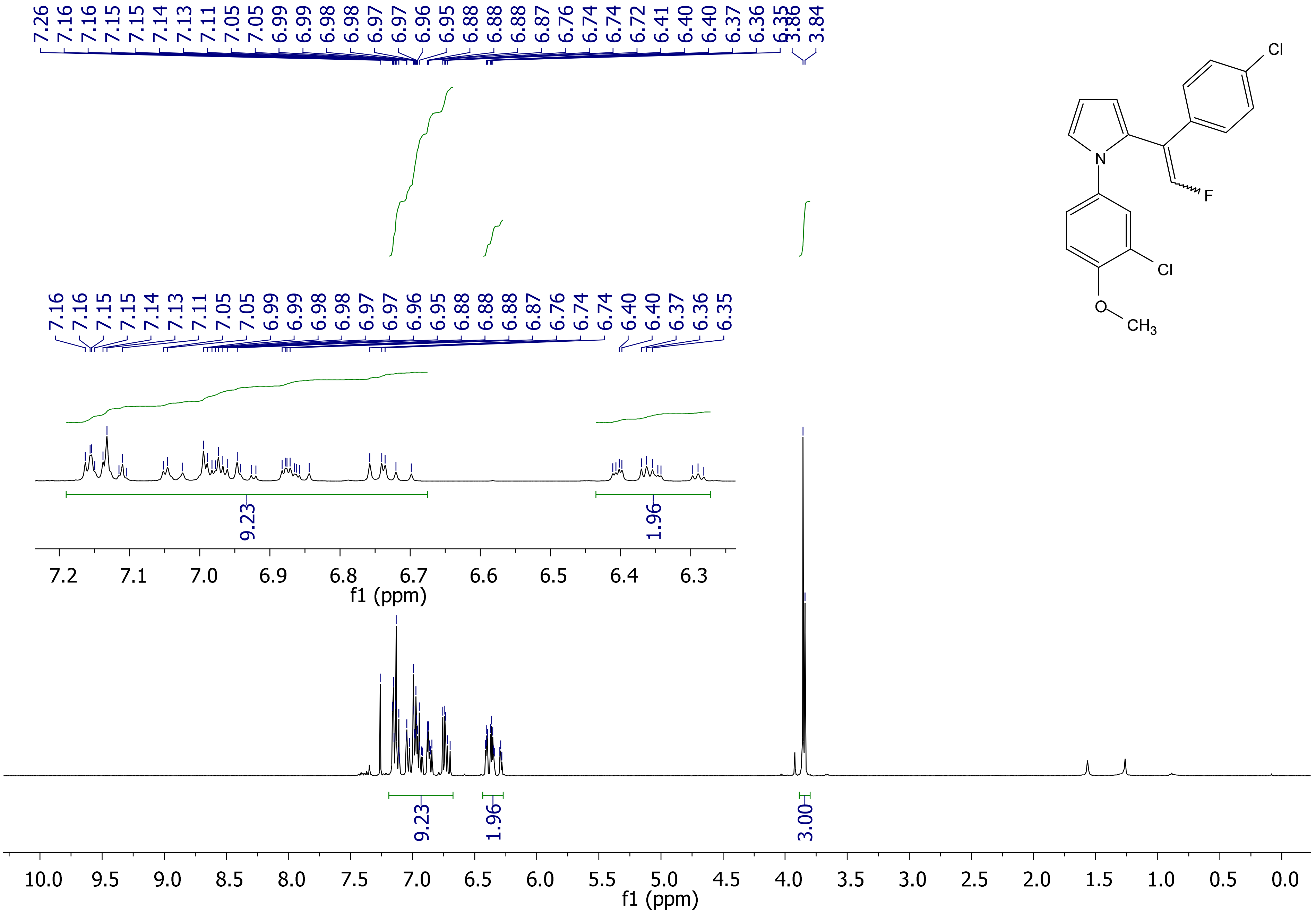

^{1}H NMR spectrum of 1-(3-chloro-4-methoxyphenyl)-2-(1-(4-chlorophenyl)-2-fluorovinyl)-1*H*-pyrrole (**4t**)

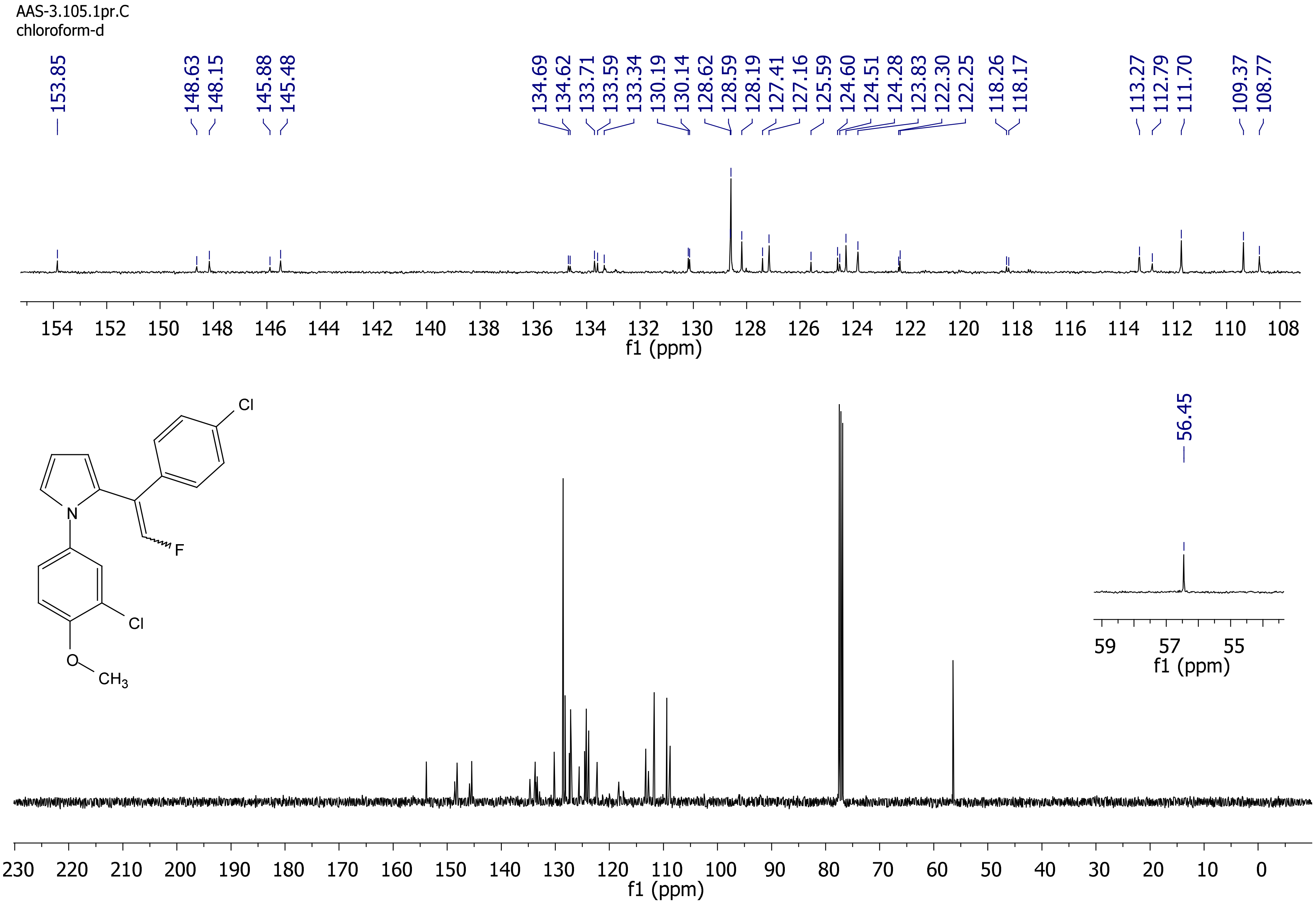

^{13}C NMR spectrum of 1-(3-chloro-4-methoxyphenyl)-2-(1-(4-chlorophenyl)-2-fluorovinyl)-1*H*-pyrrole (**4t**)

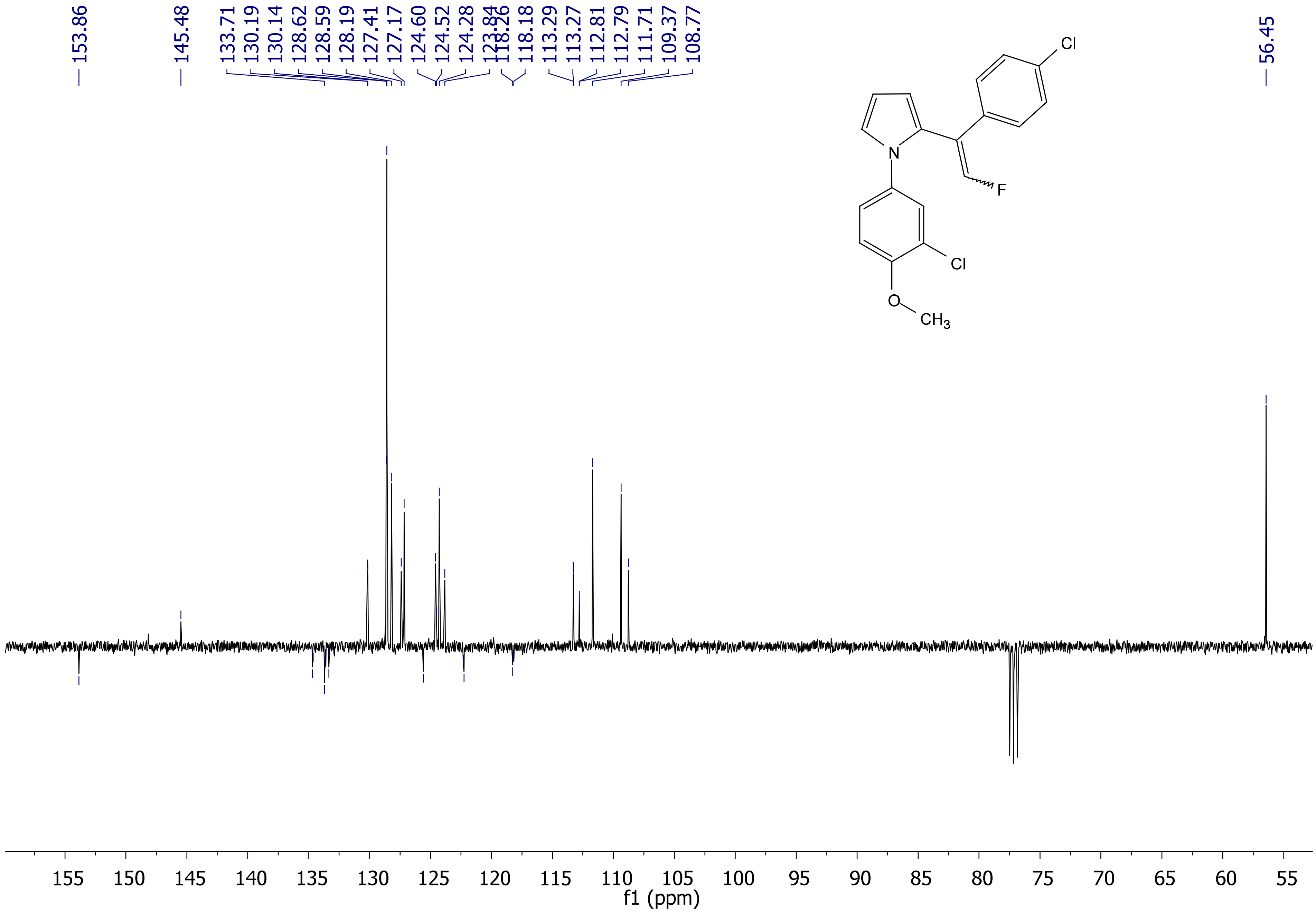

^{13}C NMR APT spectrum of 1-(3-chloro-4-methoxyphenyl)-2-(1-(4-chlorophenyl)-2-fluorovinyl)-1*H*-pyrrole (**4t**)

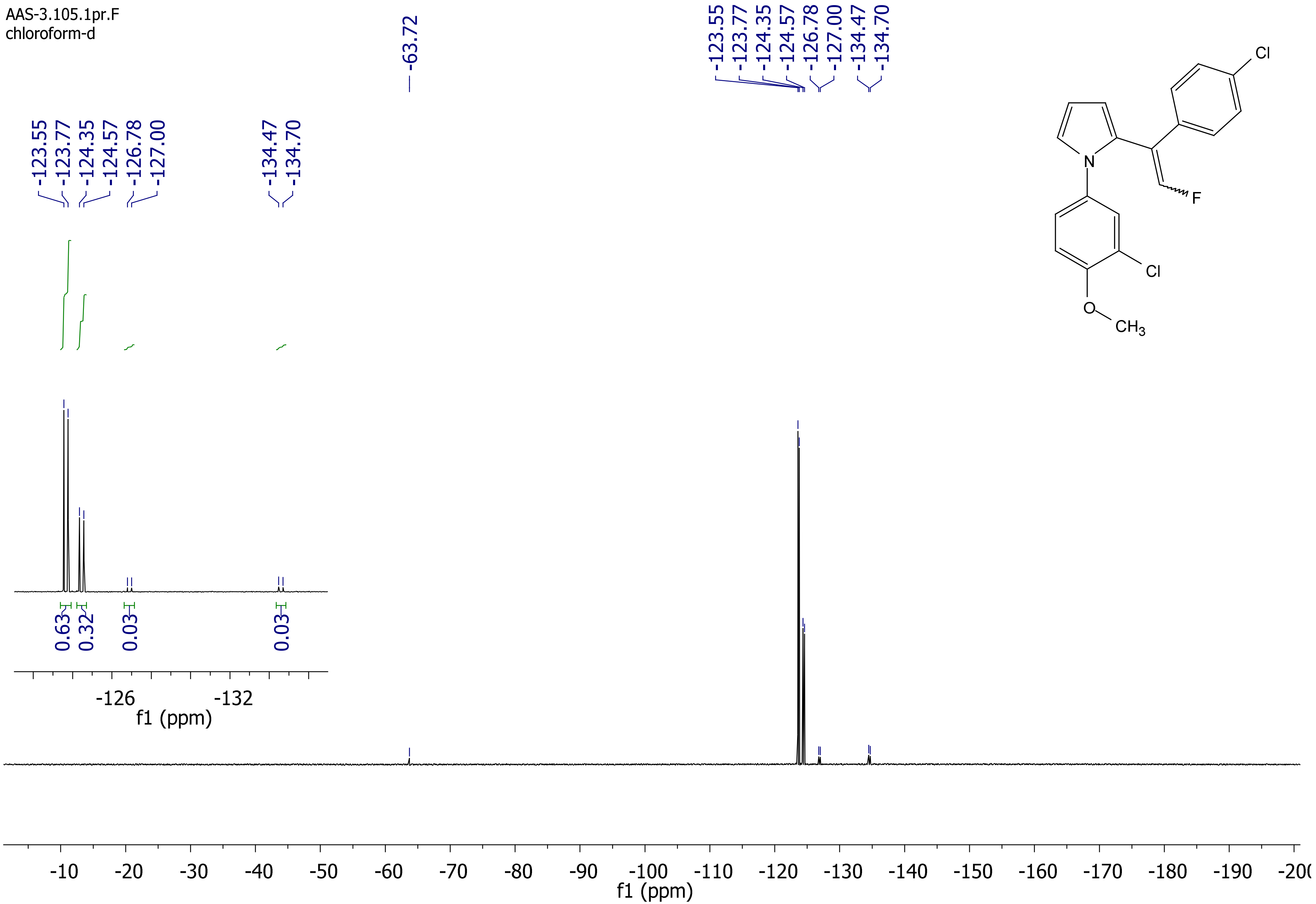

^{19}F NMR spectrum of 1-(3-chloro-4-methoxyphenyl)-2-(1-(4-chlorophenyl)-2-fluorovinyl)-1*H*-pyrrole (**4t**)

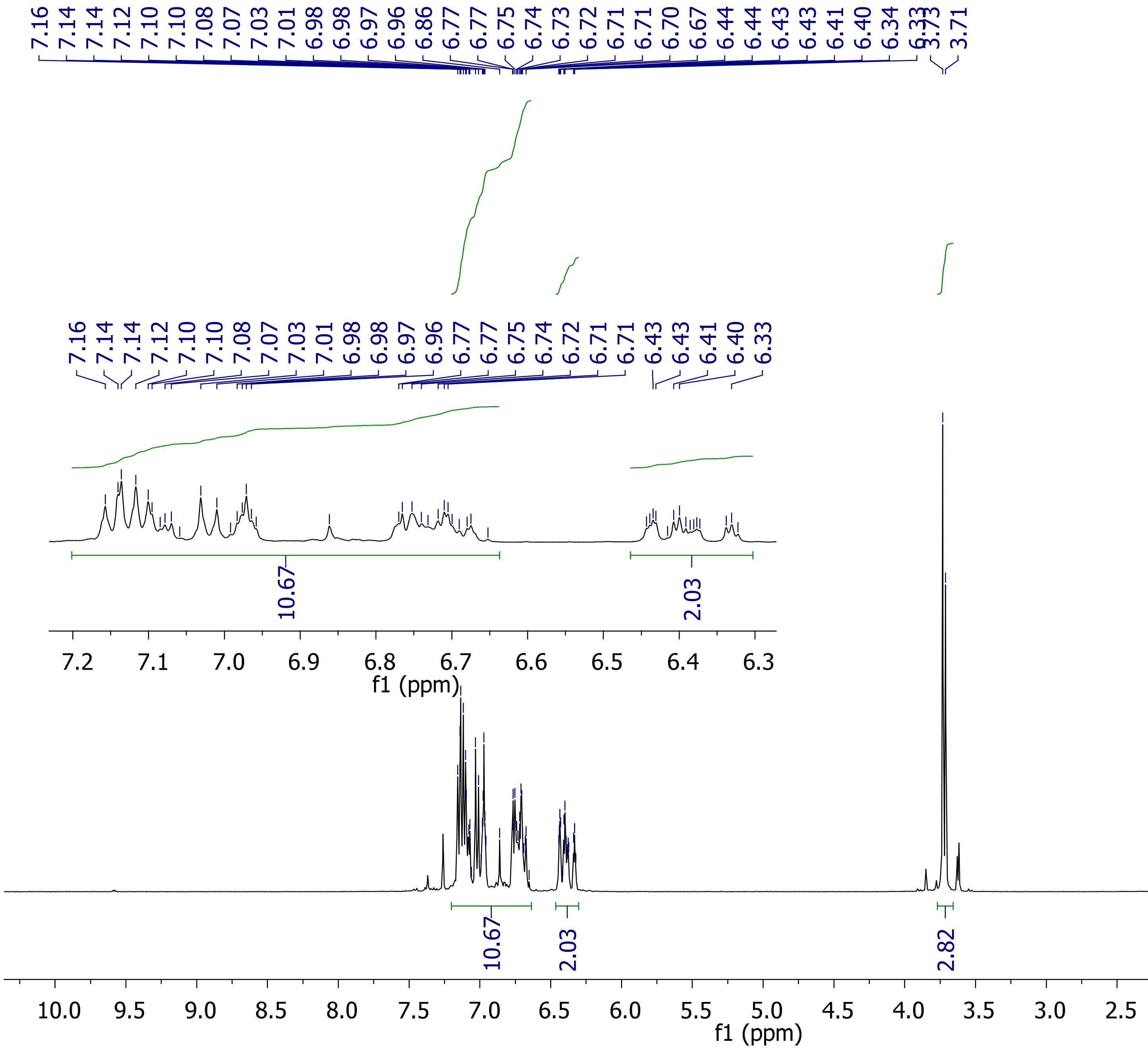

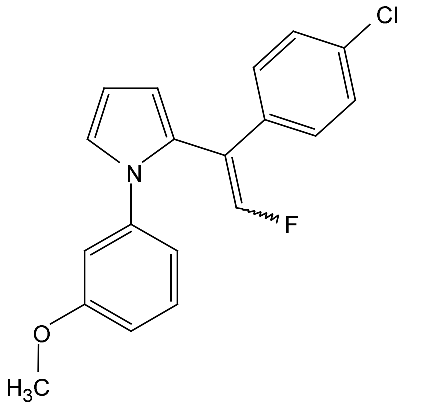

^{1}H NMR spectrum of 2-(1-(4-chlorophenyl)-2-fluorovinyl)-1-(3-methoxyphenyl)-1*H*-pyrrole (**4u**)

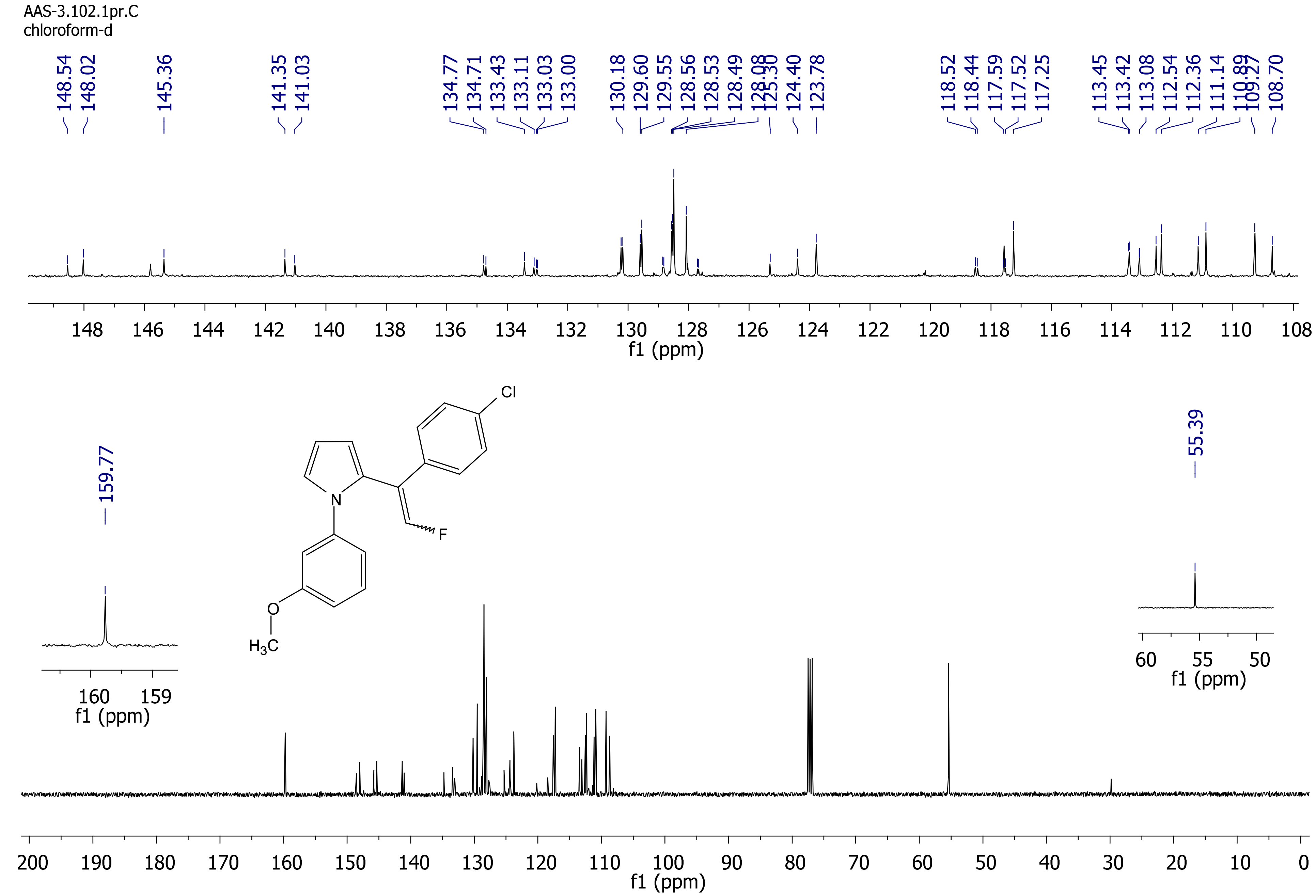

^{13}C NMR spectrum of 2-(1-(4-chlorophenyl)-2-fluorovinyl)-1-(3-methoxyphenyl)-1*H*-pyrrole (**4u**)

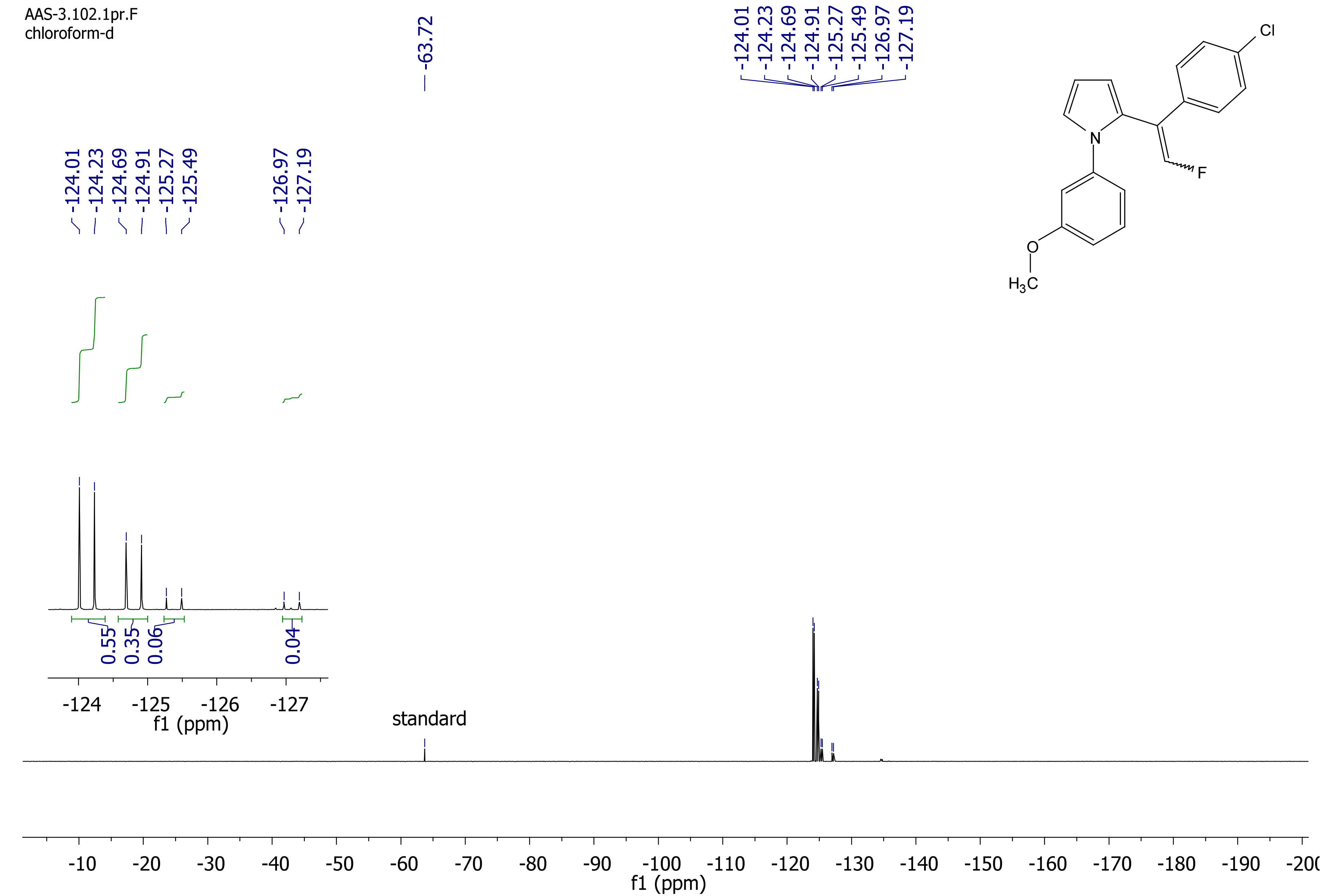

^{19}F NMR spectrum of 2-(1-(4-chlorophenyl)-2-fluorovinyl)-1-(3-methoxyphenyl)-1*H*-pyrrole (**4u**)

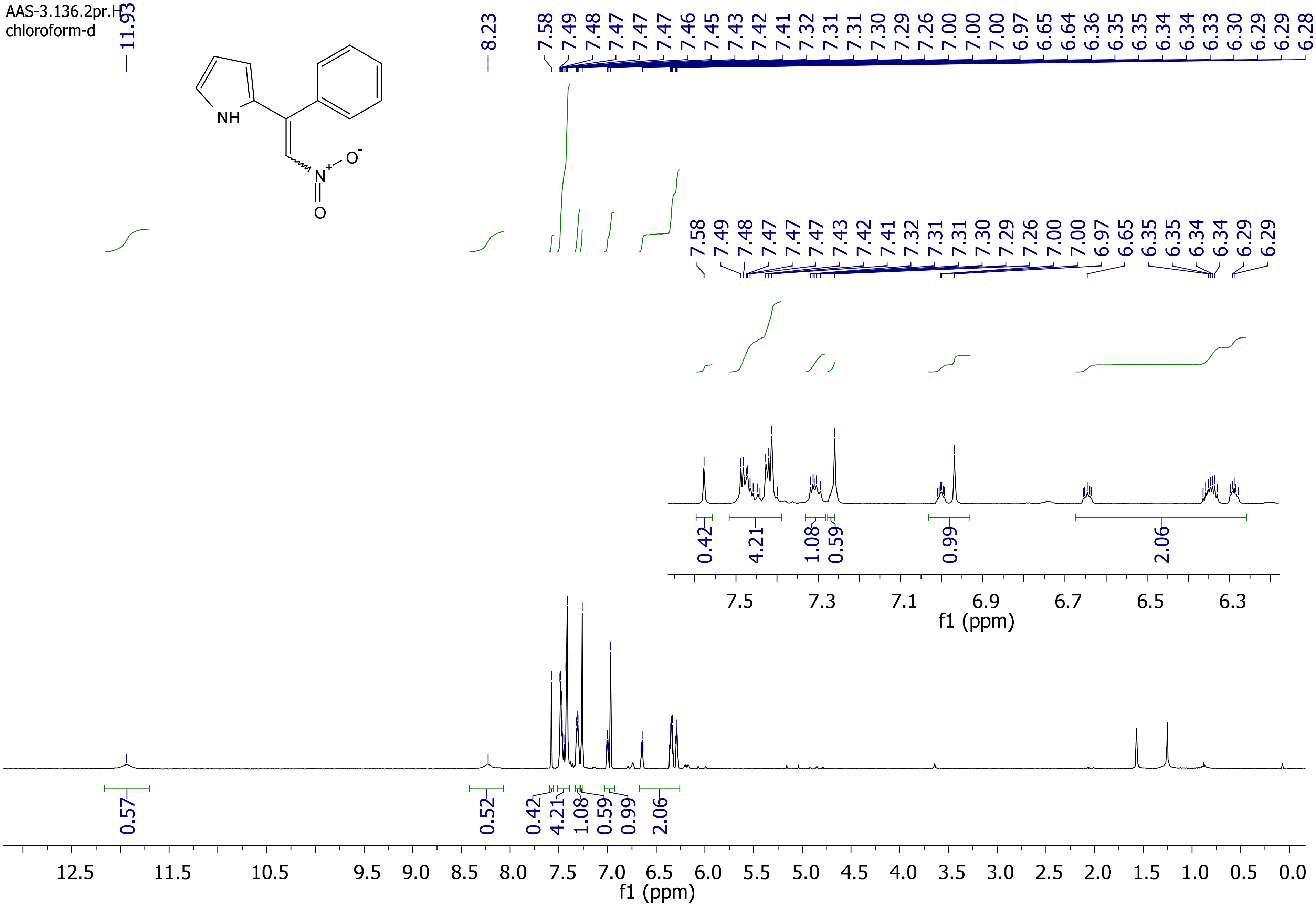

^{1}H NMR spectrum of 2-(2-nitro-1-phenylvinyl)-1*H*-pyrrole (**5a**)

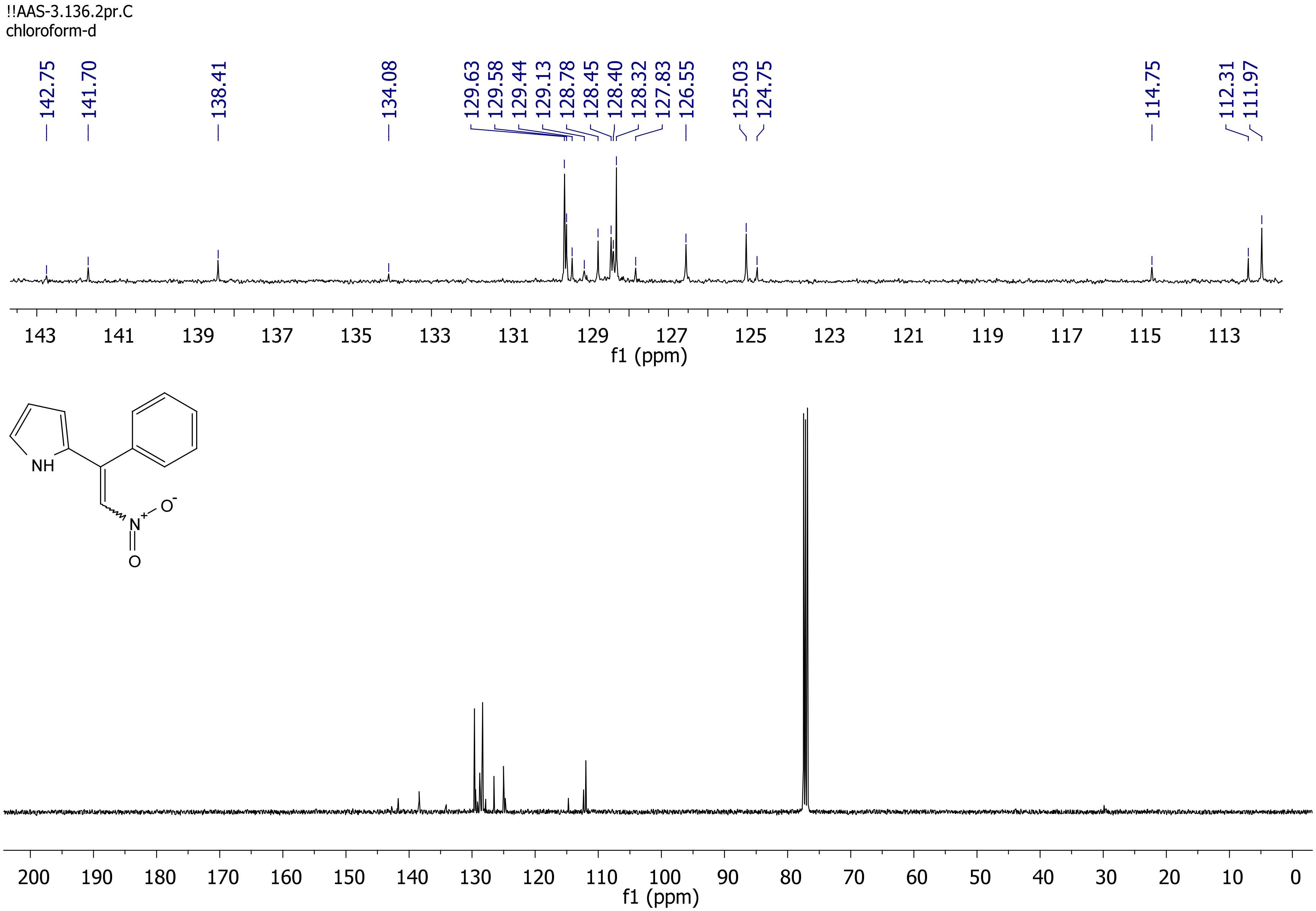

^{13}C NMR spectrum of 2-(2-nitro-1-phenylvinyl)-1*H*-pyrrole (**5a**)

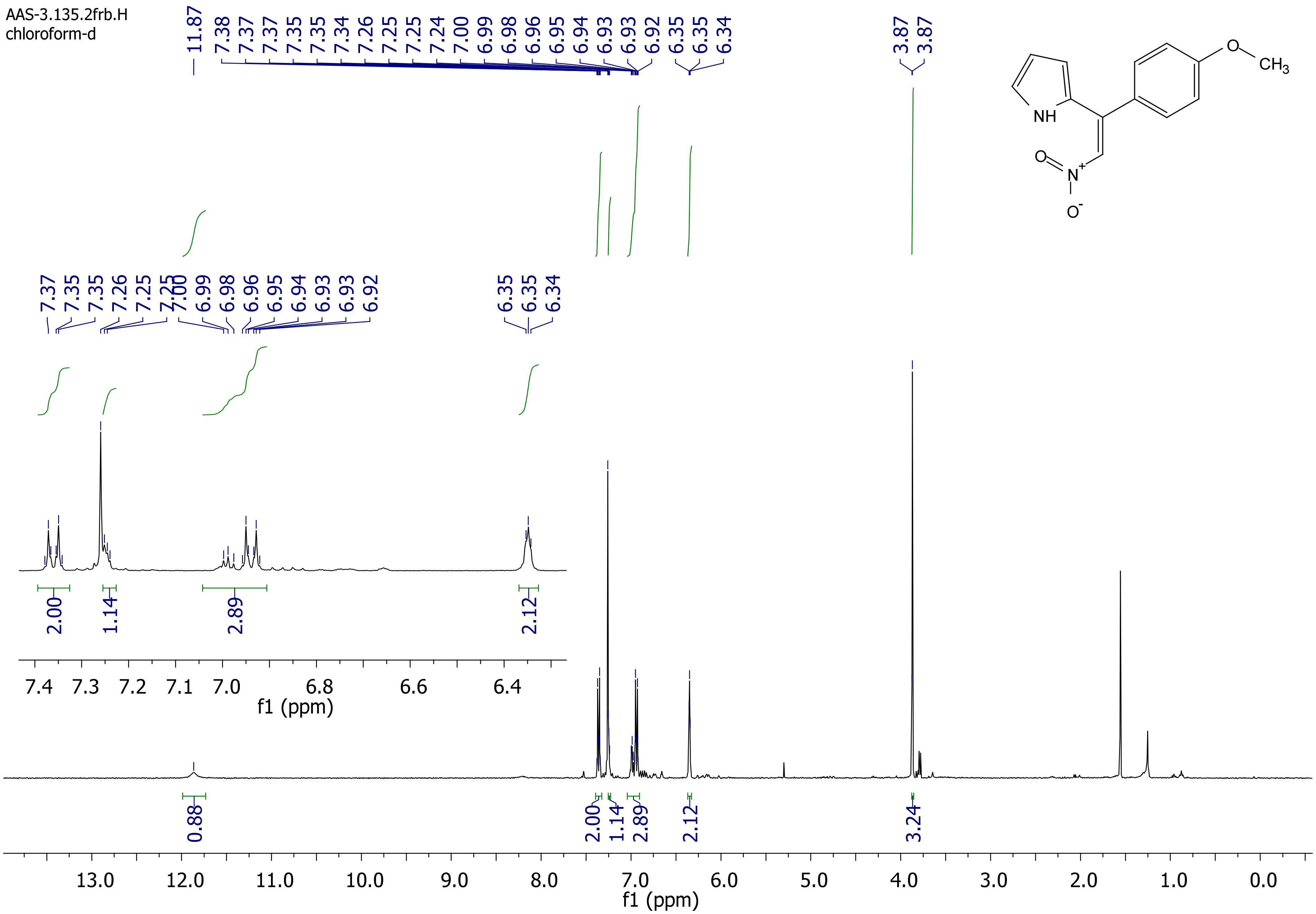

^{1}H NMR spectrum of (*Z*)-2-(1-(4-methoxyphenyl)-2-nitrovinyl)-1*H*-pyrrole (*Z*-**5b**)

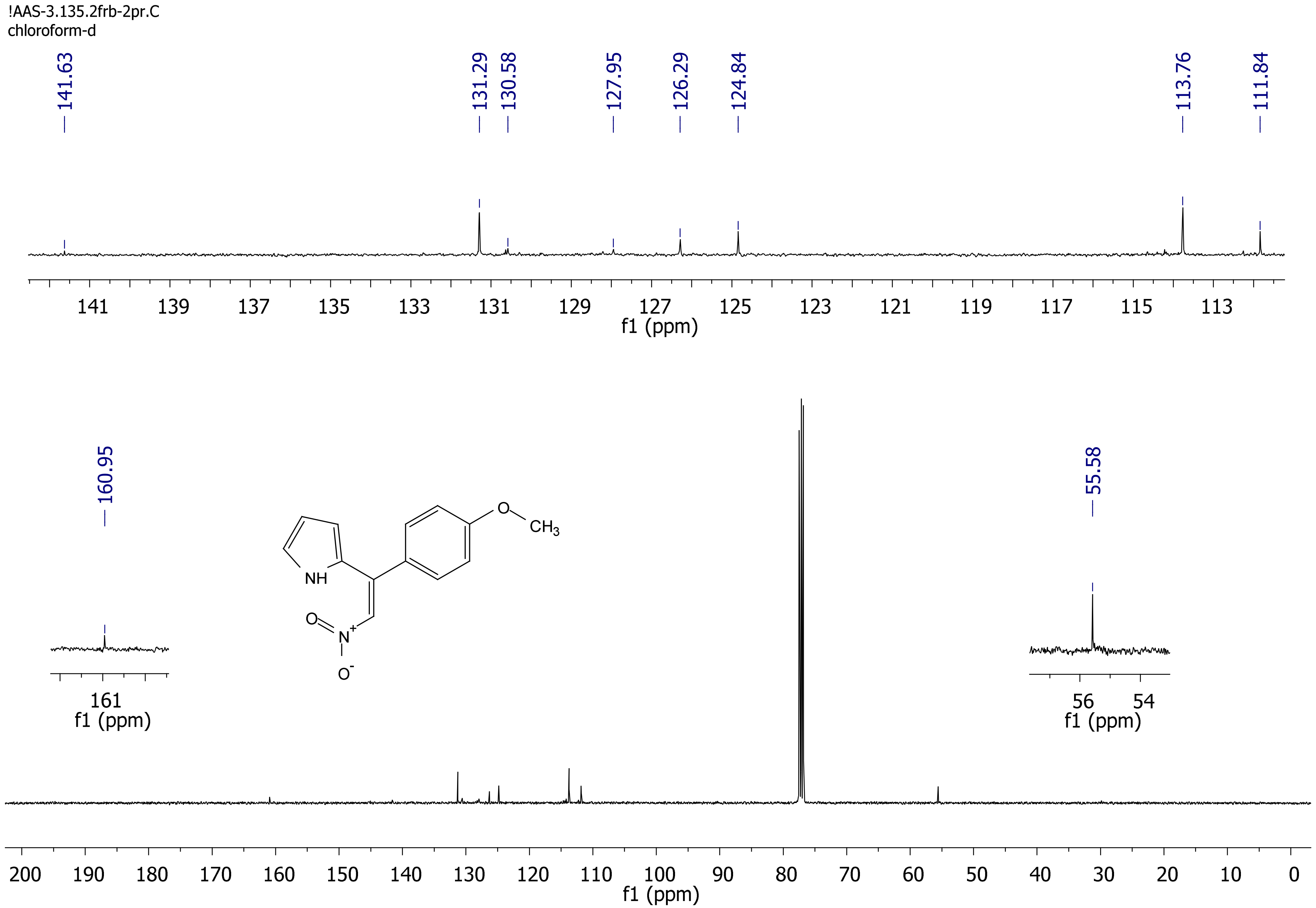

^{13}C NMR spectrum of (*Z*)-2-(1-(4-methoxyphenyl)-2-nitrovinyl)-1*H*-pyrrole (*Z*-**5b**)

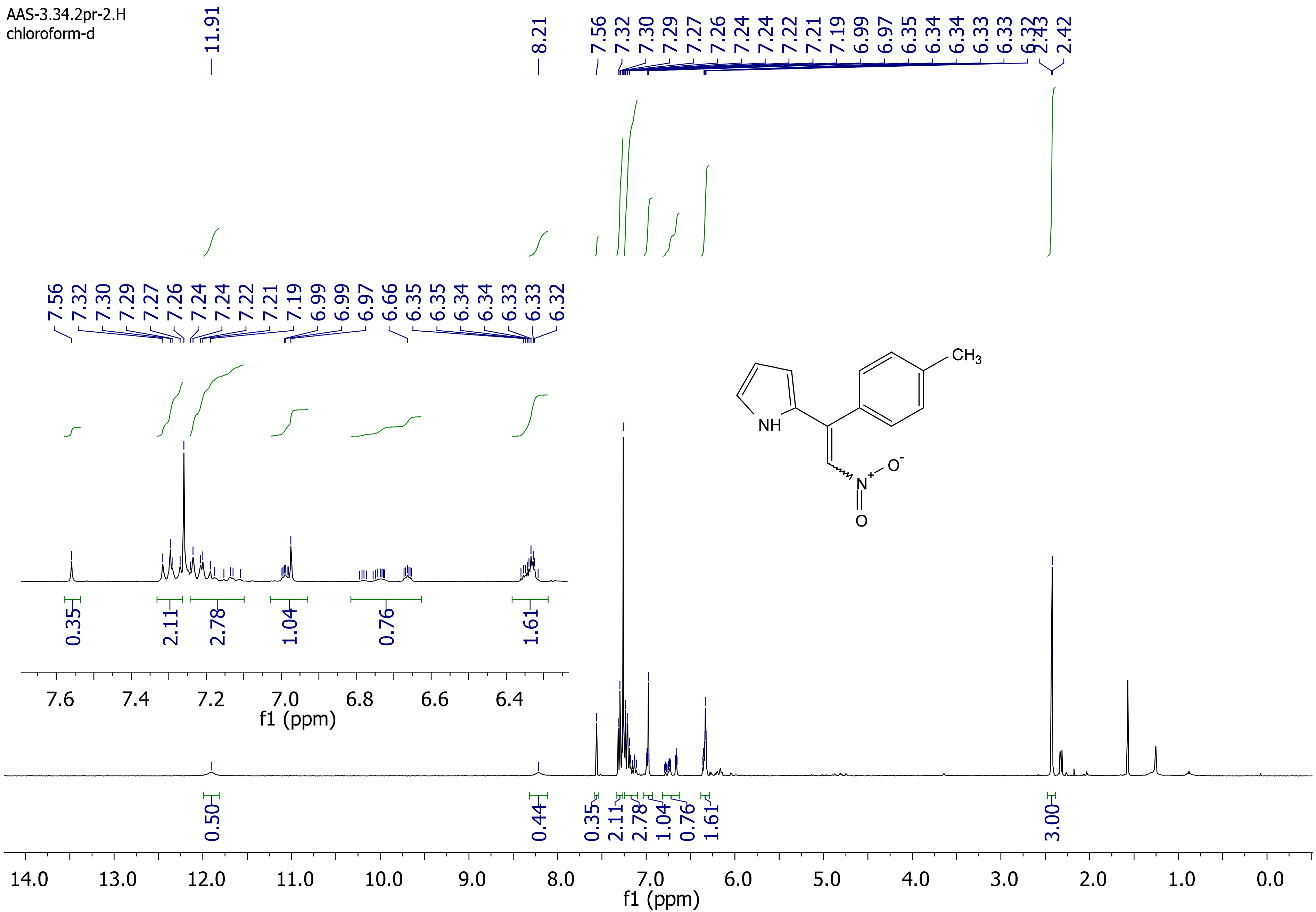

^{1}H NMR spectrum of 2-(2-nitro-1-(p-tolyl)vinyl)-1*H*-pyrrole (**5d**)

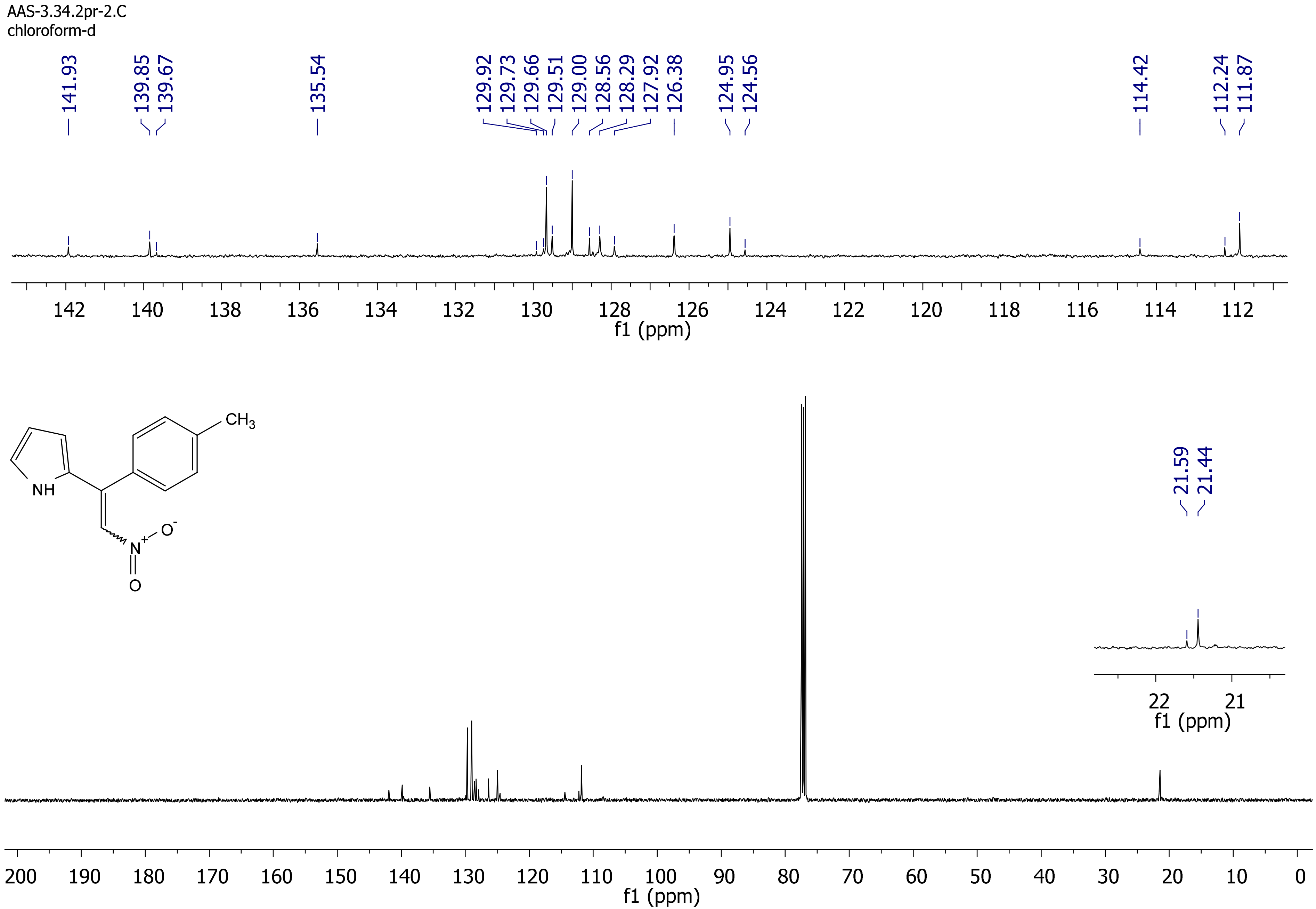

^{13}C NMR spectrum of 2-(2-nitro-1-(p-tolyl)vinyl)-1*H*-pyrrole (**5d**)

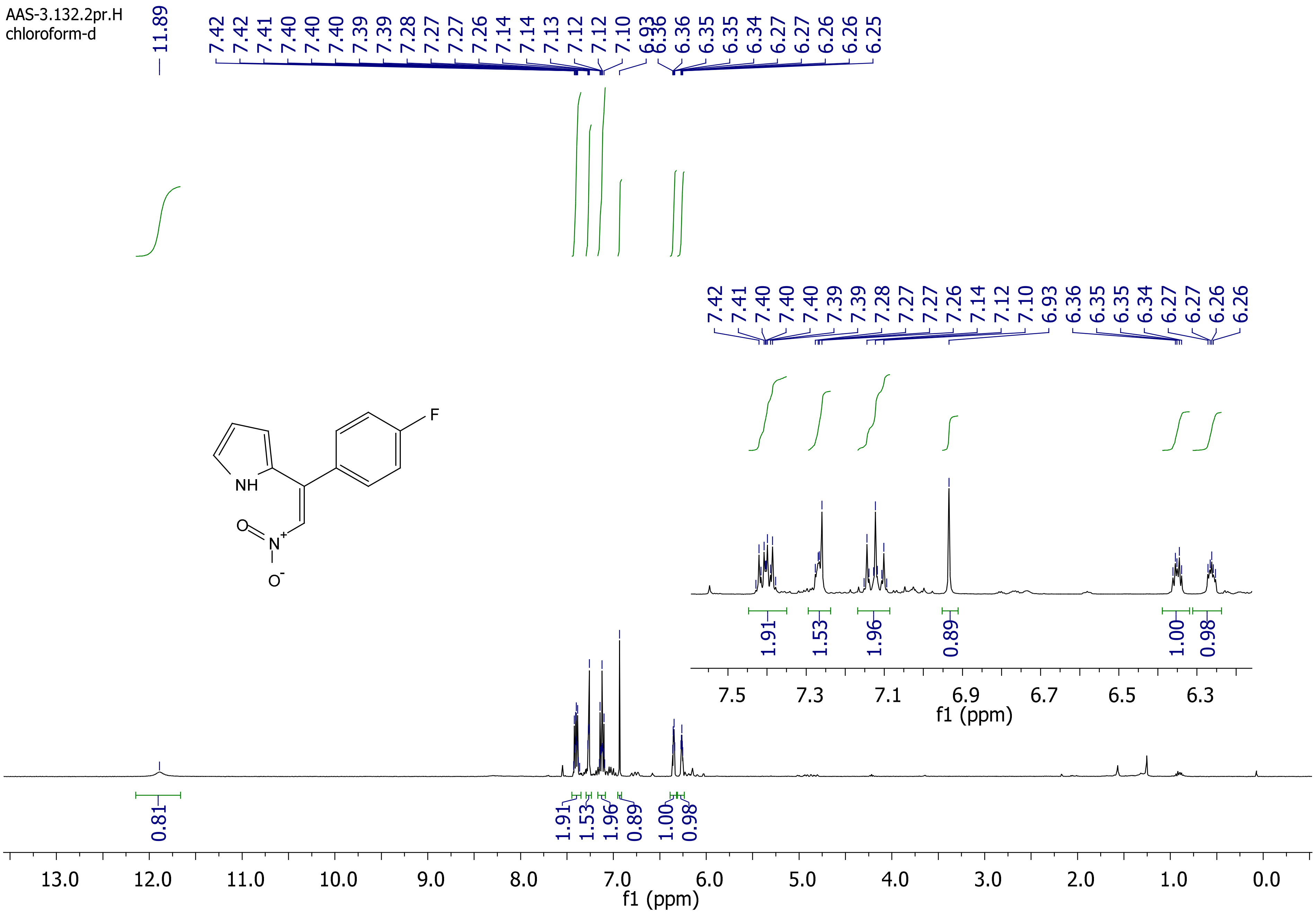

^{1}H NMR spectrum of (*Z*)-2-(1-(4-fluorophenyl)-2-nitrovinyl)-1*H*-pyrrole (*Z*-**5e**)

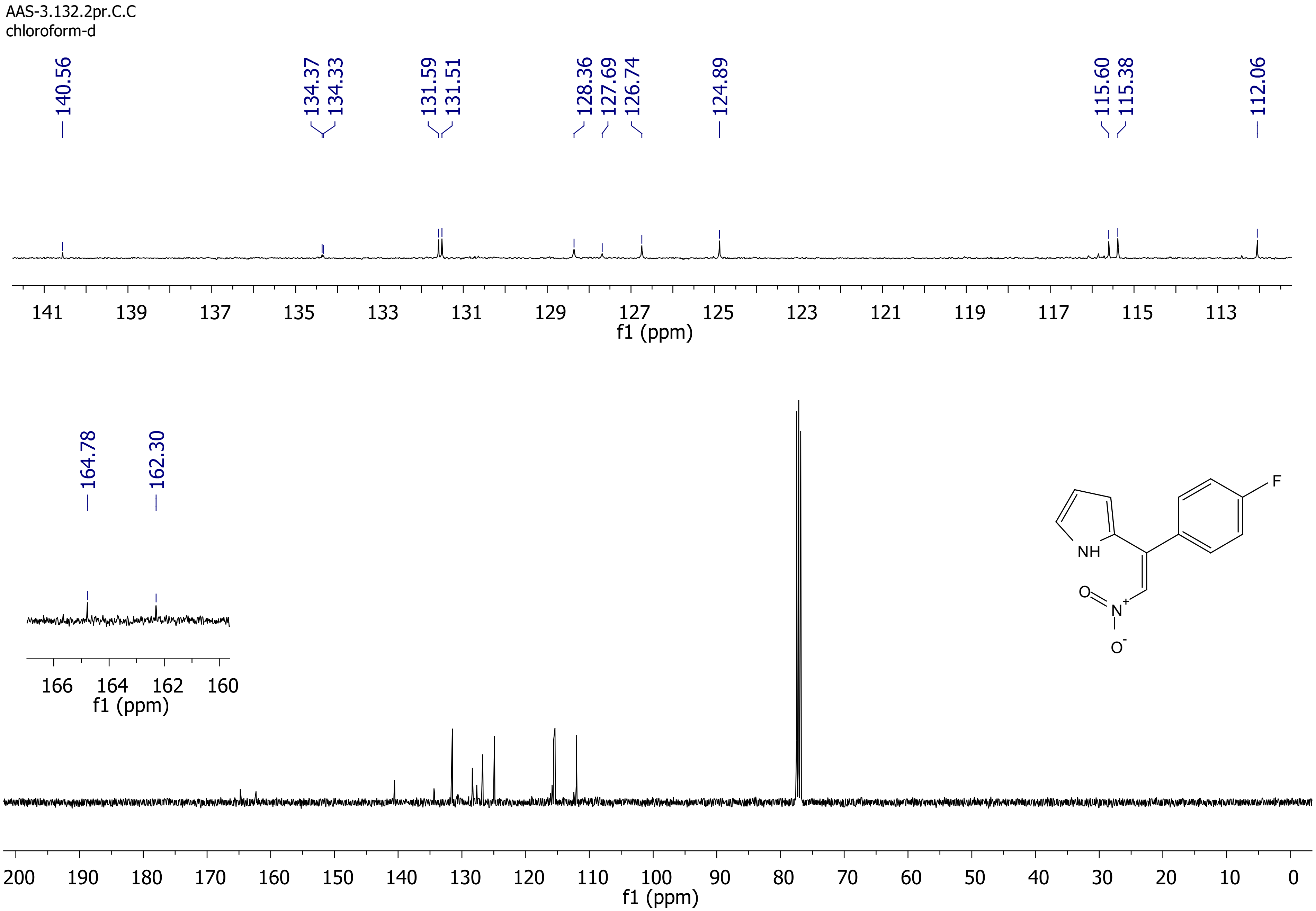

^{13}C NMR spectrum of (*Z*)-2-(1-(4-fluorophenyl)-2-nitrovinyl)-1*H*-pyrrole (*Z*-**5e**)

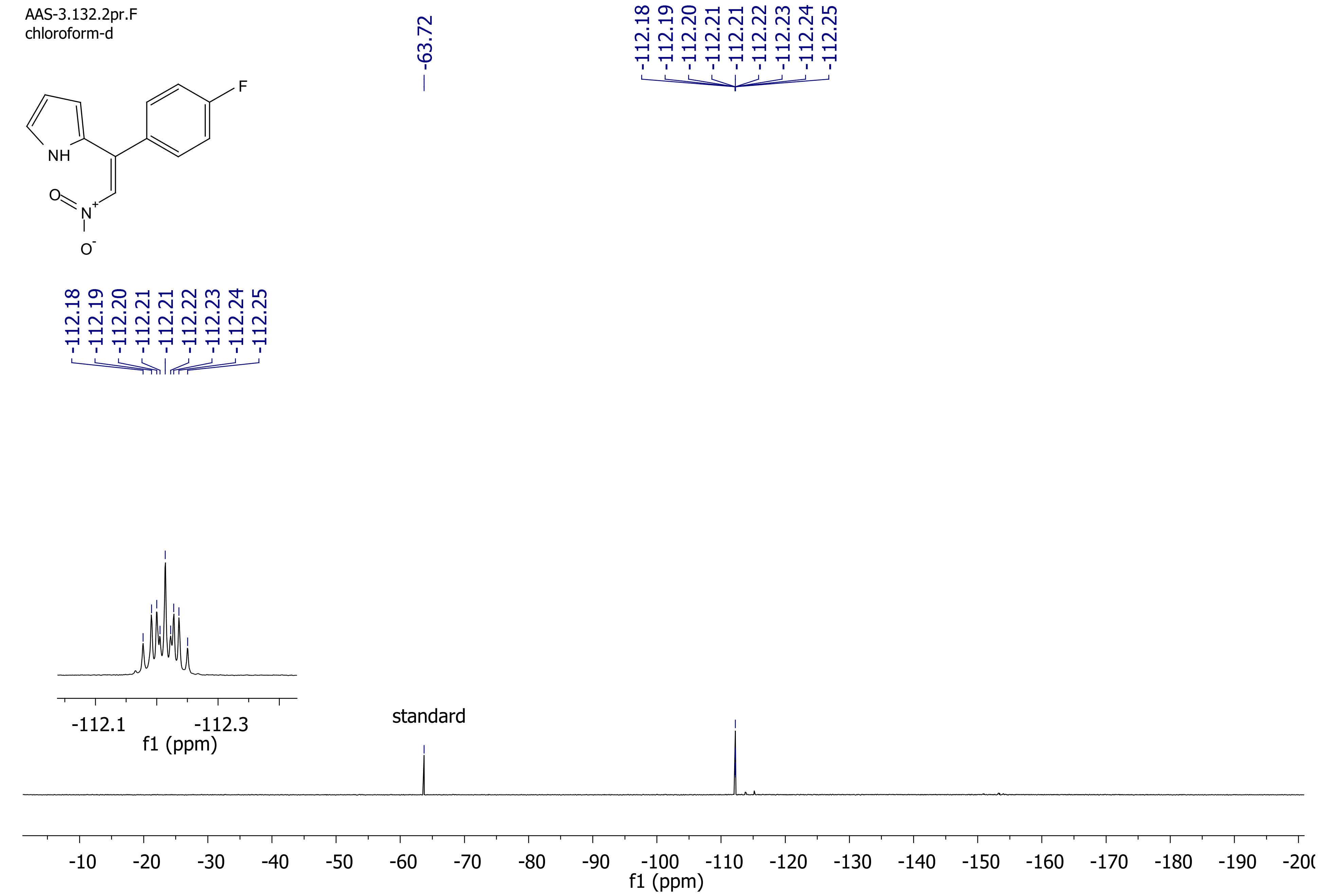

^{19}F NMR spectrum of (*Z*)-2-(1-(4-fluorophenyl)-2-nitrovinyl)-1*H*-pyrrole (*Z*-**5e**)

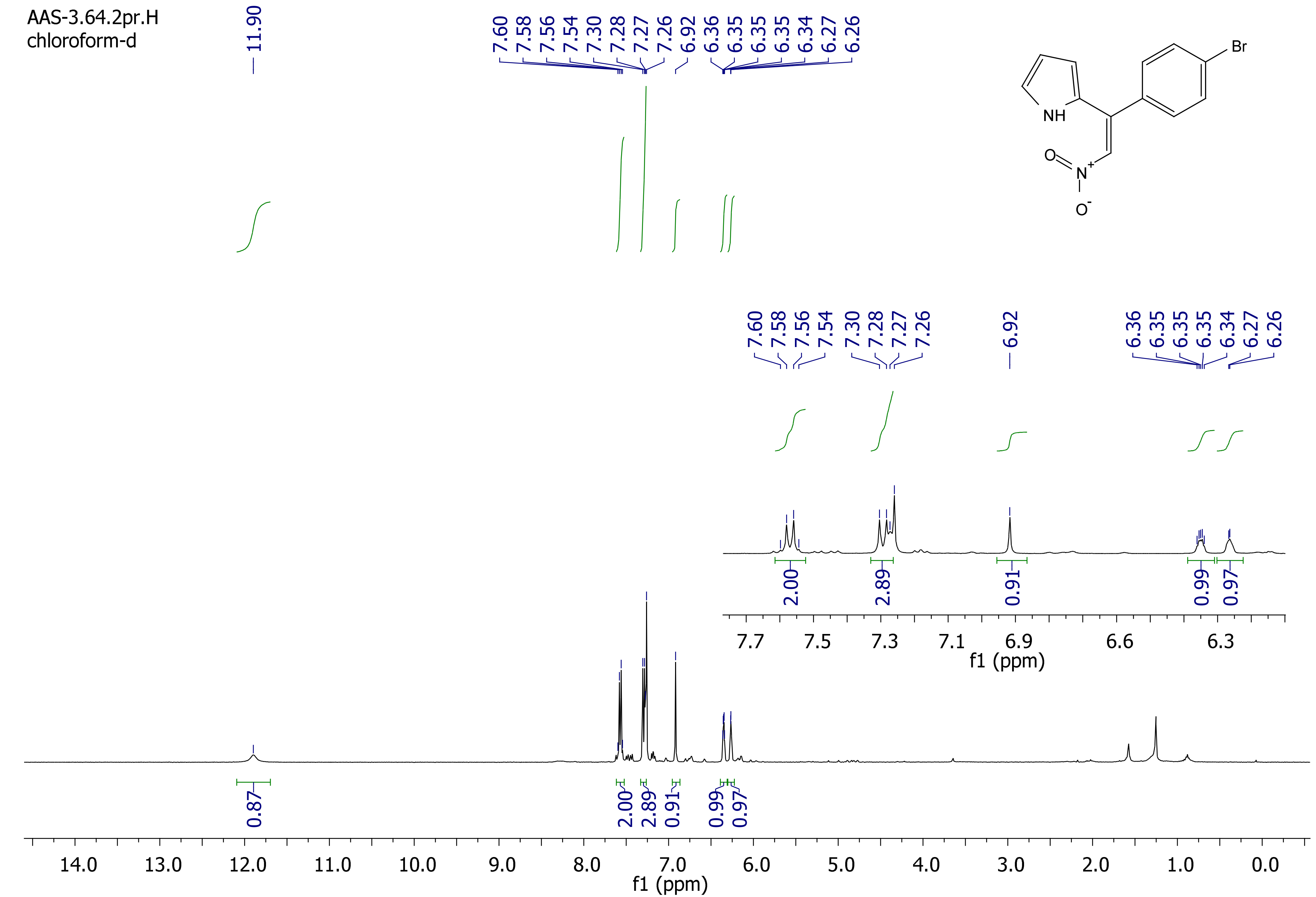

^{1}H NMR spectrum of (*Z*)-2-(1-(4-bromophenyl)-2-nitrovinyl)-1*H*-pyrrole (*Z*-**5f**)

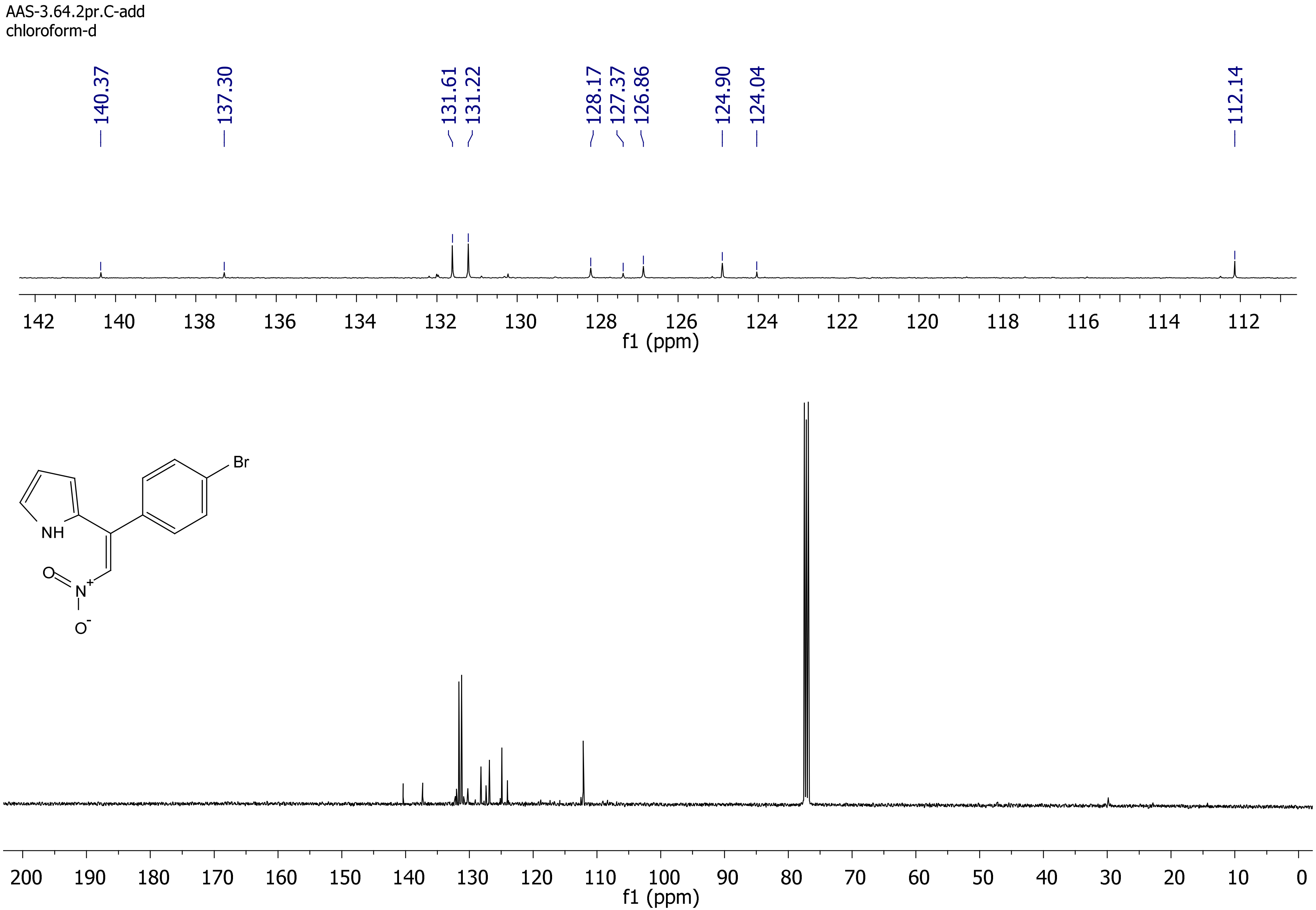

^{13}C NMR spectrum of (*Z*)-2-(1-(4-bromophenyl)-2-nitrovinyl)-1*H*-pyrrole (*Z*-**5f**)

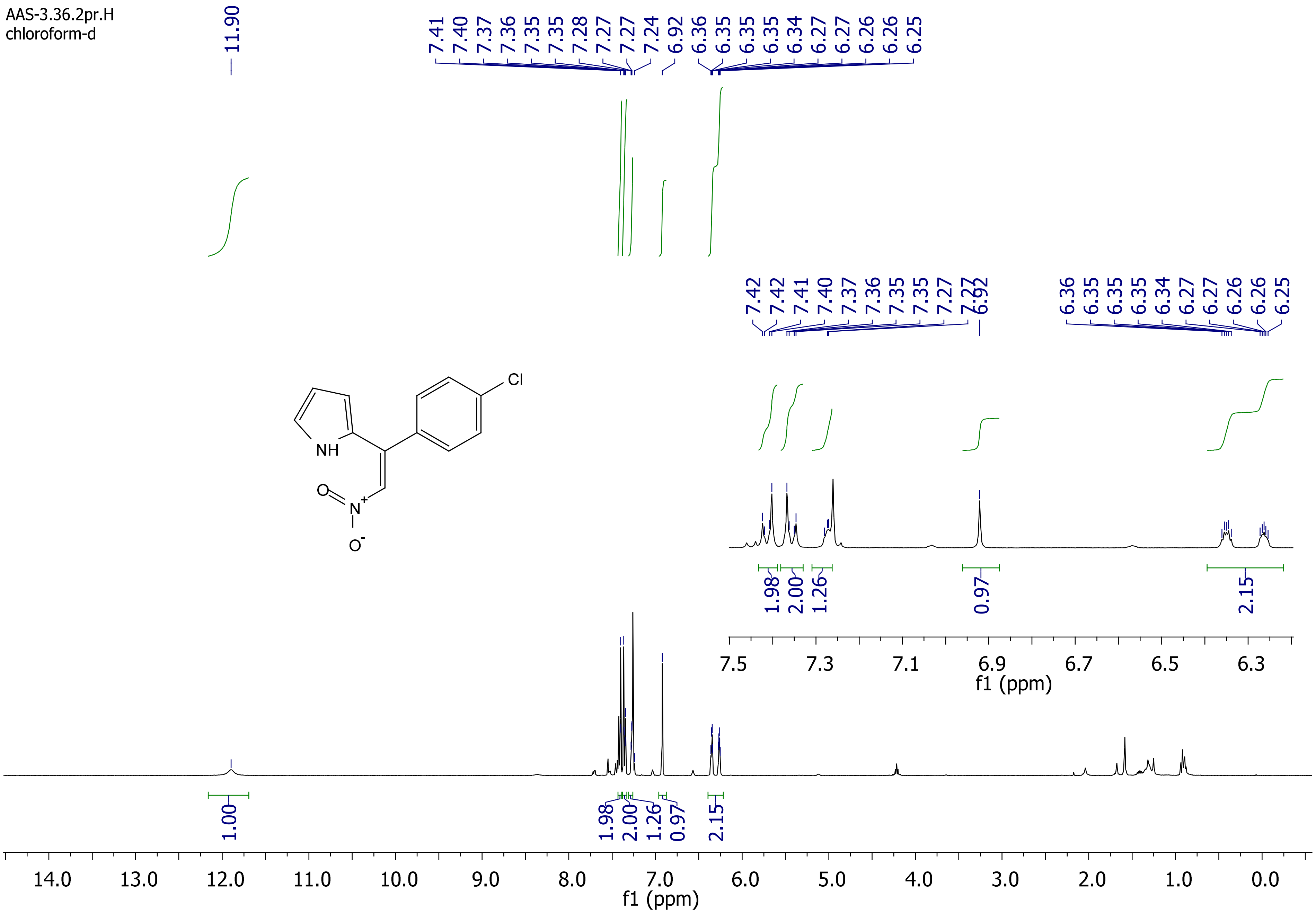

^{1}H NMR spectrum of (*Z*)-2-(1-(4-chlorophenyl)-2-nitrovinyl)-1*H*-pyrrole (*Z*-**5g**)

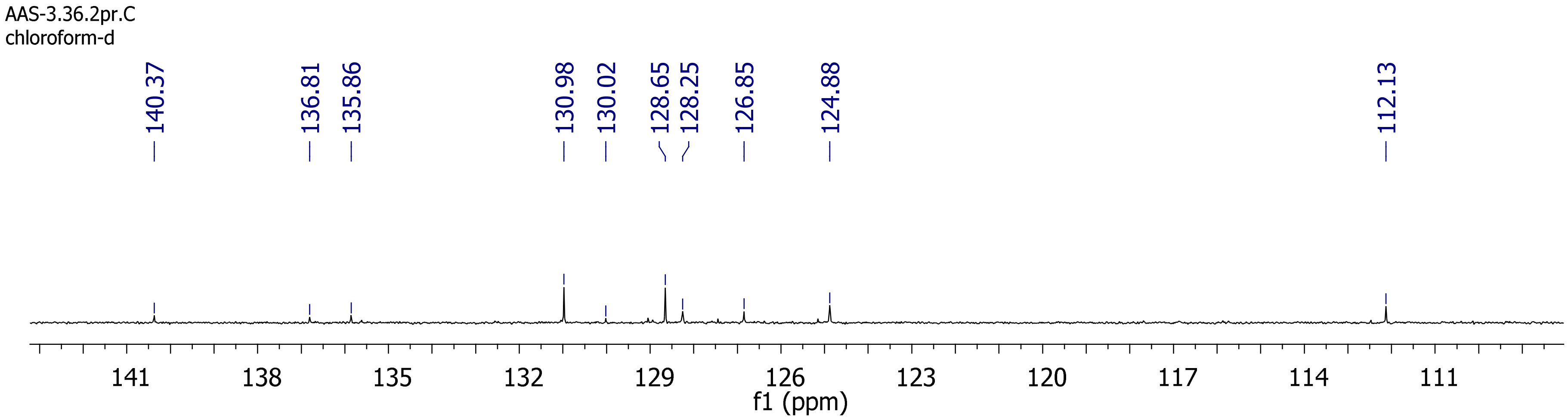

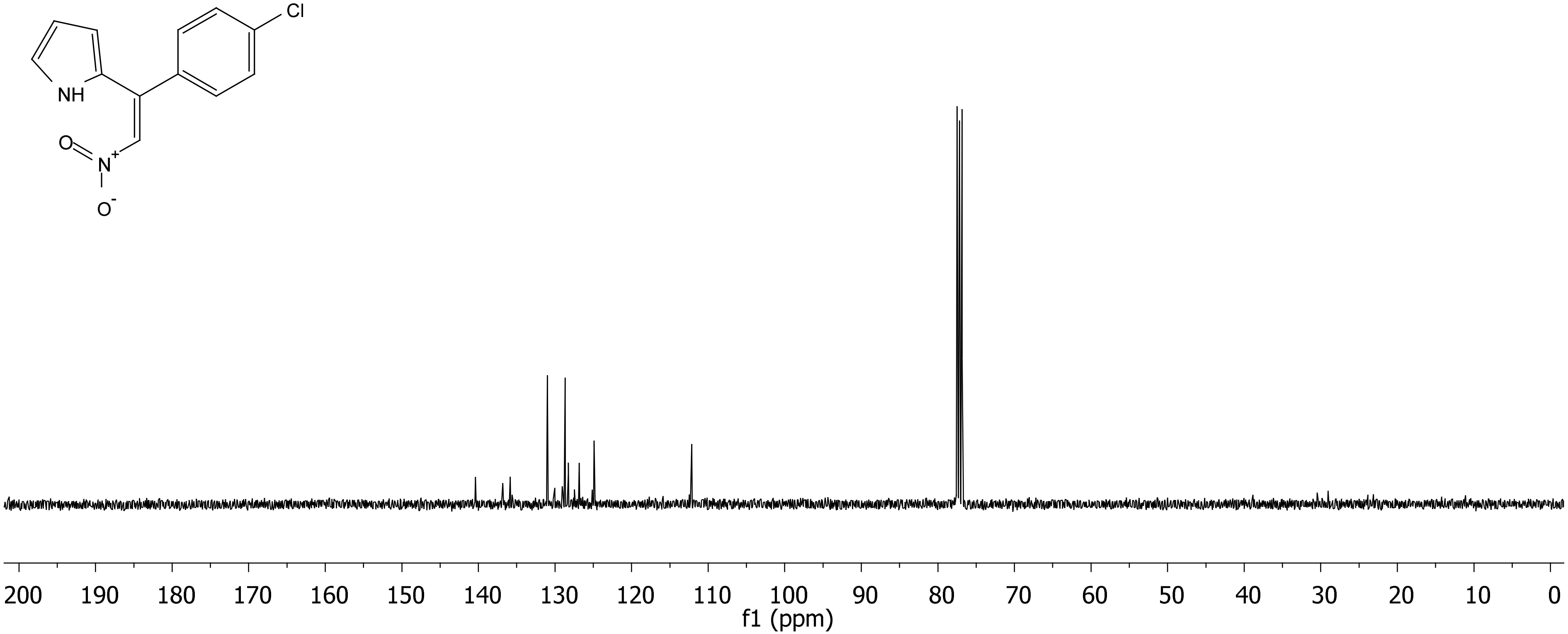

^{13}C NMR spectrum of (*Z*)-2-(1-(4-chlorophenyl)-2-nitrovinyl)-1*H*-pyrrole (*Z*-**5g**)

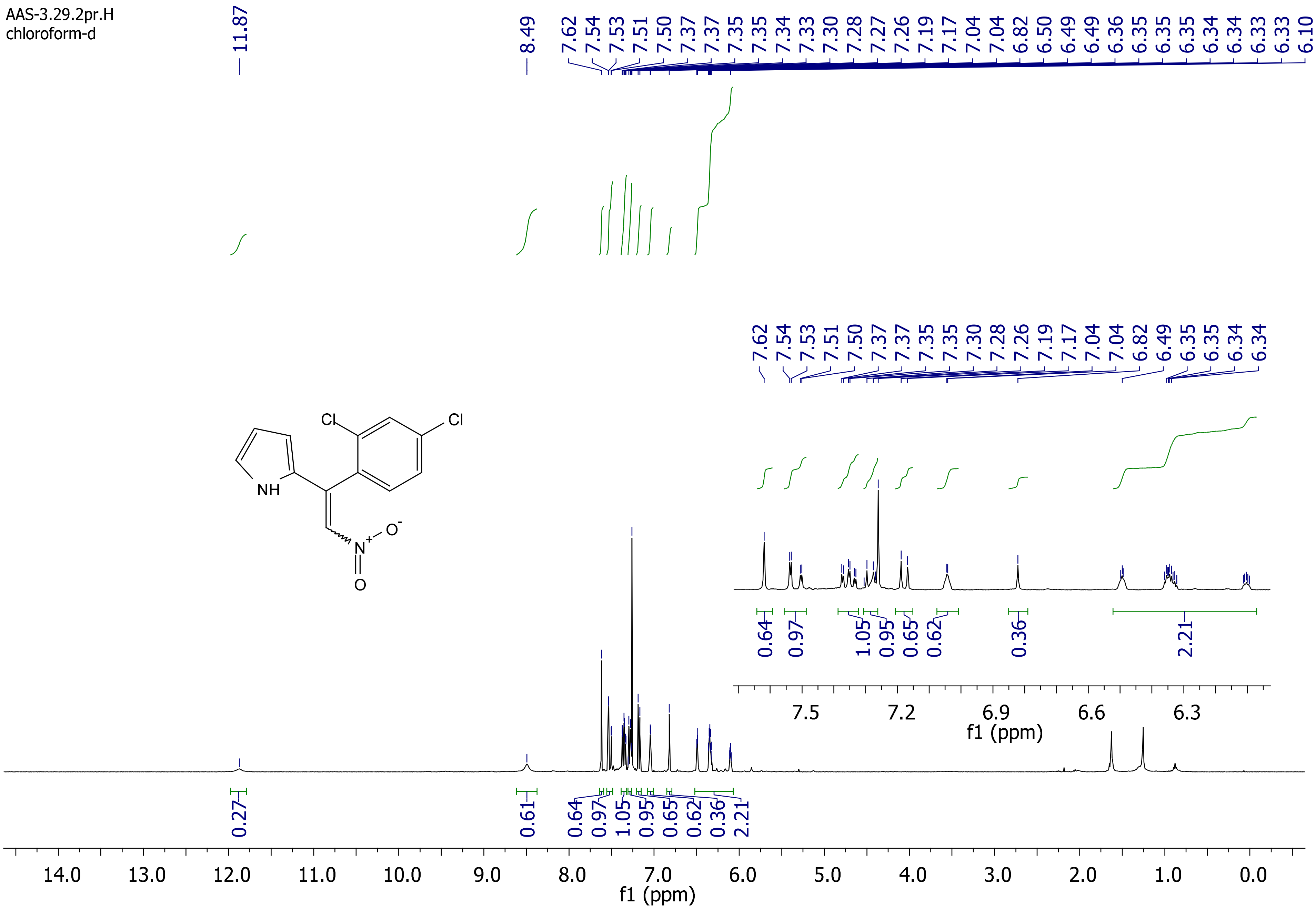

^{1}H NMR spectrum of 2-(1-(2,4-dichlorophenyl)-2-nitrovinyl)-1*H*-pyrrole (**5h**)

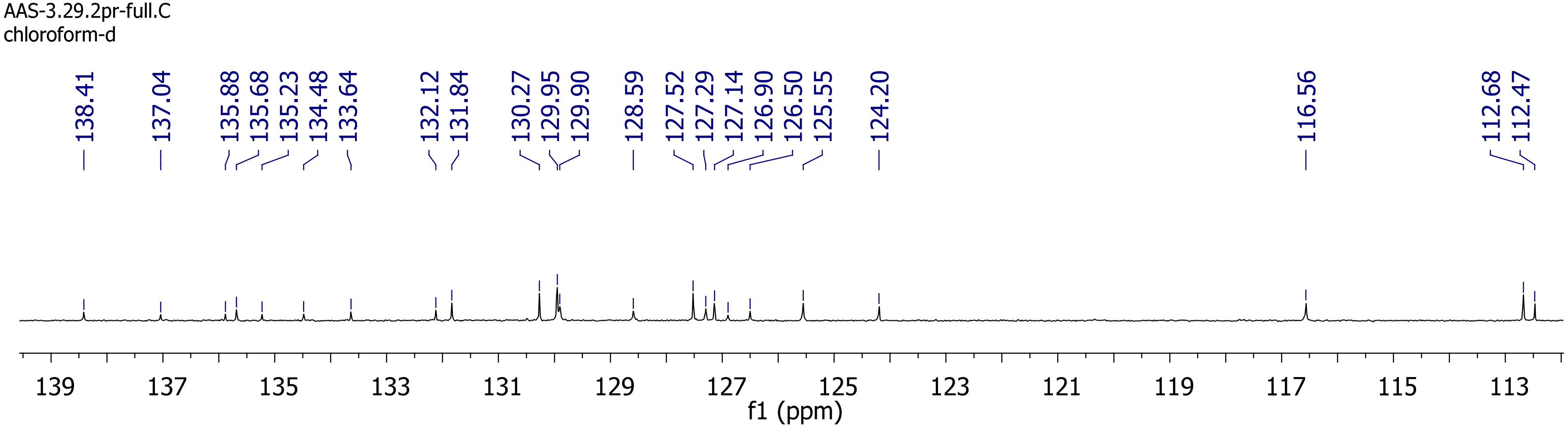

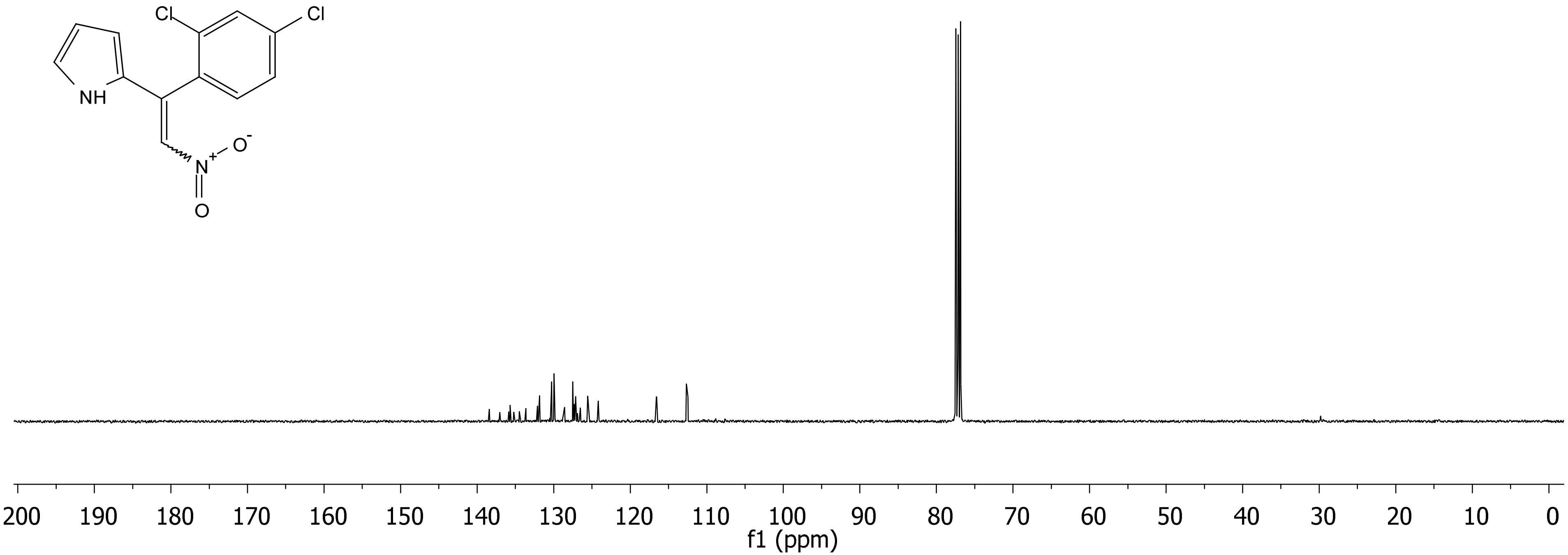

^{13}C NMR spectrum of 2-(1-(2,4-dichlorophenyl)-2-nitrovinyl)-1*H*-pyrrole (**5h**)

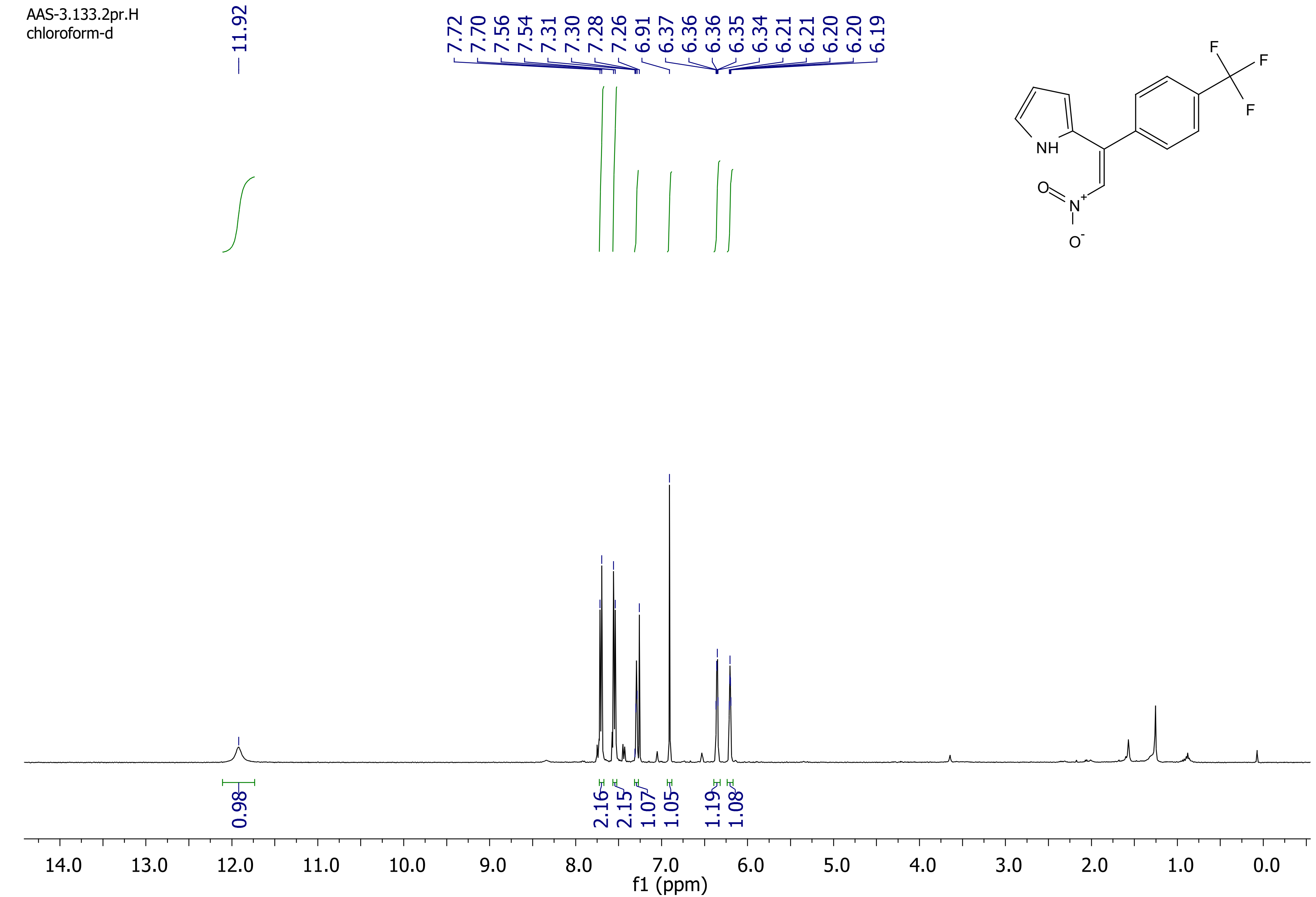

^{1}H NMR spectrum of (*Z*)-2-(2-nitro-1-(4-(trifluoromethyl)phenyl)vinyl)-1*H*-pyrrole (*Z*-**5i**)

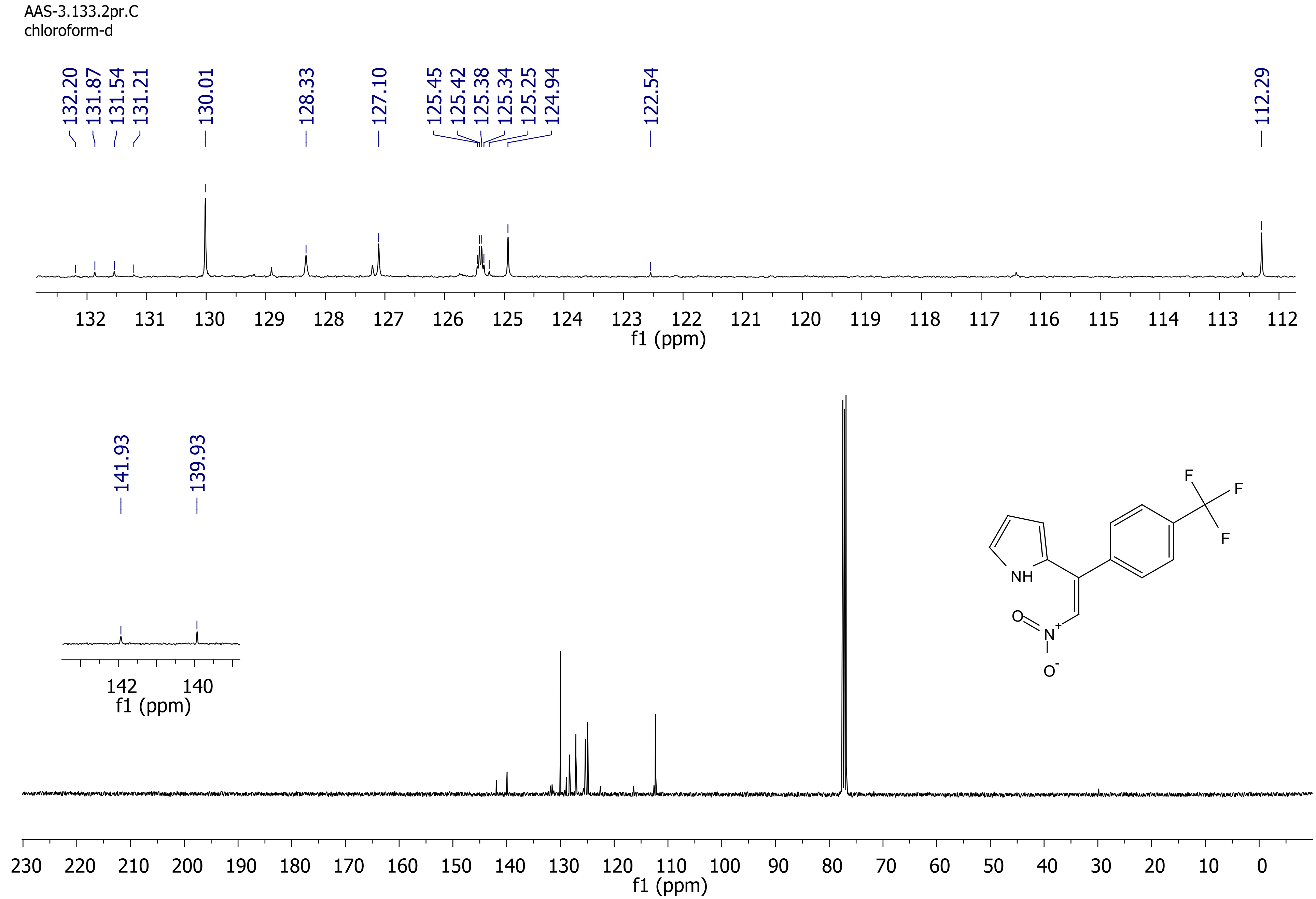

^{13}C NMR spectrum of (*Z*)-2-(2-nitro-1-(4-(trifluoromethyl)phenyl)vinyl)-1*H*-pyrrole (*Z*-**5i**)

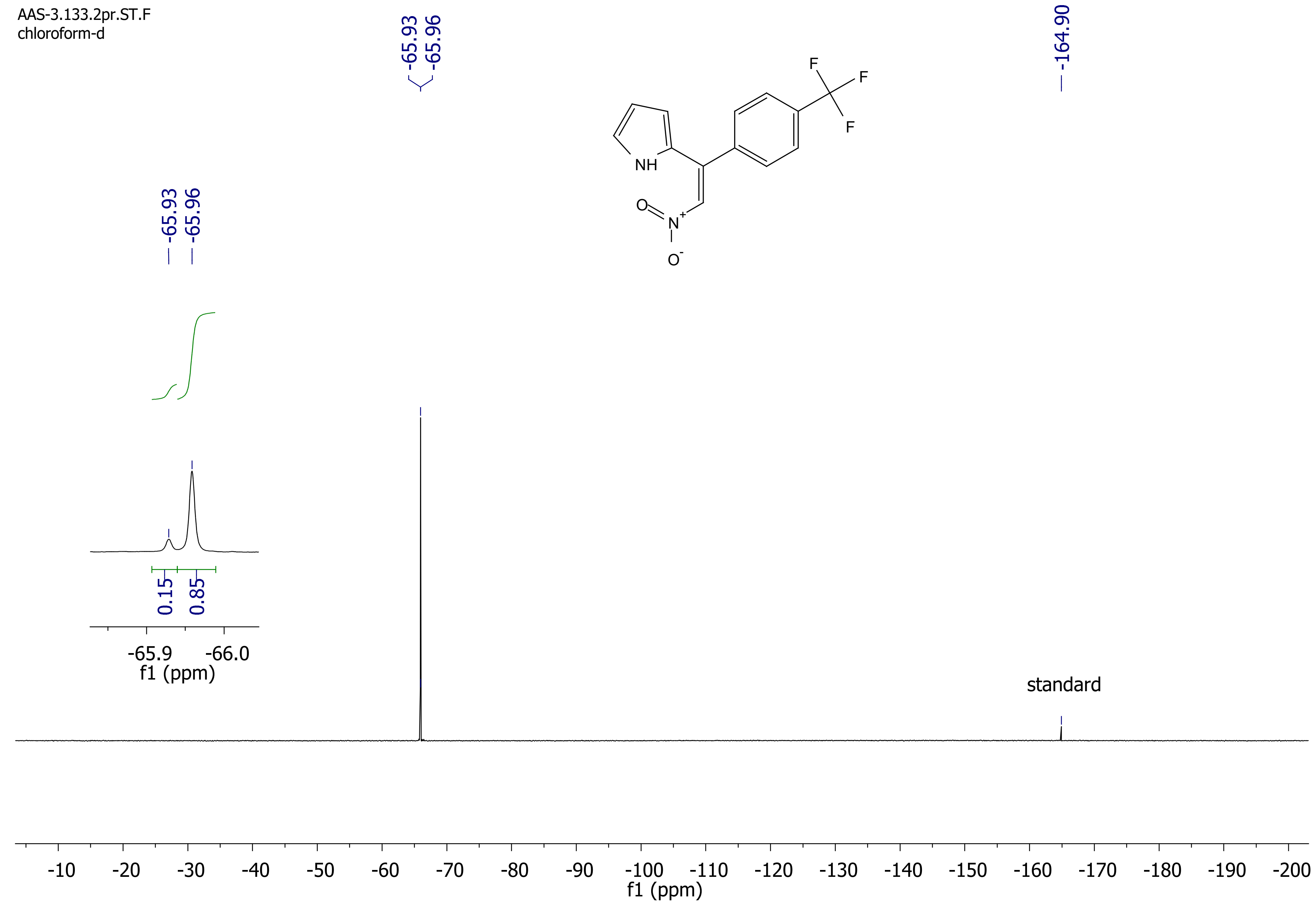

^{19}F NMR spectrum of (*Z*)-2-(2-nitro-1-(4-(trifluoromethyl)phenyl)vinyl)-1*H*-pyrrole (*Z*-**5i**)

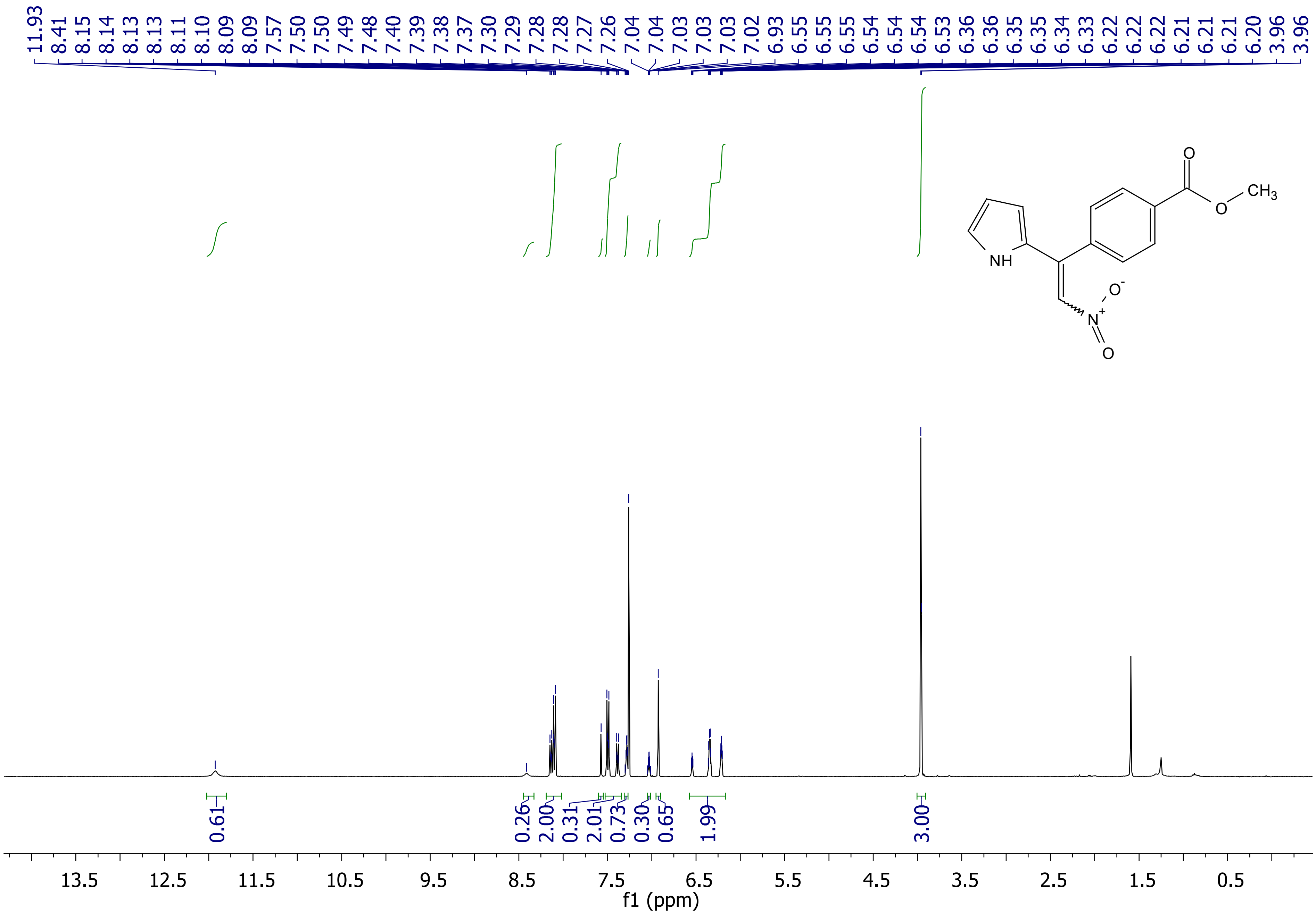

^{1}H NMR spectrum of methyl 4-(2-nitro-1-(1*H*-pyrrol-2-yl)vinyl)benzoate (**5j**)

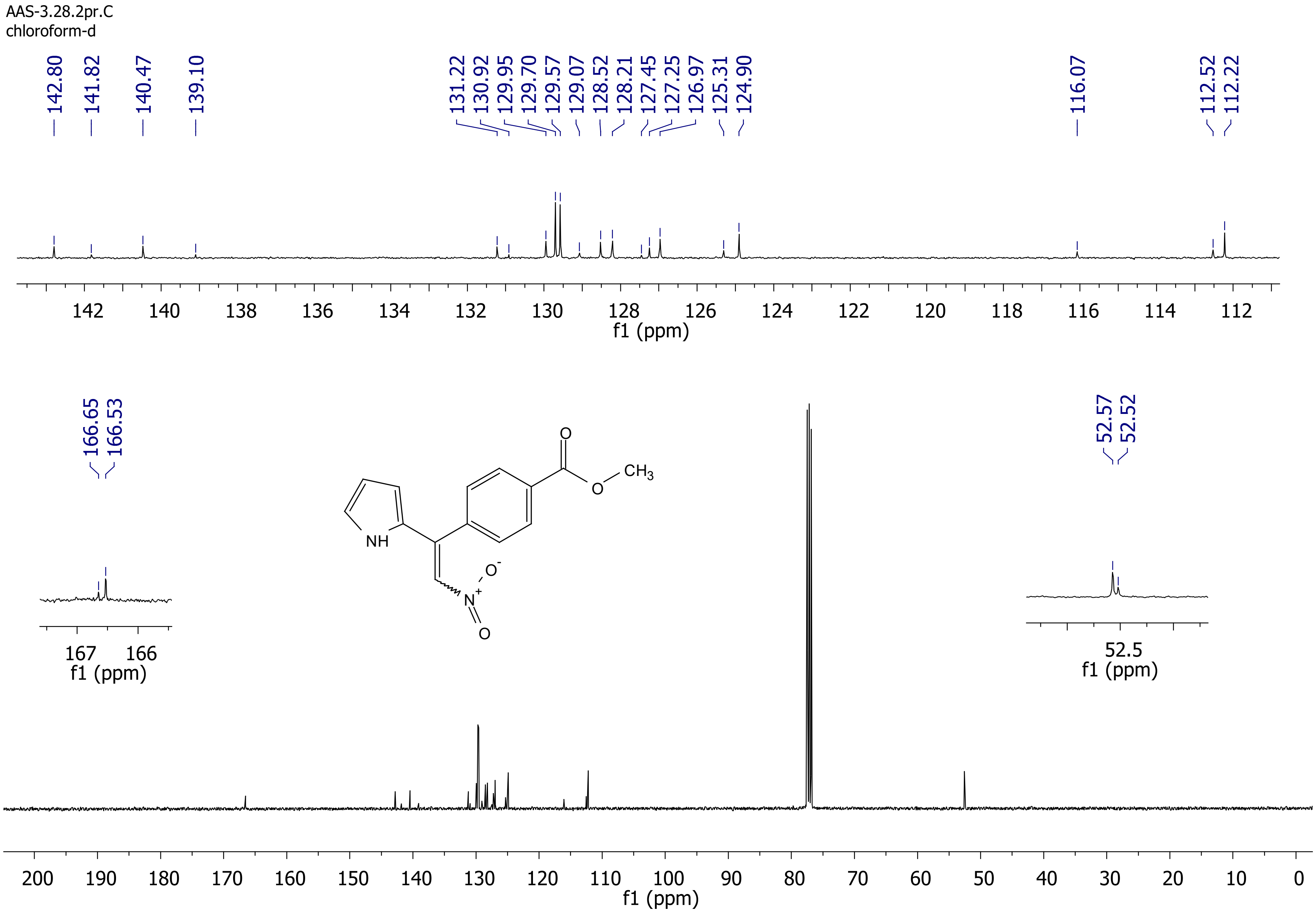

^{13}C NMR spectrum of methyl 4-(2-nitro-1-(1*H*-pyrrol-2-yl)vinyl)benzoate (**5j**)

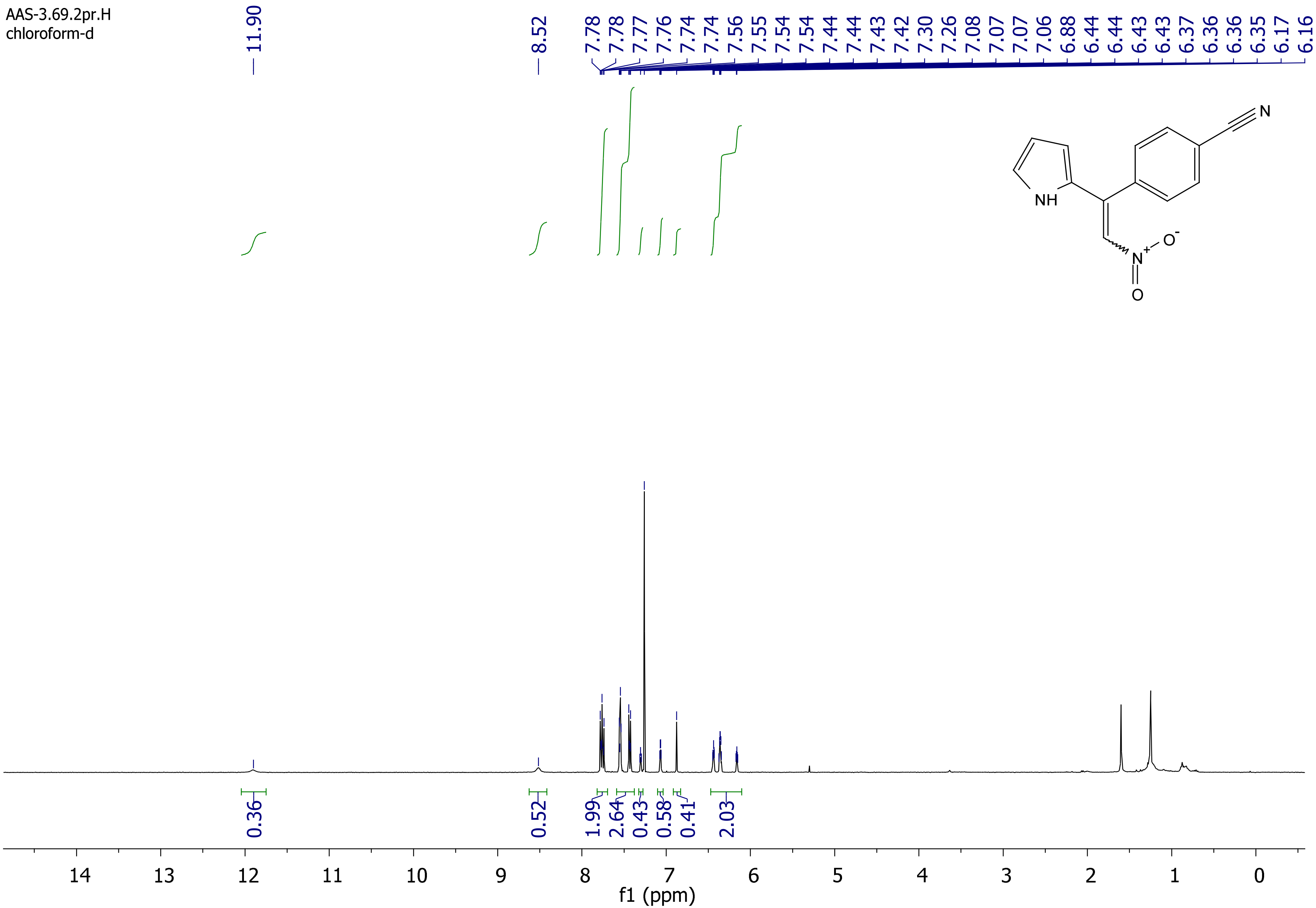

^{1}H NMR spectrum of 4-(2-nitro-1-(1*H*-pyrrol-2-yl)vinyl)benzonitrile (**5k**)

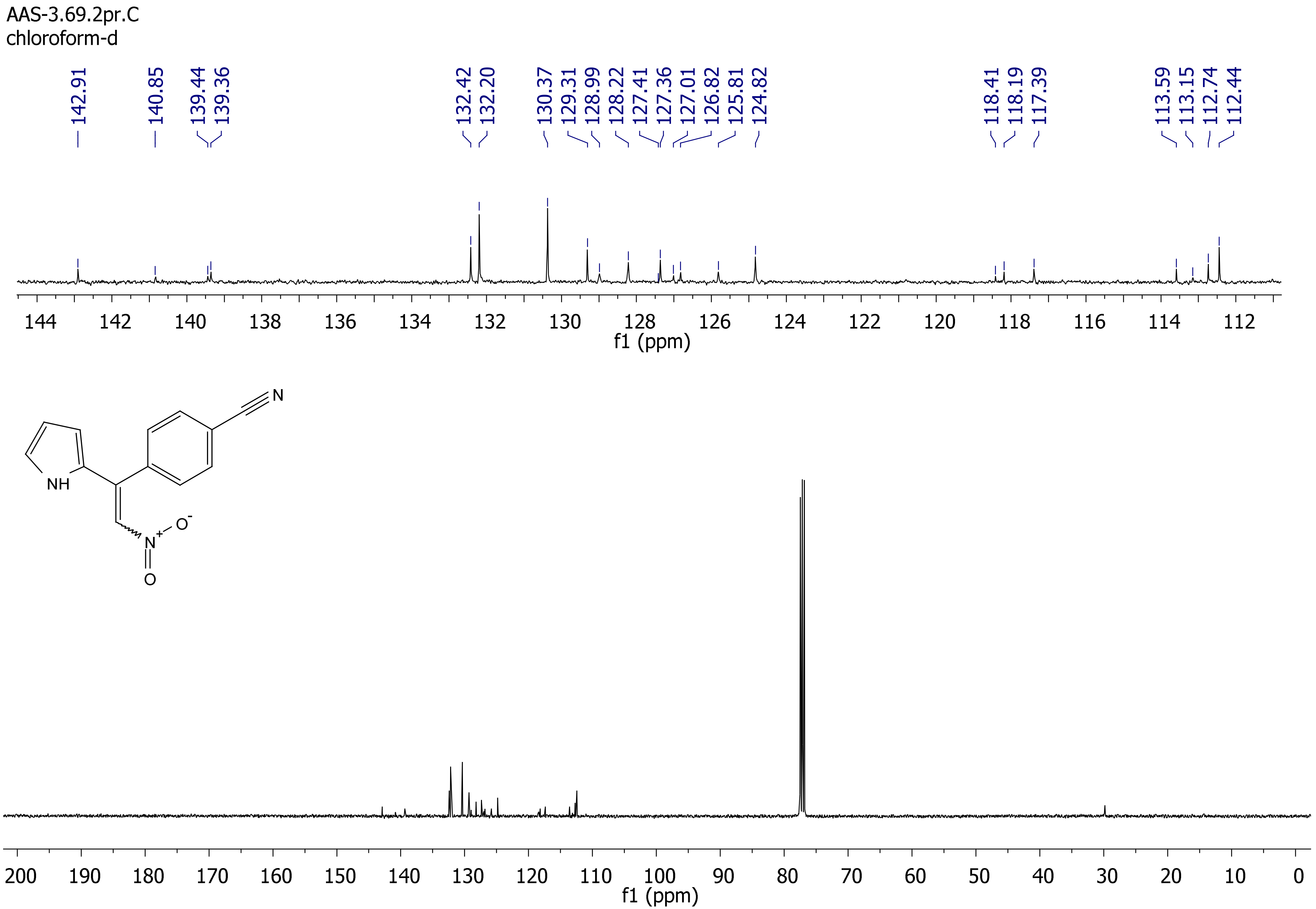

^{13}C NMR spectrum of 4-(2-nitro-1-(1*H*-pyrrol-2-yl)vinyl)benzonitrile (**5k**)

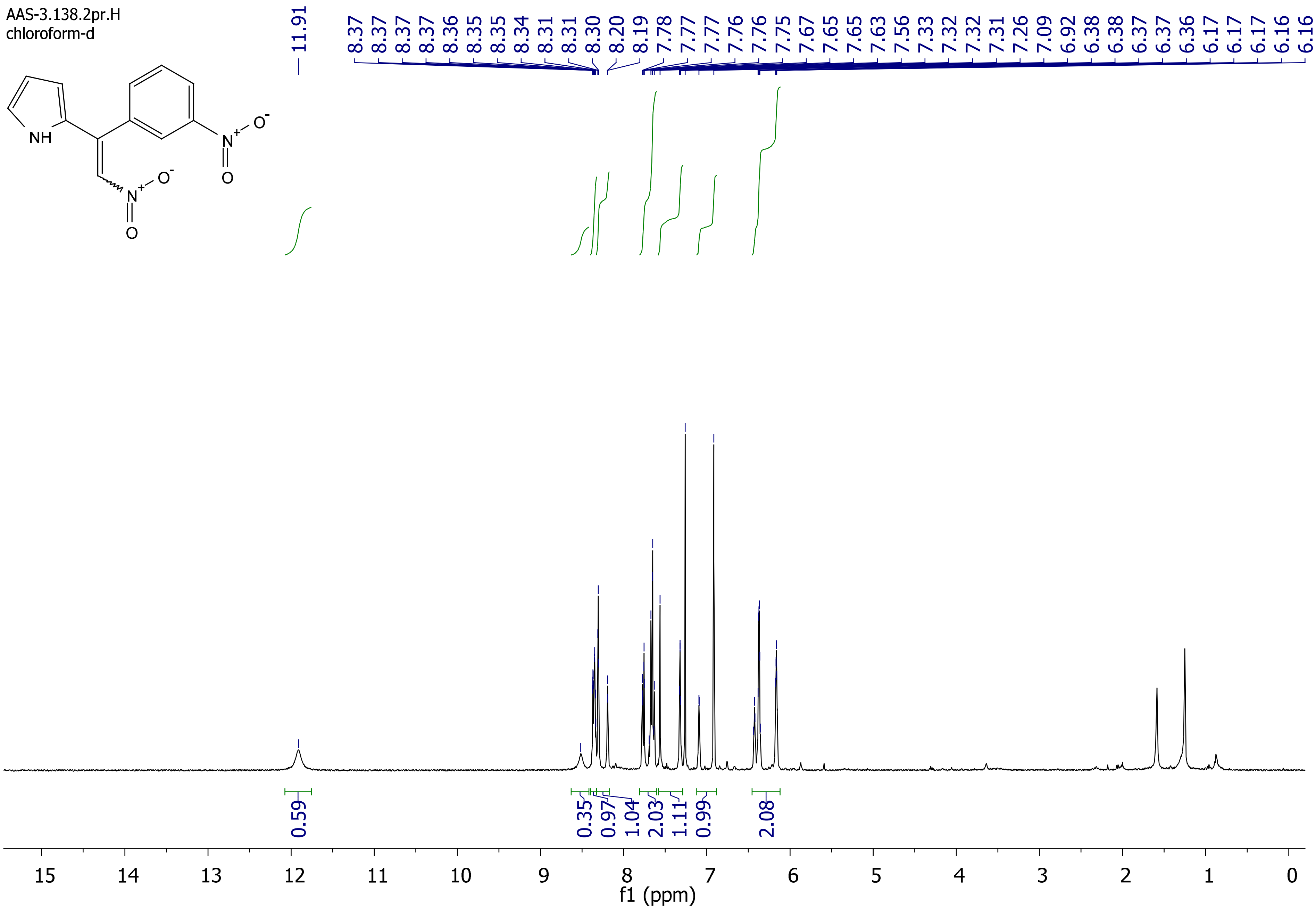

^{1}H NMR spectrum of 2-(2-nitro-1-(3-nitrophenyl)vinyl)-1*H*-pyrrole (**5m**)

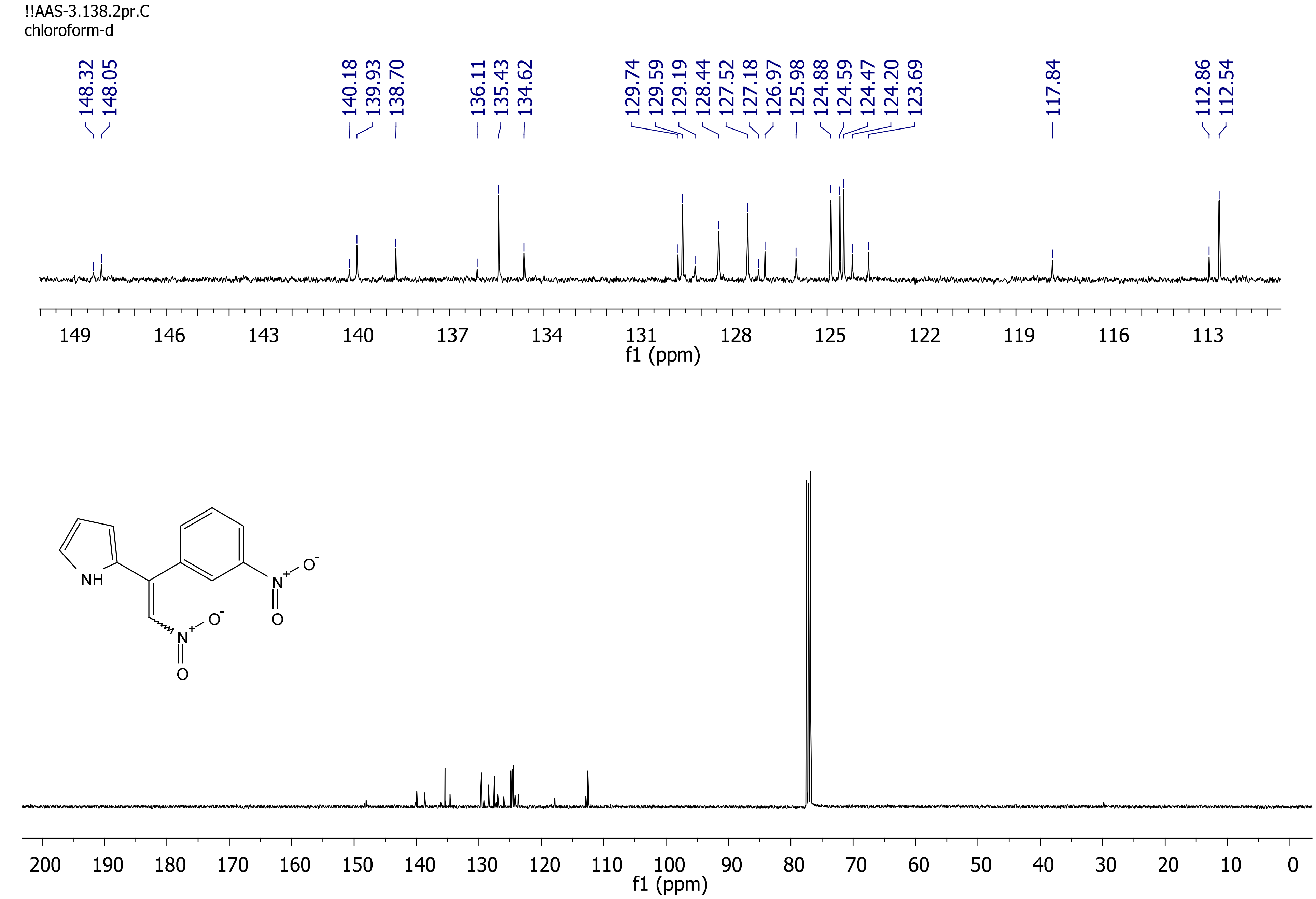

^{13}C NMR spectrum of 2-(2-nitro-1-(3-nitrophenyl)vinyl)-1*H*-pyrrole (**5m**)

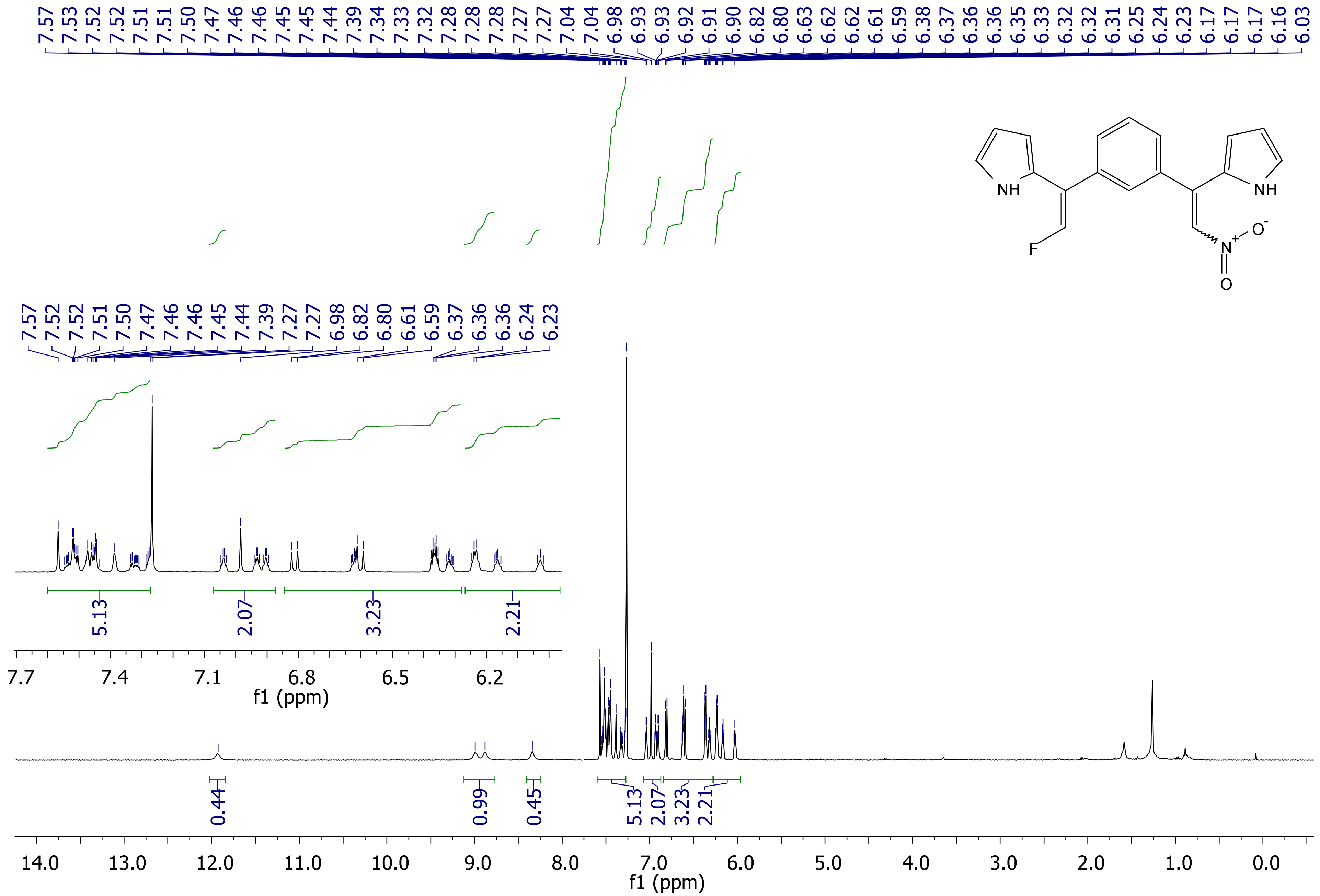

^{1}H NMR spectrum of 2-(1-(3-((*Z*)-2-fluoro-1-(1*H*-pyrrol-2-yl)vinyl)phenyl)-2-nitrovinyl)-1*H*-pyrrole (*Z*-F-**5o**)

AAS-3.117.6bfr
chloroform-d

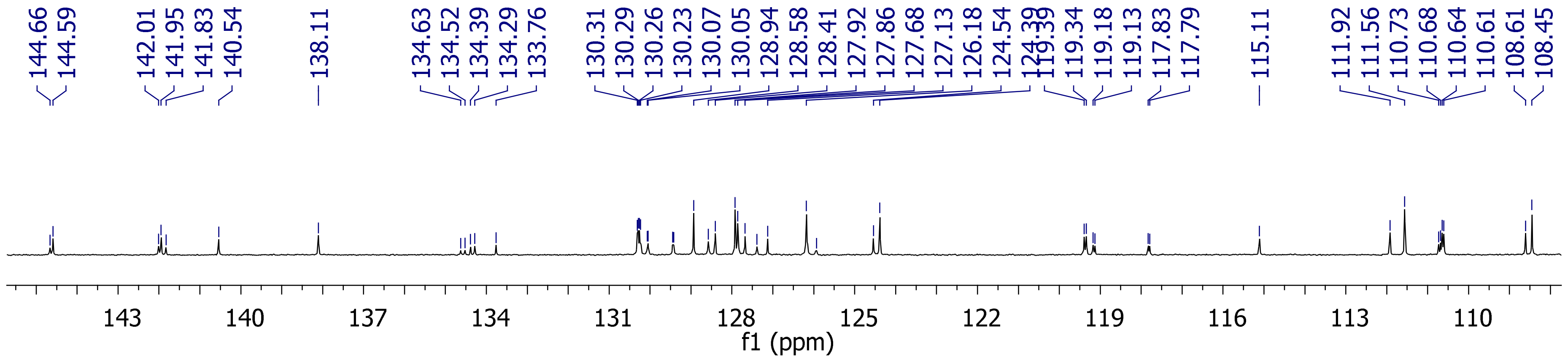

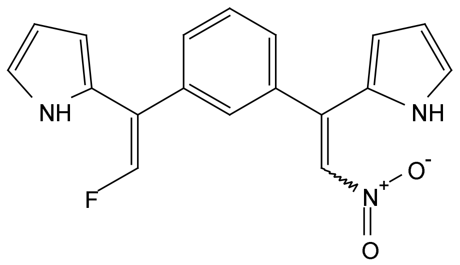

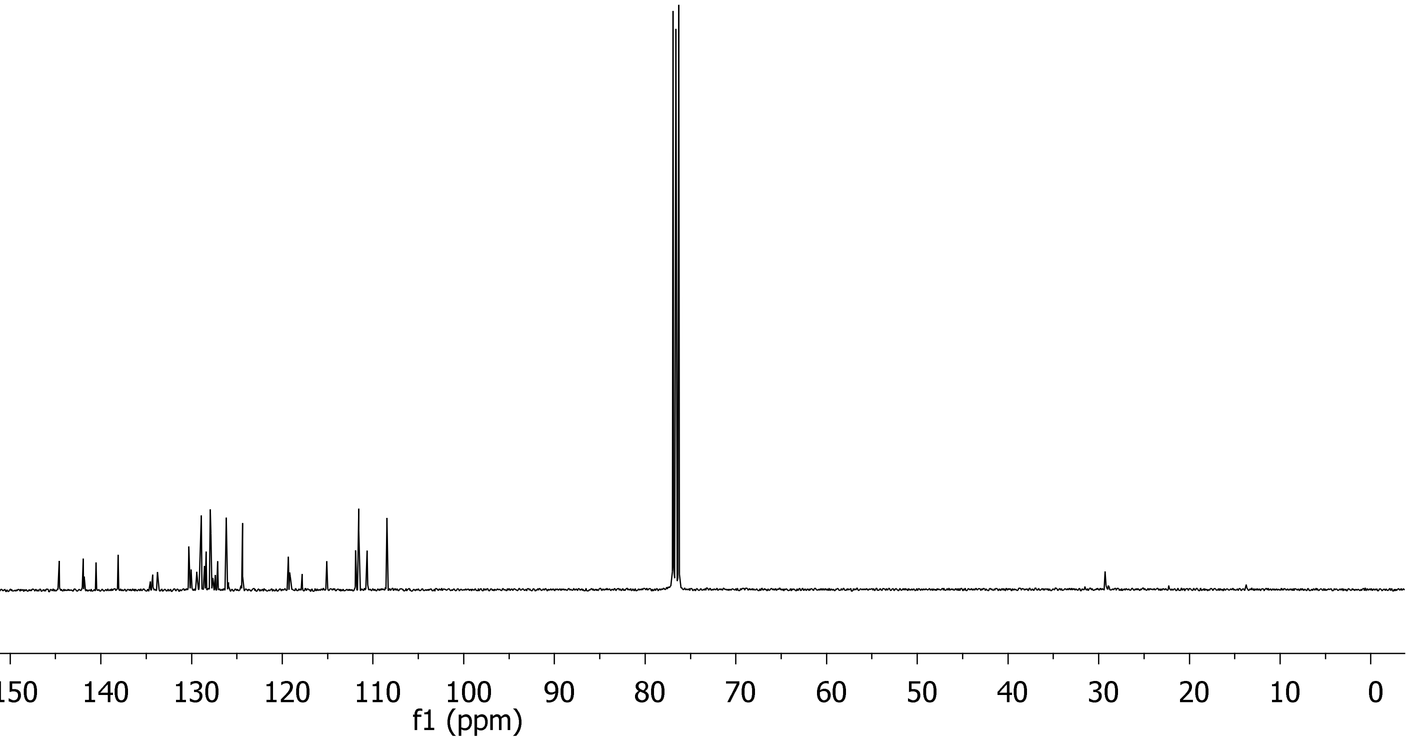

^{13}C NMR spectrum of f 2-(1-(3-((Z)-2-fluoro-1-(1*H*-pyrrol-2-yl)vinyl)phenyl)-2-nitrovinyl)-1*H*-pyrrole (*Z*-F-**5o**)

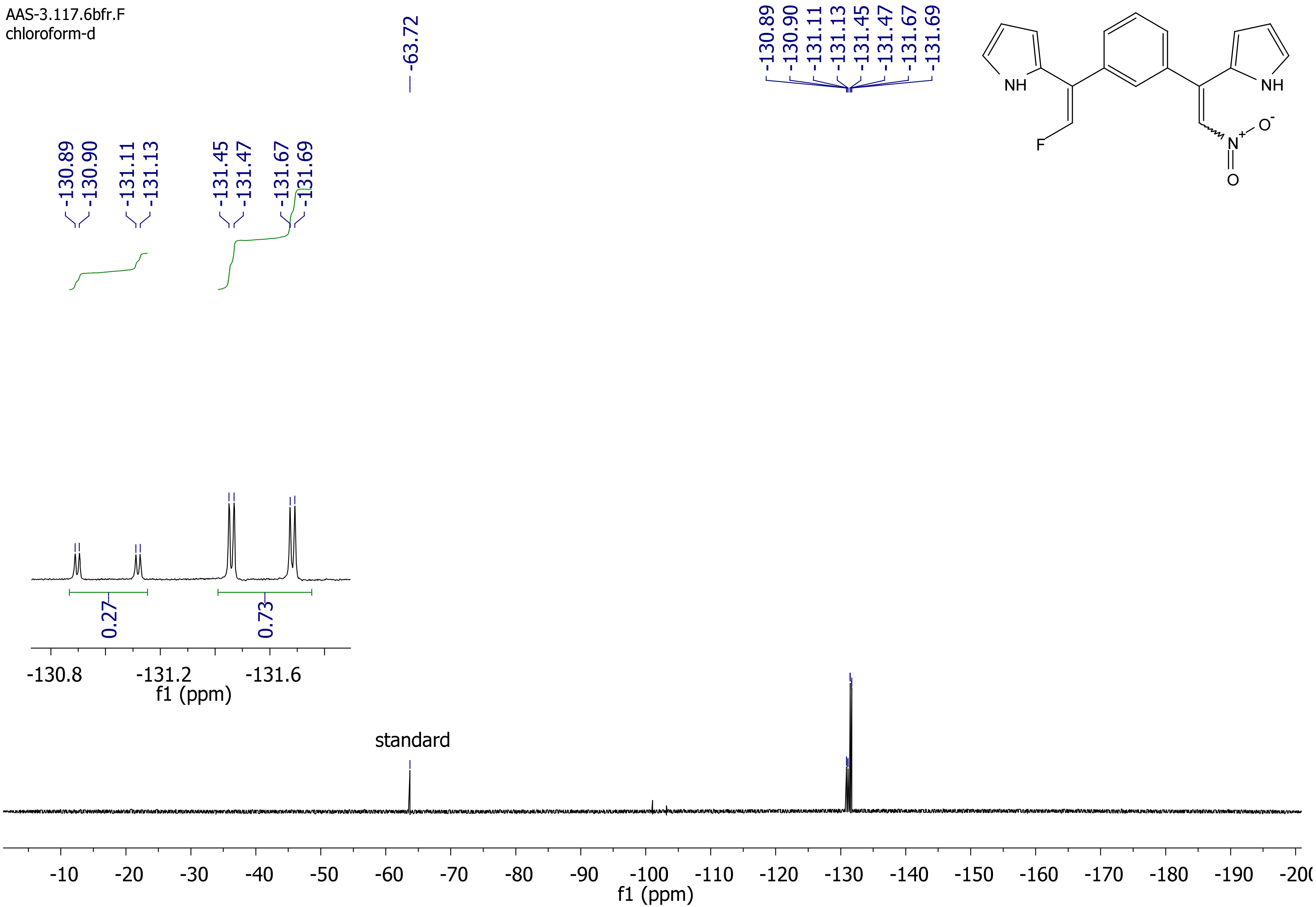

^{19}F NMR spectrum of 2-(1-(3-((Z)-2-fluoro-1-(1*H*-pyrrol-2-yl)vinyl)phenyl)-2-nitrovinyl)-1*H*-pyrrole (*Z*-F-**5o**)

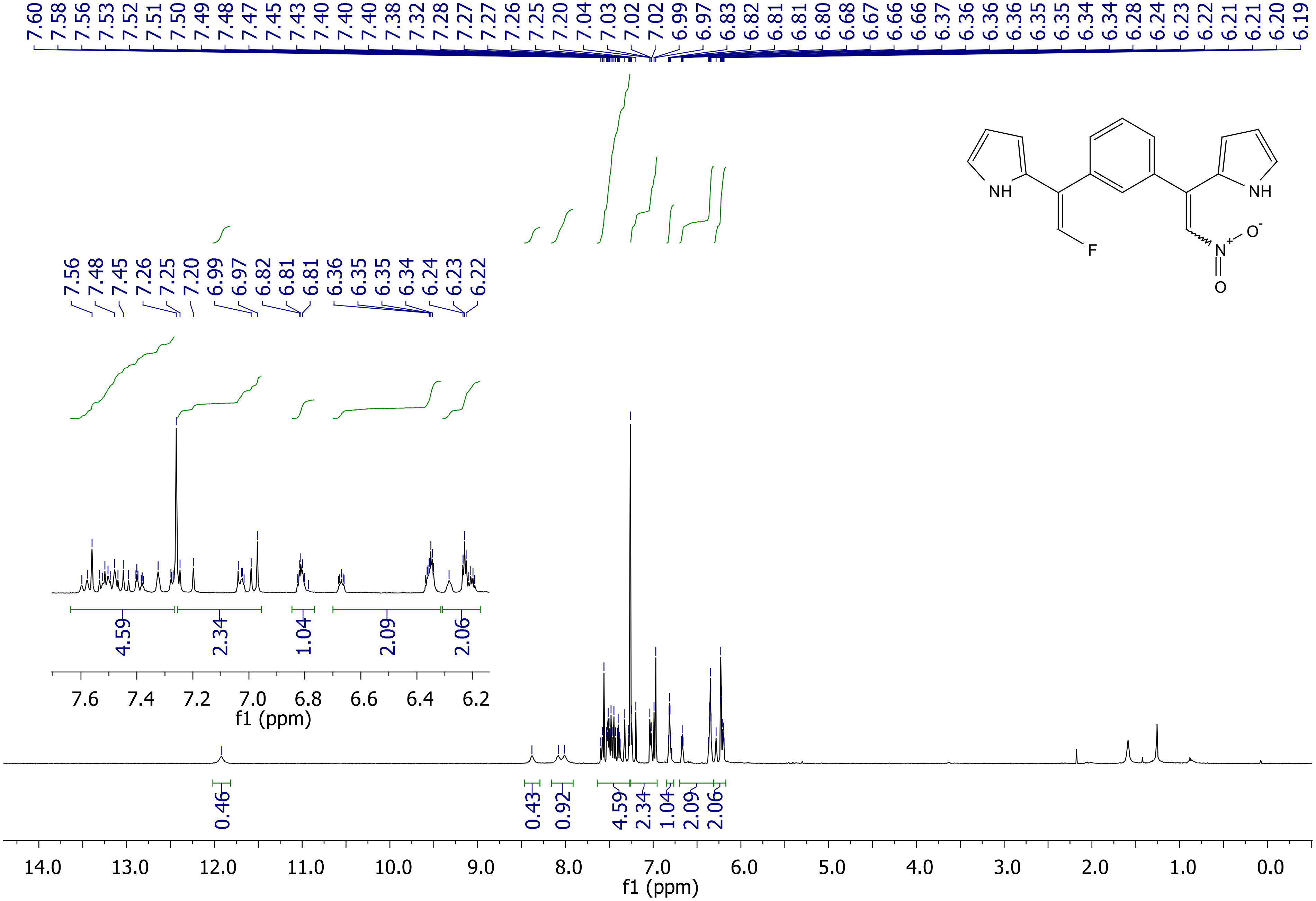

^{1}H NMR spectrum of 2-(1-(3-((*E*)-2-fluoro-1-(1*H*-pyrrol-2-yl)vinyl)phenyl)-2-nitrovinyl)-1*H*-pyrrole (*E*-F-**5o**)

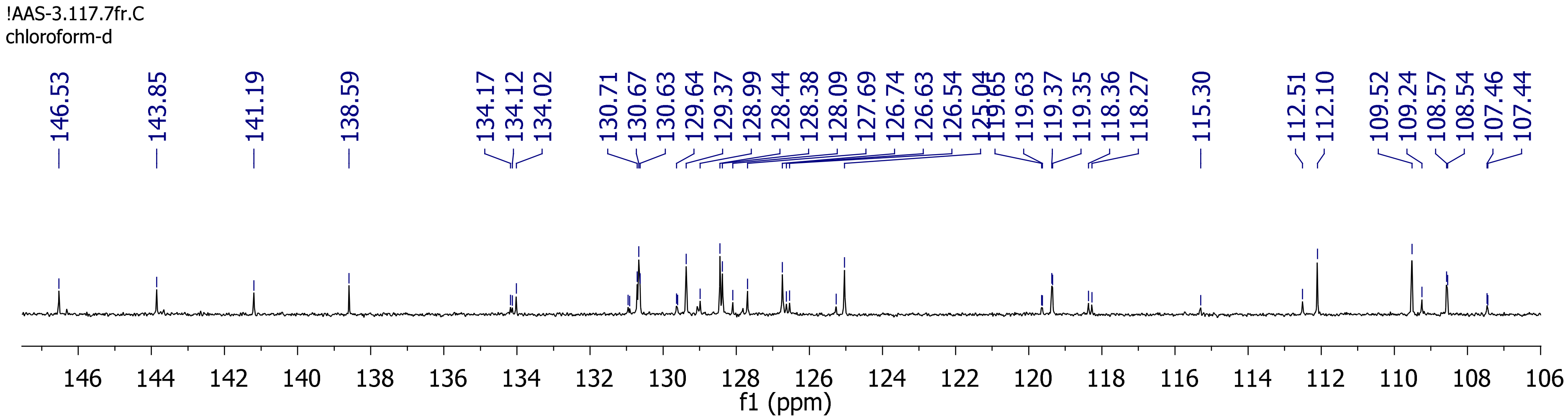

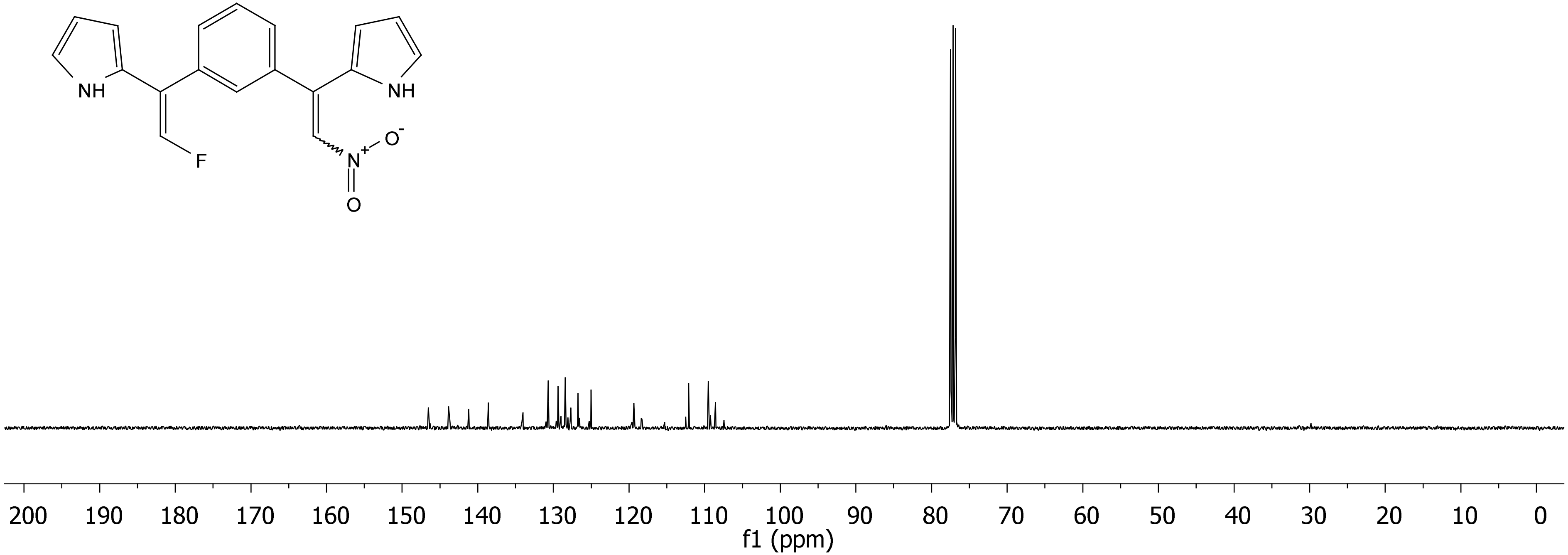

^{13}C NMR spectrum of 2-(1-(3-((*E*)-2-fluoro-1-(1*H*-pyrrol-2-yl)vinyl)phenyl)-2-nitrovinyl)-1*H*-pyrrole (*E*-F-**5o**)

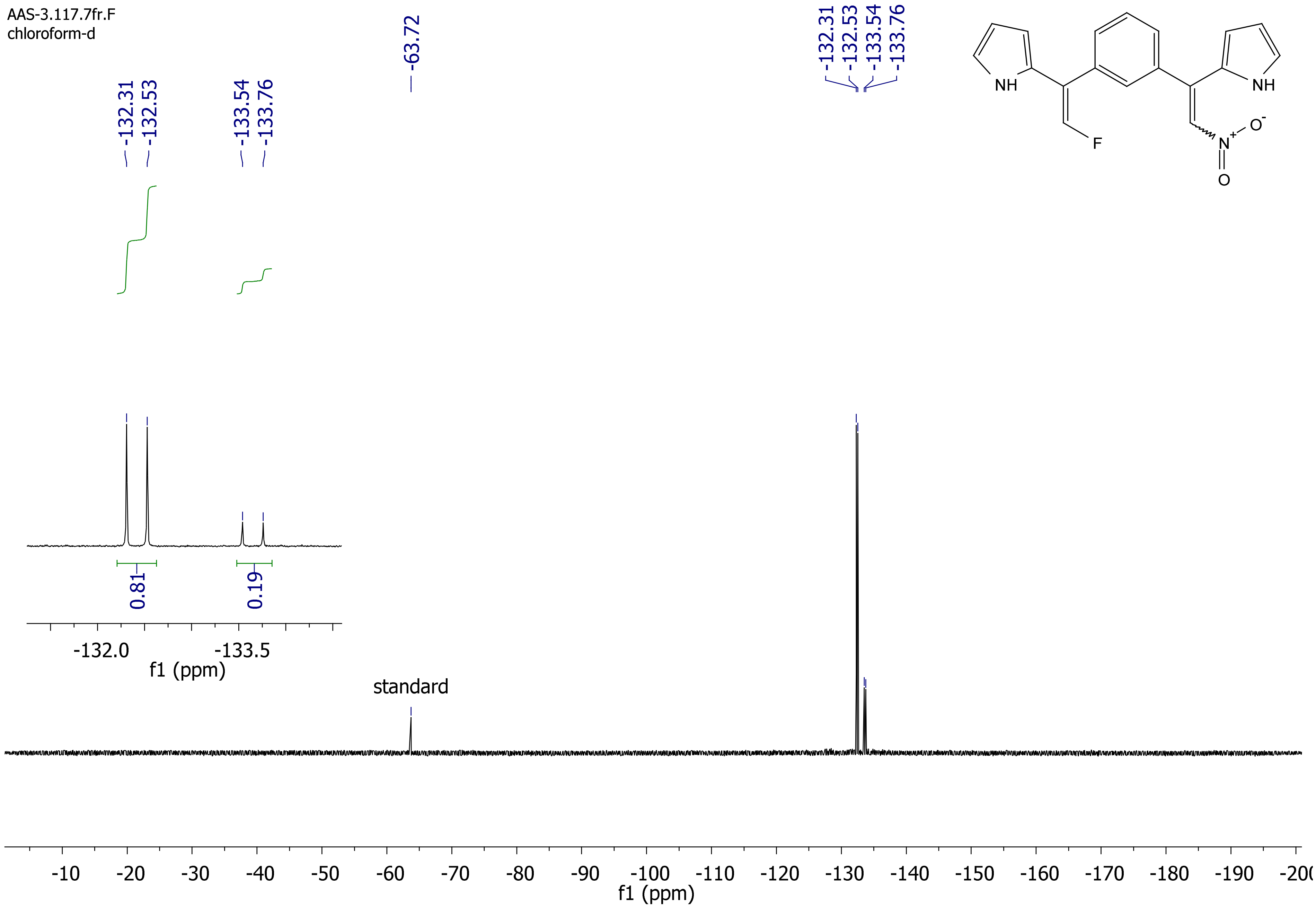

^{19}F NMR spectrum of 2-(1-(3-((*E*)-2-fluoro-1-(1*H*-pyrrol-2-yl)vinyl)phenyl)-2-nitrovinyl)-1*H*-pyrrole (*E*-F-**5o**)

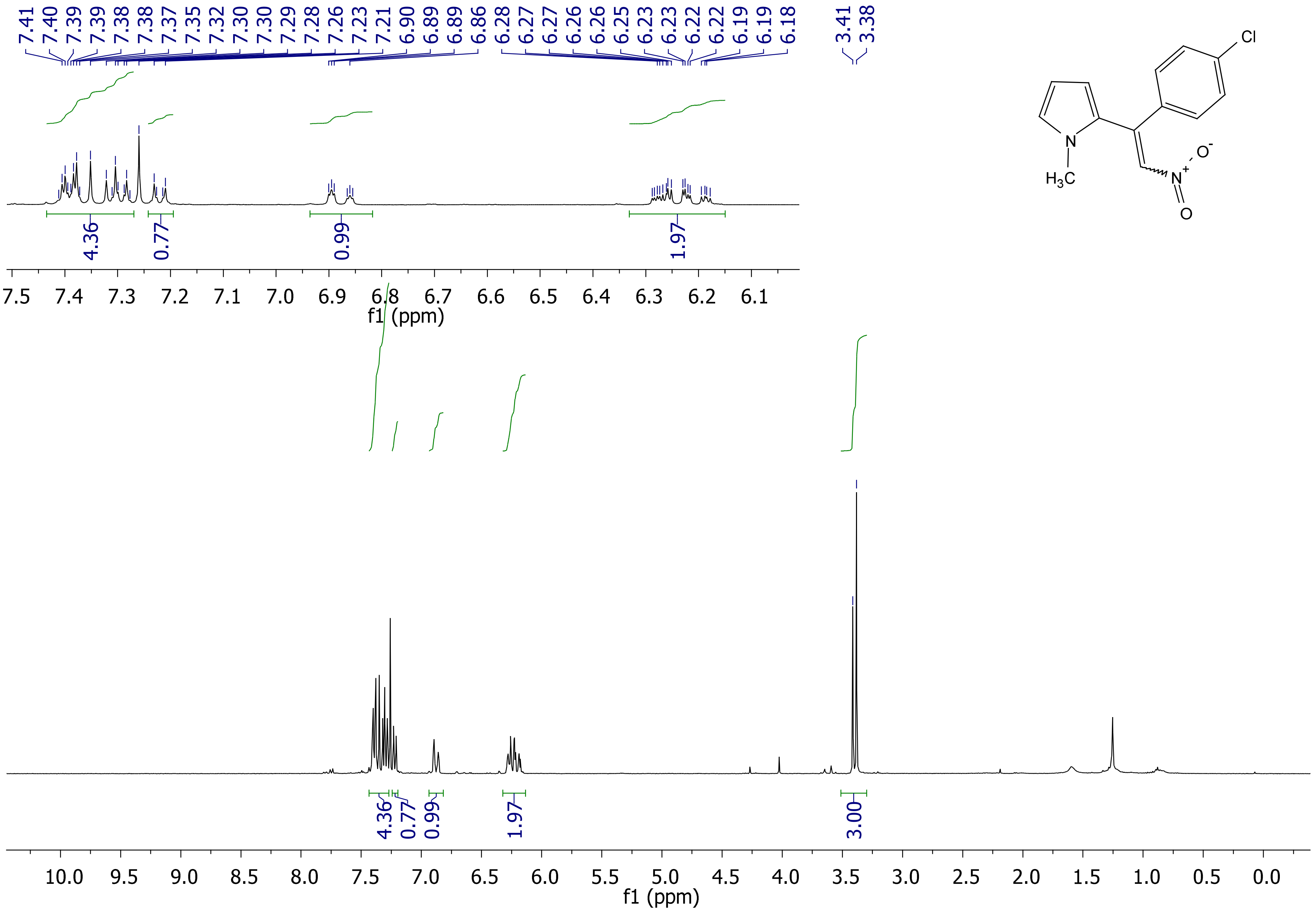

^{1}H NMR spectrum of 2-(1-(4-chlorophenyl)-2-nitrovinyl)-1-methyl-1*H*-pyrrole (**5p**)

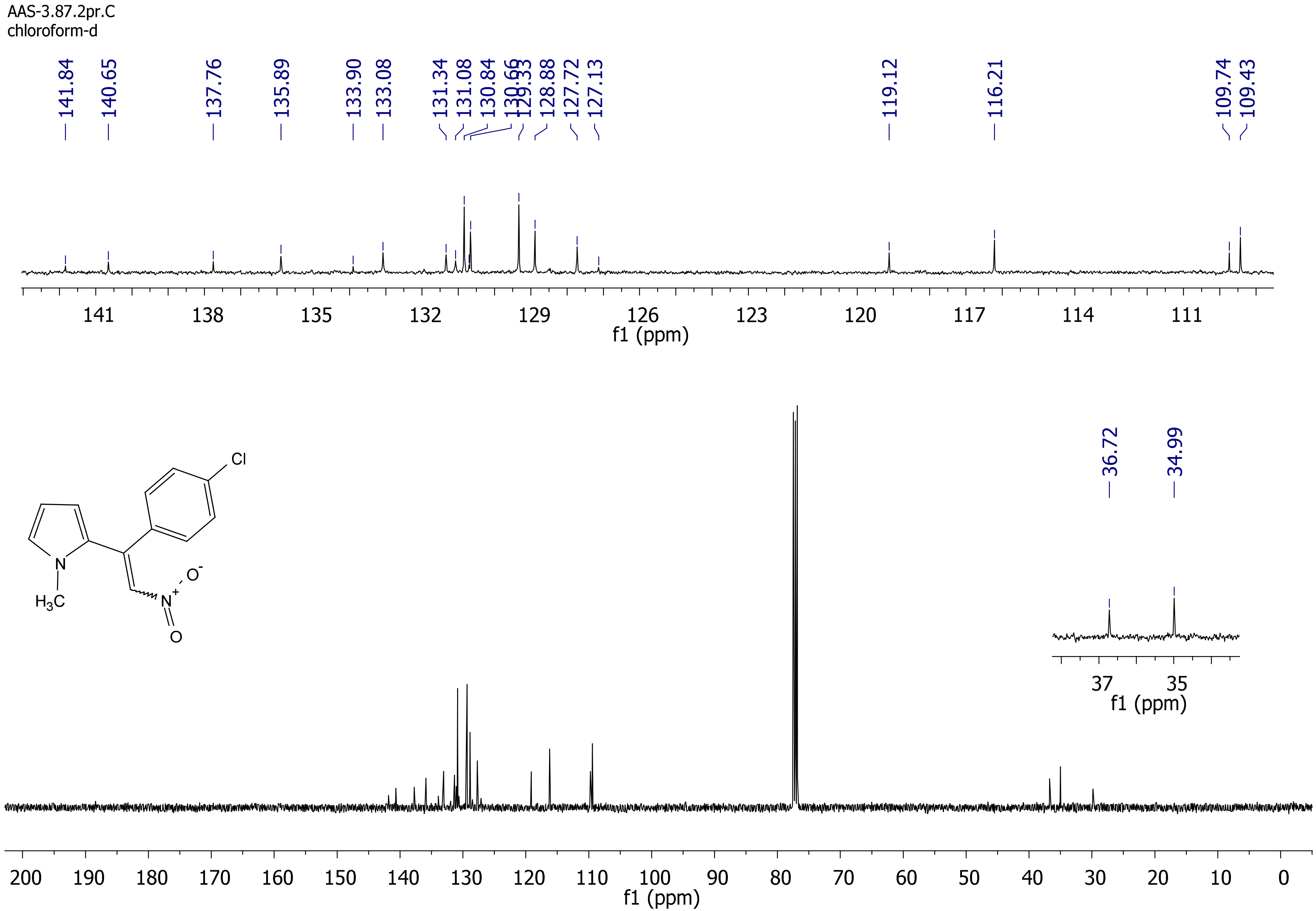

^{13}C NMR spectrum of 2-(1-(4-chlorophenyl)-2-nitrovinyl)-1-methyl-1*H*-pyrrole (**5p**)

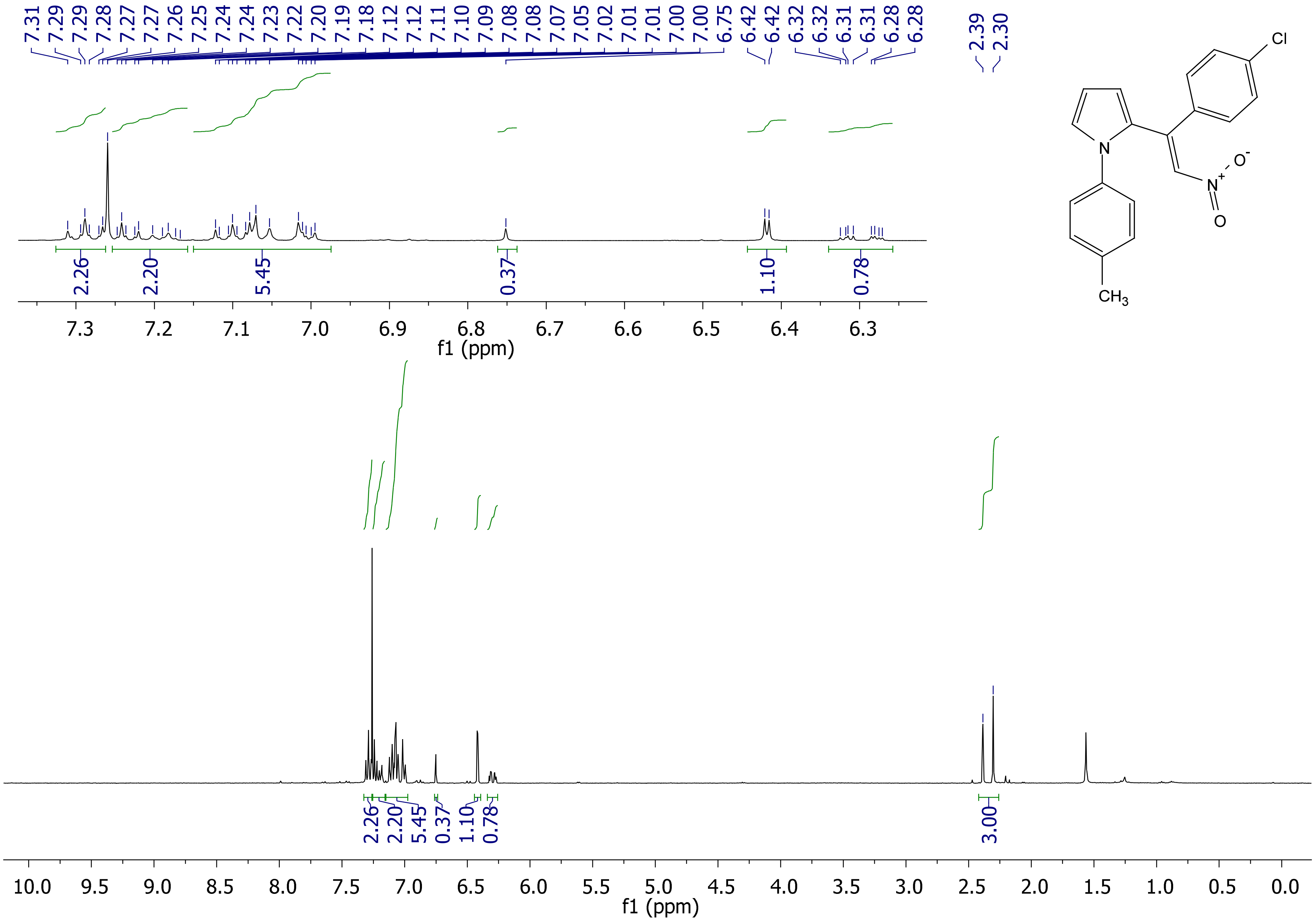

^{1}H NMR spectrum of 2-(2-nitro-1-(p-tolyl)vinyl)-1*H*-pyrrole (**5s**)

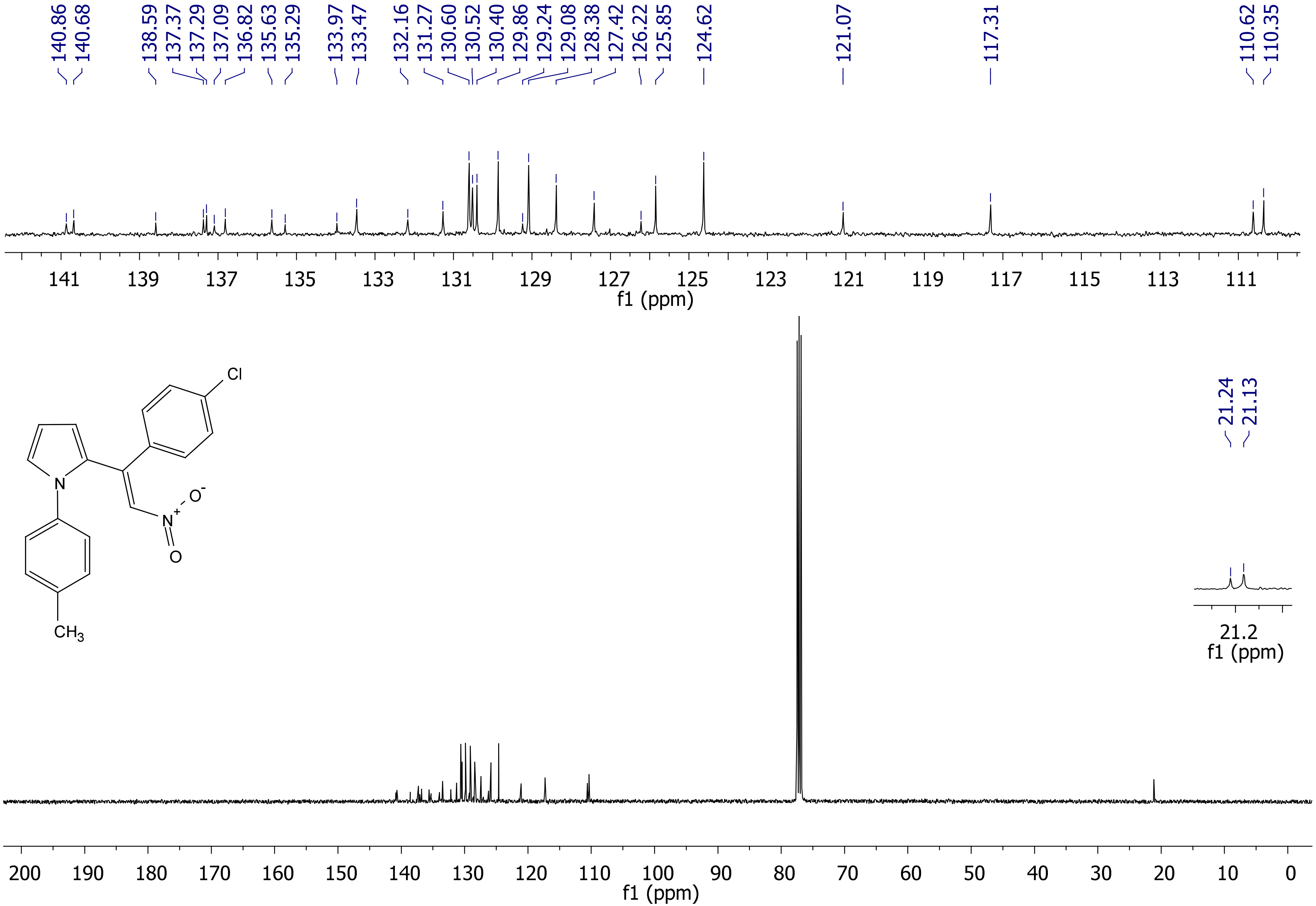

^{13}C NMR spectrum of 2-(2-nitro-1-(p-tolyl)vinyl)-1*H*-pyrrole (**5s**)

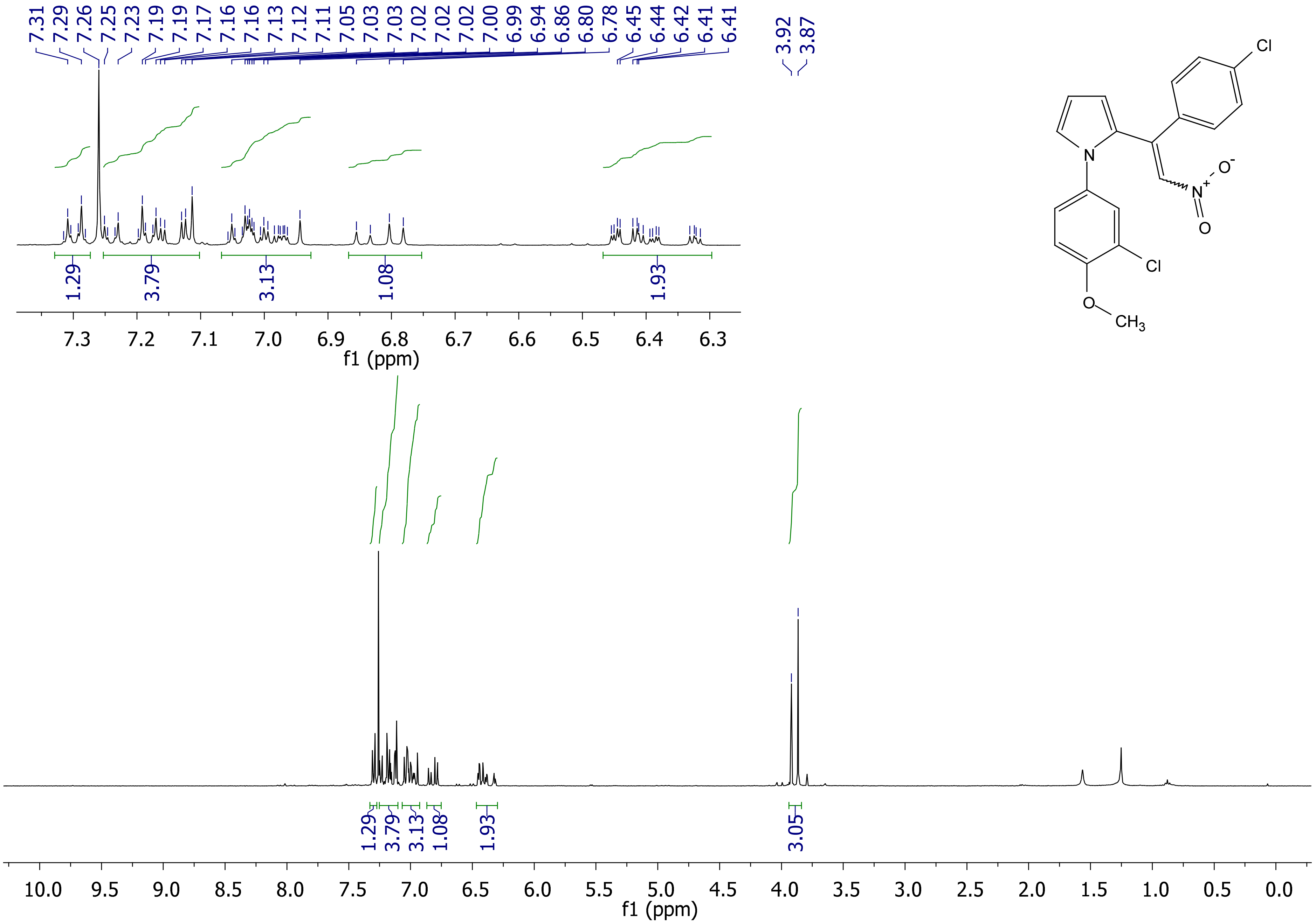

^{1}H NMR spectrum of 1-(3-chloro-4-methoxyphenyl)-2-(1-(4-chlorophenyl)-2-nitrovinyl)-1*H*-pyrrole (**5t**)

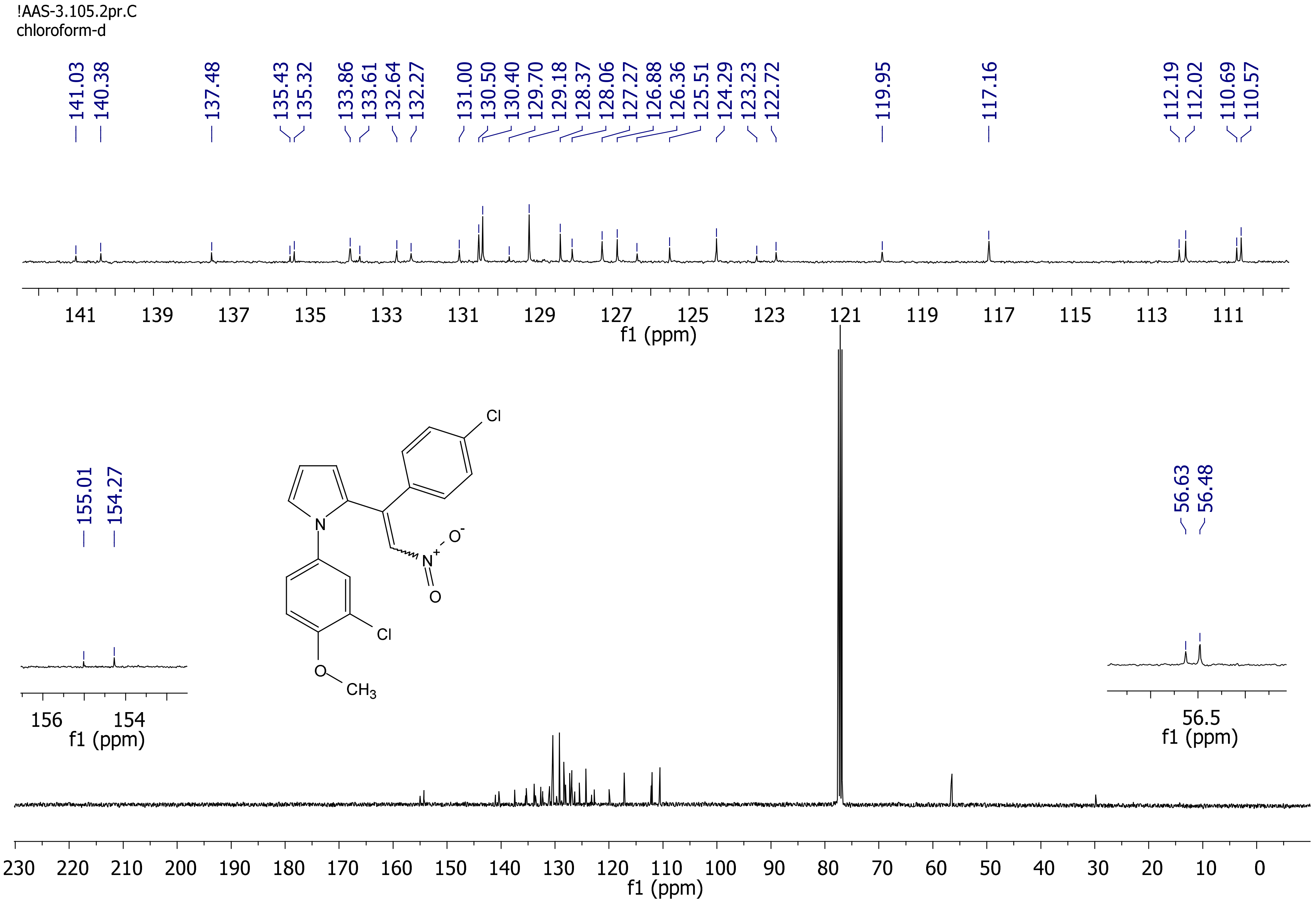

^{13}C NMR spectrum of 1-(3-chloro-4-methoxyphenyl)-2-(1-(4-chlorophenyl)-2-nitrovinyl)-1*H*-pyrrole (**5t**)

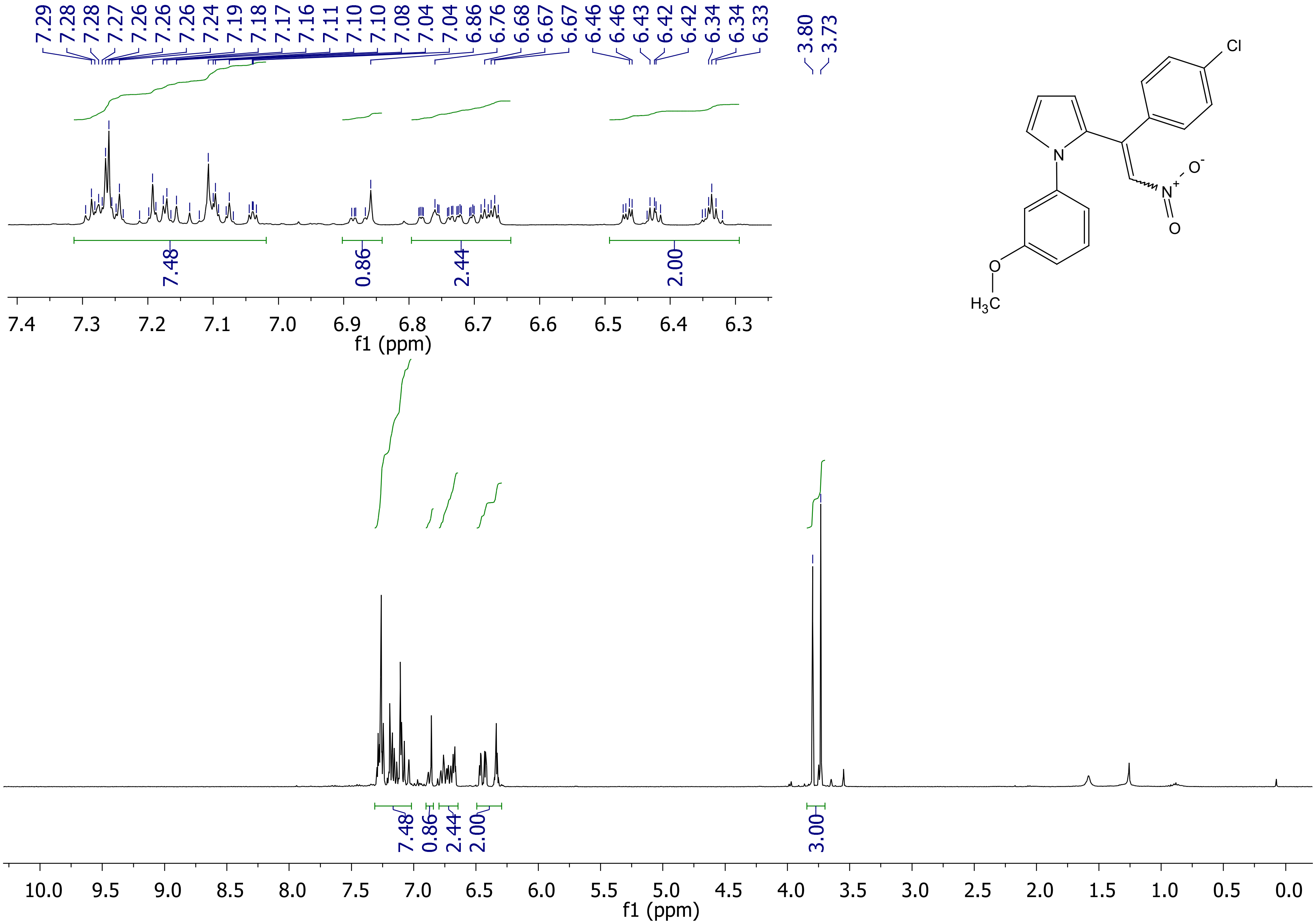

^{1}H NMR spectrum of 2-(1-(4-chlorophenyl)-2-nitrovinyl)-1-(3-methoxyphenyl)-1*H*-pyrrole (**5u**)

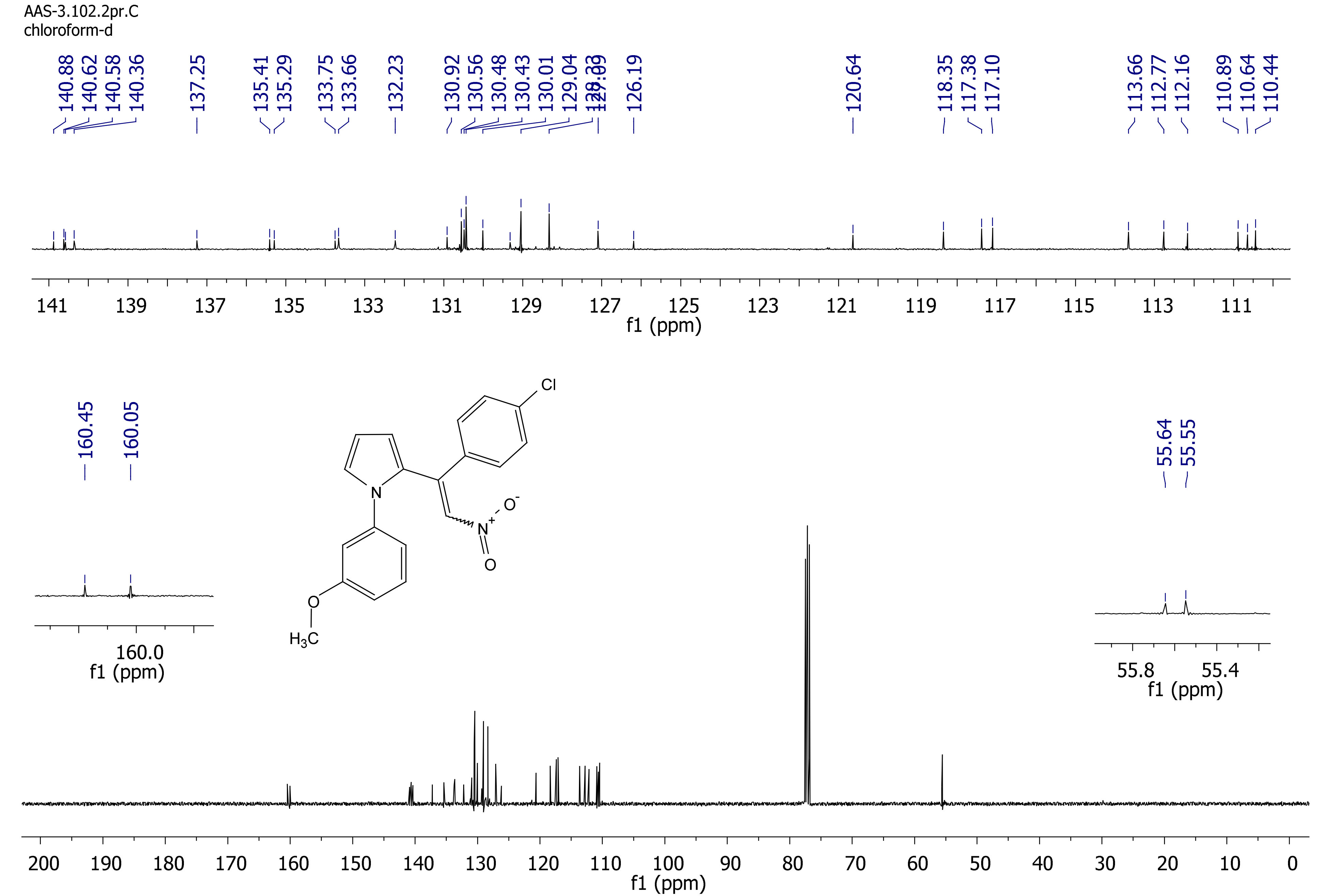

^{13}C NMR spectrum of 2-(1-(4-chlorophenyl)-2-nitrovinyl)-1-(3-methoxyphenyl)-1*H*-pyrrole (**5u**)

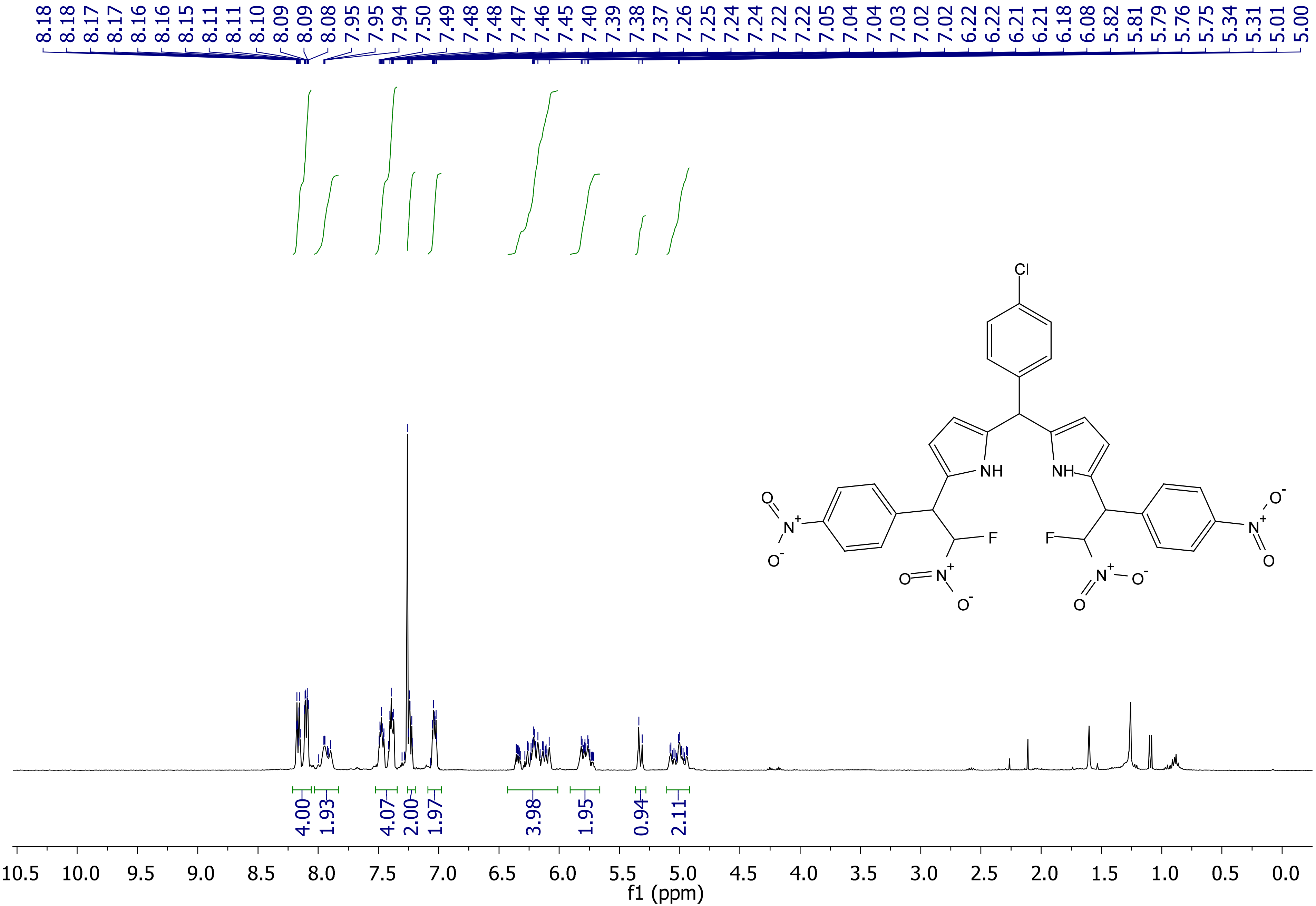

^{1}H NMR spectrum of 5,5'-((4-chlorophenyl)methylene)bis(2-(2-fluoro-2-nitro-1-(4-nitrophenyl)ethyl)-1*H*-pyrrole) (**6**)

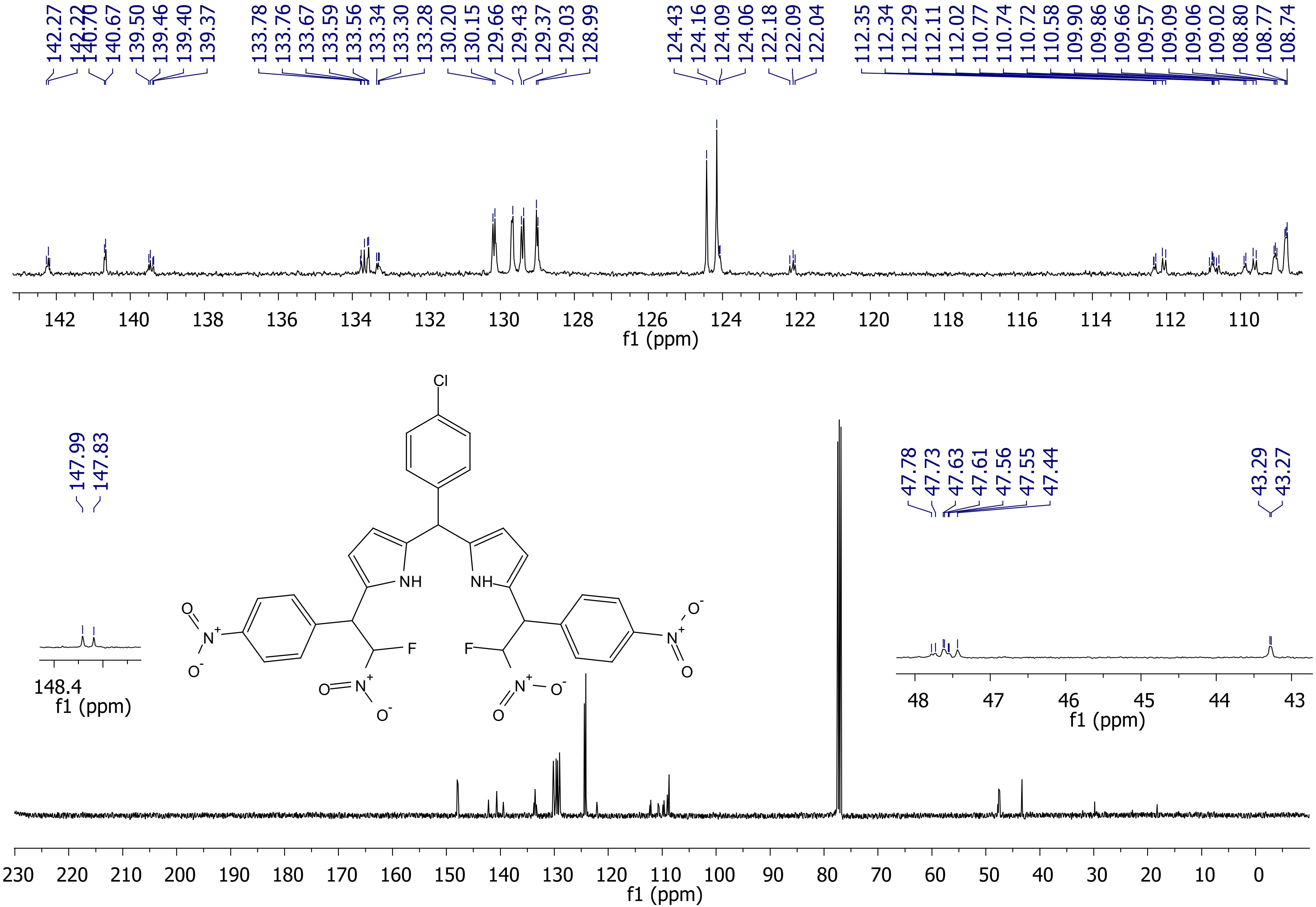

^{13}C NMR spectrum of 5,5'-((4-chlorophenyl)methylene)bis(2-(2-fluoro-2-nitro-1-(4-nitrophenyl)ethyl)-1*H*-pyrrole) (**6**)

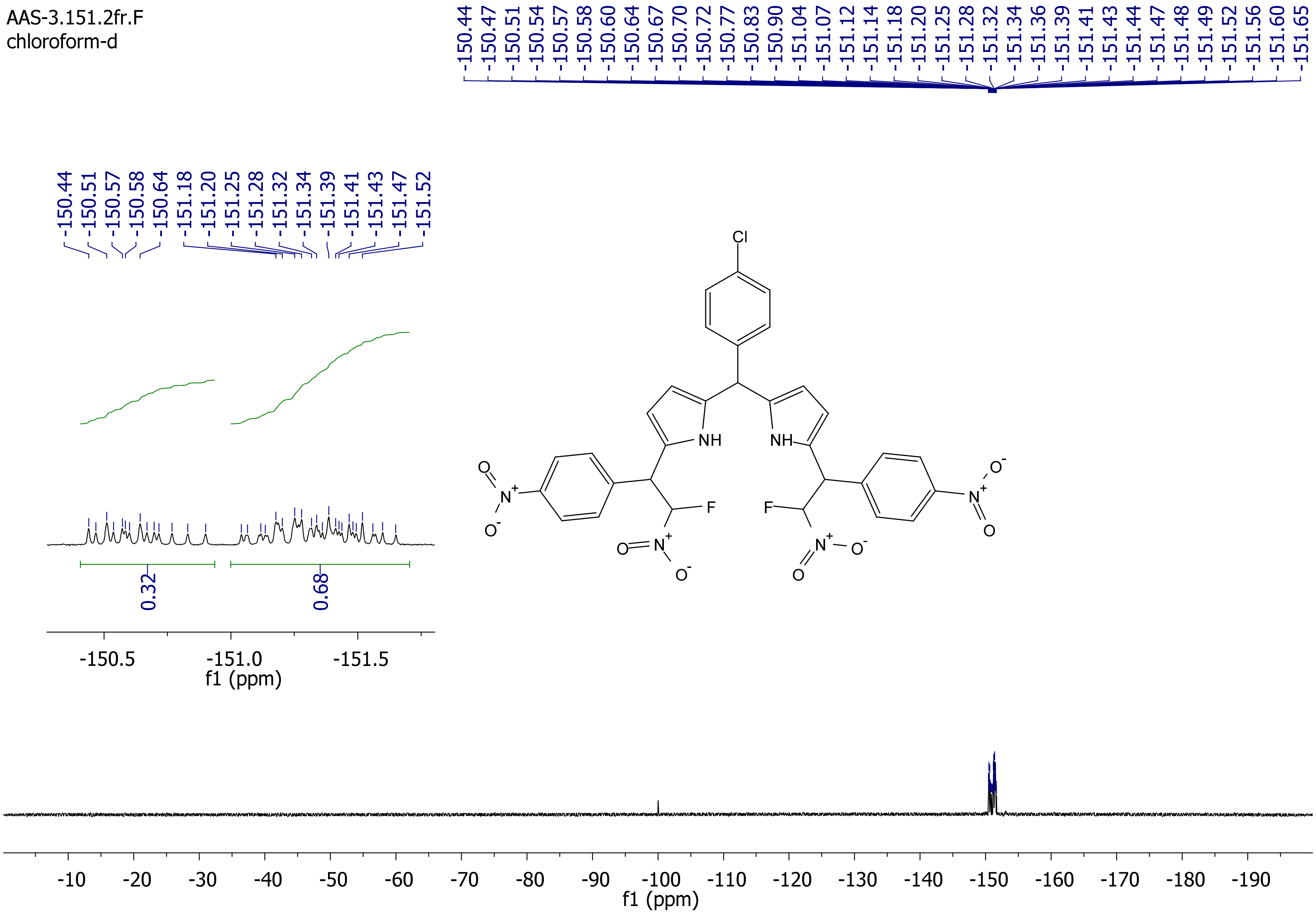

^{19}F NMR spectrum of 5,5'-((4-chlorophenyl)methylene)bis(2-(2-fluoro-2-nitro-1-(4-nitrophenyl)ethyl)-1*H*-pyrrole) (**6**)
